# Premedication practices for delivery room intubations in premature infants in France: Results from the EPIPAGE 2 cohort study

**DOI:** 10.1371/journal.pone.0215150

**Published:** 2019-04-10

**Authors:** Elizabeth Walter-Nicolet, Emilie Courtois, Christophe Milesi, Pierre-Yves Ancel, Alain Beuchée, Pierre Tourneux, Valérie Benhammou, Ricardo Carbajal, Xavier Durrmeyer

**Affiliations:** 1 Medicine and Neonatal Intensive Care Unit, Saint Joseph Hospital, Paris, France; 2 Paediatric Emergency Department. Trousseau Hospital, Assistance Publique – Hôpitaux de Paris, Paris, France; 3 Paediatric and Neonatal Intensive Care Unit, University Hospital Arnaud de Villeneuve, Montpellier, France; 4 INSERM, U1153, Epidemiology and Statistics Sorbonne Paris Cité Research Center, Obstetrical, Perinatal and Pediatric Epidemiology Team, Paris, France; 5 Paris Descartes University France, Paris, France; 6 URC - CIC P1419, Cochin Hotel-Dieu Hospital, Assistance Publique – Hôpitaux de Paris, Paris, France; 7 Division of Neonatology and CIC-1414, Department of Pediatrics, University Hospital, Rennes, France; 8 LTSI, Inserm U1099, Université de Rennes 1, Rennes, France; 9 Neonatal and Paediatric Intensive Care Unit, University hospital, Amiens, France; 10 PériTox - UMI 01, Medicine University, Picardie Jules Verne University, Amiens, France; 11 Paediatric and Neonatal Intensive Care Unit, Trousseau Hospital, Assistance Publique – Hôpitaux de Paris, Paris, France; 12 Neonatal Intensive Care Unit, Centre Hospitalier Intercommunal de Créteil, University Paris Est Créteil, Créteil, France; University of Oklahoma, UNITED STATES

## Abstract

**Objectives:**

To assess premedication practices before tracheal intubation of premature newborns in the delivery room (DR).

**Study design:**

From the national population-based prospective EPIPAGE 2 cohort in 2011, we extracted all live born preterms intubated in the DR in level-3 centers, without subsequent circulatory resuscitation. Studied outcomes included the rate and type of premedication, infants’ and maternities’ characteristics and survival and major neonatal morbidities at discharge from hospital. Univariate and multivariate analysis were performed and a generalized estimating equation was used to identify factors associated with premedication use.

**Results:**

Out of 1494 included neonates born in 65 maternities, 76 (5.1%) received a premedication. Midazolam was the most used drug accounting for 49% of the nine drugs regimens observed. Premedicated, as compared to non premedicated neonates, had a higher median [IQR] gestational age (30 [28–31] vs 28 [27–30] weeks, p<10^−3^), median birth weight (1391 [1037–1767] vs 1074 [840–1440] g, p<10^−3^) and median 1-minute Apgar score (8 [6–9] vs 6 [3–8], p<10^−3^). Using univariate analyses, premedication was significantly less frequent after maternal general anesthesia and during nighttime and survival without major morbidity was significantly higher among premedicated neonates (56/73 (81.4%) vs 870/1341 (69.3%), p = 0.028). Only 10 centers used premedication at least once and had characteristics comparable to the 55 other centers. In these 10 centers, premedication rates varied from 2% to 75%, and multivariate analysis identified gestational age and 1-minute Apgar score as independent factors associated with premedication use.

**Conclusion:**

Premedication rate before tracheal intubation was only 5.1% in the DR of level-3 maternities for premature neonates below 34 weeks of gestation in France in 2011 and seemed to be mainly associated with centers’ local policies.

## Introduction

Tracheal intubation is commonly performed in the delivery room (DR) for premature neonates. This procedure is associated with many adverse events such as bradycardia, hypoxia and increased intracranial pressure [1–4]. Awake intubation is especially associated with increased intracranial pressure, that can be partially prevented by premedication in neonates [1, 2]. In the Neonatal Intensive Care Unit (NICU), adverse tracheal intubation–associated events might be reduced by premedication use, especially paralysis [5]. In addition, an association was reported between the number of intubation attempts in the first 4 days of life and severe intra-ventricular hemorrhage (IVH) [6]. This study’s conclusion speculated that premedication might have a protective effect against IVH, but no evidence was provided. Finally tracheal intubation is painful [3, 7] but is paradoxically a procedure for which analgosedation is not systematically administered [7–9]. On the other hand, the transitional period at birth might expose neonates to a higher risk of adverse drug reactions in case of premedication. Altogether these observations establish a clinical equipoise concerning the use of premedication for non-emergent DR intubations. Currently, the American Academy of Pediatrics (AAP) recommends systematic premedication for neonatal intubation except for “resuscitation in the DR or for life-threatening situations”, based on available evidence and ethical considerations [3]. Those recommendations may theoretically be applied whatever the location of a patient, if all safety rules are gathered. Nevertheless, no clear guidance is provided for non-emergent intubations in the DR. This is probably due to the limited data available on the topic. Indeed, most observational studies on DR intubation did not mention premedication use [10] or reported a premedication rate ranging from 0% to 26%, varying with gestational age (GA) categories [5, 6, 11]. However, recent studies demonstrated the feasibility and suggested analgesic efficacy of premedication before DR intubation [12–14]. Considering that the DR setting was obviously different from the NICU setting, we decided to conduct a specific study on premedication practices in the DR. The aim of our study was to analyze the practices of premedication before tracheal intubation of premature infants’ in the DR of level-3 maternity-units, to describe outcomes before hospital discharge and to identify variables associated with premedication use at a national level using the French EPIPAGE 2 cohort [15].

## Methods

### Study cohort and design

We conducted a retrospective analysis of prospectively collected data from the EPIPAGE 2 study. EPIPAGE 2 is a population-based prospective study that recruited subjects between March and December 2011 in all the maternity hospitals (levels 1, 2 and 3) in 25 French regions. This study’s detailed design was previously described [15, 16]. Briefly this cohort included all infants live born or stillborn and all terminations of pregnancy between 22 and 31 completed weeks of gestation (WG) and a sample of moderate preterm births between 32 and 34 WG. The number of infants required according to the initial sample size calculations was provided by an 8-month recruitment period for births at 22 through 26 WG, a 6-month period for 27 through 31 WG, and a 5-weeks period for 32 through 34 WG [16]. Recruitment periods for births at 22 through 26 weeks occurred between March 28 and December 31 2011; for births at 27 through 31 weeks between March 28 and October 30 2011; for births at 32 through 34 weeks between March 28 and June 5 2011, with different time windows between regions [16].

Our study included all live born preterm neonates from level-3 maternity hospital in the EPIPAGE 2 study who were intubated in the DR. A level-3 maternity hospital in France is equipped with delivery rooms, dedicated neonatal resuscitation room and neonatal intensive care unit corresponding to the levels of neonatal care IIIB, IIIC or IIID defined by the AAP, i.e. allowing perinatal care of all risk profiles and gestational ages, with no restriction on type or duration of mechanical ventilation. We excluded infants with DR intubations performed in level 1 and 2 centers, which are less frequent. Intubations in these centers can be performed by local teams who are not familiar with neonatal intensive care, or by pediatric transportation teams. We considered that these contexts were particular and might not be comparable to level-3 centers’ NICUs practices. As some life-threatening situations can contraindicate premedication [3], we excluded infants who received chest compression or epinephrine in the DR.

### Data collection and definitions

A premedication was defined as the use of a sedative and/or an analgesic before any tracheal intubation. The use of atropine alone was excluded.

At birth and during the neonatal period, data were collected from medical records and completed by questionnaires filled by obstetrical and neonatal teams. The following neonatal data available from the EPIPAGE 2 study were used: gender, gestational age at birth, birth weight, intra-uterine growth restriction based on Olsen’s curves [17], mode of delivery, Apgar score and management in the delivery room, including tracheal intubation, age at tracheal intubation, chest compression, epinephrine use and drugs used in the DR. Some data were unavailable to guarantee anonymisation of included subjects: precise date of birth and consequently week-days, weekends, ferial days or bank holidays. We divided 24 hours in 2 periods: “day” (8 am-5.59 pm) and “night” (6 pm-7.59 am), corresponding with the on-duty period.

In hospital outcomes included survival, severe intracranial lesions defined as intraventricular hemorrhage associated with ventricular dilatation (grade III) and intraparenchymal hemorrhage (ie, large unilateral parenchymal hyperdensity or a large unilateral porencephalic cyst) [18] or cystic periventricular leukomalacia (cPVL); stages II and III necrotizing enterocolitis, according to the staging of Bell *et al*. [19]; stage 3 or higher retinopathy of prematurity, according to the international classification [20] and/or laser treatment; and severe bronchopulmonary dysplasia, defined as administration of oxygen for at least 28 days plus need for 30% or more oxygen and/or mechanical ventilatory support or continuous positive airway pressure at 36 weeks’ postmenstrual age [21].

Questionnaires were also distributed to the medical teams of maternities from participating units to collect data on their structural characteristics, organization and practices. A maternity unit was considered as performing a premedication if at least one neonate received a premedication during the study period.

### Practices’ description and analysis

We first described the frequency of premedication prior to DR intubation by gestational age categories and by centers. We then analyzed the drugs or drugs combination used. In order to identify factors associated with premedication use, we performed univariate analysis in the whole population including patients’ and centers’ characteristics. Patients-related variables included in the univariate analysis were perinatal characteristics and the period of birth (day or night) that was previously reported to be associated with analgesic treatments’ use in the NICU [22]. We excluded from this analysis all variables that were potentially or certainly posterior to the intubation, such as 5 min Apgar score or exogenous surfactant administration. Neonatal outcomes at hospital discharge were compared between the premedicated and the non-premedicated groups using univariate analysis.

Centers-related variables included: total number of births in 2011, location of the DR and duration of transportation between the DR and the NICU.

Then, within centers that performed at least one premedication, we used multivariate analysis to identify factors derived from the univariate analysis that were independently associated with premedication use. We excluded from this analysis centers in which premedication was never performed, considering that this policy was due to the center and not to the patients’ characteristics. Limiting the multivariable analysis to the centers that performed at least one premedication was thus more appropriate in order to identify specific patients’ characteristics, independently of the center’s general policy.

### Statistical analysis

Descriptive analyses are presented as means (standard deviations), medians (interquartile ranges), and ranges. Categorical variables are presented as the numbers of individuals (percentages) and 95% confidence intervals. Considering the different recruitment periods for each GA categories, weighting was used to correct disproportional sample sizes and adjust collected data to represent the population from which the sample was drawn. The weighting case was 0.625, 0.75 and 1 for infants born at 22–26 WG, 27–31 WG and 32–34 WG, respectively.

Considering that infants’ management in each center could be influenced by local policies, a generalized estimating equation (GEE) model was used for univariate and multivariate analyses, using each center as the unit of clustering [23]. Variables previously associated in the literature with premedication use in the NICU [9, 24] or showing an association with a *P* value <0.20 in the univariate analyses were included in the model. The results of the GEE model are presented with their odds ratios, and their 95% confidence intervals (95% CI). Survival and major morbidities at hospital-discharge were compared between groups using Rao–Scott F-adjusted chi-square test, as exploratory analysis. Statistical significance was set at *P*<0.05. All statistical analyses were performed with SPSS software (version 17, Chicago, IL) or SAS software (version 9.4).

### Ethics

The National Data Protection Authority (CNIL n° 911009), the consultative committee on the treatment of information on personal health data for research purposes (Reference number 10.626) and the committee for the protection of people participating in biomedical research (reference CPP SC-2873) approved this study. These 2 committees approved any analysis of the collected data. The parents of the participants provided their oral consent to participate in this study. Since this study was observational, according to the French legislation, oral consent to anonymously collect data was obtained from the parents. In case the parents expressed their opposition to data collection, the neonates were excluded from the study (around 7% of eligible patients in the main study [15]). The oral agreement to participate in the study was written in the medical records of the mothers and the newborns included. If the consent was not obtained or if the patients refused, it was also tracked in the medical charts and the data were not recorded. The ethics committees approved this consent procedure.

## Results

### Population

**Fig 1:**
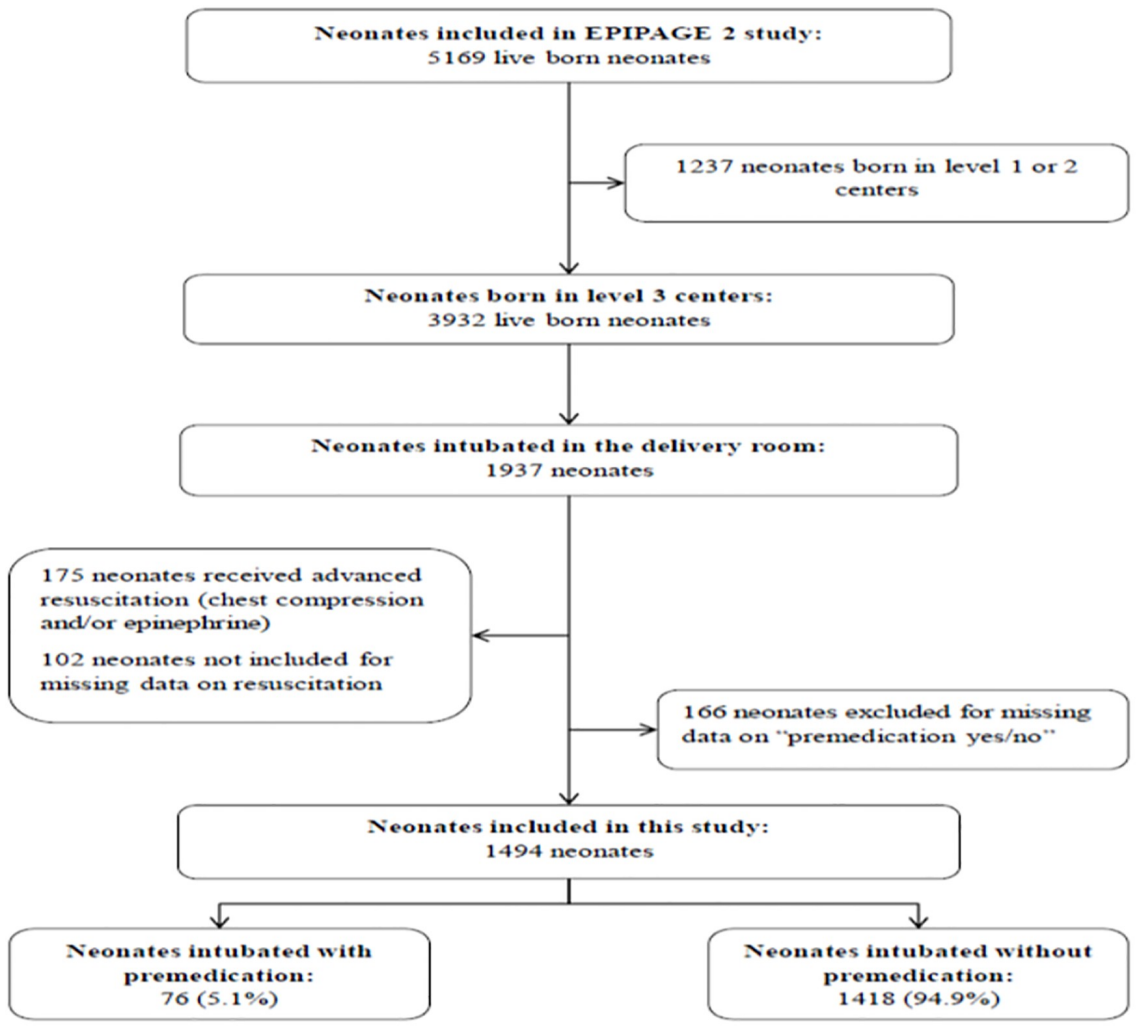
Population flow chart.

The initial EPIPAGE 2 cohort included 5169 live births. Among the 1937 neonates born in a level-3 center and intubated in the DR, 1494 (77.1%) were included in the study (Fig 1). The 166 neonates with missing data on premedication had statistically significantly higher birth weights and GAs than included infants (1254 g vs 1183 g and 28.9 WG vs 28.5 respectively), but these differences were clinically irrelevant. Other perinatal characteristics (sex, intra uterine growth restriction, mode of delivery, maternal general anesthesia, one minute Apgar score, exogenous surfactant in the delivery room, age at intubation and period of birth) were comparable between these 2 populations.

**Fig 2:**
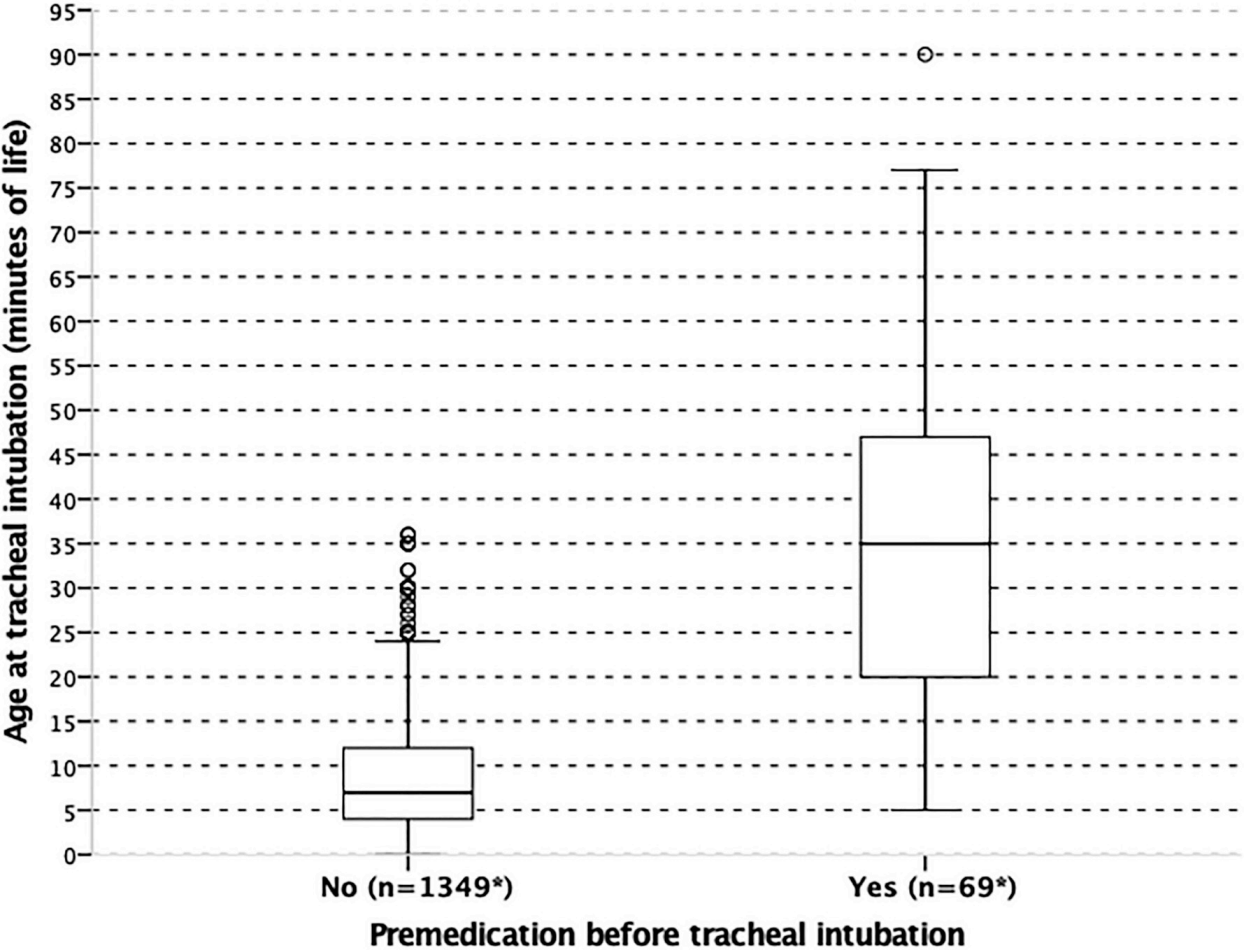
Distribution of age (minutes of life) at tracheal intubation in the delivery room according to premedication. Boxes represent values between the 1^st^ and the 3^rd^ quartile. The bar inside the box denotes median value. The adjacent values are the most extreme values within 1.5 inter-quartile range of the nearer quartile. All results (except n) are weighted to take into account differences in the sampling process between gestational age groups.

The rate of premedication for tracheal intubation was 5.1% (76/1494): 5/493 (1%) of neonates born between 22 and 26 WG, 67/959 (7%) of those born between 27 and 31 WG and 4/42 (9.5%) of those between 32 and 34 WG. Distribution of age at tracheal intubation in the premedicated and non-premedicated groups is illustrated in Fig 2. Median [IQR] age at intubation was significantly lower in the non-premedicated group as compared to the premedicated group (7 [4–12] *vs* 35 [20–47] minutes of life respectively; p<0.001). Overall 757/1467 (52%) of the population received exogenous surfactant in the DR.

Neonates were born in 65 level-3 maternities of which 10 (15.4%) used a premedication before tracheal intubation in the DR. In these 10 centers, the premedication rate varied from 2.1 to 75.0% (Table 1). Four centers performed 86.8% of all premedications.

**Table 1:** Premedication rates and number of births in 2011 in the 10 centers that performed at least one premedication.

Maternity units	Premedication rate (n premed/n intubations)	Number of births in 2011
A	75.0% (15/20)	4125
B	62.5% (20/32)	3426
C	25.9% (15/58)	5039
D	23.9% (16/67)	2989
E	16.7% (1/6)	1633
F	9.1% (2/22)	2805
G	9.1% (2/22)	3131
H	7.7% (3/39)	2346
I	2.2% (1/46)	4824
J	2.1% (1/48)	2725
Total	21.1% (76/360)	

### Drugs used for premedication

**Fig 3:**
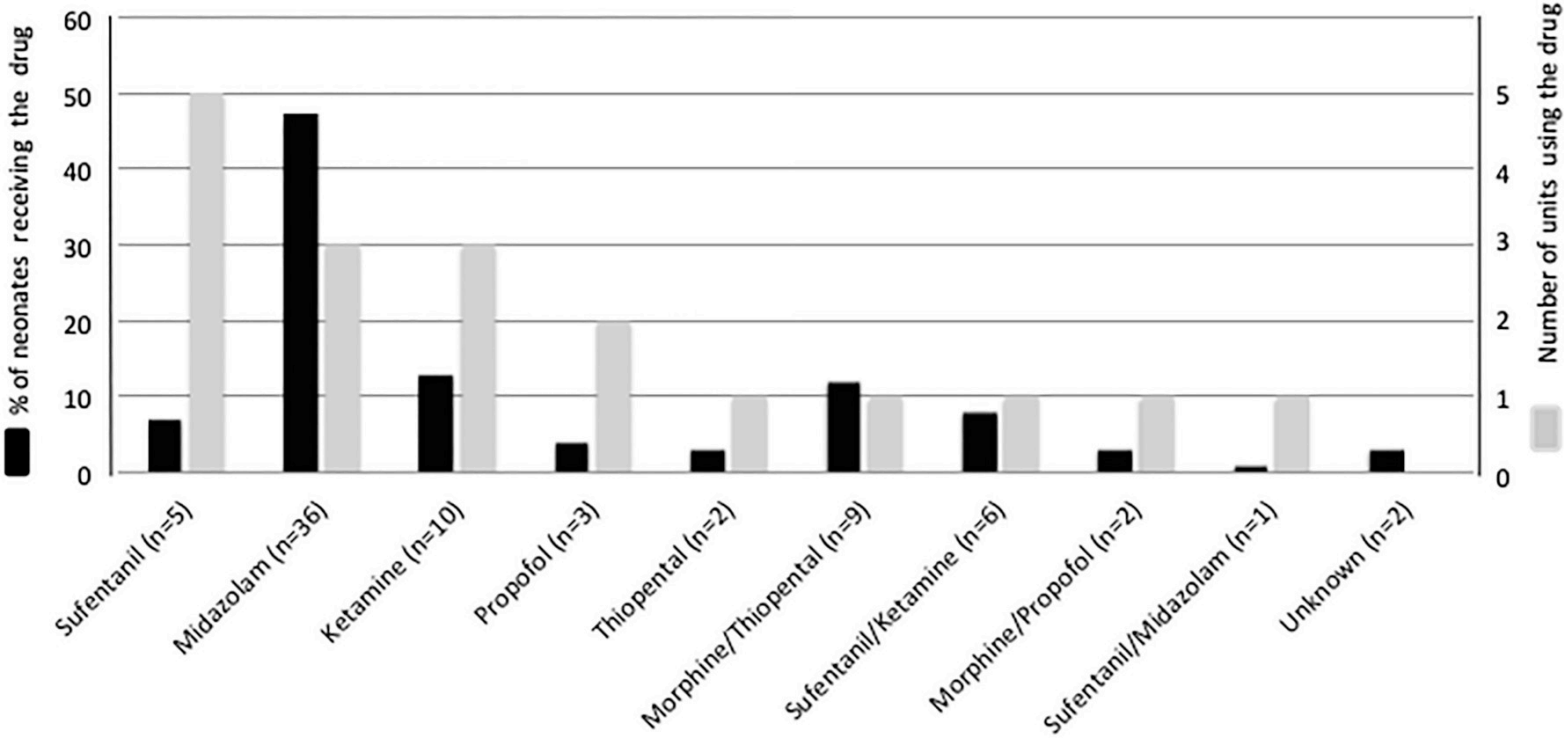
Drug(s) regimens used for premedication before tracheal intubation in the delivery room. The X axis includes all used premedication regimens with the number of patients in parenthesis. The black bars indicate the percentage of infants who received each drug or drugs combination within the premedicated group. The corresponding Y-axis is on the left side of the graph. The grey bars indicate the number of centers that used each drug or drugs combination at least once. The corresponding Y-axis is on the right side of the graph.

Nine different drugs regimens were used to perform premedication in the DR (Fig 3). In the 76 premedicated neonates, 37 (48.7%) received midazolam, 16 (21.1%) received ketamine, 12 (15.8%) received sufentanil, 11 (14.5%) received morphine, 11 (14.5%) received thiopental and 5 (6.6%) received propofol, alone or in association.

### Univariate analysis

Univariate analysis comparing premedicated with non-premedicated neonates is presented in Table 2. As compared to non-premedicated infants, premedicated infants had higher GA, birth weight and 1 min Apgar scores; their mother less frequently received general anesthesia.

**Table 2:** Population characteristics and weighted univariate analysis of risk factors associated with premedication use.

Characteristics (n with available data)	Total (n = 1494)	Weighted univariate analysis^a^		
		Premedication (n = 76)	No premedication (n = 1418)	P-value^b^
**Sex** No. (%) (n = 1494)				
**Male**	803 (53.7)	48 (56.1)	755 (55.3))	0.860
**Female**	691 (46.3)	28 (43.9)	663 (44.7)	
**GA** (weeks) (n = 1494)				
Mean (SD)	27.8 (2.2)	29.7 (2.1)	28.5 (2.5)	<0.01
Median [IQR]	28.0 [26.0–29.0]	30 [28–31]	28 [27–30]	<0.01
Range	23–34	25–34	23–34	
**Birth weight** (g) (n = 1494)				
Mean (SD)	1070 (362)	1432 (464)	1167 (430)	< .001
Median [IQR]	1000 [795–1300]	1391 [1037–1767]	1074 [840–1440]	< .001
Range	370–2610	620–2290	370–2610	
**Mode of delivery “vaginal”** (n = 1486), No. (%)	546 (36.7)	20 (69.7)	526 (64.9)	0.280
**Maternal general anesthesia** (n = 1448), No. (%)	217 (15.0)	7 (7.4)	210 (15.4)	0.016
**Intrauterine Growth Restriction** (n = 1494), No. (%)	208 (13.9)	9 (9.7)	199 (14.5)	0.136
**1 min Apgar score** (n = 1462)				
Median [IQR]	6 [3–8]	8 [6–9]	6 [3–8]	< .001
Range	0–10	1–10	0–10	
**Exogenous surfactant in the DR** (n = 1467), No (%)	757 (51.6)	53 (66.1)	704 (45.8	<0.001
**Period of birth “Day”** (n = 1439), No. (%)	679 (47.2)	41 (59.7)	638 (48.1)	0.014

^a^ All results (except n) are weighted to take into account differences in the sampling process between GA groups.

^b^ Mann-Whitney’s, t-test or chi square tests for comparisons between premedicated and non-premedicated infants.

Outcomes at discharge from hospital of neonates who received a premedication were comparable or better than those who did not, although the occurrence of major neonatal morbidities was scarse (Table 3).

**Table 3:** Univariate analysis of survival and major neonatal morbidities^1^ between premedicated and non-premedicated neonates.

Outcomes at hospital discharge ^a^	Premedication (n = 76)	No premedication (n = 1418)	P-value
Survival No (%)	68/76 (91.5)	1200/1418 (87)	0.22
Survival without severe mordidity^b^ No (%)	56/73 (81.4)	870/1341 (69.3)	0.028
Among survivors^b^			
Severe cerebral lesion No (%)	7/65 (9.1)	80/1189 (6.1)	0.30
Necrotizing enterocolitis No (%)	2/67 (2.1)	44/1186 (3.9)	0.39
Severe bronchopulmonary dysplasia No (%)	1/67 (1.2)	143/1146 (10.1)	0.008
Severe retinopathy of prematurity No (%)	0/59	23/942	0.17

^a^ n are crude and % are weighted

^b^ Denominators are lower than the total number of patients in each group, due to missing data

Severe morbidity was defined as severe bronchopulmonary dysplasia, necrotizing enterocolitis, severe retinopathy of prematurity or any of the following severe cerebral abnormalities on cranial ultrasonography: intraventricular hemorrhage with ventricular dilatation or intraparenchymal hemorrhage, or cystic periventricular leukomalacia.

Univariate analysis also compared the 10 centers which performed a premedication with the 54 others (1 center did not provide organizational data). All parameters included in the analysis were comparable (centers with premedication vs no premedication): mean (SD) total number of births in 2011 (3004 (1077) *vs* 3031 (887), p = 0.390), place of DR and NICU (same building: 77.8% *vs* 83.3%, p = 0.650), transportation time between DR and NICU (< 5 minutes: 88.9% vs 85.2%, p = 1.000).

### Factors associated with premedication in centers where premedication was used

The multivariate analysis of neonatal factors associated with a premedication across centers that performed at least one premedication is shown in Table 4. Only GA and 1 minute Apgar score were significantly associated with the use of premedication in the DR for preterm neonates: neonates between 22 and 26 WG had lower probability to receive a premedication than late preterm neonates (32–34 WG) (aOR (95% CI), 0.10 (0.03–0.42)), whereas neonates with a higher 1-minute Apgar score had higher probability to receive premedication (aOR (95% CI), 1.14 (1.05–1.25)) (Table 4).

**Table 4:** Multivariate analysis of neonatal factors associated with a premedication across centers that performed at least one premedication in the DR.

Risk factors (n with available data)	No. of neonates	Crude analysis (n = 360)				Multivariate analysis (n = 335)	
		Premedicated (n = 76)	Non-premedicated (n = 284)	OR (95% CI)	*P*-value	Adjusted OR (95% CI)	*P-*value
**GA categories** (weeks) No. (%) (n = 360)							
32–34	13	4 (30.8)	9 (69.2)	Reference		Reference	
27–31	250	67 (26.7)	183 (73.3)	0.82 (0.24–2.86)	0.760	0.69 (0.20–2.27)	0.519
22–26	97	5 (5.2)	92 (94.8)	0.12 (0.02–0.66)	0.015	0.10 (0.03–0.42)	0.001
**IUGR**, No. (%)^a^ (n = 360)							
No	318	67 (24.7)	251 (75.3)	Reference		Reference	
Yes	42	9 (16.7)	33 (83.3)	0.84 (0.57–1.25)	0.389	0.77 (0.37–1.28)	
**1 min Apgar score** ^b^ (n = 353)	353	7.3 (2.6)	5.3 (3.0)	1.16 (1.07–1.26)	< .001	1.14 (1.05–1.25)	0.002
**Maternal general anesthesia**, No. (%)^a^ (n = 349)							
**No**	299	68 (25.9)	231 (74.1)	Reference		Reference	
**Yes**	50	7 (13.0)	43 (87.0)	0.52 (0.18–1.50)	0.225	0.57 (0.20–1.64)	0.295
**Period of birth**, No. (%)^a^ (n = 352)							
Night	192	32 (17.6)	160 (82.4)	Reference		Reference	
Day	160	41 (29.3)	119 (70.7)	1.64 (0.59–4.57)	0.349	1.68 (0.57–4.97)	0.347

^a^ n are crude and % are weighted,

^b^ mean (SD) weighted.

Abbreviations: IUGR, intra-uterine growth retardation; OR, odds ratio; CI, confidence interval

## Discussion

To our knowledge, this is one of the first large descriptive studies to prospectively evaluate premedication before tracheal intubation in the DR for preterm neonates. A premedication was performed for about 5% of the selected population, in less than 15% of the level-3 maternity units and with a large variety of drugs regimens. Survival and severe neonatal morbidity at hospital discharge were not increased in premedicated infants. Only two patient’s independent factors, GA and 1 minute Apgar score, were associated with the use of premedication for intubation.

This 5% premedication rate is much lower than in the two previous declarative studies on DR intubations performed in France [25, 26]. In these two survey-based questionnaires, the declared premedication rate was 11% for prophylactic surfactant administration and 20% for all neonates necessitating tracheal intubation in the DR. The results of an observational study, conducted in 2 level-3 maternity units in France, showed that 0 to 25% of the neonates intubated in the DR received a specific premedication [11]. In another single-center retrospective study in the US, 1.1% of infants with birth weight < 1500g who were intubated in the DR received a premedication [6]. In an international multicenter registry 10% of infants intubated in the DR were premedicated [5].

In our study, we observed premedication rates varying from 2 to 75% across centers, revealing a major center effect. Only 10 level-3 maternity units out of 65 performed a premedication in the DR, 4 of them performing about 87% of all premedications. All compared organizational factors were similar between the centers that performed a premedication and those that did not. However, we did not assess the existence of a clinical care team dedicated to the DR or the intervention of the NICU team in the DR, since both policies exist in France. This variable was too complex to collect, since organizations often vary within a same center between day- and night-time, weekdays and holidays. These results may express the prevailing impact of a specific organization and of a center’s local policy for the practice of a premedication before tracheal intubation in the DR. This is supported by the recent report from the NEAR4NEOS registry stating that 1 out of 10 academic centers routinely performed premedication before intubation in the DR [5].

In the present study, at least 6 drugs, alone or in association were used. When confronted to the recommendations for non-emergent intubation, most of these drugs were not recommended or not described [3]. The choice and the optimal dosing for the sedative and/or analgesic drugs for this procedure are not consensual [3, 27]. Midazolam, which was the most frequently used drug in our study, should be used in combination with analgesic agents and is not recommended for premature infants, because of its hypotensive effect [3, 28–31]. We observed though that it was used alone in nearly 50% of premedications in this study, and only once associated with sufentanil. Ketamine was the second most frequently used drug in our study. The use of ketamine is controversial due to its potential apoptotic effects [32, 33], but it has been successfully used in the DR with a significant decrease in pain scores and no short-term adverse effect [12]. In a recent study comparing nasal midazolam and nasal ketamine for neonatal intubation in the DR [14], ketamine was less efficient than midazolam in achieving rapid and adequate sedation, with a comparable and adequate comfort, assessed by the Faceless Acute Neonatal pain Scale [34]. Thiopental was most often used in association with morphine, an association that has never been reported, although experience with thiopental exists [35–37]. Fentanyl, the preferred opioid according to the AAP [3] and the Canadian Paediatric Society [38] was not used in our study, but sufentanil was the most frequently used short-acting opioid, despite limited data in premature neonates [39]. Propofol, which is a preferred option [3] was the less frequently used drug, possibly because hypotension was feared [40, 41]. Overall, heterogeneity in centers’ policies and in drugs used denotes the lack of evidence and underlines the need for research in this field.

In our study, neonates who received a premedication in the DR had a significantly higher morbidity-free survival rate at hospital discharge than those who were not premedicated after univariate analysis. However, the imbalance between groups in baseline characteristics precludes any firm conclusion, since the number of premedicated patients was too limited to perform reliable adjustments for confounders through multivariate analysis. This result, combined with others [5, 6], only confirms the current clinical equipoise concerning premedication use for DR intubation and supports future prospective trials.

Birth weight, maternal general anesthesia, period of birth, 1 minute Apgar score and GA seemed to be associated with the use of a premedication in univariate analysis. Decreased frequency of premedication in the smallest infants has already been observed in the DR [6]. A decrease in neonatal motor activity after maternal general anesthesia has been reported in the first minutes after birth, which is consistent with a decreased use of premedication when intubation is performed [42], The more frequent use of premedication in the day-time is consistent with the difference for pain management between day and night previously observed in a large observational study [22]. In the GEE model, GA and 1-minute Apgar score were the only identified independent factors associated with premedication use in the level-3 maternity units which performed it. The most premature neonates, 22 to 26 WG, were less likely to receive a premedication than those born after 32 WG, as well as neonates with a low 1-minute Apgar score. The association between GA and premedication use has already been observed for intubations performed in the NICU [24], although inconstantly [9]. These results are also consistent with two previous declarative studies conducted in France where neonates under 28 WG never received a premedication, as those with a low Apgar score [11, 26].

Although we excluded infants who required chest compression or epinephrine, some infants might have required intubation as a life-saving procedure because they were not responding to face-mask ventilation. In this case, avoiding premedication was appropriate according to the current recommendations [3]. This might have occurred for 30 to 35% of intubated infants based on previous reports [43, 44]. An alternative approach would have consisted in excluding very early intubations (e.g. before 3 minutes of life), but this might have inadvertently excluded infants who were rapidly intubated only for the purpose of surfactant administration, because of local protocols and/or policies and who were in the scope of our study. On the other hand, around half of intubated infants in our study received surfactant in the DR. This group probably included premature infants who were intubated for initial stabilization and subsequently treated with surfactant, and extremely premature infants who received prophylactic surfactant. This was in accordance with most recent European guidelines in 2011, issued in 2010, which stated that “prophylaxis should be given to almost all babies of <26 WG” or “preterm babies with RDS who require intubation for stabilization” [45]. These infants were not eligible for premedication but their proportion in the whole population of surfactant-treated infants was necessarily limited because our study included infants up to 34 WG. The overall 5% rate of premedication contrasts with high premedication rates in some centers whose characteristics were similar to those of centers that had lower premedication rates. This observation underlines the weight of local policies and supports the feasibility of premedication before DR intubation. The non-premedicated group had a significantly lower age at intubation compared to the premedicated one (7 vs 35 minutes of life) and underwent tracheal intubation very soon after birth. The same result was observed in one of the two observational studies conducted in the DR [11]. This observation can suggest that the non-premedicated babies were the sickest and were not eligible for premedication, whereas time was allowed for the healthiest neonates to probably be stabilized by non-invasive ventilation while preparing the premedication. Nevertheless, we cannot rule out that the time required for premedication’s preparation, administration and effect delayed intubation in the premedicated infants. In a previous observational study in the DR, surfactant administration occurred significantly later after ketamine analgesia compared to no analgesia, but 79.5% of the neonates received surfactant before 30 minutes of life [12]. After nasal midazolam administration, the median delay between sedation and intubation was about 10 minutes [14].

This study has several limitations as it was not designed to specifically assess premedication before neonatal tracheal intubation in the DR. Some items are not documented such as the reason for intubation, the mode of ventilation before tracheal intubation, drugs’ administration routes and dosage, pain scores, intubation conditions, experience of the operator, number of attempts, success rate of first attempt and the existence of a specific protocol for premedication in the DR. We did not explain why such differences for the use of premedication between French DRs were observed, although some factors were previously identified, such as operator’s experience [8], the lack of national guidelines and local protocols [11, 26], no vascular access insertion in the DR [25, 26] or a low compliance to the protocol when available [9, 11]. Whether premedication use decreases the number of attempts in the DR is important in view of the recently reported association between number of intubation attempts and IVH [6]. In the NICU, some studies found that premedication use decreased the number of attempts before successful intubation [46, 47], sometimes independently of the operator’s experience [48]. Future studies on premedication before DR intubation should assess these aspects.

The strength of this study is its large, exhaustive, population-based design reflecting daily practice in French level-3 centers. Although prospective trials are essential to guide practice, such observational studies also provide valuable information on bedside practices and can inform on the implementation’s chances of randomized trials’ results in daily care. Our results suggest that the use of analgosedation before DR intubation is tied by the local policies and willpower of each center rather than their intrinsic factors. Clinical data on the safety and efficacy of available drugs for premedication in the DR are currently too scarce to edit recommendations. However, alleviation of pain and stress during the neonatal period should remain an essential clinical goal, even in the first minutes of life. This study reinforces the existing clinical equipoise regarding premedication use before neonatal intubation in the DR and shows that neonatal mortality and major morbidities are not increased by premedication, which justifies future clinical trials.

## Supporting information

S1 File Maternity Questionnaire—Original French version. Data that had to be fulfilled with the maternal files and obstetrical team.
(DOCX)

S2 File Annotated maternal interview. Item completed during a face to face interview with the mother to complete socio-economic data and pregnancy-birth informations.
(DOCX)

S3 File Neonatal Questionnaire—English version. Data that had to be fulfilled with the neonates’ files and the neonatal team.
(DOC)

S4 File Neonatal Questionnaire—Original French version. Data that had to be fulfilled with the neonates’ files and the neonatal team.
(DOCX)

S1 Dataset Dataset of all the variables used for this study. (SAV)

## References

[1] Kelly MA, Finer NN. Nasotracheal intubation in the neonate: physiologic responses and effects of atropine and pancuronium. J Pediatr. 1984;105(2):303–9. . 6747766 10.1016/s0022-3476(84)80137-7

[2] Friesen RH, Honda AT, Thieme RE. Changes in anterior fontanel pressure in preterm neonates during tracheal intubation. Anesth Analg. 1987;66(9):874–8. . 3619094

[3] Kumar P, Denson SE, Mancuso TJ, Committee on Fetus and Newborn Section on Anesthesiology and Pain Medicine. Premedication for nonemergency endotracheal intubation in the neonate. Pediatrics. 2010;125(3):608–15. Epub 2010/02/24. 10.1542/peds.2009-2863 . 20176672

[4] Hatch LD, Grubb PH, Lea AS, Walsh WF, Markham MH, Whitney GM, et al Endotracheal Intubation in Neonates: A Prospective Study of Adverse Safety Events in 162 Infants. J Pediatr. 2016;168:62–6 e6. 10.1016/j.jpeds.2015.09.077 . 26541424 PMC4698044

[5] Foglia EE, Ades A, Sawyer T, Glass KM, Singh N, Jung P, et al Neonatal Intubation Practice and Outcomes: An International Registry Study. Pediatrics. 2019;143(1). 10.1542/peds.2018-0902 conflicts of interest to disclose. 30538147 PMC6317557

[6] Sauer CW, Kong JY, Vaucher YE, Finer N, Proudfoot JA, Boutin MA, et al Intubation Attempts Increase the Risk for Severe Intraventricular Hemorrhage in Preterm Infants-A Retrospective Cohort Study. J Pediatr. 2016;177:108–13. 10.1016/j.jpeds.2016.06.051 . 27470688

[7] Porter FL, Wolf CM, Gold J, Lotsoff D, Miller JP. Pain and pain management in newborn infants: a survey of physicians and nurses. Pediatrics. 1997;100(4):626–32. Epub 1997/10/02. . 9310516 10.1542/peds.100.4.626

[8] Muniraman HK, Yaari J, Hand I. Premedication Use Before Nonemergent Intubation in the Newborn Infant. Am J Perinatol. 2015;32(9):821–4. 10.1055/s-0034-1543987 . 25607227

[9] Durrmeyer X, Daoud P, Decobert F, Boileau P, Renolleau S, Zana-Taieb E, et al Premedication for neonatal endotracheal intubation: results from the epidemiology of procedural pain in neonates study. Pediatr Crit Care Med. 2013;14(4):e169–75. Epub 2013/02/27. 10.1097/PCC.0b013e3182720616 . 23439457

[10] Leone TA, Rich W, Finer NN. Neonatal intubation: success of pediatric trainees. J Pediatr. 2005;146(5):638–41. 10.1016/j.jpeds.2005.01.029 . 15870667

[11] Walter-Nicolet E, Zanichelli C, Coquery S, Cimerman P. [Implementation of a specific premedication protocol for tracheal intubation in the delivery room. Practice in two level-III hospitals]. Arch Pediatr. 2014;21(9):961–7. 10.1016/j.arcped.2014.02.006 . 24726672

[12] Barois J, Tourneux P. Ketamine and atropine decrease pain for preterm newborn tracheal intubation in the delivery room: an observational pilot study. Acta Paediatr. 2013;102(12):e534–8. Epub 2013/09/11. 10.1111/apa.12413 . 24015945

[13] Baleine J, Milesi C, Mesnage R, Rideau Batista Novais A, Combes C, Durand S, et al Intubation in the delivery room: experience with nasal midazolam. Early Hum Dev. 2014;90(1):39–43. Epub 2013/12/18. 10.1016/j.earlhumdev.2013.10.007 . 24331827

[14] Milesi C, Baleine J, Mura T, Benito-Castro F, Ferragu F, Thiriez G, et al Nasal midazolam vs ketamine for neonatal intubation in the delivery room: a randomised trial. Arch Dis Child Fetal Neonatal Ed. 2017;103(3):F221–F6. Epub 2017/08/19. 10.1136/archdischild-2017-312808 . 28818854

[15] Ancel PY, Goffinet F, Kuhn P, Langer B, Matis J, Hernandorena X, et al Survival and morbidity of preterm children born at 22 through 34 weeks’ gestation in France in 2011: results of the EPIPAGE-2 cohort study. JAMA Pediatr. 2015;169(3):230–8. . 25621457 10.1001/jamapediatrics.2014.3351

[16] Ancel PY, Goffinet F. EPIPAGE 2: a preterm birth cohort in France in 2011. BMC Pediatrics. 2014;14:97 Epub 2014/04/11. 10.1186/1471-2431-14-97 . 24716860 PMC3991913

[17] Olsen IE, Groveman SA, Lawson ML, Clark RH, Zemel BS. New intrauterine growth curves based on United States data. Pediatrics. 2010;125(2):e214–24. 10.1542/peds.2009-0913 . 20100760

[18] Volpe JJ. Brain injury in premature infants: a complex amalgam of destructive and developmental disturbances. Lancet Neurol. 2009;8(1):110–24. 10.1016/S1474-4422(08)70294-1 . 19081519 PMC2707149

[19] Bell MJ, Ternberg JL, Feigin RD, Keating JP, Marshall R, Barton L, et al Neonatal necrotizing enterocolitis. Therapeutic decisions based upon clinical staging. Ann Surg. 1978;187(1):1–7. . 413500 10.1097/00000658-197801000-00001 PMC1396409

[20] International Committee for the Classification of Retinopathy of P. The International Classification of Retinopathy of Prematurity revisited. Arch Ophthalmol. 2005;123(7):991–9. 10.1001/archopht.123.7.991 . 16009843

[21] Jobe AH, Bancalari E. Bronchopulmonary dysplasia. Am J Respir Crit Care Med. 2001;163(7):1723–9. 10.1164/ajrccm.163.7.2011060 . 11401896

[22] Guedj R, Danan C, Daoud P, Zupan V, Renolleau S, Zana E, et al Does neonatal pain management in intensive care units differ between night and day? An observational study. BMJ open. 2014;4(2):e004086 10.1136/bmjopen-2013-004086 24556241 PMC3931991

[23] Liang KY Z S. Longitudinal Data Analysis Using Generalized Linear Models. Biometrika. 1986;73:13–22.

[24] Simon L, Trifa M, Mokhtari M, Hamza J, Treluyer JM. Premedication for tracheal intubation: a prospective survey in 75 neonatal and pediatric intensive care units. Crit Care Med. 2004;32(2):565–8. Epub 2004/02/06. 10.1097/01.CCM.0000108883.58081.E3 . 14758180

[25] Walter-Nicolet E, Flamant C, Negrea M, Parat S, Hubert P, Mitanchez D. [Premedication before tracheal intubation in French neonatal intensive care units and delivery rooms]. Arch Pediatr. 2007;14(2):144–9. Epub 2006/12/19. . 17175145 10.1016/j.arcped.2006.10.023

[26] Bissuel M, Deguines C, Tourneux P. [A national survey on pain management before tracheal intubation in neonates in French type III maternity units]. Arch Pediatr. 2013;20(2):123–9. Epub 2012/12/19. 10.1016/j.arcped.2012.11.004 . 23245868

[27] Carbajal R, Eble B, Anand KJ. Premedication for tracheal intubation in neonates: confusion or controversy? Semin Perinatol. 2007;31(5):309–17. 10.1053/j.semperi.2007.07.006 . 17905186

[28] Attardi DM, Paul DA, Tuttle DJ, Greenspan JS. Premedication for intubation in neonates. Arch Dis Child Fetal Neonatal Ed. 2000;83(2):F161 Epub 2000/09/30. 10.1136/fn.83.2.F160c . 11012274 PMC1721146

[29] van Alfen-van der Velden AA, Hopman JC, Klaessens JH, Feuth T, Sengers RC, Liem KD. Effects of midazolam and morphine on cerebral oxygenation and hemodynamics in ventilated premature infants. Biol Neonate. 2006;90(3):197–202. Epub 2006/05/24. 10.1159/000093489 . 16717443

[30] van Straaten HL, Rademaker CM, de Vries LS. Comparison of the effect of midazolam or vecuronium on blood pressure and cerebral blood flow velocity in the premature newborn. Dev Pharmacol Ther. 1992;19(4):191–5. . 1364197 10.1159/000457484

[31] Ng E T A, Ohlsson A. Intravenous midazolam infusion for sedation of infants in the neonatal intensive care unit. Cochrane Database Syst Rev 2017;1 31;1(CD002052). 10.1002/14651858.CD002052.pub3 PMC6464963 28141899

[32] Bhutta AT. Ketamine: a controversial drug for neonates. Semin Perinatol. 2007;31(5):303–8. 10.1053/j.semperi.2007.07.005 . 17905185

[33] Dong C, Anand KJ. Developmental neurotoxicity of ketamine in pediatric clinical use. Toxicol Lett. 2013;220(1):53–60. 10.1016/j.toxlet.2013.03.030 . 23566897

[34] Milesi C, Cambonie G, Jacquot A, Barbotte E, Mesnage R, Masson F, et al Validation of a neonatal pain scale adapted to the new practices in caring for preterm newborns. Arch Dis Child Fetal Neonatal Ed. 2010;95(4):F263–6. 10.1136/adc.2008.144758 . 19221401

[35] Bhutada A, Sahni R, Rastogi S, Wung JT. Randomised controlled trial of thiopental for intubation in neonates. Arch Dis Child Fetal Neonatal Ed. 2000;82(1):F34–7. 10.1136/fn.82.1.F34 . 10634839 PMC1721021

[36] Millar C, Bissonnette B. Awake intubation increases intracranial pressure without affecting cerebral blood flow velocity in infants. Can J Anaesth. 1994;41(4):281–7. 10.1007/BF03009904 . 8004731

[37] Norman E, Wikstrom S, Hellstrom-Westas L, Turpeinen U, Hamalainen E, Fellman V. Rapid Sequence Induction is Superior to Morphine for Intubation of Preterm Infants: A Randomized Controlled Trial. J Pediatr. 2011;159(6):893–9.e1. Epub 2011/07/30. 10.1016/j.jpeds.2011.06.003 . 21798556

[38] Barrington K. Premedication for endotracheal intubation in the newborn infant. Paediatr Child Health. 2011;16(3):159–71. . 22379381 10.1093/pch/16.3.159 PMC3077307

[39] Durrmeyer X, Dahan S, Delorme P, Blary S, Dassieu G, Caeymaex L, et al Assessment of atropine-sufentanil-atracurium anaesthesia for endotracheal intubation: an observational study in very premature infants. BMC Pediatrics. 2014;14(1):120 10.1186/1471-2431-14-120 24886350 PMC4028002

[40] Papoff P, Mancuso M, Caresta E, Moretti C. Effectiveness and safety of propofol in newborn infants. Pediatrics. 2008;121(2):448; author reply -9. Epub 2008/02/05. 10.1542/peds.2007-3132 . 18245439

[41] Welzing L, Kribs A, Eifinger F, Huenseler C, Oberthuer A, Roth B. Propofol as an induction agent for endotracheal intubation can cause significant arterial hypotension in preterm neonates. Paediatr Anaesth. 2010;20(7):605–11. Epub 2010/07/21. 10.1111/j.1460-9592.2010.03330.x . 20642659

[42] Mancuso A, De Vivo A, Giacobbe A, Priola V, Maggio Savasta L, Guzzo M, et al General versus spinal anaesthesia for elective caesarean sections: effects on neonatal short-term outcome. A prospective randomised study. J Matern Fetal Neonatal Med. 2010;23(10):1114–8. 10.3109/14767050903572158 . 20088721

[43] Finer NN, Carlo WA, Walsh MC, Rich W, Gantz MG, Laptook AR, et al Early CPAP versus surfactant in extremely preterm infants. N Engl J Med. 2010;362(21):1970–9. Epub 2010/05/18. 10.1056/NEJMoa0911783 . 20472939 PMC3071534

[44] Aly H, Massaro AN, Patel K, El-Mohandes AA. Is it safer to intubate premature infants in the delivery room? Pediatrics. 2005;115(6):1660–5. 10.1542/peds.2004-2493 . 15930230

[45] Sweet DG, Carnielli V, Greisen G, Hallman M, Ozek E, Plavka R, et al European consensus guidelines on the management of neonatal respiratory distress syndrome in preterm infants—2010 update. Neonatology. 2010;97(4):402–17. 10.1159/000297773 . 20551710

[46] Roberts KD, Leone TA, Edwards WH, Rich WD, Finer NN. Premedication for nonemergent neonatal intubations: a randomized, controlled trial comparing atropine and fentanyl to atropine, fentanyl, and mivacurium. Pediatrics. 2006;118(4):1583–91. 10.1542/peds.2006-0590 . 17015550

[47] Oei J, Hari R, Butha T, Lui K. Facilitation of neonatal nasotracheal intubation with premedication: a randomized controlled trial. J Paediatr Child Health. 2002;38(2):146–50. Epub 2002/05/29. . 12030995 10.1046/j.1440-1754.2002.00726.x

[48] Le CN, Garey DM, Leone TA, Goodmar JK, Rich W, Finer NN. Impact of premedication on neonatal intubations by pediatric and neonatal trainees. J Perinatol. 2014;34(6):458–60. 10.1038/jp.2014.32 . 24577435

