# Supplementary material for: Premedication practices for delivery room intubations in premature infants in France: Results from the EPIPAGE 2 cohort study
Source: PLoS One. 2019 Apr 10;14(4):e0215150. doi: 10.1371/journal.pone.0215150 (PMC6457540; doi:10.1371/journal.pone.0215150)
Supplement: S1 File — Data that had to be fulfilled with the maternal files and obstetrical team. (DOCX) [file pone.0215150.s001.docx]

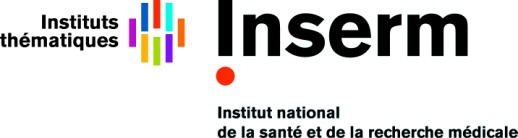


| VARIABLES SURLIGNEES | **Variable indirectement identifiante – Transmise sous condition** |
| --- | --- |
| VARIABLES EN ROUGE | **Nom Variable** |
| ***Variables en vert*** | **Variables calculées** |
| ***Variables en marron*** | **Variables de synthèse/corrigé** |
| *MODALITÉS DE RÉPONSES (D_NOM_DICO)* | **Nom Dictionnaire** |
| Encadrements en noir | **Blocs conditionnels** |

Les **variables DATE indirectement identifiantes** seront données en **âge gestationnel (SA+J)** calculé à partir de la date de naissance de l’enfant

**
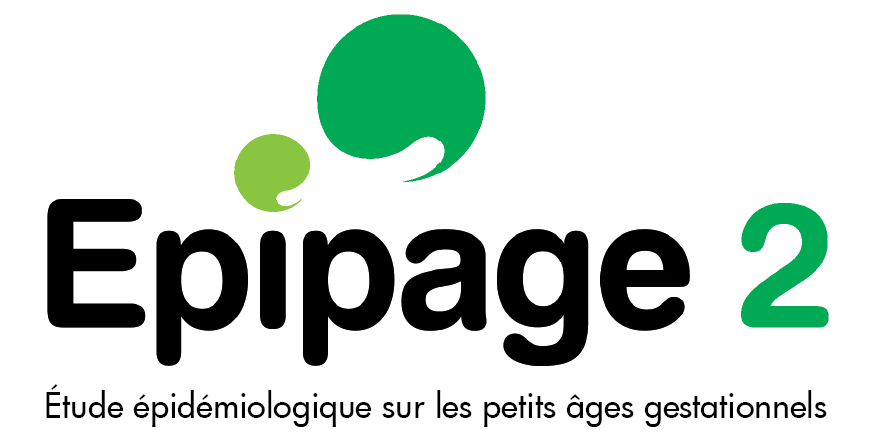
**

**Questionnaire MATERNITÉ**

**ANNOTE**

**Données à recueillir à partir**

**du dossier maternel obstétrical**

**et de l’équipe obstétricale**

***Les définitions, codages et précisions nécessaires seront fournis dans le guide de l’enquêteur et / ou dans l’outil de collecte informatisée.***

**Table des matières**

**PARTIE MERE**

**CARACTERISTIQUES GENERALES DE LA MERE page 3**

**ANTECEDENTS MATERNELS page 4**

**GROSSESSE ACTUELLE page 7**

**Généralités  page 7**

**Grossesse multiple page 9**

**Dépistage prénatal page 10**

**Discussions de limitation / d'interruption des soins, d'IMG page 11**

**Pathologie(s) et Hospitalisation(s) en cours de grossesse page 13**

**Traitements médicamenteux en cours de grossesse page 14**

**Hospitalisation se terminant par l’accouchement page 17**

**Parcours de la mère page 17**

**Pathologies page 18**

**Traitements page 23**

**Derniers examens avant le travail / la césarienne page 25**

**ACCOUCHEMENT page 31**

**Contexte, décisions page 31**

**Périnée, Histologie placentaire page 33**

**SEJOUR DE LA MERE EN MATERNITE page 34**

**PARTIE ENFANT**

**PARTIE A COMPLETER DANS UN CAS D’ IMG page 36**

**NAISSANCE page 36**

**PARTIE A COMPLETER DANS UN CAS D’ENFANT(S) MORT-NE(S) ou IMG page 38**

**PRISE EN CHARGE EN SALLE DE NAISSANCE page 40**

**CARACTERISTIQUES GENERALES DE LA MÈRE** Préfixé par : MERE_MA

**Type d’accouchement l__l ENR_TYPE_NAISSANCE**

*1 : Singleton - 2 : Jumeau - 3 : Triplet - 4 : Quadruplet D_TYPNAIS*

**Age de la mère à l'accouchement en année révolue**  l__l __l **COR_AGE_MERE**

Commune de résidence (en clair) :

………………… ………………………………………………………………………………… MERE_MA4 Code postal l__l__l__l__l__l MERE_MA5

Pays de naissance (en clair) : ……………………………………………………..……………… MERE_MA6

n° insee l__l__l__l__l__l MERE_MA7

coder l__l MERE_MA8

Données dossier maternité complété par les données de l’entretien maternel et 1 an COR_PAYS_NAISS

*1 : France D_PAYS2F*

*2 : autre pays d’Europe*

*3 : pays d’Afrique du Nord*

*4 : autre pays d’Afrique,*

*5 : autre*

Nationalité (en clair) : ……………………………………………………..…………………… MERE_MA9

+ coder : l__l MERE_MA11

*1 : France D_PAYS2F*

*2 : autre pays d’Europe*

*3 : pays d’Afrique du Nord*

*4 : autre pays d’Afrique*

*5 : autre*

Couverture sociale : l__l MERE_MA12

*1 : Sécurité sociale*

*2 : CMU*

*3 : AME ou soins urgents D_SOCIALE*

*4 : aucune*

La mère vit-elle en couple ? non=0, oui=1 l__l MERE_MA13

Situation vis-à-vis de l’emploi : l__l MERE_MA14

*1 : activité professionnelle*

*2 : chômage, recherche d’emploi D_ACTIVITE*

*3 : au foyer*

*4 : étudiante*

*5 : autre*

Quelle est la profession de la mère, ou la dernière profession exercée ? (en clair) :

……………....……………………………………………………………………… MERE_MA15

+ coder : l__l__l MERE_MA16

*1 : agricultrice D_PROFESSION*

*2 : artisan, commerçante*

*3 : cadre (profession libérale, professeur, ingénieur ...)*

*4 : profession intermédiaire (institutrice, infirmière, technicienne, contremaître...)*

*5 : employée de la fonction publique ou administrative des entreprises*

*6 : employée de commerce*

*7 : personnel de service pour les particuliers*

*8 : ouvrière qualifiée*

*9 : ouvrière non qualifiée*

*0 : sans profession*

Situation vis-à-vis de l’emploi du conjoint/partenaire : l__l MERE_MA17

*1 : activité professionnelle*

*2 : chômage, recherche d’emploi D_ACTIVITE*

*3 : au foyer*

*4 : étudiant*

*5 : autre*

Quelle est la profession du conjoint/partenaire, ou la dernière profession exercée ? (en clair) :

…………………………………………………………………………………………………… MERE_MA18

+ coder : l__l__l MERE_MA19

*1 : agriculteur* *D_PROFESSION*

*2 : artisan, commerçant*

*3 : cadre (profession libérale, professeur, ingénieur...)*

*4 : profession intermédiaire (instituteur, infirmier, technicien, contremaître...)*

*5 : employé de la fonction publique ou administrative des entreprises*

*6 : employé de commerce*

*7 : personnel de service pour les particuliers*

*8 : ouvrier qualifié*

*9 : ouvrier non qualifié*

*0 : sans profession*

**CSP du ménage (ou de la maman si vit seule) COR_CSPMENAGE**

| 1 : Cadre |
| --- |
| 2 : Intermédiaire |
| 3 : Administratif, chef E, fonction publique, étudiant |
| 4 : Empl service,commerce |
| 5 : Ouvrier |
| 6 : Sans profession |

*Données dossier médical complété par l’entretien*

**ANTECEDENTS MATERNELS / PATERNEL** Préfixé par : MERE_MB

##

**Taille de la mère** l__l__l__l cm MERE_MB1

**Poids de la mère avant cette grossesse** l__l__l__l kg MERE_MB2

**Taille du père de l’enfant** l__l__l__l cm MERE_MB3

**Poids du père de l’enfant** l__l__l__l kg MERE_MB4

## Antécédents médicaux MATERNELS

**HTA permanente en dehors d’une grossesse** : non=0, oui=1 l**_**_l MERE_MB5

**Diabète en dehors d’une grossesse** : non=0, oui=1 l**_**_l MERE_MB6

Si oui, *1 : diabète type 1 (insuliné) ;* l**_**_l MERE_MB6A

*2 : diabète type 2 (régime seul, antidiab. oraux)* *D_DIABETE*

**Antécédent de troubles psychiatriques**: non=0, oui=1 l__l MERE_MB7

Si oui, antécédent de dépression traitée : non=0, oui=1 l__l MERE_MB8

antécédent de tentative de suicide : non=0, oui=1 l__l MERE_MB9

**Alcoolisme connu** : non=0, oui=1 l__l MERE_MB10

**Toxicomanie connue** : non=0, oui=1 l__l MERE_MB11

Si oui : cannabis : non=0, oui=1 l**__**l MERE_MB12

cocaïne : non=0, oui=1 l__l MERE_MB13

héroïne : non=0, oui=1 l__l MERE_MB14

autre : non=0, oui=1 l__l MERE_MB15

Si autre, précisez en clair ……………………………………………..…MERE_MB15A

**Autre(s) pathologie(s) chronique(s) :** non=0, oui=1 l__l MERE_MB16

Si oui, détails en clair MERE_MB16A

## Antécédents obstétricaux

## **Nombre de grossesses antérieures :** l__l__l MERE_MB17

## Si la grossesse actuelle est la 1^ère^ grossesse, passer directement à la partie «grossesse actuelle»

**Antécédent de pré-éclampsie ou HTA gravidique** : non=0, oui=1 l__l MERE_MB18

**Antécédent de RCIU ou hypotrophie** : non=0, oui=1 l__l MERE_MB19

## Remplir le tableau ci-dessous : MERE_MB21_1 à MERE_MB37_10

| **Grossesse** | **Multiple**  Non=0  Oui=1 | **AG de l’issue**  *D_AGISSUE*  1 : > 37 SA  2 : 29-36 SA  3 : 22-28 SA  4 : 14-21 SA | **Si < 22 SA** | | **Si > 22 SA** | | **IMG :**  Non=0  Oui=1 | **Malformation :**  Non=0  Oui=1  Si oui, type de malf. en clair |
| --- | --- | --- | --- | --- | --- | --- | --- | --- |
|  |  |  | 1 : IVG *  2 : FCS**  *D_IVG* | **Chirurgie / curetage**  Non=0  Oui=1 | 1 : Enfant né vivant  2 : Mort-né  *D_STATUTENF* | 1: Voie basse  2:Cesar.  *D_ACCVOIE* |  |  |
| n°1 | \|**21_1**\| | \|**22_1**\| | \|**23_1**\| | \|**24_1**\| | \|**25_1**\| | \|**26_1**\| | \|**27_1**\| | \|**28_1**\|……**29_1**…………… |
| *si gémellaire* |  | \|**30_1**\| | \|**31_1**\| | \|**32_1**\| | \|**33_1**\| | \|**34_1**\| | \|**35_1**\| | \|**36_1**\|……**37_1**……...... |
| n°2 | \|**21_2**\| | \|**22_2**\| | \|**23_2**\| | \|**24_2**\| | \|**25_2**\| | \|**26_2**\| | \|**27_2**\| | \|**28_2**\|……**29_2**……....... |
| *si gémellaire* |  | \|**30_2**\| | \|**31_2**\| | \|**32_2**\| | \|**33_2**\| | \|**34_2**\| | \|**35_2**\| | \|**36_2**\|……**37_2**…....... |
| n°3 | \|**21_3**\| | \|**22_3**_\| | \|**23_3**\| | \|**24_3**\| | \|**25_3**\| | \|**26_3**\| | \|**27_3**\| | \|**28_3**\|……**29_3**………....... |
| *si gémellaire* |  | \|**30_3**\| | \|**31_3**\| | \|**32_3**\| | \|**33_3**\| | \|**34_3**\| | \|**35_3**\| | \|**36_3**\|……**37_3**……...... |
| n°4 | \|**21_4**\| | \|**22_4**\| | \|**23_4**\| | \|**24_4**\| | \|**25_4**\| | \|**26_4**\| | \|**27_4**\| | \|**28_4**\|……**29_4**…………… |
| *si gémellaire* |  | \|**30_4**\| | \|**31_4**\| | \|**32_4**\| | \|**33_4**\| | \|**34_4**\| | \|**35_4**\| | \|**36_4**\|……**37_4**……...... |
| n°5 | \|**21_5**\| | \|**22_5**\| | \|**23_5**\| | \|**24_5**\| | \|**25_5**\| | \|**26_5**\| | \|**27_5**\| | \|**28_5**\|……**29_5**…………… |
| *si gémellaire* |  | \|**30_5**\| | \|**31_5**\| | \|**32_5**\| | \|**33_5**\| | \|**34_5**\| | \|**35_5**\| | \|**36_5**\|……**37_5**……...... |
| n°6 | \|**21_6**\| | \|**22_6**\| | \|**23_6**\| | \|**24_6**\| | \|**25_6**\| | \|**26_6**\| | \|**27_6**\| | \|**28_6**\|……**29_6**…………… |
| *si gémellaire* |  | \|**30_6**\| | \|**31_6**\| | \|**32_6**\| | \|**33_6**\| | \|**34_6**\| | \|**35_6**\| | \|**36_6**\|……**37_6**……...... |
| n°7 | \|**21_7**\| | \|**22_7**\| | \|**23_7**\| | \|**24_7**\| | \|**25_7**\| | \|**26_7**\| | \|**27_7**\| | \|**28_7**\|……**29_7**…………… |
| *si gémellaire* |  | \|**30_7**\| | \|**31_7**\| | \|**32_7**\| | \|**33_7**\| | \|**34_7**\| | \|**35_7**\| | \|**36_7**\|……**37_7**……...... |
| n°8 | \|**21_8**\| | \|**22_8**\| | \|**23_8**\| | \|**24_8**\| | \|**25_8**\| | \|**26_8**\| | \|**27_8**\| | \|**28_8**\|……**29_8**…………… |
| *si gémellaire* |  | \|**30_8**\| | \|**31_8**\| | \|**32_8**\| | \|**33_8**\| | \|**34_8**\| | \|**35_8**\| | \|**36_8**\|……**37_8**……...... |
| n°9 | \|**21_9**\| | \|**22_9**\| | \|**23_9**\| | \|**24_9**\| | \|**25_9**\| | \|**26_9**\| | \|**27_9**\| | \|**28_9**\|……**29_9**…………… |
| *si gémellaire* |  | \|**30_9**\| | \|**31_9**\| | \|**32_9**\| | \|**33_9**\| | \|**34_9**\| | \|**35_9**\| | \|**36_9**\|……**37_9**……...... |
| n°10 | \|**21_10**\| | \|**22_10**\| | \|**23_10**\| | \|**24_10**\| | \|**25_10**\| | \|**26_10**\| | \|**27_10** | \|**28_10**\|……**29_10**…… |
| *si gémellaire* |  | \|**30_10**\| | \|**31_10**\| | \|**32_10**\| | \|**33_10**\| | \|**34_10**\| | \|**35_10** | \|**36_10**\|……**37_10**……...... |

***** **IVG** : Interruption volontaire de grossesse

**** FCS** : fausse-couche spontanée

**Antécédent de grossesse triple ou quadruple**: non=0, oui=1 l__l MERE_MB38

Si oui, remplir le tableau ci-dessous : MERE_MB39_1 à MERE_MB47_4_2

| **Grossesse**  *Mettre le n° de grossesse correspondant au tableau*  *ci-dessus (si la grossesse n°3 était triple, coder « 3 » dans la case et remplir la ligne « triple »)* | | **AG de l’issue**  1 : > 37 SA  2 : 29-36 SA  3 : 22-28 SA  4 : 14-21 SA  5 : <14 SA | **Si < 22 SA** | | **Si > 22 SA** | | **IMG :**  Non=0  Oui=1 | **Malformation :**  Non=0  Oui=1  Si oui, type de malf. en clair |
| --- | --- | --- | --- | --- | --- | --- | --- | --- |
|  |  |  | 1 : IVG *  2 : FCS** | **Chirurgie / curetage**  Non=0  Oui=1 | 1 : Enfant né vivant  2 : Mort-né | 1: Voie basse  2:Cesar. |  |  |
| n°\| _\| **mb39_1** | *grossesse triple* | \|__\| **mb40_3_1** | \|__\| **mb41_3_1** | \|__\| **mb42_3_1** | \|__\| **mb43_3_1** | \|__\| **mb44_3_1** | \|__\| **mb45_3_1** | \|__\| **mb46_3_1 mb47_3_1**….... |
|  | *grossesse quadruple* | \|__\| **mb40_4_1** | \|__\| **mb41_4_1** | \|__\| **mb42_4_1** | \|__\| **mb43_4_1** | \|__\| **mb44_4_1** | \|__\| **mb45_4_1** | \|__\| **mb46_4_1 mb47_4_1**….... |
| n°\|__\| **mb39_2** | *grossesse triple* | \|__\| **mb40_3_2** | \|__\| **mb41_3_2** | \|__\| **mb42_3_2** | \|__\| **mb43_3_2** | \|__\| **mb44_3_2** | \|__\| **mb45_3_2** | \|__\|  **mb46_3_2 mb47_3_2**…... |
|  | *grossesse quadruple* | \|__\| **mb40_4_2** | \|__\| **mb41_4_2** | \|__\| **mb42_4_2** | \|__\| **mb43_4_2** | \|__\| **mb44_4_2** | \|__\| **mb45_4_2** | \|__\| **mb46_4_2 mb47_4_2**….... |

**Parité : Accouchement antérieur à 22SA ou plus**  l__lMERE_PARITECL

| *0 = primipare D_PARITECL* |
| --- |
| *1 = 1* |
| *2 = 2 et +* |

# GROSSESSE ACTUELLE Prefixé par MERE_MC

**Première consultation pour cette grossesse avant 15 SA, quel que soit le lieu :**

non=0,oui=1 l__l MERE_MC1

**Suivi régulier de la grossesse (consultation mensuelle ou hospitalisation) :** non=0, oui=1 l__l MERE_MC2

**Tabac pendant la grossesse (même durée courte) :** non=0, oui=1 l__l MERE_MC3

Si oui, nombre moyen de cigarettes par jour l__l__l MERE_MC4

Arrêt pendant la grossesse ? l__l MERE_MC5

*0 : Non*  *D_ARRET_TABAC*

*1 : Oui au cours du 1^er^ trimestre*

*2 : Oui au cours du 2^ème^ trimestre*

*3 : Oui au cours du 3^ème^ trimestre*

*4 : Oui, sans renseignement sur la date*

**Traitement de l’infertilité** : non=0, oui=1 l__l MERE_MC6

Si oui : Type de traitement l__l MERE_MC7

*1 : FIV avec ICSI D_INFERTILITE*

*2 : FIV sans ICSI*

*3 : Insémination artificielle*

*4 : Stimulation hormonale seule*

Don de sperme : non=0, oui=1 l__l MERE_MC8

Don d’ovocyte : non=0, oui=1 l__l MERE_MC9

Don d’embryon : non=0, oui=1 l__l MERE_MC10

Traitement de l’infertilité effectué en France : non=0, oui=1 l__l MERE_MC11

si « non », préciser le pays …………*D_PAYS*…………………………….MERE_MC12A

( + code INSEE : l__l__l__l__l__l ) MERE_MC12

**Date de début de grossesse estimée dans le dossier** (JJ/MM/AAAA)**:** l__l__l/ l__l__l /l__l__l__l__l MERE_MC13

**Date des dernières règles** (JJ/MM/AAAA) :__ l__l__l/ l__l__l /l__l__l__l__**l** MERE_MC16

**Echographie 1^er^ Trimestre** : non=0, oui=1 l__l MERE_MC19

Si oui,

Nombre de fœtus au 1^er^ trimestre : l__l MERE_MC20

Date de l’écho *(si pas de date, donner l’âge gestationnel)* : l__l__l/ l__l__l /l__l__l__l__l MERE_MC21

ou l__l__l SA + l__l jours MERE_MC24 MERE_MC25

**Date écho calculée (SA)**  l__l__l **MERE_MC21A (J)** l__l **MERE_MC21B**

Concernant le fœtus unique, ou un des fœtus si grossesse multiple :

LCC mesurée : l__l__l mm MERE_MC26

Clarté nucale : l__l,l__l mm MERE_MC27

Concernant un 2^ème^ fœtus si grossesse multiple :

LCC mesurée : l__l__l mm MERE_ MC28

Clarté nucale : l__l,l__l mm MERE_MC29

Concernant un 3^ème^ fœtus si grossesse multiple :

LCC mesurée : l__l__l mm MERE_MC30

Clarté nucale : l__l,l__l mm MERE_MC31

## Grossesse multiple

**Grossesse multiple (en début de grossesse):** non=0, oui=1 l__l MERE_MC32

Si non, passer à la partie « dépistage prénatal »

Si oui,

**Nombre de fœtus :** l__l MERE_ MC33

Si deux : Chorionicité l__l MERE_MC34

*1 : bichorial / biamniotique*

*2 : monochorial / monoamniotique D_CHORIONICITE*

*3 : monochorial / biamniotique*

Si  trois ou plus : Nombre de placentas l__l MERE_MC35

Nombre de sacs amniotiques l__l MERE_MC36

**Réduction embryonnaire (si < 15 SA) ?** non=0, oui=1 l__l MERE_MC37

Si oui, de l__l à l__l embryon(s) MERE_MC38 / MC39

**Interruption sélective de grossesse (si > 15 SA) ?** non=0, oui=1 l__l MERE_MC40

Si oui, de l__l à l__l fœtus MERE_MC41 / MC42

**Fausse-couche spontanée < 22SA d’un des fœtus ?** non=0, oui=1 l__l MERE_MC43

Si oui, nombre de fausses-couches spontanées < 22 SA l__l MERE_MC44

A quel terme ? (la plus tardive si plusieurs) l__l__lSA MERE_MC45

**Syndrome transfuseur/transfusé**: non=0, oui=1 l__l MERE_MC46

Si oui,

**Traitement par laser**: non=0, oui=1, non documenté=9 l__l MERE_MC47

Si oui, AG au début de traitement (1^ère^ intervention) l__l__l SA MERE_MC48

**Amniodrainage**: non=0, oui=1, non documenté=9 l__l MERE_MC49

Si oui, AG du 1^er^ amniodrainage l__l__l SA MERE_MC50

## **Autres pathologies spécifiques des grossesses multiples, préciser en clair** **:** MERE_MC51

##

##

## **Cas difficiles, préciser en clair** : MERE_MC53

##

##

## Dépistage prénatal Prefixé par MERE_MD

**Dépistage de la Trisomie 21 ?** non=0, oui=1 l__l MERE_MD1

Si oui, Méthode l__l MERE_MD2

*1 = Combiné T1 D_TRISOMIE*

*2 = Intégré T2*

*3 = Marqueur sériques uniquement T2*

Résultat pathologique : non=0, oui=1 l__l MERE_ MD3

**Autres actes de diagnostic prénatal (pour au moins 1 fœtus si grossesse multiple) :**

non=0, oui=1 l__l MERE_MD4

Si oui,

**Echographie en centre de référence** : non=0, oui=1 l__l MERE_MD5

**IRM** : non=0, oui=1 l__l MERE_MD6

**Biopsie du trophoblaste** : non=0, oui=1 l__l MERE_ MD7

Si oui : date l__l__l/ l__l__l /l__l__l__l__l MERE_MD8

**Date biopsie trop. calculée (SA) l__l__l MERE_MD8A (J) l__l MERE_MD8B**

**Amniocentèse** : non=0, oui=1 l__l MERE_MD11

Si oui : date l__l__l/ l__l__l /l__l__l__l__l MERE_ MD12

**Date amnio. calculée (SA) l__l__l MERE_MD12A (J) l__l MERE_MD12B**

**Autre Prélèvement fœtal** : non=0, oui=1 l__l MERE_MD15

Si oui : date l__l__l/ l__l__l /l__l__l__l__l MERE_ MD16

**Date prélèv. fœtal calculée (SA) l__l__l MERE_MD16A (J) l__l MERE_MD16B**

Préciser : MERE_MD19

**Prélèvement de sang fœtal** : non=0, oui=1 l__l MERE_ MD20

Si oui : date l__l__l/ l__l__l /l__l__l__l__l MERE_ MD21

**Date prélèv. sang fœt. calculée (SA) l__l__l MERE_MD21A (J) l__l MERE_MD21B**

**Caryotype réalisé** : non=0, oui=1 l__l MERE_MD24

Si oui : résultat normal ? non=0, oui=1 l__l MERE_MD25

Si résultat anormal, préciser en clair : MERE_MD26

**Diagnostic prénatal d’une pathologie fœtale ou d’une séroconversion infectieuse** :

non=0, oui=1 l__l MERE_MD27

Si oui, terme du diagnostic  l__l__l SA MERE_MD28

Les principales pathologies : l__l MERE_MD29

*1 : chromosomique  D_PATHOFOETALE*

| *2 : génique  4 : malformation(s)* |
| --- |
| *3 : infectieuse  5 : autre pathologie foetale* |
|  |
|  |
|  |
| autre : préciser ……………………………………………………………….MERE_MD30 |
|  |
|  |
|  |

# Discussion anténatale de limitation ou d’arrêt des thérapeutiques, quels que soient le terme et l’issue (né vivant, mort-né ou IMG) Prefixé par MERE_ME

# Pour cet enfant, ou un des enfants (en cas de grossesse multiple) y-a-t-il eu discussion d’IMG, de limitation ou d’arrêt des thérapeutiques devant se dérouler pendant la grossesse ?

# non=0, oui=1 l__l MERE_ME1

Si «non», passer à la partie « Pathologie(s) et hospitalisation(s) en cours de grossesse »

Si «oui», renseigner les items suivants :

**Si grossesse multiple,** cela concerne (plusieurs réponses possibles)

# J1 / T1 : non=0, oui=1 l__l MERE_ME2

J2 / T2 : non=0, oui=1 l__l MERE_ME3

T3 : non=0, oui=1 l__l MERE_ME4

**Motif(s) ayant mené à la discussion** :

# Menace d’accouchement prématuré : non=0, oui=1 l__l MERE_ME5

# Rupture prématurée des membranes avant terme : non=0, oui=1 l__l MERE_ME6

# RCIU : non=0, oui=1 l__l MERE_ME7

# Causes maternelles : non=0, oui=1 l__l MERE_ME8

# Anomalies constitutionnelles fœtales, confirmées ou suspectées : non=0, oui=1 l__l MERE_ME9

# Autre : non=0, oui=1 l_ l MERE_ME10

Si oui, préciser en clair ……………………………………………………………………………….MERE_ME11

Au cas où plusieurs motifs sont associés, y a-t-il un motif dominant ? non=0, oui=1 l__**l** MERE_ME12

Si oui, préciser en clair ……………………………………………………………………………….MERE_ME13

**Y a-t-il eu une ou plusieurs discussion(s) pluridisciplinaire(s)** **?** non=0, oui=1 l__l MERE_ME14

**Y a-t-il eu un ou plusieurs entretien(s) préalable(s) à la décision avec le ou les parent(s)?**

non=0, oui=1 l__ l MERE_ME 15

Si oui, Avec le pédiatre: non=0, oui=1 l__l MERE_ME16

Avec l’obstétricien: non=0, oui=1 l__l MERE_ME17

Conjointement avec l’obstétricien et le pédiatre : non=0, oui=1 l__l MERE_ME18

Contenu de l’entretien reporté dans le dossier médical : non=0, oui=1 l__**l** MERE_ME19

**Degré d’implication des parents dans la prise de décision :** l__l MERE_ME20

*1 : Les parents n’ont pas été informés de la prise d’une décision D_DECISION*

*2 : Les parents ont été informés, sans qu’un avis ne leur ait été demandé*

*3 : L’avis des parents a été demandé*

*4 : Les parents ont décidé*

### Opinion des parents en anténatal :

Demande d’IMG : non=0, oui=1 l__l MERE_ME21

Poursuite de la grossesse : non=0, oui=1 l__l MERE_ME22

Si oui, l__l MERE_ME23

*1 : Expectative et soins palliatifs à la naissance (si enfant vivant)  D_POURSUITEG*

*2 : Prise en charge active complète*

Indécision : non=0, oui=1 l__l MERE_ME24

Les parents s’en remettent à l’équipe médicale pour la décision : non=0, oui=1 l__l MERE_ME25

Information non disponible : non=0, oui=1 l__l MERE_ME26

Autre : non=0, oui=1 l__l MERE_ME27

Si oui, préciser en clair ……………………………………………………………………. MERE_ME28

**Décisions portant sur la poursuite de la grossesse :**

**Demande d’avis CPDP** : non=0, oui=1 I__l MERE_ME29

Si oui : Conclusion du CPDP :

Date (JJ/MM/AAA) l__l__l/ l__l__l/l__l__l__l__l MERE_ME30

**Date conclusion CPDP calculée (SA) l__l__l MERE_ME30A (J) l__l MERE_ME30B**

Acceptation IMG non=0, oui=1 l__l MERE_ME33

Si oui, pour motif : l__l MERE_ME34

*1 : fœtal, 2 : maternel, 3 : les deux D_IMGMOTIF*

**Demande d’IMG pour raison maternelle** (hors CPDP) : non=0, oui=1 l__l MERE_ME35

Si oui : Date attestation médicale l__l__l/ l__l__l /l__l__l__l__l MERE_ME36

**Date attestation méd. calculée (SA) l__l__l MERE_ME36A (J) l__l MERE_ME36B**

Motif : ……………………………………………………………………………… MERE_ME39

**Décision anténatale d’IMG ?** non=0, oui=1 l__l MERE_ME40

Si oui, *1 : Avec foeticide*, l__l MERE_ME41

*2 : Sans foeticide D_FOETICIDE*

**Décision anténatale de limitation/abstention thérapeutique anténatale** : non=0, oui=1 l__l MERE_ME42

Si oui, objet de la décision :

Renoncement à un transfert in utero : non=0, oui=1 l__l MERE_ME43

Abstention d’une extraction césarienne : non=0, oui=1 l__l MERE_ME44

Pas de surveillance par enregistrement du RCF : non=0, oui=1 l__l MERE_ME45

Pas de décision d’extraction envisagée si anomalies du RCF : non=0, oui=1 l__l MERE_ME46

Pas de corticothérapie anténatale : non=0, oui=1 l__l MERE_ME47

**Décision anténatale de poursuite de la grossesse et de prise en charge et traitement** :

non=0, oui=1 l__| MERE_ME48

**Au total : la situation a-t-elle débouché sur une mort fœtale in utero (MFIU) ?** non=0, oui=1l__lMERE_ME49

# Décisions anténatales portant sur la prise en charge pédiatrique anticipée à la naissance :

# non=0, oui=1 l__l MERE_ ME50

**Options de prise en charge** l__l MERE_ME51

*1 : Offrir une réanimation complète D_CHARGE*

*2 : Ne pas réanimer, quel que soit l’ état à la naissance*

*3 : Réanimer selon l’état de l’enfant à la naissance*

*4 : Faire ou débuter soins palliatifs ou soins de confort en salle de naissance*

## **Pathologie(s) et Hospitalisation(s) en cours de grossesse** Préfixé par MERE_MH

## **Hospitalisation(s) au cours de la grossesse** (en dehors de l’hospitalisation se terminant par l’accouchement traitée dans la suite du questionnaire) : non=0, oui=1 l__l MERE_MH1

Si oui : *1 : rpm - 2 : infection  - 3 : pathologie hypertensive  - 4 : métrorragies  - 5 : autre*  *D_HOSPMOTIF*

| Etablissement  (Nom + Ville) | Terme (environ) à l’hospitalisation. | Motif d’hospitalisation |
| --- | --- | --- |
| nom MERE_MH2  ville MERE_MH3 | L**_**l__l SA MERE_MH4 | MERE_MH5 *D_HOSPMOTIF*  autre : préciser MERE_MH6 |
| nom MERE_MH7  ville MERE_MH8 | L_l__l SA MERE_MH9 | MERE_MH10 *D_HOSPMOTIF*  autre : préciser MERE_MH11 |
| nom MERE_MH12  ville MERE_MH13 | \|__l__l SA MERE_MH14 | MERE_MH15 *D_HOSPMOTIF*  autre : préciser MERE_MH16 |

**Cerclage**: non=0, oui=1 l__l MERE_MH17

Si oui, date l__l__l/ l__l__l /l__l__l__l__l MERE_MH18

**Date cerclage calculée (SA) l__l__l MERE_MH18A (J) l__l MERE_MH18B**

Indication : l__l MERE_MH21

*1=prophylactique  - 2=échographique  - 3=en urg^ce^ pour dilatation*  *D_CERCLAGE*

**Métrorragies 1^er^ trimestre** : non=0, oui=1 l__l MERE_MH22

**Métrorragies  2^ème^ trimestre** : non=0, oui=1 l__l MERE_MH23

**Métrorragies  3^ème^ trimestre** : non=0, oui=1 l__l MERE_MH24

**Diabète gestationnel** : non=0, oui=1 l__l MERE_MH25

**HTA pendant la grossesse** (PAS>=14mmHg et/ou PAD>=90mmHg) : non=0, oui=1l__l MERE_MH26

**Statut viral :**

HIV positif : non=0, oui=1 l__l MERE_MH27

HBV positif : non=0, oui=1 l__l MERE_MH28

HCV positif : non=0, oui=1 l__l MERE_MH29

**Autres pathologies,**en clair :

………………………………………………………………………………………………………………MERE_MH30

……………………………………………………………………………………………………………………………….

**Autres évènements pendant la grossesse (en dehors des évènements ayant conduits à l’accouchement)**, en clair :

…………………………………………………………………………………………………………………MERE_MH31

Traitements médicamenteux en cours de grossesse Préfixé par MERE_MI

**Traitement(s) médicamenteux pris pendant le 1^er^ trimestre de la grossesse mentionné dans le dossier médical**(y compris traitements hormonaux, ttt des pathologies aigües ou chroniques, anesthésiques…) :

non=0, oui=1 l__l MERE_MI1

Si oui, préciser en clair le(s) traitement(s) :……………………………… …………………….………MERE_MI2

……………………………………………………………………………………………………………………………

Les items suivants concernent les traitements pris après le 1^er^ trimestre, y compris ceux commencés au 1^er^ trimestre :

**Traitement psychotrope mentionné dans le dossier** : non=0, oui=1 l__l MERE_MI3

Si oui : anxiolytiques : non=0, oui=1 l__l MERE_MI4

antidépresseurs : non=0, oui=1 l__l MERE_MI5

somnifères : non=0, oui=1 l__l MERE_MI6

autres en clair : ……………………………… …………………….……… MERE_MI7

…………………………………………………………………………………………………………..MERE_MI8

##

## **Aspirine à faible dose :** non=0, oui=1 l__l MERE_MI9

Si oui, Indication (2 réponses possibles) l__l MERE_MI10 l__l MERE_MI11

*1= ATCD de RCIU ou de pré éclampsie*

*2= thrombophilie et/ou ATCD de pathologie thromboembolique D_INDICATIONASP*

*3= ATCD d’avortements à répétition inexpliqués*

*4= Syndrome des anti-phospholipides ou autre pathologie immunologique*

*5= autre : préciser en clair* MERE_MI12A MERE_MI12B

Date de début l__l__l/ l__l__l /l__l__l__l__l MERE_MI13 **date calculée (SA+J) MERE_MI13A /MERE_MI13B**

Date de fin (si arrêt) l__l__l/ l__l__l /l__l__l__l__l MERE_MI16 **date calculée (SA+J) MERE_MI16A /MERE_MI16B**

**Progestérone :** non=0, oui=1 l__l MERE_MI19

Si oui, Indication  (2 réponses possibles) l__l MERE_MI20 l__l MERE_MI21

*1=ATCD d’accouchement prématuré ou de fausse couche tardive D_INDICATIONPRO*

*2=Col court*

*3=Autre : précisez* ……………………………………… MERE_MI22A MERE_MI22B

Si col court : mesure du col à la mise en place du traitement : l__l__l cm MERE_MI23

Date de début l__l__l/ l__l__l /l__l__l__l__l MERE_MI24 **date calculée (SA+J) MERE_MI24A /MERE_MI24B**

Date de fin l__l__l/ l__l__l /l__l__l__l__l MERE_MI27 **date calculée (SA+J) MERE_MI27A /MERE_MI27B**

Voie d’administration : *1=per os / 2=Intra-Vaginal / 3=IM*  l__l MERE_MI30

*D_ADMINISTRATION*

## **Traitement antihypertenseur :** non=0, oui=1 l__l MERE_MI31

Si oui :

| *1 : Aldomet, 2 : Trandate,3 : Loxen, 4 : Adalate, 5 : Nepressol, 6 : Eupressyl*  *7 : autre traitement à visée antihypertensive à =noter en clair* *D_ANTIHYPERTENSEUR* | **Date de début** | **Date de fin**  **si arrêt avant l’accouchement** | **Mode d’administ.**  1=per os, 2=IV , 3=IM *D_ADMINISTRATIONC* |
| --- | --- | --- | --- |
| MERE_MI32  **autre :** MERE_MI32A | MERE_MI33- **MI33A / B**  l__l__l/l__l__l/l__l__l__l__l | MERE_MI36 **MI36A / B**  l__l/l__l__l/l__l__l__l__l | l__l MERE_MI39 |
| MERE_MI40  **autre :** MERE_MI40A | MERE*_*MI41 **MI41A / B**  l__l__l/l__l__l/l__l__l__l__l | MERE_MI44 **MI44A / B**  l__l/l__l__l/l__l__l__l__l | l__l MERE_MI47 |
| MERE_MI48  **autre :** MERE_MI48A | MERE_MI49 **MI49A / B**  l__l__l/l__l__l/l__l__l__l__l | MERE_MI52 **MI52A / B**  l__l/l__l__l/l__l__l__l__l | l__l MERE_MI55 |

**Les dates calculées (SA+J) ont pour suffixes A & B**

## **Traitement antidiabétique :** non=0, oui=1 l__l MERE_MI64

##

Si oui : Insuline : non=0, oui=1 l__l MERE_MI65

**Traitement immunosuppresseur** (par exemple : corticothérapie au long cours, interféron…)

non=0, oui=1 l__l MERE_MI66

Si oui : Nom commercial et /ou DCI: l__l MERE_MI67

| 1 : prednisone (cortancyl) | 10 : methoject  *D_IMMUNOSUP* |  |
| --- | --- | --- |
| 2 : prednisolone (solupred) | 11 : ciclosporine (neoral, sandimum) | |
| 3 : méthylprednisolone (solumédrol) | 12 : mycophénolate mofétil (cellcept, myfortic) | |
| 4 : béclométhasone (bécotide) | 13 : infliximab (remicade) | |
| 5 : budésonide (pulmicort) | 14 : etanercept (enbrel) | |
| 6 : autres corticoïdes par voie locale (cutanée, nasale, occulaire) | 15 : adalimumab (humira) | |
| 7 : azathioprine (imurel) | 16 : rituximab (mabthera) | |
| 8 : 6-mercaptopurine | 17 : autres | |
| 9 : méthotrexate |  | |

autre : …………………………………………………………………………………………………………………. MERE_MI67A

Date de début l__l__l/ l__l__l /l__l__l__l__l MERE_MI68 **date calculée (SA+J) MERE_MI68A /MERE_MI68B**

Date de fin (si arrêt) l__l__l/ l__l__l /l__l__l__l__l MERE_MI71 **date calculée (SA+J) MERE_MI71A /MERE_MI71B**

Nom commercial et /ou DCI: l__l *D_IMMUNOSUP* MERE_MI74

autre : ………………………………………………………………………………………………………… MERE_MI74A

Date de début l__l__l/ l__l__l /l__l__l__l__ MERE_MI75 **date calculée (SA+J) MERE_MI75A /MERE_MI75B**

Date de fin (si arrêt) l__l__l/ l__l__l /l__l__l__l__l MERE_MI78 **date calculée (SA+J) MERE_MI78A /MERE_MI78B**

**Corticothérapie à visée maturative :** non=0, oui=1 l__l MERE_MI81

Si oui :

Date de la 1^ère^ injection l__l__l/ l__l__l /l__l__l__l__l MERE_MI82

**date calculée (SA+J) MERE_MI82A /MERE_MI82B**

Date de la dernière injection l__l__l/ l__l__l /l__l__l__l__l MERE_MI85

**date calculée (SA+J) MERE_MI85A /MERE_MI85B**

Heure de la dernière injection l__l__l h l__l__l mn MERE_MI88

Corticoïde utilisé pour la dernière injection : l__l MERE_MI90

1 : Bétaméthasone (=Célestène)

2 : Dexaméthasone (=Soludécadron**)** *D_CORTICOIDE*

3 : autre, préciser en clair : …………………………………………… MERE_MI91

Nombre total d’injections  l__l__l MERE_MI92 Nombre de cures complètes l__l MERE_MI93

*(1 cure complète = 2 injections si Béthamétasone ou 4 injections si Déxaméthasone)*

**Variable de synthèse (Equipe CArnaud)**

**Corticothérapie anténatale l__l MERE_STATUTCAN**

| 0="absence de CAN" | *D_STATUTCAN* |
| --- | --- |
| 1="cure incomplète" | 4="2 cures ou plus avec délai <=7 jours" |
| 2="1 cure complète avec délai <=7 jours" | 5="2 cures ou plus avec délai > 7 jours" |
| 3="1 cure complète avec délai > 7 jours" | 6="Corticothérapie anténatale, information incomplète sur nbre/durée/delai des cures" |

**Au moins une cure de corticoïde complète** non=0, oui=1 **l__l MERE_CURECOMP**

**Rescue course** non=0, oui=1 **l__l MERE_RESCUE**

**Autre(s) traitement(s) (en dehors des traitements commencés lors de l’hospitalisation se terminant par l’accouchement :** non=0, oui=1 l__l MERE_MI94

Si oui :

Nom commercial et /ou DCI en clair (1) : ……………………………………………………………………… MERE_MI95

Date de début l__l__l/ l__l__l /l__l__l__l__l MERE_MI96 **date calculée (SA+J) MERE_MI96A /MERE_MI96B**

Date de fin (si arrêt) l__l__l/ l__l__l /l__l__l__l__l MERE_MI99 **date calculée (SA+J) MERE_MI99A /MERE_MI99B**

Nom commercial et /ou DCI en clair (2): ……………………………………………………………………… MERE_MI102

Date de début l__l__l/ l__l__l /l__l__l__l__l MERE_MI103 **date calculée (SA+J) MERE_MI103A /MERE_MI103B**

Date de fin (si arrêt) l__l__l/ l__l__l /l__l__l__l__l MERE_MI106 **date calculée (SA+J) MERE_MI106A /MERE_MI106B**

## **Hospitalisation se terminant par l’accouchement** Préfixé par MERE_MJ

**Parcours de la mère**

**Etablissement d’accouchement**:

Etablissement de naissance : …………………………………………………………………………………………… ENR_ETABNAISS

N° finess : l__l__l__l__|__l__l__l__l__l ENR_FINESSNAISS

Type : 1 : I  / 21 : IIa   / 22 : IIb / 3 : III  *D_ETABNIV*  l__l ENR_NIVEAU2NAISS

Etablissement de naissance anonymisé : l__l__l ENR_NUMETABNAISS

Date d’admission : l__l__l/ l__l__l /l__l__l__l__l MERE_MJ5

**date calculée (SA+J) MERE_MJ5A /MERE_MJ5B**

Heure d’admission (hh:mm) si disponible dans dossier**:** l__l__l h l__l__l mn MERE_MJ8 / 9

**Durée en minutes entre admission et accouchement** l__l__l__l__l **MERE_MJ9A**

Type de transport : 1= personnel / 2= ambulance / 3= SMUR-SAMU / 4= autre  *D_TRANSPORT* l__I MERE_MJ10

Si autre, préciser en clair :………………………………………………………………………………………………… MERE_MJ11

**La patiente était-elle déjà connue de l’établissement d’accouchement pour cette grossesse** (hospitalisation ou consultation) ?  non=0, oui=1 l__l MERE_MJ12

**Transfert in utero (TIU) d’un autre établissement ?** non=0, oui=1 l__l MERE_MJ13

Un transfert est :

- un changement d’établissement d’hospitalisation (n° finess géographique différent)

- par un vecteur de transport spécifique (exclusion du véhicule personnel) : SAMU/SMUR, Ambulance

- avec ou sans accompagnant médicalisé (médecin, sage femmes) ou infirmier

Si transfert in utero :

**Quel est l’établissement d’origine qui transfère ?**

Nom en clair : ……………………………………………………………………………………………………………. MERE_MJ14

N°établissement anonymisé l__l__l MERE_MJ14A

Ville : ……………………………………………………………………………………………………………………….. MERE_MJ15

N° finess : l__l__l__l__|__l__l__l__l__l MERE_MJ16

Type : 1 : I  / 21 : IIa   / 22 : IIb / 3 : III  *D_ETABNIV* l__l MERE_MJ17

**Durée de l’hospitalisation dans cet établissement avant transfert**:

**< 24h**: non=0, oui=1 l__l MERE_MJ18

**Si >24h,** nombre de jours : l__l__l MERE_MJ19

**Traitement tocolytique pendant le transfert :** non=0, oui=1 l__l MERE_MJ20

**Traitement antihypertenseur pendant le transfert :** non=0, oui=1 l__l MERE_MJ21

**L’enfant est-il vivant au moment du transfert ?** non=0, oui=1 l__l MERE_MJ22

Si absence de transfert in utero :

**La patiente a-t-elle été orientée ou adressée par un professionnel de santé, à la maternité où elle a accouché, pour une raison médicale maternelle ou fœtale ?** non=0, oui=1 l__l MERE_MJ22A

*Une orientation est un changement de maternité de naissance par rapport au choix initialement prévu, pour des raisons médicales, à l’exclusion des transferts in utero.*

**Si l’établissement d’accouchement n’est pas de type III pour une naissance < 33 SA** :

**Motif d’accouchement d’un prématuré de <33SA dans un établissement de type I ou II :**

non=0, oui=1 l__l MERE_MJ23

Si oui :

Hospitalisation en accord avec les recommandations du réseau : non=0, oui=1 l__l MERE_MJ24

Manque de place en type III : maternité et /ou en service de néonatologie : non=0, oui=1 l__l MERE_MJ25

Manque de ressource de transfert (véhicule/personnel) : non=0, oui=1 l__l MERE_MJ26

Accouchement inopiné : non=0, oui=1 l__l MERE_MJ27

MFIU : non=0, oui=1 l__l MERE_MJ28

Autre : non=0, oui=1 l__l MERE_MJ29

Si autre, préciser : ……………………………………………………………………………………………………………. MERE_MJ30

## **Cas particulier d’admission (hors transfert in utero), préciser en clair** :

## ……………………………………………………………………………………………………………………………………………… MERE_MJ31

**Pathologies présentes à l’admission ou au cours de l’hospitalisation**

## **Rupture prématurée des membranes (RPM) :** non=0, oui=1 l__l MERE_MJ32

## (Rupture des membranes au moins 12h avant l’entrée en travail)

Si oui,

Date de la RPM __l__l/ l__l__l /l__l__l__l__l MERE_MJ33 **date calculée (SA+J) MERE_MJ33A /MERE_MJ33B**

Heure de la RPM l__l__l h l__l__l mn MERE_MJ36 / 37

**Durée en minutes entre RPM et accouchement** l__l__l__l__l **MERE_MJ37A**

Bilan à l’admission de l’hospitalisation pour RPM (ou lors de la RPM si elle survient en cours d’hospitalisation pour MAP) :

CRP : l__l__l__l mg/l MERE_MJ38

Leucocytes : l__l__l__l__l__l__l mm^3^ MERE_MJ39

Température corporelle : l__l__l , l__l °C MERE_MJ40

Prélèvement Vaginal fait : non=0, oui=1 l__l MERE_MJ41

Si oui, germes(s) retrouvé(s) : non=0, oui=1 l__l MERE_MJ42

si oui , 2 germes possibles l__l MERE_MJ43

l__l MERE_MJ44

1 : Escherichia coli 4 : Candida, autre mycose D_GERME

2 : Streptocoque B 5 : Autre bacille gram négatif

3 : Entérocoque 6 : Autre cocci gram positif

7 : Autre germe

| *Variable corrigée Elsa, tronc commun* |  |
| --- | --- |
| **Age gestationnel à la rupture des membranes** | **mere_ag_rupture** |

## **Menace d’accouchement prématuré (MAP) à membranes intactes :** non=0, oui=1 l__l MERE_MJ45

## (= Modifications cervicales et / ou contractions utérines)

## **Date du 1^er^ diagnostic de MAP au cours de cette grossesse**  l__l__l/ l__l__l /l__l__l__l__l MERE_MJ46

**date calculée (SA+J) MERE_MJ46A /MERE_MJ46B**

## **A l’admission,**

**TV fait ?** non=0, oui=1 l__l MERE_MJ49

## Si oui,

## **Longueur du col** : 1 : long / 2 : mi-long / 3 : court D_LONGCOL l__l MERE_MJ50

**Etat du col** : 1 : fermé / 2 : ouvert à 1 cm / 3 : ouvert à 2 cm ou plus *D_ETATCOL* l__l MERE_MJ51

**Mesure échographique de la longueur du col mentionnée dans le dossier** : non=0, oui=1 l__l MERE_MJ52

Si oui, valeur : l__l__l mm MERE_MJ53

**Métrorragies associées** : non=0, oui=1 l__l MERE_MJ54

**Contractions utérines (CU) douloureuses ressenties** : non=0, oui=1 l__l MERE_MJ55

**Nombre de CU au monitorage sur 20 minutes :** l__l__l MERE_MJ56

**Pathologies hypertensives de la grossesse :**

## **HTA** (PAS >140 mm Hg et/ou PAD >90 mm Hg) **:** non=0, oui=1 l__l MERE_MJ57

Si oui, date de diagnostic : l__l__l/ l__l__l /l__l__l__l__l MERE_MJ58 **date calculée (SA+J) MERE_MJ58A /MERE_MJ58B**

## **Pré-éclampsie**(HTA + protéinurie > 0,3g/24h)**:** non=0, oui=1 l__l MERE_MJ61

Si oui, date de diagnostic : l__l__l/ l__l__l /l__l__l__l__l MERE_MJ62 **date calculée (SA+J) MERE_MJ62A /MERE_MJ62B**

## **Eclampsie** (survenue d’une crise convulsive tonico-clonique dans un contexte de pathologie hypertensive de la grossesse) **:** non=0, oui=1 l__l MERE_MJ65

## Si oui, date de diagnostic : l__l__l/ l__l__l /l__l__l__l__l MERE_MJ66 **date calculée (SA+J) MERE_MJ66A /MERE_MJ66B**

## **HELLP syndrome**(association d’une hémolyse, une cytolyse hépatique et d’une thrombopénie) **:**

## non=0, oui=1 l__l MERE_MJ69

## Si oui, date de diagnostic : l__l__l/ l__l__l /l__l__l__l__l MERE_MJ70 **date calculée (SA+J) MERE_MJ70A /MERE_MJ70B**

## **Hématome rétro-placentaire (HRP) :** non=0, oui=1 l__l MERE_MJ73

## **Infection bactérienne:** non=0, oui=1 l__l MERE_MJ74

Si oui : préciser (3 réponses possibles) l__l MERE_MJ75A l__l MERE_MJ75B l__l MERE_MJ75C

| 1 : infection urinaire basse | 6 : infection pulmonaire | *D_INFECTIONBACTE* |
| --- | --- | --- |
| 2 : infection haute | 7 : infection digestive |  |
| 3 : infection vaginale | 8 : infection ostéo-articulaire |  |
| 4 : méningite/méningo-encéphalite | 9 : autre, préciser |  |
| 5 : infection orl |  |  |

Si autre : préciser : ………………………………………………………………………………………… MERE_MJ75D ..………………………………………………………………………………………… MERE_MJ75E

## **Diagnostic ou suspicion de « Retard de Croissance Intra Utérine» (RCIU) ou « petit poids pour l’âge gestationnel » (PPAG) de l’enfant (ou 1^er^ fœtus si grossesse multiple) :**

## non=0, oui=1 l__l MERE_MJ76

Si oui,

## Date de diagnostic : l__l__l/ l__l__l /l__l__l__l__l MERE_MJ77 **date calculée (SA+J) MERE_MJ77A /MERE_MJ77B**

Poids fœtal estimé (PFE): l__l__l__l__l g MERE_MJ80

Percentile de poids retenu pour le diagnostic : l__l__l MERE_MJ81

ou

Tranche de percentile si valeur exacte non mentionnée : l__l MERE_MJ82

1 : PFE < 3^ème^ percentile

2 : < 5^ème^ percentile *D_PERCENTILE*

3 : < au 10^ème^ percentile

Périmètre abdominal (PA) : l__l__l__l mm MERE_MJ83

Percentile de PA retenu pour le diagnostic : l__l__l MERE_MJ84

ou

Tranche de percentile si valeur exacte non mentionnée l__l MERE_MJ85

1 : PA < 3^ème^ percentile *D_PERCENTILE*

2 : < 5^ème^ percentile

3 : < au 10^ème^ percentile

Arrêt de croissance (ou cassure de la courbe) diagnostiqué(e) par l’équipe :

non=0, oui=1 l__l MERE_MJ86

## **Diagnostic ou suspicion de « Retard de Croissance Intra Utérine» (RCIU) ou « petit poids pour l’âge gestationnel » (PPAG) du 2^ème^ fœtus si grossesse multiple :** non=0, oui=1 l__l MERE_MJ87

Si oui,

## Date de diagnostic : l__l__l/ l__l__l /l__l__l__l__l MERE_MJ88 **date calculée (SA+J) MERE_MJ88A /MERE_MJ88B**

Poids fœtal estimé (PFE) l__l__l__l__l g MERE_MJ91

Percentile de poids retenu pour le diagnostic : l__l__l MERE_MJ92

ou

Tranche de percentile si valeur exacte non mentionnée l__l MERE_MJ93

1 : PFE < 3^ème^ percentile

2 : < 5^ème^ percentile *D_PERCENTILE*

3 : < au 10^ème^ percentile

Périmètre abdominal (PA) l__l__l__l mm MERE_MJ94

Percentile de PA retenu pour le diagnostic : l__l__l MERE_MJ95

ou

Tranche de percentile si valeur exacte non mentionnée l__l MERE_MJ96

1 : PA < 3^ème^ percentile

2 : < 5^ème^ percentile *D_PERCENTILE*

3 : < au 10^ème^ percentile

Arrêt de croissance (ou cassure de la courbe) diagnostiqué(e) par l’équipe : non=0, oui=1 l__l MERE_MJ97

## **Diagnostic ou suspicion de « Retard de Croissance Intra Utérine» (RCIU) ou « petit poids pour l’âge gestationnel » (PPAG) du 3^ème^ fœtus si grossesse multiple :** non=0, oui=1 l__l MERE_MJ98

Si oui,

## Date de diagnostic : l__l__l/ l__l__l /l__l__l__l__l MERE_MJ99 **date calculée (SA+J) MERE_MJ99_1 /MERE_MJ99_2**

Poids fœtal estimé (PFE) l__l__l__l__l g MERE_MJ102

Percentile de poids retenu pour le diagnostic : l__l__l MERE_MJ103

ou

Tranche de percentile si valeur exacte non mentionnée l__l MERE_MJ104

1 : PFE < 3^ème^ percentile

2 : < 5^ème^ percentile *D_PERCENTILE*

3 : < au 10^ème^ percentile

Périmètre abdominal (PA) l__l__l__l mm MERE_MJ105

Percentile de PA retenu pour le diagnostic : l__l__l MERE_MJ106

ou

Tranche de percentile si valeur exacte non mentionnée l__l MERE_MJ107

1 : PA < 3^ème^ percentile

2 : < 5^ème^ percentile *D_PERCENTILE*

3 : < au 10^ème^ percentile

Arrêt de croissance (ou cassure de la courbe) diagnostiqué(e) par l’équipe : non=0, oui=1 l__l MERE_MJ108

## **Diagnostic ou suspicion de « Retard de Croissance Intra Utérine» (RCIU) ou « petit poids pour l’âge gestationnel » (PPAG) du 4^ème^ fœtus si grossesse multiple :** non=0, oui=1 l__l MERE_MJ98B

Si oui,

## Date de diagnostic : l__l__l/ l__l__l /l__l__l__l__l MERE_MJ99B

## **date calculée (SA+J) MERE_MJ99B_1 /MERE_MJ99B_2**

Poids fœtal estimé (PFE) l__l__l__l__l g MERE_MJ102B

Percentile de poids retenu pour le diagnostic : l__l__L MERE_MJ103B

ou

Tranche de percentile si valeur exacte non mentionnée l__l MERE_MJ104B

1 : PFE < 3^ème^ percentile

2 : < 5^ème^ percentile *D_PERCENTILE*

3 : < au 10^ème^ percentile

Périmètre abdominal (PA) l__l__l__l mm MERE_MJ105B

Percentile de PA retenu pour le diagnostic : l__l__l MERE_MJ106B

ou

Tranche de percentile si valeur exacte non mentionnée l__l MERE_MJ107B

1 : PA < 3^ème^ percentile

2 : < 5^ème^ percentile *D_PERCENTILE*

3 : < au 10^ème^ percentile

Arrêt de croissance (ou cassure de la courbe) diagnostiqué(e) par l’équipe : non=0, oui=1I__lMERE_MJ108B

## **Décompensation d’une pathologie chronique maternelle préexistante :**

## non=0, oui=1 l__l MERE_MJ109

Si oui, préciser en clair ……………………………………………………………………………………………………… MERE_MJ110

Y avait-il eu un suivi spécialisé de cette pathologie pendant la grossesse : non=0, oui=1 l__l MERE_MJ111

**Traitements médicamenteux au cours de l’hospitalisation se terminant par l’accouchement (dernière hospitalisation)**

## **Tocolyse au cours de la dernière hospitalisation :** non=0, oui=1 l__l MERE_MJ112

Si oui,

| **Nom commercial / DCI d_tocolyse** | **Mode d’administ.**  **d_administrationb** | **Date de début** | **Date de fin**  **si arrêt avant l’accouchement** |
| --- | --- | --- | --- |
| MERE_MJ113  **autre :** MERE_MJ113A | MERE_MJ114 l__l | MERE_MJ115 **MJ115A / B**  l__l__l/l__l__l/l__l__l__l__l | MERE_MJ118 **MJ118A / B** l__l__l/l__l__l/l__l__l__l__l |
| MERE_MJ121  **autre :** MERE_MJ121A | MERE_MJ122 l__l | MERE_MJ123 **MJ123A / B**  l__l__l/l__l__l/l__l__l__l__l | MERE_MJ126 **MJ126A / B** L_l__l/l__l__l/l__l__l__l__l |
| MERE_MJ129  **autre :** MERE_MJ129A | MERE_MJ130 l__l | MERE_MJ131 **MJ131A / B**  l__l__l/l__l__l/l__l__l__l__l | MERE_MJ134 **MJ134A / B** L_l__l/l__l__l/l__l__l__l__l |

**Les dates calculées (SA+J) ont pour suffixes A & B**

| *D_TOCOLYSE* |  | *D_ADMINISTRATIONB* | | |  |
| --- | --- | --- | --- | --- | --- |
| 1 : atosiban (tractocile) |  | 1 : per os | | |  |
| 2 : nicardipine (loxen) |  | 2 : IV | | |  |
| 3 : nifedipine (adalate) | | |  |  | |
| 4 : salbutamol (salbumol) | | |  |  | |
| 5 : autre | | |  |  | |

**Arrêt de la tocolyse** : non=0, oui=1 l__l MERE_MJ137

Si oui, Date de l’arrêt définitif : l__l__l/ l__l__l /l__l__l__l__l MERE_MJ138

**date calculée (SA+J) MERE_MJ138A /MERE_MJ138B**

Motif de l’arrêt :

Fin du traitement : non=0, oui=1 l__l l__l MERE_MJ141

Signe clinique d’infection : non=0, oui=1 l__l MERE_MJ142

Signe biologique d’infection : non=0, oui=1 l__l MERE_MJ143

Effet secondaire nécessitant l’arrêt du traitement : non=0, oui=1 l__l MERE_MJ144

Si effet secondaire, préciser en clair ………………………………………… MERE_MJ145

Rupture des membranes : non=0, oui=1 l__l MERE_MJ146

Autre : non=0, oui=1 l__l MERE_MJ147

Si autre, précisez en clair ………………………………………………………… MERE_MJ148

##

## **Traitement anti-infectieux au cours de la dernière hospitalisation :** non=0, oui=1 l__l MERE_MJ149

Si oui,

| Nom commercial / DCI **d_anti_infect** | Mode admin°  **d_administrationc** | Date de début | Date de fin (si arrêt)  *(même que date de début si dose unique)* | Indication :  **d_indicinfect** | Agent(s) infectieux identifié(s)non=0, oui=1 | Si oui, type d’agent(s) infectieux identifié(s)  en clair |
| --- | --- | --- | --- | --- | --- | --- |
| **mere_mj150**  **autre : mere_mj150a** | **mere_mj151** | **mere_mj152 MJ152A / B**  l__l__l/l__l__l/l__l__l__l__ | **mere_mj155 MJ155A / B** l__l__l/l__l__l/l__l__l__l__ | L__l **mere_mj158** | **mere_mj159** | **mere_mj160** |
|  |  |  |  | **autre : mere_mj158a** |  |  |
| **mere_mj161**  **autre : mere_mj161a** | **mere_mj162** | **mere_mj163 MJ163A / B**  l__l__l/l__l__l/l__l__l__l__ | **mere_mj166** **MJ166A / B** l__l__l/l__l__l/l__l__l__l__ | l__l **mere_mj169** | **mere_mj170** | **mere_mj171** |
|  |  |  |  | **autre : mere_mj169a** |  |  |
| **mere_mj172**  **autre : mere_mj172a** | **mere_mj173** | **mere_mj174** **MJ174A / B** l__l__l/l__l__l/l__l__l__l__ | **mere_mj177** **MJ177A / B** l__l__l/l__l__l/l__l__l__l__ | l__l **mere_mj180**  **autre : mere_mj180a** | **mere_mj181** | **mere_mj182** |

**Les dates calculées (SA+J) ont pour suffixes A & B**

| *D_ANTI_INFECT* | *D_ADMINISTRATIONC* | *D_INDICINFECT* |
| --- | --- | --- |
| 1 : amoxicilline (clamoxyl) | 1 : per os | 1 : RPM |
| 2 : amoxicilline +acide clavulanique (augmentin) | 2 : IV | 2 : prélèvement vaginal (pv) positif |
| 3 : pénicilline v et g (oracilline, extencilline) | 3 : IM | 3 : MAP à mb intacte |
| 4 : céfixime (oroken) |  | 4 : infection urinaire basse |
| 5 : ceftriaxone (rocéphine)  6 : céfotaxime (claforan) |  | 5 : autre |
| 7 : erythromycine (erythrocine) |  |  |
| 8 : spiramycine (rovamycine) |  |  |
| 9 : josamycine (josacine) |  |  |
| 10 : clindamycine (dalacine) |  |  |
| 11 : autre traitement anti-infectieux |  |  |

**Administration anténatale de Sulfate de Magnésium au cours de la dernière hospitalisation :**

non=0, oui=1 l__l MERE_MJ183

Si oui,

Indication : l__l MERE_MJ184

préeclampsie = 1 / neuroprotection = 2 / autre = 3 *D_INDICSULFMAG*

Date de la 1^ère^ administration :  l__l__l/ l__l__l /l__l__l__l__l MERE_MJ185

**date calculée (SA+J) MERE_MJ185A /MERE_MJ185B**

Dose totale reçue : l__l__l__l g MERE_MJ188

# Derniers examens avant le travail ou la césarienne : principaux résultats Préfixé par MERE_MK

**Résultats cliniques :**

**Date :**  l__l__l/ l__l__l /l__l__l__l__l MERE_MK1 **date calculée (SA+J) MERE_MK1A /MERE_MK1B**

**Heure :** l__l__l h l__l__l mn MERE_MK4 / MK5

**Durée en minutes entre derniers résultats clin. et accouchement** l__l__l__l__l **MERE_MK5A**

**Température maternelle** : l__l__| , l__l C° MERE_MK6

**Douleur abdominale (ou utérine)** : non=0, oui=1 l__l MERE_MK7

**Si rupture, couleur du liquide amniotique :** l__l MERE_MK8

clair=1 / teinté=2 / sanglant=3 / méconial=4 / purulent=5 *D_AMNIOCOLOR*

**Tachycardie maternelle** (FC>90/min) : non=0, oui=1 l__l MERE_MK9

**L’équipe juge-t-elle qu’il y a une chorioamniotite dans les 48 h précédant l’accouchement ?**

non=0, oui=1 l__l MERE_MK10

**Derniers résultats biologiques avant l’accouchement :**

**Date :** l__l__l/ l__l__l /l__l__l__l__l MERE_MK11 **date calculée (SA+J) MERE_MK11A /MERE_MK11B**

**Heure :** l__l__l h l__l__l mn MERE_MK14 / MK15

**Durée en minutes entre derniers résultats bio. et accouchement** l__l__l__l__l **MERE_MK15A**

**CRP**: l__l__l__l mg/l MERE_MK16

**Leucocytes :** l__l__l l__l__l__l mm^3^ MERE_MK17

**Créatininémie :** l__l__l__l µmol/l MERE_MK18

En cas d’HTA essentielle, HTA gravidique, prééclampsie, eclampsie, HELLP syndrome, HRP, RCIU :

non=0, oui=1 l__l MERE_MK19

Si oui : **Derniers éléments cliniques et biologiques :**

Date du dernier bilan : l__l__l/ l__l__l /l__l__l__l__l MERE_MK20 **date calculée (SA+J) MERE_MK20A /MERE_MK20B**

TAS > 140mmHg et /ou TAD > 90mmHg : non=0, oui=1 l__l MERE_MK23

Si oui, TAS la plus élevée : l__l__l__l mmHg MERE_MK24

TAD la plus élevée : l__l__l__l mmHg MERE_MK25

Dernière protéinurie / 24h : l__l , l__l g/l MERE_MK26

Oligurie : non=0, oui=1 l__l MERE_MK27

Dernière diurèse : l__l__l__l ml/24h MERE_MK28

Œdème pulmonaire : non=0, oui=1 l__l MERE_MK29

Barre épigastrique : non=0, oui=1 l__l MERE_MK30

Survenue d’une crise convulsive tonico-clonique : non=0, oui=1 l__l MERE_MK31

Dernière créatininémie : l__l__l__l µmol/l MERE_MK32

Troubles neurologiques (troubles visuels, ROT polycinétiques, Céphalées) : non=0, oui=1 l__l MERE_MK33

Dernier taux de plaquettes : l__l__l__l__l__l__l / mm^3^ MERE_MK34

Derniers taux de transaminases : ASAT :   l__l__l__l UI/l MERE_MK35

ALAT :  l__l__l__l UI/l MERE_MK36

Hématome rétroplacentaire (HRP) : non=0, oui=1 l__l MERE_MK37

**Grossesse Unique ou J1/T1 si grossesse multiple :**

**CHORIOAMNIOTITE CLINIQUE (Concerne les enfants inclus du tronc commun, créée par Héloïse Torchin)**

**Chorioamniotite clinique**  **mere_chorio_clinique**

| 0 : Pas de chorioamniotite clinique |  |
| --- | --- |
| 1 : Chorioamniotite clinique : T° maternelle ≥ 37.8°C + ≥ 2 critères parmi : tachycardie maternelle, tachycardie fœtale, LA purulent, douleurs utérines, hyperleucocytose maternelle ≥ 15 000/mm^3^ |  |
|  |  |
|  |  |

**Dernière écho-doppler, ou écho-doppler décisionnelle si ce n’est pas la dernière**

**Date:** l__l__l/ l__l__l /l__l__l__l__l MERE_MK38 **date calculée (SA+J) MERE_MK38_1 /MERE_ MK38_2**

**et heure** l__l__l h l__l__l mn MERE_MK41 / MK42

**Durée en minutes entre dernière echo-doppler et accouchement J1** l__l__l__l__l **MERE_MK42A**

**Poids fœtal estimé (PFE) (de J1 si multiple)** l__l__l__l__lg MERE_MK43

**Périmètre abdominal (PA) (de J1 si multiple)** l__l__l__l mm MERE_MK44

**Diagnostic de RCIU posé ?** non=0, oui=1 l__l MERE_MK45

Si oui : sur PFE = 1 / sur  PA = 2 / sur  les deux = 3 / autre = 4 *D_RCIU* l__l MERE_MK46

**Périmètre crânien (PC) (de J1 si multiple)** l__l__l__l mm MERE_MK47

**Longueur du fémur (de J1 si multiple)** l__l__l__l mm MERE_MK48

**Oligoamnios** **(de J1 si multiple)** (grande citerne<2cm ou index amniotique <5) : non=0, oui=1 l__l MERE_MK49

**Diminution des mouvements fœtaux (de J1 si multiple)** **:** non=0, oui=1 l__l MERE_MK50

**Doppler ombilical (de J1 si multiple) fait** :  non=0, oui=1 l__l MERE_MK51

Si oui, résultat : l__l MERE_MK52

1=Normal / 2= Pathologique avec diastole conservée / 3=Diastole nulle / 4=Reverse flow *D_DOPPLEROMB*

**Doppler cérébral**  **(de J1 si multiple)** **fait**:   non=0, oui=1 l__l MERE_MK53

Si oui, résultat : l__l MERE_ MK54

1=normal / 2=Redistribution cérébrale / 3=Vasodilatation sans redistr° cérébrale  *D_DOPPLERCERE*

**Ductus Venosus** **(de J1 si multiple) fait**:   non=0, oui=1 l__l MERE_MK55

Si oui, onde «a » : l__l MERE_MK56

1=Normale / 2=Diminuée / 3=Nulle / 4= « reverse flow **»** *D_DUCTUS*

**Arrêt de la croissance (de J1 si multiple)** **jugée sur mesures biométriques :** non=0, oui=1 l__l MERE_MK57

Si oui,  délai en jours ayant permis de poser le diagnostic : l__l__l j MERE_MK58

**Dernier RCF (Rythme Cardiaque Fœtal)**

**Date du dernier enregistrement :** l__l__l/ l__l__l /l__l__l__l__l MERE_MK59

**date calculée (SA+J) MERE_MK59A /MERE_MK59B**

**heure du dernier enregistrement** l__l__l h l__l__l mn MERE_MK62 / 63

**Durée en minutes entre dernier RCF et accouchement J1** l__l__l__l__l **MERE_MK63A**

**Normal (pour J1 si multiple) ?** : non=0, oui=1 l__l MERE_MK64

Si non,

**Plat ou très peu oscillant** **(pour J1 si multiple) :** non=0, oui=1 l__l MERE_MK65

**Tachycardie (pour J1 si multiple)** (fréquence cardiaque supérieure à 160/min) :

non=0, oui=1 l__l MERE_MK66

**Décélérations (pour J1 si multiple)** **?** : non=0, oui=1 l__l MERE_MK67

**VCT  faite (Variabilité à court terme, par analyse informatisée du RCF) :** non=0, oui=1 l__l MERE_MK68

Si oui, résultat du dernier VCT (pour J1 si multiple) : l__l , l__l ms MERE_MK69

date  l__l__l/ l__l__l /l__l__l__l__l MERE_MK70 **date calculée (SA+J) MERE_MK70A/MERE_MK70B**

et heure : l__l__l h l__l__l mn MERE_MK73 / 74

**Durée en minutes entre VCT et accouchement J1** l__l__l__l__l **MERE_MK74A**

**SI GROSSESSE MULTIPLE : J2 / T2**

**Dernière écho-doppler, ou écho-doppler décisionnelle si ce n’est pas la dernière**

**Date:** l__l__l/ l__l__l /l__l__l__l__l MERE_MK75 **date calculée (SA+J) MERE_MK75A /MERE_MK75B**

**et heure** l__l__l h l__l__l mn MERE_MK78 / 79

**Durée en minutes entre dernière echo-doppler et accouchement J2** l__l__l__l__l **MERE_MK79A**

**Poids fœtal estimé (PFE) de J2** l__l__l__l__lg MERE_MK80

**Périmètre abdominal (PA) de J2** l__l__l__l mm MERE_MK81

**Diagnostic de RCIU posé pour J2 ?** non=0, oui=1 l__l MERE_MK82

Si oui : sur PFE = 1 / sur  PA = 2 / sur  les deux = 3 / autre = 4 *D_RCIU* l__l MERE_MK83

**Périmètre crânien (PC) de J2** l__l__l__l mm MERE_MK84

**Longueur du fémur de J2** l__l__l__l mm MERE_MK85

**Oligoamnios** **de J2** (grande citerne<2cm ou index amniotique <5) : non=0, oui=1 l__l MERE_MK86

**Diminution des mouvements fœtaux de J2** **:** non=0, oui=1 l__l MERE_MK87

**Doppler ombilical de J2** **fait** :  non=0, oui=1 l__l MERE_MK88

Si oui, résultat: 1=Normal / 2=Pathologique avec diastole conservée / 3=Diastole nulle / 4=Reverse flow l__l

*D_DOPPLEROMB* MERE_MK89

**Doppler cérébral** **de J2** **fait**:   non=0, oui=1 l__l MERE_MK90

Si oui, résultat : 1=normal / 2=Redistribution cérébrale / 3=Vasodilatation sans redistr° cérébrale  l__l

*D_DOPPLERCERE* MERE_MK91

**Ductus Venosus** **de J2** **fait**:   non=0, oui=1 l__l MERE_MK92

Si oui, onde «a » : 1=Normale / 2=Diminuée / 3=Nulle / 4= « reverse flow »  *D_DUCTUS* l__l MERE_MK93

**Arrêt de la croissance de J2 :**   non=0, oui=1 l__l MERE_MK94

Si oui,  délai en jours ayant permis de poser le diagnostic l__l__l j MERE_MK95

**Dernier RCF (Rythme Cardiaque Fœtal)**

**Date du dernier enregistrement :** l__l__l/ l__l__l /l__l__l__l__l MERE_MK96 **date calculée (SA+J) MERE_MK96A /MERE_MK96B**

**heure du dernier enregistrement** l__l__l h l__l__l mn MERE_MK99 / 100

**Durée en minutes entre dernier RCF et accouchement J2** l__l__l__l__l **MERE_MK100A**

**Normal pour J2** : non=0, oui=1 l__l MERE_MK101

Si non,

**Plat ou très peu oscillant** **de J2** : non=0, oui=1 l__l MERE_MK102

**Tachycardie de J2** (**fréquence cardiaque supérieure à 160/min)**: non=0, oui=1 l__l MERE_MK103

**Décélérations de J2**: non=0, oui=1 l__l MERE_MK104

**VCT  faite (Variabilité à court terme, par analyse informatisée du RCF) :** non=0, oui=1 l__l MERE_MK105

Si oui, résultat du dernier VCT de J2 : l__l , l__l ms MERE_MK106

date : l__l__l/ l__l__l /l__l__l__l__l MERE_MK107 **date calculée (SA+J) MERE_MK107A/MERE_MK107B**

et heure  l__l__l h l__l__l mn MERE_MK110 / 111

**Durée en minutes entre VCT et accouchement J2** l__l__l__l__l **MERE_MK111A**

**SI GROSSESSE MULTIPLE : T3**

**Dernière écho-doppler, ou écho-doppler décisionnelle si ce n’est pas la dernière**

**Date** l__l__l/ l__l__l /l__l__l__l__l MERE_MK112 **date calculée (SA+J) MERE_MK112A /MERE_MK112B**

**et heure :** l__l__l h l__l__l mn MERE_MK115/ 116

**Durée en minutes entre dernière echo-doppler et accouchement T3** l__l__l__l__l **MERE_MK116A**

**Poids fœtal estimé (PFE) de T3** l__l__l__l__lg MERE_MK117

**Périmètre abdominal (PA) de T3** l__l__l__l mm MERE_MK118

**Diagnostic de RCIU posé pour T3 ?** non=0, oui=1 *D_RCIU* l__l MERE_MK119

Si oui : sur PFE = 1 / sur  PA = 2 / sur  les deux = 3 / autre = 4 l__l MERE_MK120

**Périmètre crânien (PC) de T3** l__l__l__l mm MERE_MK121

**Longueur du fémur de T3** l__l__l__l mm MERE_MK122

**Oligoamnios** **de T3** (grande citerne<2cm ou index amniotique <5) : non=0, oui=1 l__l MERE_MK123

**Diminution des mouvements fœtaux de T3** **:** non=0, oui=1 l__l MERE_MK124

**Doppler ombilical de T3** **fait** :  non=0, oui=1 l__l MERE_MK125

Si oui, résultat: 1=Normal / 2= Pathologique avec diastole conservée / 3=Diastole nulle / 4=Reverse flow l__l

*D_DOPPLEROMB* MERE_MK126

**Doppler cérébral** **de T3** **fait**:   non=0, oui=1 l__l MERE_MK127

Si oui, résultat : 1=normal / 2=Redistribution cérébrale / 3=Vasodilatation sans redistr° cérébrale  l__l

*D_DOPPLERCERE* MERE_MK128

**Ductus Venosus** **de T3** **fait**:   non=0, oui=1 l__l MERE_MK129

Si oui, onde «a » : 1=Normale / 2=Diminuée / 3=Nulle / 4= « reverse flow »  *D_DUCTUS* l__l MERE_MK130

**Arrêt de la croissance de T3 :**    non=0, oui=1 l__l MERE_MK131

Si oui,  délai en jours ayant permis de poser le diagnostic l__l__l j MERE_MK132

**Dernier RCF (Rythme Cardiaque Fœtal)**

**Date du dernier enregistrement :** l__l__l/ l__l__l /l__l__l__l__l MERE_MK133

**date calculée (SA+J) MERE_MK133A /MERE_MK133B**

**et heure du dernier enregistrement** l__l__l h l__l__l mn MERE_MK136 / 137

**Durée en minutes entre dernier RCF et accouchement T3** l__l__l__l__l **MERE_MK137A**

**Normal pour T3** : non=0, oui=1 l__l MERE_MK138

Si non,

**Plat ou très peu oscillant** : non=0, oui=1 l__l MERE_MK139

**Tachycardie de T3**  (**fréquence cardiaque supérieure à 160/min)**: non=0, oui=1 l__l MERE_MK140

**Décélérations de T3**: non=0, oui=1 l__l MERE_MK141

**VCT  faite (Variabilité à court terme, par analyse informatisée du RCF) :** non=0, oui=1 l__l MERE_MK142

Si oui, résultat du dernier VCT de T3 : l__l , l__l ms MERE_MK143

Date  l__l__l/ l__l__l /l__l__l__l__l MERE_MK144 **date calculée (SA+J) MERE_MK144A /MERE_MK144B**

et heure : l__l__l h l__l__l mn MERE_MK147 / 148

**Durée en minutes entre VCT et accouchement T3** l__l__l__l__l **MERE_MK148A**

**SI GROSSESSE MULTIPLE : T4**

**Dernière écho-doppler, ou écho-doppler décisionnelle si ce n’est pas la dernière**

**Date** l__l__l/ l__l__l /l__l__l__l__l MERE_MK149 **date calculée (SA+J) MERE_MK149A /MERE_MK1494B**

**et heure :** l__l__l h l__l__l mn MERE_MK152 / 153

**Durée en minutes entre dernière echo-doppler et accouchement T4** l__l__l__l__l **MERE_MK153A**

**Poids fœtal estimé (PFE) de T4** l__l__l__l__lg MERE_MK154

**Périmètre abdominal (PA) de T4** l__l__l__l mm MERE_MK155

**Diagnostic de RCIU posé pour T4 ?** non=0, oui=1 l__l MERE_MK156

Si oui : sur PFE = 1 / sur  PA = 2 / sur  les deux = 3 / autre = 4 *D_RCIU* l__l MERE_MK154

**Périmètre crânien (PC) de T4** l__l__l__l mm MERE_MK158

**Longueur du fémur de T4** l__l__l__l mm MERE_MK159

**Oligoamnios** **de T4** (grande citerne<2cm ou index amniotique <5) : non=0, oui=1 l__l MERE_MK160

**Diminution des mouvements fœtaux de T4** **:** non=0, oui=1 l__l MERE_MK161

**Doppler ombilical de T4** **fait** :  non=0, oui=1 l__l MERE_MK162

Si oui, résultat: 1=Normal / 2= Pathologique avec diastole conservée / 3=Diastole nulle / 4=Reverse flow l__l

*D_DOPPLEROMB* MERE_MK163

**Doppler cérébral** **de T4** **fait**:   non=0, oui=1 l__l MERE_MK164

Si oui, résultat : 1=normal / 2=Redistribution cérébrale / 3=Vasodilatation sans redistr° cérébrale  l__l

*D_DOPPLERCERE* MERE_MK165

**Ductus Venosus** **de T4** **fait**:  non=0, oui=1 l__l MERE_MK166

Si oui, onde «a » : 1=Normale / 2=Diminuée / 3=Nulle / 4= « reverse flow » *D_DUCTUS* l__l MERE_MK167

**Arrêt de la croissance de T4 :**    non=0, oui=1 I__IMERE_MK168

Si oui,  délai en jours ayant permis de poser le diagnostic l__l__l j MERE_MK169

**Dernier RCF (Rythme Cardiaque Fœtal)**

**Date du dernier enregistrement :** l__l__l/ l__l__l /l__l__l__l__l MERE_MK170 **date calculée (SA+J) MERE_MK170A /MERE_MK170B**

**et heure du dernier enregistrement :** l__l__l h l__l__l mn MERE_MK173 / 174

**Durée en minutes entre dernier RCF et accouchement T4** l__l__l__l__l **MERE_MK174A**

**Normal pour T4** : non=0, oui=1 l__l MERE_MK175

Si non,

**Plat ou très peu oscillant** : non=0, oui=1 l__l MERE_MK176

**Tachycardie de T4**  (**fréquence cardiaque supérieure à 160/min)**: non=0, oui=1 l__l MERE_MK177

**Décélérations de T4**: non=0, oui=1 l__l MERE_MK178

**VCT  faite (Variabilité à court terme, par analyse informatisée du RCF) :** non=0, oui=1 l__l MERE_MK179

Si oui, résultat du dernier VCT de T4 : l__l , l__l ms MERE_MK180

Date l__l__l/ l__l__l /l__l__l__l__l MERE_MK181 **date calculée (SA+J) MERE_MK181A/MERE_MK181B**

et heure  l__l__l h l__l__l mn MERE_MK184 / 185

**Durée en minutes entre VCT et accouchement T4** l__l__l__l__l **MERE_MK185A**

**ACCOUCHEMENT Préfixé par** MERE_ML

**Accouchement** **à domicile** : non=0, oui=1 l__l MERE_ML1

Pendant le transport : non=0, oui=1 l__l MERE_ML2

**Entrée en salle de travail  :**

- mère admise directement en salle de travail pour accouchement  : non=0, oui=1 l__l MERE_ML3

- mère hospitalisée dans l’établissement d’accouchement avant l’accouchement :

non=0, oui=1 l__l MERE_ML4

Si oui, service d’hospitalisation : 1 = maternité / 2 = réanimation adulte / 3 = autre l__l MERE_ML5

*D_SERVICEHOSP*

Si autre, précisez …………………………………………………………………………………… MERE_ML5A

**Date de l’entrée en salle de travail :** l__l__l/ l__l__l /l__l__l__l__l MERE_ML6

**heure de l’entrée en salle de travail** l__l__l h l__l__l mn MERE_ML9 / 10

**Durée en minutes entre entrée en salle de travail et accouchement** l__l__l__l__l **MERE_ML10A**

**Dilatation du col à l’entrée en salle de travail :**  l__l__lcm MERE_ML11

**Travail spontané =** l__l MERE_ML12

1 : Travail spontané 2 : Déclenchement  3 : Césarienne avant travail  *D_TRAVAILMOD*

Si « déclenchement » :

Technique de départ : l__l MERE_ML13

1 : Ocytocine +/- rupture des membranes

2 : Prostaglandine

3 : Misoprostol *D_TECHDEPART*

4 : Rupture des membranes seule

5 : Autre, précisez ………………………………………………… MERE_ML13A

Si « césarienne avant travail » :

Programmée (au moins 24h avant) : non=0, oui=1 l__l MERE_ML14

Date: l__l__l/ l__l__l /l__l__l__l__l MERE_ML15

et heure  l__l__l h l__l__l mn MERE_ML18 / 19

**Durée en minutes entre césarienne avant travail et accouchement** l__l__l__l__l **MERE_ML19A**

**Tocolyse au moment du début du travail ?** : non=0, oui=1 l__l MERE_ML20

**Date de la rupture des membranes confirmée :** l__l__l/ l__l__l /l__l__l__l__l MERE_ML21

**Date de la rupture des membranes confirmée (SA + J)** l__l__l (SA) **MERE_ML21A**  l__l (J) **MERE_ML21B**

**et heure de la rupture des membranes confirmée** l__l__l h l__l__l mn MERE_ML24 / 25

**Durée en minutes entre rupture confirmée des memb. et accouchement** l__l__l__l__l **MERE_ML25A**

**Rupture des membranes spontanée** : non=0, oui=1 l__l MERE_ML26

**Couleur du liquide amniotique juste avant l’accouchement**: l__l MERE_ML27

1 : clair 3 : sanglant *D_AMNIOCOLOR*

2 : teinté 4 : méconial

5 : purulent

**Si décision d’arrêt de la grossesse, c’est à dire déclenchement ou césarienne avant travail, motifs de décision :**

|  | non=0, oui=1 |
| --- | --- |
| - HTA | MERE_ML28 |
| - Préeclampsie : indication maternelle de l’arrêt de grossesse | MERE_ML29 |
| - Préeclampsie : indication fœtale de l’arrêt de grossesse | MERE_ML30 |
| - Eclampsie | MERE_ML31 |
| - HELLP syndrome | MERE_ML32 |
| - HRP | MERE_ML33 |
| - Infection materno-foetale ou suspicion d’infection materno-foetale | MERE_ML34 |
| - Rupture prématurée des membranes sans signe d’infection | MERE_ML35 |
| - Retard de croissance intra utérin | MERE_ML36 |
| - Diabète | MERE_ML37 |
| - Placenta Prævia hémorragique | MERE_ML38 |
| - Autre pathologie maternelle :  Si oui, préciser en clair :…………… MERE_ML39A…………………… | MERE_ML39 |
| - Malformation fœtale | MERE_ML40 |
| - Anomalies du RCF évoquant une hypoxie fœtale | MERE_ML41 |
| - Anomalie de la vct | MERE_ML42 |
| - Anomalies des dopplers foetaux | MERE_ML43 |
| - Autre pathologie fœtale :  Si oui, préciser en clair : ………………………. MERE_ML44A………… | MERE_ML44 |

## **Pour tous les cas, compte tenu du contexte au moment du travail, caractériser la prématurité** l__l MERE_ML45

1 : Prématurité induite = césarienne avant travail / travail déclenché *D_PREMATURITE*

2 : Prématurité spontanée acceptée = arrêt intentionnel de tocolyse, absence

intentionnelle de prescription de tocolyse, dilatation trop avancée pour tocolyse

3 : Prématurité spontanée non acceptée = échec de tocolyse

**Si travail, complications du travail avant l’accouchement :** non=0, oui=1 l__l MERE_ML46

Si oui, Métrorragies : non=0, oui=1 l__l MERE_ML47

Hyperthermie maternelle ≥ 38° : non=0, oui=1 l__l MERE_ML48

Autre : non=0, oui=1 l__l MERE_ML49

Si autre, préciser en clair : …………………………………………………………. MERE_ML49A

**Traitement(s) administré(s) en salle de travail :**

Nubain : non=0, oui=1 l__l MERE_ML50

Morphiniques (hors péridurale et AG) : non=0, oui=1 l__l MERE_ML51

Ocytociques – Syntocinon : non=0, oui=1 l__l MERE_ML52

Antibiotiques : non=0, oui=1 l__l MERE_ML53

Si oui, type d’antibiotiques *D_ANTI_INFECT* l__l MERE_ML54

Si autre : MERE_ML54A

Indication : l__l MERE_ML55

1 : accouchement prématuré inexpliqué

2 : RPM

3 : antécédent IMF *D_INDICANTIBIO*

4 : PV StreptoB+

5 : PV Ecoli+

6 : autre, préciser en clair : ………………………………………………… MERE_ML55A

Antihypertenseurs : non=0, oui=1 l__l MERE_ML56

Autres, préciser en clair : ………………………………………………………………… ……………… MERE_ML57

**Date à 5 cm de dilatation du col :**  l__l__l/ l__l__l /l__l__l__l__l MERE_ML58

**et heure à 5 cm de dilatation du col** l__l__l h l__l__l mn MERE_ML61 / 62

**Durée en minutes entre dilatation du col à 5cm et accouchement** l__l__l__l__l **MERE_ML62A**

**Anesthésie péridurale :** non=0, oui=1 l__l MERE_ML63

**Rachianesthésie :** non=0, oui=1 l__l MERE_ML64

**Anesthésie Générale (AG) :** non=0, oui=1 l__l MERE_ML65

Si oui, date de début d’AG : l__l__l/ l__l__l /l__l__l__l__l MERE_ML66

et heure de début d’AG l__l__l h l__l__l mn MERE_ML69 / 70

**Durée en minutes entre anesthésie générale et accouchement** l__l__l__l__l **MERE_ML70A**

#### Périnée  Prefixé par MERE_MN

**Etat du périnée après accouchement** : l__l MERE_MN1

1 : Intact

2 : Episiotomie *D_PERINEE*

3 : Déchirure 1^er^ ou 2^ème^ degré

4 : Déchirure 3^ème^ ou 4^ème^ degré (complet)

## Histologie placentaire

***NOTE : les variables .._resume ont été mis à jour après retour au CR d'examen histologique (réalisé par ELSA LORTHE - concerne les enfants du tronc commun mort-nés ou nés vivants).***

**Examen histologique placentaire pratiqué :** non=0, oui=1 l__l MERE_MN2

**mere_mn2_resume**

Si oui,

Résultats de l'examen histologique en attente? non=0, oui=1 l__l MERE_MN2A

**mere_mn2a_resume**

*** Signes de chorioamniotite** : MERE_MN3

**mere_mn3_resume**

1 : Absent

2 : Chorioamniotite sans funiculite *D_CHORIO*

3 : Funiculite

*** Signes/pathologies vasculaires (HRP…) :** non=0, oui=1 MERE_MN4

*** Grossesses multiples** :

Si jumeaux MERE_MN5

**mere_mn5_resume**

1 : bichorial / biamniotique

2 : monochorial / monoamniotique *D_CHORIONICITE*

3 : monochorial / biamniotique

# Si triplés ou quadruplés, description, en clair :………………………………………………… MERE_MN6

……………………………………………………………………………………………………… MERE_MN7

**mere_info_anapath : champs texte avec info des CR**

**ANATOMOPATHOLOGIE PLACENTAIRE (Concerne les enfants inclus du tronc commun, créée par Héloïse Torchin)**

**Chorioamniotite histologique**  **mere_anapath**

| *0 : Examen anatomopathologique du placenta non réalisé* |  |
| --- | --- |
| *1 : Examen anatomopathologique réalisé et absence de chorioamniotite histologique* |  |
| *2 : Chorioamniotite histologique (sans funiculite)* |  |
| *3 : Funiculite* |  |

**SEJOUR DE LA MERE EN MATERNITE Prefixé par** MERE_MQ

Synthèse du séjour dans l’établissement d’accouchement

Avant l’accouchement

**Nombre de jours passés** **en Réanimation**  l__l j MERE_MQ1

Pour quelles complications ? …………………………………………………………… MERE_MQ1A

**Nombre de jours passés en Soins polyvalents continus** l__l j MERE_MQ2

Pour quelles complications ? …………………………………………………………… MERE_MQ2A

**Nombre de jours passés dans un autre service** (hors obstétrique/ réanimation / soins polyvalents

continus) : l__l j MERE_MQ3 Type de service : ……………………………………………… MERE_MQ4

Après l’accouchement

**Nombre de jours passés** **en Réanimation**  l__l j MERE_MQ5

Pour quelle complication ? …………………………………………………………… MERE_MQ5A

**Nombre de jours passés en Soins polyvalents continus** l__l j MERE_MQ6

Pour quelles complications ? …………………………………………………………… MERE_MQ6A

**Nombre de jours passés dans un autre service** (hors obstétrique/ réanimation / soins polyvalents

continus) : l__l j MERE_MQ7 Type de service : ……………………………………… MERE_MQ8

Complications maternelles graves ou pathologies graves des suites de couches :

**Hémorragie du post-partum**: non=0, oui=1 l__l MERE_MQ9

Si oui, transfusion : non=0, oui=1 l__l MERE_MQ10

**Complications de la préeclampsie**: non=0, oui=1 l__l MERE_MQ11

**Insuffisance rénale**: non=0, oui=1 l__l MERE_MQ12

**OAP** : non=0, oui=1 l__l MERE_MQ13

**Complications cardiovasculaires**  non=0, oui=1 l__l MERE_MQ14

**Autres** : non=0, oui=1 l__l MERE_MQ15

Si oui, en clair : …………………………………………………………… MERE_MQ15A

Sortie :

**Date de sortie de la maternité** l__l__l/ l__l__l /l__l__l__l__l MERE_MQ16

**Sortie de maternité de la mère, nombre jours après l'accouchement** l__l__l__l__l **MERE_MQ16A**

**Lieu de sortie :**  l__l MERE_MQ19

1=Domicile

2=Transfert pour complication dans un autre établissement *D_SORTIE*

3=Transfert pour rapprochement mère-enfant

Si « 2 » : motif de transfert : en clair ……………………………… MERE_MQ19A

**Mode de sortie :** 1 = vivante / 2 = décédée *D_SORTIEMOD* MERE_MQ20

Si décédée, **date de décès** : l__l__l/ l__l__l /l__l__l__l__l MERE_MQ21

**Décès de la mère, nombre jours après l'accouchement** l__l__l__l__l **MERE_MQ21A**

**motif du décès** en clair …………………………………………… MERE_MQ24

**CONTEXTE DE NAISSANCE (Concerne les enfants inclus du tronc commun)**

**Contexte de pathologie vasculaire en 4 classes**  l__I **MERE_CONTEXTE_HTA_4_CL**

| *0 : Pas d’HTA* | *D_CONTEXTHTA* |
| --- | --- |
| *1 : HTA permanent en dehors de la grossesse* |  |
| *2 : HTA pendant la grossesse* |  |
| *3 : HTA hors et pendant la grossesse* |  |

**Contexte d’hématome rétro-placentaire(HRP) non=0, oui=1 l__l MERE_CONTEXTE_HRP**

**Contexte hémorragique non=0, oui=1 l__l MERE_CONTEXTE_HEMO**

**Contexte de rupture prématurée des membranes**  **non=0, oui=1 l__l MERE_CONTEXTE_RPM**

**Contexte infectieux en 4 classes l__l MERE_CONTEXTE_INFECT_CHORIO_4CL**

| *0 : Pas de complication infectieuse, ni chorioamniotite* | *D_CONTEXTINF* |
| --- | --- |
| *1 : Chorioamniotite sans autre complication infectieuse* |  |
| *2 : Complication infectieuse sans chorioamniotite* |  |
| *3 : Complication infectieuse + chorioamniotite* |  |

**MAP à membranes intactes   non=0, oui=1 l__l MERE_MAP_SI**

**CAUSE DE PREMATURITE** *(concerne les singletons nés vivants 24-34SA+6J du tronc commun, à exclure enfants avec malformation, grossesses multiples en début de grossesse, pathologies rares ou non classables (107cas) et 6 cas de données manquantes)*

**Cause de prématurité détaillée en 12 classes l__l MERE_CAUSE**

| *0 : Pas de cause* | *D_CAUSEPREMA* |
| --- | --- |
| *1 : W spont.* | *7 : RCIU* |
| *2 : RPM* | *8 : HRP* |
| *3 : Pré-éclampsie* | *9 : P MAT* |
| *4 : Eclampsie* | *10 : METRO* |
| *5 : HTA*  *6 : HELLP* | *11 : P FOET*  *12 : Mixte* |
|  |  |

**Cause de prématurité en 6 classes**   **l__l MERE_SUPERCAUSE**

| *1 : Preterm labor* | *D_SUPERCAUSE* | *Avec :* |
| --- | --- | --- |
| *2 : PROM* | *5 : HRP isolé* | *RPM : de +24h* |
| *3 : Pathologie vasculaire SANS RCIU* | *6 : RCIU isolé* | *RCIU : Suspicion de RCIU anténatal* |
| *4 : Pathologie vasculaire ET RCIU* |  | *HRP isolé : Pas de rupture,* |
|  |  | *pas de MAP de +24h, pas de patho. vasc* |

|  |  |  |
| --- | --- | --- |

**Cause de prématurité grossesse géméllaire:** (Cause de prématurité pour les grossesses gémellaires hors 3 cas de grossesses avec un seul jumeau inclus dans Epipage2, hors accouchement à domicile, hors accouchement avec naissance des jumeaux à deux temps différents et hors cas de grossesses ou au moins un enfant est IMG ou un enfant est MN avant l’admission et avant le début du travail n=1616 (808 grossesses) créée par M.Quere

**Cause de prématurité jumeau** ____________________________________________[__] **Mere_causejumeau**

| *1 :* MAP |
| --- |
| *2 :* RPM |
| *3 :* Plusieurs causes retrouvées (soit MAP-RPM, ou MAP-pathologie vasculaire, ou RPM-pathologie vasculaire) |
| *4 :* Pathologie vasculaire ou HRP isolé |
| 5 : RCIU *isolé* |

| *Enfants tronc commun nés vivants, sans malformation congénitale majeure, AG<32 SA (Variable HTorchin)* | | |
| --- | --- | --- |
| **Pathologies vasculaires gravidiques** | **mere_pathovasc_4cl** | 0 : Absence |
|  |  | 1 : RCIU suspecté pendant la grossesse sans atteinte maternelle  2 : Pathologie vasculaire avec retentissement maternel isolé (HTA gravidique, prééclampsie, eclampsie, HELLP) SANS RCIU |
|  |  |  |
|  |  | 3 : Pathologie vasculaire avec retentissement maternel isolé (HTA gravidique, prééclampsie, eclampsie, HELLP)  + RCIU suspecté pendant la grossesse |

**MATERNITE – PARTIE ENFANT**

**Etablissement d’accouchement**:

Etablissement de naissance : …………………………………………………………………………………………… ENR_ETABNAISS

N° finess : l__l__l__l__|__l__l__l__l__l ENR_FINESSNAISS

Type : 1 : I  / 21 : IIa   / 22 : IIb / 3 : III  *D_ETABNIV*  l__l ENR_NIVEAU2NAISS

Etablissement de naissance anonymisé : l__l__l ENR_NUMETABNAISS

**Date de naissance corrigée (JJ/MM/AAA)** l__l__l/ l__l__l /l__l__l__l__l  **COR_DDN_ENFANT**

**et heure de naissance corrigée (hh:mn)** l__l__l h l__l__l mn  **COR_HEURE_NAISS COR_MIN_NAISS**

**Age gestationnel à la naissance corrigé** l__l l__l SA + l__l j **COR_AGN / COR_AGN_J**

**Sexe de l’enfant corrigé : garçon =1 / fille=2 / indéterminé = 3** D_SEXE l__l **COR_SEXE_ENFANT**

**Statut vital de l'enfant corrigée:**  l__I COR_STATUTNAIS

| *1 : IMG* | *D_STATNAIS* |
| --- | --- |
| *2 : Mort-né* |  |
| *3 : Décédé en salle de naissance* |  |
| *4 : Décédé en neonat* |  |
| *5 : Sortis vivant neonat* |  |
| *9 : Transféré en neonat, statut sortie neonat incomplet* |  |

**Partie à compléter dans le cas d’une IMG Préfixé par :** ENFANT_MG

**Foeticide :** non=0, oui=1 _ l__l ENFANT_MG11

**Mode d’accouchement :** Voie basse = 1 / Césarienne = 2 *D_ACCVOIE* l__l ENFANT_MG12

**NAISSANCE cas Mort-né et né vivant Préfixé par :** ENFANT_MM

####

#### Naissance de l^’^enfant :

**Date de naissance corrigée (JJ/MM/AAA)** l__l__l/ l__l__l /l__l__l__l__l  **COR_DDN_ENFANT**

**et heure de naissance corrigée (hh:mn)** l__l__l h l__l__l mn  **COR_HEUR_NAISS COR_MIN_NAISS**

**Age gestationnel à la naissance corrigé** l__l l__l SA + l__l j **COR_AGN / COR_AGN_J**

**Sexe de l’enfant corrigé : garçon =1 / fille=2 / indéterminé = 3** D_SEXE l__l **COR_SEXE_ENFANT**

**Statut vital de l'enfant corrigée:**  l__I COR_STATUTNAIS

| *1 : IMG* | *D_STATNAIS* |
| --- | --- |
| *2 : Mort-né* |  |
| *3 : Décédé en salle de naissance* |  |
| *4 : Décédé en neonat* |  |
| *5 : Sortis vivant neonat* |  |
| *9 : Transféré en neonat, statut sortie neonat incomplet* |  |

## **Présentation diagnostiquée avant l’accouchement et d’éventuelles manœuvres** :

## céphalique=1 / siège =2 / autre=3 D_PRESENTATION l__I ENFANT_MM10

**Mode d’accouchement :** l__I ENFANT_MM11

Voie basse non instrumentale=1 / Voie basse instrumentale=2 / Césarienne=3 *D_ACCVOIE1F*

Si voie basse,

**Date à dilatation complète :** l__l__l/ l__l__l /l__l__l__l__l ENFANT_MM12

**Heure** **à dilatation complète** l__l__l h l__l__l mn ENFANT_MM15/16

**Durée en minutes entre dates dilatation complète et accouchement** l__l__l__l__l **ENFANT_MM16A**

**Durée des efforts expulsifs :** l__l__l mn ENFANT_MM17

**Extraction instrumentale** : non=0, oui=1 l **__**l ENFANT_MM18

Si oui, Ventouse : non=0, oui=1 l__I ENFANT_MM19

Forceps sur tête dernière : non=0, oui=1 l__I ENFANT_MM20

Autre indication de forceps : non=0, oui=1 l**__**l ENFANT_MM21

Spatules : non=0, oui=1 l**__**l ENFANT_MM22

Si voie basse et présentation du siège :

**Rétention de tête dernière** : non=0, oui=1 l**__**l ENFANT_MM23

**Rétraction du col** : non=0, oui=1 l**__**l ENFANT_MM24

**Incision du col** : non=0, oui=1 l**__**l ENFANT_MM25

**Manœuvres**: non=0, oui=1 l**__**l ENFANT_MM26

Si oui, manœuvre réalisée l**__**l ENFANT_MM27

1 : Manœuvre d’accompagnement sans dystocie

2 : Manœuvre pour rétention tête dernière *D_MANŒUVRE*

3 : Manœuvre pour autre dystocie

#### Si voie basse du 2^ème^ enfant :

**Type d’extraction** l**__**l ENFANT_MM37

1 : Sans manœuvre

2 : Extraction instrumentale *D_EXTRACTTYPE*

3 : PE/GE (petite extraction / grande extraction)

4 : VMI +GE (version par manœuvre interne + grande extraction)

**Aisance de l’extraction** :

Rétraction du col : non=0, oui=1 l**__**l ENFANT_MM38

Incision du col : non=0, oui=1 l**__**l ENFANT_MM39

Forceps pour rétention de tête dernière : non=0, oui=1 l**__**l ENFANT_MM40

Autres manœuvres pour rétention de tête dernière : non=0, oui=1 l**__**l ENFANT_MM41

Si oui, préciser en clair ENFANT_MM41A

Si césarienne,

**Indication de la césarienne :** 2 réponses possibles l__I ENFANT_MM28

I__I ENFANT_MM29

1 : Systématique pour terme ou présentation fœtale ou grossesse multiple *D_INDICCESA*

2 : Anomalies du RCF pendant le travail

3 : Stagnation de la dilatation

4 : Non engagement et décision d’absentions de manœuvre

5 : Autre pathologie fœtale (rciu, etc)

6 : Autre pathologie maternelle (préeclampsie, etc)

7 : Autre, précisez ENFANT_MM28A

#### Hystérotomie :

Incision transversale : non=0, oui=1 I__I ENFANT_MM30

Incision verticale : non=0, oui=1 I__I ENFANT_MM31

Segmentaire : Incision verticale : non=0, oui=1 I__I ENFANT_MM32

Corporéale : non=0, oui=1 I__I ENFANT_MM33

Refend (accidentel ou volontaire) de l’incision : non=0, oui=1 I__I ENFANT_MM34

#### Aisance de l’extraction : simple=1 / difficile=2 I__I ENFANT_MM35

*D_EXTRACT*

**Si césarienne pour le 2^ème^ enfant**

**Indication de la césarienne sur J2** : I__I ENFANT_MM42

1 : Echec extraction de J2 *D_INDICCESA2F*

2 : Non engagement et décision d’abstention de manœuvre

**Clampage tardif du cordon :** non=0, oui=1 l__l ENFANT_MM36

**Partie à compléter dans le cas d’un (ou plusieurs) enfants mort-nés ou si IMG Préfixé par** ENFANT_MO

**Date du décès** **de l’enfant** l__l__l/ l__l__l /l__l__l__l__l ENFANT_MO5

**date calculée (SA+J) ENFANT_MO5A/ENFANT_MO5B**

Si date inconnue, estimation du terme au décès l__l__l SA ENFANT_MO6

**Poids mesuré en salle de naissance corrigée** l__l__l__l__l g COR_PDS_NCE

**Causes du décès** (enchaînement des causes suivant le certificat de décès) :

*Causes d’origine fœtale ou néonatale*

**Cause d’origine fœtale ou néonatale ayant directement provoqué le décès**

**En clair**: ENFANT_MO8

**Due à :**  ENFANT_MO9

**Autre(s) cause(s) associée(s)** : non=0, oui=1 l__l ENFANT_MO10

Si oui, préciser en clair : ENFANT_MO11

***Codage CIM10 causes du décès* *des variables ENFANT_MO8 - ENFANT_MO9 - ENFANT_MO11 (codage concerne les enfants mort-nés et IMG)***

***|__|__|__|__|__| ENFANT_CIM10_MALF1***

***|__|__|__|__|__| ENFANT_CIM10_MALF2***

***|__|__|__|__|__| ENFANT_CIM10_MALF3***

***|__|__|__|__|__| ENFANT_CIM10_MALF4***

**Synthèse Malformation pas de malformation=0, au moins 1 malformation majeure=1, uniquement une malformation mineure=2, doute sur une malformation ou sur critère de gravité=9 l__I ENFANT_MALFO_MAJEUR**

*(Concerne les enfants inclus du tronc commun- Variable N.Lelong, registre des malformations congénitales*

***Décès au moment de l’admission (concerne les enfants mort-nés) ___________[] enfant_mfiuadmission***

créée par M.Quere:

0 : DCD à l’admission *d_mfiu*

1 : Vivant à l’admission

9 : NON APPLICABLE ( i.e enfant dont le statut de naissance n’est pas mort-né  ; grossesse triple ou + ; acc à domicile)

**Décès en début de travail  *(concerne les enfants mort-nés)* ____________________[] *enfant*_mfiutravail**

créée par M.Quere

0 : DCD en début de travail *d_mfiub*

1 : Vivant en début de travail

9 : NON APPLICABLE ( i.e enfant dont le statut de naissance n’est pas mort-né  ; grossesse triple ou + ; acc à domicile ; DCD à l’admission ; pour certains AG<24 lorsque l’algorithme ne permettait pas de reclasser les enfants)

*Causes d’origine obstétricale ou maternelle*

**Cause d’origine obstétricale ou maternelle déterminante de la mort**

**En clair**: ENFANT_MO12

**Autre(s) cause(s) associée(s)** : non=0, oui=1 l__l ENFANT_MO13

Si oui, préciser en clair : ENFANT_MO14

**Une autopsie a-t-elle été (ou va-t-elle être) pratiquée** ? l__l ENFANT_MO15

0 : non

1 : oui, résultat non disponible *D_AUTOPSIE*

2 : oui, résultat disponible

Si résultat disponible : l__l ENFANT_MO16

1 : L’autopsie établit la cause du décès

2 : L’autopsie confirme la cause suspectée du décès *D_AUTOPSIERES*

# 3 : L’autopsie est non-informative

**PRISE EN CHARGE DU NOUVEAU-NE EN SALLE DE NAISSANCE Préfixé par :** ENFANT_MP

**Poids mesuré en salle de naissance corrigée** l__l__l__l__l g COR_PDS_NCE

| **Poids de naissance courbes EPOPE (z-score)** | **neo_PNzscore_epope** | variable continue |
| --- | --- | --- |
|  |  |  |
| **Poids de naissance courbes EPOPE (percentiles)** | **neo_PNperc_epope** | variable continue |
|  |  |  |
|  | **neo_PN10p_epope** | 0 : poids de naissance <10e percentile selon courbes EPOPé |
|  | **D_10pn** | 1 : poids de naissance ≥10e percentile selon courbes EPOPé |
|  |  |  |
|  | **neo_PN3p_epope** | 0 : poids de naissance <3e percentile selon courbes EPOPé |
|  | **D_3pn** | 1 : poids de naissance ≥3e percentile selon courbes EPOPé |

***REF courbe EPOPé :*** *1 Ego A, Prunet C, Lebreton E et al. Customized and non-customized French intrauterine growth curves. I – Methodology J Gynecol Obstet Biol Reprod 2016 ;45:155-64*

**Anomalies congénitales** : non=0, oui=1 l__l ENFANT_MP4

Si oui, description finale des anomalies congénitales, y compris les syndromes (en précisant pour chacune d’elle la description la plus détaillée possible en clair) :

1^er^ malformation : *en clair + CIM10* ENFANT_MP5

2^ème^ malformation : *en clair + CIM10* ENFANT_MP6

3^ème^  malformation : *en clair + CIM10* ENFANT_MP7

**Score d’Apgar à 1 min** l__l__l ENFANT_MP8

**Score d’Apgar à 5 min**  l__l__l ENFANT_MP9

**Score d’Apgar à 10 min** l__l__l ENFANT_MP10

**Manœuvres de réanimation à la naissance** : non=0, oui=1 l__l ENFANT_MP11

Si non : pourquoi l__l ENFANT_MP12

1 : Car inutile (l’enfant va bien)

2 : Car décision anténatale d’absence de réanimation  *D_REANIMAT*

3 : Autre

**Pour cet enfant, quel est le protocole théorique de prise en charge respiratoire à la naissance ?**  l__l ENFANT_MP13

1 : Aucun *D_RESPIRATOIRE*

2 : CPAP

3 : Surfactant prophylactique

4 : INSURE

**Oxygénation** : non=0, oui=1 l__l ENFANT_MP14

**Application de Pressions Positives** : non=0, oui=1 l__l ENFANT_MP15

**Intubation trachéale** : non=0, oui=1 l__l ENFANT_MP16

Si oui,

Age (en min) l__l__l min ENFANT_MP17

Médicament(s) administré(s) avant l’intubation : non=0, oui=1 l__l ENFANT_MP18

Si oui,

Atropine : non=0, oui=1 l__l ENFANT_MP19

Fentanyl : non=0, oui=1 l__l ENFANT_MP20

Sufentanil : non=0, oui=1 l__l ENFANT_MP21

Hypnovel (midazolam) ® : non=0, oui=1 l__l ENFANT_MP22

Ketamine : non=0, oui=1 l__l ENFANT_MP23

Diprivan (propofol) ® : non=0, oui=1 l__l ENFANT_MP24

Autre non=0, oui=1 l__l ENFANT_MP25

Si autre, précisez en clair : ENFANT_MP25A

**Massage cardiaque :** non=0, oui=1 l__l ENFANT_MP26

**Adrénaline :** non=0, oui=1 l__l ENFANT_MP27

**Remplissage (au moins 10 ml/kg) :** non=0, oui=1 l__l ENFANT_MP28

**Gaz du sang au cordon :** non=0, oui=1 l__l ENFANT_MP29

Si oui, artériel : non=0, oui=1 l__l ENFANT_MP30

Si oui, pH : l__l, l__l l__| ENFANT_MP31

Base deficit : l__l, l__l mmol/l ENFANT_MP32

Lactates : l__l l__l mmol/l ENFANT_MP33

**Une limitation de traitements actifs ou d’abstention thérapeutique est-elle survenue en salle de naissance ?** non=0, oui=1 l__l ENFANT_MP34

Si oui :

Cette décision avait-elle été prise en anténatal ? non=0, oui=1 l__l ENFANT_MP35

Le contenu de la discussion obstétrico-pédiatrique en salle de naissance a-t-il été transcrit dans le dossier de

la mère ou de l’enfant ? non=0, oui=1 l__l ENFANT_MP36

Information des parents et recueil de leur avis : le contenu de cet entretien a-t-il été transcrit dans le dossier

de la mère ? non=0, oui=1 l__l ENFANT_MP37

**L’enfant est-il décédé en salle de naissance** : non=0, oui=1 l__l ENFANT_MP38

Si non,

**Lieu d’hospitalisation de l’enfant :**  l__l ENFANT_MP39

1 : Même établissement que le lieu dans lequel l’enfant est né

2 : Autre établissement que le lieu dans lequel l’enfant est né *D_HOSPLIEU*

**Unité d’hospitalisation :**  l__l ENFANT_MP40

1 : réanimation néonatale

2 : soins intensifs

3 : médecine néonatale

4 : unité kangourou

5 : réanimation pédiatrique polyvalente *D_HOSPUNIT*

6 : chirurgie pédiatrique

7 : autre, préciser ENFANT_MP40A

Préciser en clair le nom et le lieu d’hospitalisation : Nom………………………………………...….. ENFANT_MP41

Lieu ……………………………………………… ENFANT_MP42

Si oui,

**Date du décès** **de l’enfant** l__l__l/ l__l__l /l__l__l__l__l ENFANT_MP43

**Heure et minute du décès** **de l’enfant** l__l__l h l__l__l mn ENFANT_MP46 / 47

**Durée de vie de l’enfant en minutes** l__l__l__l__I **ENFANT_MP47A**

**Causes du décès** (enchaînement des causes suivant le certificat de décès)

*Causes d’origine fœtale ou néonatale*

**Cause d’origine fœtale ou néonatale ayant directement provoqué le décès**

**En clair**: ENFANT_MP48

**Due à :**  ENFANT_MP49

**Autre(s) cause(s) associée(s)** : non=0, oui=1 l__l ENFANT_MP50

Si oui, préciser en clair : ENFANT_MP50A

*Causes d’origine obstétricale ou maternelle*

**Cause d’origine obstétricale ou maternelle déterminante de la mort**

**En clair**: ENFANT_MP51

**Autre(s) cause(s) associée(s)** : non=0, oui=1 l__l ENFANT_MP52

Si oui, préciser en clair : ENFANT_MP52A

**Une autopsie a-t-elle été (ou va-t-elle être) pratiquée** ? l__l ENFANT_MP53

0 : non

1 : oui, résultat non disponible *D_AUTOPSIE*

2 : oui, résultat disponible

Si résultat disponible : l__l ENFANT_MP54

1 : L’autopsie établit la cause du décès

2 : L’autopsie confirme la cause suspectée du décès *D_AUTOPSIERES*

# 3 : L’autopsie est non-informative

#### En cas de décès en salle de naissance suite à une décision de limitation / d’abstention thérapeutique

**Durée de vie de l’enfant :** l__l__l h l__l__l mn ENFANT_MP55 / 56

**Autres informations relatives aux circonstances du décès :** ENFANT_MP57

**Médicaments administrés dans l’intention d’assurer le confort de l’enfant :** non=0, oui=1l__lENFANT_MP58

Si oui, lesquels ?

Benzodiazépines : non=0, oui=1 l__l ENFANT_MP59

Phénobarbital : non=0, oui=1 l__l ENFANT_MP60

Morphiniques : non=0, oui=1 l__l ENFANT_MP61

Thiopenthal : non=0, oui=1 l__l ENFANT_MP62

Curare : non=0, oui=1 l__l ENFANT_MP63

Autres : non=0, oui=1 l__l ENFANT_MP64

Les doses ont été proportionnées aux besoins de sédation/analgésie : non=0, oui=1 l__l ENFANT_MP65

A-t-il été nécessaire d’intensifier le traitement ? non=0, oui=1 l__l ENFANT_MP66

**Rapport écrit dans le dossier sur le temps de vie et les moyens mis en œuvre** :

non=0, oui=1 l__l ENFANT_MP67

**MALFORMATIONS FŒTALES**

**Cause d’IMG l__l ENFANT_MALFOFOETALE**

*(Concerne tous les enfants du tronc commun : inclus ou refus)*

1 : Malformation *D_MALFOFOETALE*

0 : Autre cause

**Malformation pouvant avoir un impact sur la survie non=0, oui=1 l__I ENFANT_MALFOFOETALE2**

*(Concerne les enfants inclus du tronc commun – Variable P. Delorme)*

**Synthèse Malformation pas de malformation=0, au moins 1 malformation majeure=1, uniquement une malformation mineure=2, doute sur une malformation ou sur critère de gravité=9 l__I ENFANT_MALFO_MAJEUR**

**D_malfo**

*(Concerne les enfants inclus du tronc commun- Variable N.Lelong, registre des malformations congénitales*
