# Supplementary material for: Premedication practices for delivery room intubations in premature infants in France: Results from the EPIPAGE 2 cohort study
Source: PLoS One. 2019 Apr 10;14(4):e0215150. doi: 10.1371/journal.pone.0215150 (PMC6457540; doi:10.1371/journal.pone.0215150)
Supplement: S2 File — Item completed during a face to face interview with the mother to complete socio-economic data and pregnancy-birth informations. (DOCX) [file pone.0215150.s002.docx]

**
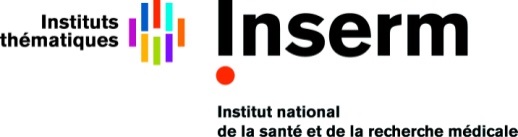
**

**Epidemiological study on low gestational age infants**

**ANNOTATED**

**MATERNAL INTERVIEW**

| HIGHLIGHTED VARIABLES | **Variable IDENTITY** |
| --- | --- |
| HIGHLIGHTED VARIABLES IN RED | **Indirectly identifying variable – Conditionally transferred** |
| VARIABLES IN RED | **Variable name** |
| **VARIABLES IN GREEN** | **Calculated variables** |
| **Variables in brown** | **Synthetic variables** |
| *RESPONSE OPTIONS* | **Dictionary name** |
| Boxes | **Conditional block** |

**Date of delivery:** __ _ **|__|__| / |__|__| / |__|__|__|__|** EM_ENTMATACC

This interview is to be carried out with all mothers whose infant was discharged alive from the first neonatal hospitalization department (where the infant’s stay was at least 72h), or at discharge from the maternity department if the infant was not transferred elsewhere. It relates to social, demographic and economic characteristics and to management and care received.

**NB:** certain missing social data can be recovered from the self-administered questionnaire

***Begin the interview by asking for news of the infant/s before moving on to the set questions.***

***« This interview is part of the Epipage2 study. It aims to describe your child’s environment. Together, we’ll look at some general questions, questions about your health before and during the pregnancy, and we’ll finish with questions about the birth and your baby »***

**Date of the interview (dd/mm/yyyy) |__|__| / |__|__| / |__|__|__|__|**EM_ENTMAT1

**Age of the infant at interview, in days** l__l__l__l__l **EM_ENTMAT1C**

***« Let’s start by talking about your circumstances, your daily life… »***

**What is your date of birth? (dd/mm/yyyy) ___ |__|__| / |__|__| / |__|__|__|__|** EM_ENTMAT4

**Age of the mother at delivery, in completed years ___** l__l __l COR_AGE_MERE

**What is your department of residence? |__|__|__|**EM_ENTMAT7

**What is your town of residence, in full:** EM_ENTMAT8

**postcode: |__|__|__|__|__|** EM_ENTMAT9

**What is your level of education? |__|**EM_ENTMAT10

0: no schooling

1: primary schooling *D_ETUDE*

2: junior secondary school (up to age 15)

3: short vocational training leading to a diploma

4: higher secondary school, general education (including general high school diploma)

5: higher secondary school, technological education (including technological high school diploma)

6: higher secondary school, vocational education (including vocational high school diploma)

7: high school diploma level + 1 or 2 years (including university diploma in technology, advanced vocational diploma)

8: high school diploma level + 3 or 4 years (including bachelor’s degree, master’s degree)

9: high school diploma level + 5 years or more (including specialized master’s degree, engineering diploma)

**Date of the maternal interview completed at 1 year |__| COR_ETUDE_MERE_CL**

1: < Higher secondary school

2: Higher secondary school

3: High school diploma +1 +2

4: > High school diploma +3

**What was your employment status during your pregnancy? |__|** EM_ENTMAT11

1: in employment

2: apprentice under contract or paid internship *D_SITUATION*

3: student, pupil, in training or internship

4: unemployed or job seeker

5: homemaker

6: child-care leave

7: other, please specify: EM_ENTMAT12

**If you were employed during your pregnancy, what type of employment contract did you have? |__|**EM_ENTMAT13 1: self-employed, employer

2: permanent contract or public sector employee *D_CONTRAT*

3: fixed-term contract

4: other types of temporary employment, please specify: EM_ENTMAT14 5: other, please specify: EM_ENTMAT15

**If you were employed during your pregnancy, at what date did you finally stop work before having your baby? (dd/mm/yyyy) |__|__| / |__|__|/ |__|__|__|__|**EM_ENTMAT16

**Calculated gestational age (weeks’gestation) when the mother stopped working :**

**|__|__| EM_ENTMAT16A / |__|__|EM_ENTMAT16B**

**If you were not in employment during your pregnancy, did you previously have a professional activity?** (0: no  1: yes **) |__|**EM_ENTMAT19

**What is your current profession or your last profession?**

Please specify your profession in detail: EM_ENTMAT20

Code: **|__|**EM_ENTMAT21

1: farmer

2: crafts and related trades, shopkeeper, entrepreneur *D_PROFESSION*

3: managerial (liberal profession, teacher, engineer...)

4: intermediate profession (primary school teacher, nurse, technician, supervisor...)

5: public sector worker or company administrative worker

6: shop worker

7: private domestic employee

8: skilled worker

9: unskilled worker

0: none

**Socioprofessional category of the household (or the mother if living alone) COR_CSPMENAGE**

*Data from the medical records completed by the maternal interview at one year*

| 1: Managerial |
| --- |
| 2: Intermediate |
| 3: Administrator, company director, civil servant, student |
| 4: Domestic or sales employee |
| 5: Worker |
| 6: None |

**What is your country of birth?**

In full: EM_ENTMAT22

*D_PAYS*

Code **|__|**EM_ENTMAT23

1: France (metropolitan or overseas) *D_PAYS2F*

2: other European country INSEE country code **|__|__|__|** EM_ENTMAT22B

3: North African country

4: other African country

5: other country

Data from maternity records completed by data from the maternal interview at 1 year : **|__|** COR_PAYS_NAISS

1: France (metropolitan or overseas) *D_PAYS2F*

2: other European country

3: North African country

4: other African country

5: other country

If you were born in France: in which department or overseas territor? **|__|__|__|**EM_ENTMAT24

If you were born abroad: in what year did you arrive in France? (yyyy) **|__|__|__|__|**EM_ENTMAT25

**What is your nationality? |__|**EM_ENTMAT26

1: French *D_NATIONALITE*

2: foreign or stateless

If foreign: what nationality? in full: EM_ENTMAT27

INSEE country code **|__|__|__|**EM_ENTMAT27B

**What is your mother tongue (the language your parents spoke when you were a child/a little girl)**

**|__|**EM_ENTMAT28

1: French only *D_LANGUE*

2: another language only

3: French as well as another language

If other language/s, which? (note up to 2) EM_ENTMAT29

EM_ENTMAT29B

**What is your mother’s country of birth?**

In full : EM_ENTMAT30

*D_PAYS*

INSEE country code : **|__|__|__|** EM_ENTMAT30B

Code **|__|**EM_ENTMAT31

1: France (metropolitan or overseas) *D_PAYS2F*

2: other European country

3: North African country

4: other African country

5: other country

**What languages do you usually speak at home? |__|**EM_ENTMAT32

1: French only

2: another language only *D_LANGUE*

3: French as well as another language

If other language/s, which? (note up to 2) EM_ENTMAT33

EM_ENTMAT33B

**Do you live with a spouse or partner? (**0: no  1: yes ) **|__|** EM_ENTMAT34

If yes, do you live in the same household? **|__|**EM_ENTMAT35

1: no, for reasons of distance from work or study

2: no, other reason *D_COUPLE*

3: yes

**What is your marital status? |__|**EM_ENTMAT36

1: single

2: civil partnership *D_MATRIMONIAL*

3: married

4: other

**How many children (aged under 18) live in your household (including those who board elsewhere or who are in shared custody)? |__|__|**EM_ENTMAT37

**In which country was your spouse or partner born?**

In full: EM_ENTMAT38

*D_PAYS*

INSEE country code **|__|__|__|** EM_ENTMAT38B

Code **|__|**EM_ENTMAT39

1: France (metropolitan or overseas)

2: other European country

3: North African country *D_PAYS2F*

4: other African country

5: other country

**What is his nationality? |__|**EM_ENTMAT40

1: French

2: foreign or stateless *D_NATIONALITE*

If foreign: what nationality?

in full : EM_ENTMAT41

INSEE country code : EM_ENTMAT41B

**What is his employment status? |__|**EM_ENTMAT42

1: in employment

2: apprentice under contract or paid internship *D_SITUATION*

3: student, pupil, in training or internship

4: unemployed or job seeker

5: homemaker

6: child-care leave

7: other, please specify : EM_ENTMAT43

If he is not employed, has he worked previously? (0: no; 1: yes) : **|__|** EM_ENTMAT44

**What is his current profession or last profession?**

Please specify his profession in detail : EM_ENTMAT45

Code : **|__|** EM_ENTMAT46

1: farmer

2: crafts and related trades, shopkeeper, entrepreneur *D_PROFESSION*

3: managerial (liberal profession, teacher, engineer...)

4: intermediate profession (primary school teacher, nurse, technician, supervisor...)

5: public sector worker or company administrative worker

6: shop worker

7: private domestic employee

8: skilled worker

9: unskilled worker

0: none

**Where do you live? |__|** EM_ENTMAT47

1: in personal accommodation (rented, bought or loaned)

2: with family/relatives *D_LOGEMENT*

3: with friends

4: in a rented hotel room

5: in emergency or temporary accommodation (hostel, night shelter, mother-and-child center, hotel paid for by an association)

6: in a caravan or mobile home

7: in a place not intended for habitation (street, car, building entrance, makeshift shelter)

**How many people (adults and children) habitually occupy this accommodation? (not including your baby) |__|__|**EM_ENTMAT48

***For the answers « personal accommodation», « with family/relatives », « with friends »:***

**In your accommodation, how many rooms are there, not counting the kitchen, the bathroom or the toilet? |__|__|** EM_ENTMAT49

**Is there an inside bathroom in your accommodation, with a bath or shower? (**0: no**;** 1: yes) : **|__|** EM_ENTMAT50

**Is there an inside toilet in your accommodation? (**0: no; 1: yes ) **|__|**EM_ENTMAT51

**Taking into account all sources of income (wages, pensions, benefits, allowances or other income), what is your and your spouse or partner’s usual monthly income? |__|**EM_ENTMAT52

1: less than 500 € a month *D_ARGENT*

2: less than 1,000 €

3: less than 1,500 €

4: less than 2,000 €

5: less than 3,000 €

6: less than 4,000 €

7: 4,000 € or more

8: prefer not to answer

9: don’t know

**During your pregnancy, what were you and your spouse or partner’s sources of income?**

Income from work, whether salaried or not: no = 0; yes = 1 **|__|**EM_ENTMAT53

Unemployment benefits: no = 0; yes = 1 **|__|**EM_ENTMAT54

Earned income supplement: no = 0; yes = 1 **|__|**EM_ENTMAT55

Sickness, disability or invalidity benefit: no = 0; yes = 1 **|__|**EM_ENTMAT56

Other benefits no = 0; yes = 1 **|__|**EM_ENTMAT57

**At the present time, would you say that financially: |__|**EM_ENTMAT58

1: You are comfortable

2: You get by *D_FINANCIERE*

3: There’s nothing to spare, you have to be careful

4: It’s difficult or impossible to make ends meet

**Do you find it difficult financially to feed your family, to buy clothes or to pay the rent and utility bills (electricity, gas, heating)?** 0: no; 1: yes**|__|** EM_ENTMAT59

**During your pregnancy, did you have to give up medical tests or dental care for financial reasons?** 0: no; 1: yes **|__|** EM_ENTMAT60

**If you have problems, are there any people among your family and friends who you can rely on for material help (financial help, help in the home…)?** 0: no; 1: yes **|__|**EM_ENTMAT61

**If you have problems, are there any people among your family and friends who you can rely on for accommodation for a few days if you need it?** 0: no; 1: yes **|__|**EM_ENTMAT62

**What type of social security do you have? |__|**EM_ENTMAT63

1: Universal health coverage (CMU) with supplementary coverage

2: Universal health coverage (CMU) without supplementary coverage *D_SECU*

3: State medical assistance (AME) or emergency care coverage

4: National health insurance or other social security, with top-up insurance;

5: National health insurance or other social security, without top-up insurance

6: None

***« I’m now going to ask you some questions about your health before and during your pregnancy »:***

**What was your weight at birth?|__|__|__|__|**g EM_ENTMAT64

**Were you born one month or more before term?** 0: no; 1: yes; 2: don’t know **|__|** EM_ENTMAT65

*D_YORN2F*

**What is your height? |__|__|__|** cm EM_ENTMAT66

**What was your weight before pregnancy? |__|__|__|** kgEM_ENTMAT67

**What is the height of the baby’s father? |__|__|__|** cm EM_ENTMAT68

**What is the weight of the baby’s father? |__|__|__|** kg EM_ENTMAT69

**Did you smoke just before your pregnancy?** 0: no; 1: yes **|__|**EM_ENTMAT70

If yes, how many cigarettes did you smoke on average per day? **|__|__|**EM_ENTMAT71

**Does your spouse/partner smoke?** 0: no; 1 : yes **|__|**EM_ENTMAT72

**Did you smoke during the second half of your pregnancy? *(question to be asked all women, including those who did not smoke just before pregnancy)*** 0: no; 1: yes **|__|**EM_ENTMAT73

If yes, how many cigarettes did you smoke on average per day? **|__|__|**EM_ENTMAT74

**Did you take folic acid supplements (including multivitamins) around the time of conception (= during the month before pregnancy and/or during the 1^st^ month of pregnancy):** 0: no; 1: yes **|__|**EM_ENTMAT75

**In order to become pregnant, did you have treatment for sterility, including medical treatment?** 0: no; 1: yes

**|__|**EM_ENTMAT76

If yes, what treatment? **|__|**EM_ENTMAT77

1: IVF with ICSI

2: IVF without ICSI *D_INFERTILITE*

3: artificial insemination

4: hormonal stimulation only

Did you use sperm donation?  0: no; 1: yes **|__|**EM_ENTMAT78

Did you use oocyte donation? 0: no; 1: yes **|__|**EM_ENTMAT79

Did you use embryo donation?  0: no; 1: yes **|__|**EM_ENTMAT80

Did you receive infertility treatment in France? 0: no; 1: yes **| __|**EM_ENTMAT81

If no, please specify the country: EM_ENTMAT82

*D_PAYS*

INSEE country code **|__|__|__|** EM_ENTMAT82B

**Had you already been pregnant before this pregnancy:** 0: no; 1: yes **|__|**EM_ENTMAT83

If yes,

**During the previous pregnancies, did you have one or several voluntary terminations, whether by surgical procedure (aspiration, curettage), or by medication only (RU, Mifegyne)?** 0: no; 1: yes **|__|**EM_ENTMAT84

If yes,

How many voluntary terminations have you had in all? **|__|__|**EM_ENTMAT85

How many voluntary terminations by medication only? **|__|__|** EM_ENTMAT86

***« Now we’re going to talk about the care you received during your pregnancy »:***

**When did you declare the pregnancy (to the Health Insurance)? |__|**EM_ENTMAT87

1: 1^st^ trimester

2: 2^nd^ trimester *D_DECGROSS*

3: 3^rd^ trimester

4: not declared

**Who was the healthcare professional who made the declaration? |__|**EM_ENTMAT88

1: general practitioner

2: ob/gyn in private practice *D_DECPROF*

3: ob/gyn in maternity department

4: midwife in private practice

5: other: please specify : EM_ENTMAT89

**Where did you initially plan to give birth? |__|**EM_ENTMAT90

1: in the maternity department where you did in fact give birth

2: in a different maternity department from the one where you gave birth *D_ACCLIEU*

3: other: please specify : EM_ENTMAT91

**Regarding the maternity department you initially chose for your delivery, what were your reasons for the choice?**

You had had complications during a previous pregnancy or a health problem at the beginning of your pregnancy and this maternity department was the best choice for you 0: no; 1: yes **|__|** EM_ENTMAT92

You had already given birth in this maternity department 0: no; 1: yes **|__|** EM_ENTMAT93

It is close to your home 0: no; 1: yes **|__|** EM_ENTMAT94

The ob/gyn or midwife chosen for the follow-up and/or delivery work in this department 0: no; 1: yes **|__|** EM_ENTMAT95

This maternity department was recommended by your doctor or midwife0: no; 1: yes **|__|** EM_ENTMAT96

Other: please specify : EM_ENTMAT97

**If you delivered in a different maternity department from the one initially planned:**

***If not, continue to « Questions for all women » page 9***

**At which gestational age was the choice of maternity department changed?** **|__|__|** EM_ENTMAT98

**What were the reasons?**

You had a complication during the pregnancy and this maternity department was the best choice for you. 0: no; 1: yes **|__|** EM_ENTMAT99

You gave birth unexpectedly when away from home : 0: no; 1: yes **|__|**EM_ENTMAT100

You chose to change to this maternity department : 0: no; 1: yes **|__|**EM_ENTMAT101

It was close to your home : 0: no; 1: yes **|__|**EM_ENTMAT102

The ob/gyn or the midwife chosen for the delivery work in this department : 0: no; 1: yes **|__|**EM_ENTMAT103

It was recommended by your doctor or midwife : 0: no; 1: yes **|__|**EM_ENTMAT104

Other: please specify : EM_ENTMAT105

**Questions for all women:**

**During the pregnancy, did you have at least one consultation or test:**

**- in a different maternity department to the one where you were followed:** (do not include here in utero transfers or changes of maternity department : 0: no; 1: yes **|__|**EM_ENTMAT106

**- or with a specialist** other than your own ob/gyn (except for routine consultation with an anesthetist before delivery): 0: no; 1: yes **|__|**EM_ENTMAT107

If yes, what specialist or specialists? EM_ENTMAT108

what were the reason/s*?* in full EM_ENTMAT109

**Were you admitted to hospital during this pregnancy, apart from hospitalization for the birth?** 0: no; 1: yes **|__|**EM_ENTMAT110

If yes, where? **|__|**EM_ENTMAT111

1: this maternity department *D_HOSPIGROSS*

2: another maternity department

3: other department, in full: EM_ENTMAT112

**How did you travel to the maternity department for the delivery, or for the hospitalization during which you gave birth? |__|** EM_ENTMAT113

1: private car

2: ordinary ambulance (without medical accompaniment) *D_TRANSPACC*

3: emergency ambulance service

4: other: please specify EM_ENTMAT114

**Did you require in utero transfer?** 0: no; 1: yes **|__|** EM_ENTMAT115

***In utero transfer is:***

- ***A change of hospital***
- ***by specialized transport: emergency ambulance service, ambulance (does not include private car)***
- ***with or without medical accompaniment (doctor, midwife) or nurse***

**If yes**,

***« I’m now going to ask you some questions about your transfer before the birth »:***

**At the beginning of your pregnancy, was it explained to you that you might have to give birth in another maternity department if there was a complication?** 0: no; 1: yes **|__|**EM_ENTMAT116

**At the time of the transfer, were the reasons clearly explained to you? |__|**EM_ENTMAT117

1: No, not at all

2: Not really

3: Yes, reasonably clearly *D_TRANSFERTRAISON*

4: Yes, definitely

**Did you have the impression that there was good continuity of care between the first maternity department and the one where you were transferred? |__|**EM_ENTMAT118

1: No, not at all

2: Not really *D_TRANSFERTRAISON*

3: Yes, reasonably good

4: Yes, definitely

**Did you feel that you had a good welcome at the maternity department where you were transferred? |__|**EM_ENTMAT119

1: No, not at all

2: Not really *D_TRANSFERTRAISON*

3: Yes, reasonably good

4: Yes, definitely

**Were the explanations given you about the course of your pregnancy and the infant’s condition in the original maternity department and the one where you finally gave birth coherent or contradictory? |__|**EM_ENTMAT120

1: Very contradictory

2: Rather contradictory *D_EXPLICATION*

3: Quite coherent

4: Fully coherent

**Did it disturb you to find yourself in a different maternity department than the one originally planned? |__|**EM_ENTMAT121

1: No, not at all

2: No, not much *D_SENTIMENT*

3: Yes, a little

4: Yes, very much

If « Yes, very much » or «Yes, a little » were you **|__|**EM_ENTMAT122

1: anxious about the baby/babies

2: disturbed by finding yourself with a care team that you did not know *D_SENTIMENT2F*

3: both

**Questions for all women:**

**Were you in hospital for more than 48h before the birth?** 0: no; 1: yes **|__|** EM_ENTMAT123

**If yes,**

**Were the staff ready and available to answer your questions? |__|**EM_ENTMAT124

1: No, not at all

2: Not really *D_TRANSFERTRAISON*

3: Yes, reasonably available

4: Yes, definitely

**Was the information you were given coherent? |__|** EM_ENTMAT125

1: No, not at all

2: Not really *D_TRANSFERTRAISON*

3: Yes, reasonably coherent

4: Yes, definitely

**Were you able to give your opinion about your care? |__|** EM_ENTMAT126

1: No, not at all

2: Not really *D_TRANSFERTRAISON*

3: Yes, reasonably able

4: Yes, definitely

If yes, was your opinion taken into account in your care? **|__|** EM_ENTMAT127

1: No, not at all

2: Not really *D_TRANSFERTRAISON*

3: Yes, reasonably so

4: Yes, definitely

**Did you see a psychologist while you were in hospital?** 0: no; 1: yes **|__|** EM_ENTMAT128

If yes: 1: it was offered, or 2: you asked for it **|__|**EM_ENTMAT129

*D_PROPO1F*

If no: 1: it was not offered, or 2: it was offered and you refused **|__|** EM_ENTMAT130

*D_PROPO2F*

**Did you see a paediatrician before the birth of your baby/babies?** 0: no; 1: yes **|__|** EM_ENTMAT131

***« Now I’d like to ask you some questions about the way your baby is/your babies are fed »:***

***Adapt the questions according to whether the mother has only one child (or one surviving child), twins or triplets***

**Before the birth or just after, were you asked how you wanted to feed your baby/babies?** 0: no; 1: yes **|__|**EM_ENTMAT132

**Did you breast-feed or pump milk or express milk by hand in the first 3 days?** 0: no; 1: yes **|__|**EM_ENTMAT133

If yes, did you begin in the first 24 hours of the life of your baby/babies? 0: no; 1: yes **|__|**EM_ENTMAT134

**Was your baby or one of your babies transferred to another facility just after birth?** 0: no; 1: yes **|__|**EM_ENTMAT135

**If yes,**

***« And now to finish, some questions about your child: your baby/babies did not stay with you after the birth, he/she was/they were transferred to another facility. I am going to ask you some questions about the transfer »***

**Were you told before the birth that your baby/babies might be transferred?** 0: no; 1: yes

**|__|**EM_ENTMAT136

**Was the reason for the transfer clearly explained to you? |__|**EM_ENTMAT137

1: No, not at all

2: Not really *D_TRANSFERTRAISON*

3: Yes, reasonably clearly

4: Yes, definitely

***If it was a singleton pregnancy or if only one infant was transferred:***

**Were you able to see the baby before the transfer? _**0: no; 1: yes **|__|**EM_ENTMAT138

**Was the baby’s father able to see him/her? |__|**EM_ENTMAT139

1: No

2: Yes, he saw him/her *D_VOIR*

3: Yes, he saw him/her and accompanied him/her

**Were you able to be transferred to the same facility as your baby?** 0: no; 1: yes **|__|**EM_ENTMAT140

***Was it a multiple pregnancy with transfer of two or three infants?*** 0: no; 1: yes **|__|**EM_ENTMAT141

If yes,

***2^nd^ child:***

**Were you able to see your baby before the transfer?** 0: no; 1: yes **|__|**EM_ENTMAT142

**Was the baby’s father able to see him/her? |__|**EM_ENTMAT143

1: No

2: Yes, he saw him/her *D_VOIR*

3: Yes, he saw him/her and accompanied him/her

**Were you able to be transferred to the same facility as your baby?** 0: no; 1: yes **|__|**EM_ENTMAT144

***3^rd^ child:***

**Were you able to see your baby before the transfer?** 0: no; 1: yes **|__|**EM_ENTMAT145

**Was the baby’s father able to see him/her?|__|** EM_ENTMAT146

1: No

2: Yes, he saw him/her *D_VOIR*

3: Yes, he saw him/her and accompanied him/her

**Were you able to be transferred to the same facility as your baby?** 0: no; 1: yes **|__|**EM_ENTMAT147

***4^th^ child:***

**Were you able to see your baby before the transfer?** 0: no; 1: yes **|__|**EM_ENTMAT148

**Was the baby’s father able to see him/her? |__|**EM_ENTMAT149

1: No

2: Yes, he saw him/her *D_VOIR*

3: Yes, he saw him/her and accompanied him/her

**Were you able to be transferred to the same facility as your baby?** 0: no; 1: yes **|__|**EM_ENTMAT150
