# Supplementary material for: Premedication practices for delivery room intubations in premature infants in France: Results from the EPIPAGE 2 cohort study
Source: PLoS One. 2019 Apr 10;14(4):e0215150. doi: 10.1371/journal.pone.0215150 (PMC6457540; doi:10.1371/journal.pone.0215150)
Supplement: S3 File — Data that had to be fulfilled with the neonates’ files and the neonatal team. (DOC) [file pone.0215150.s003.doc]

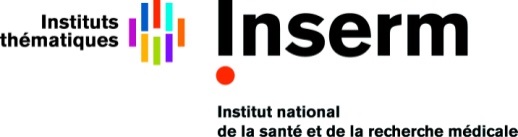


**Epidemiological study on low gestational age infants**

**Annotated**

**NEONATOLOGY QUESTIONNAIRE**

**Data to be collected from neonatal records**

**and the neonatology team**

| **Variables highlighted in red** | **Identifying variables – Not transferred** |
| --- | --- |
| **Variables highlighted in yellow** | **Indirectly identifying variables – Conditionally transferred** |
| ***Variables in green*** | **Calculated variables** |
| ***Variables in brown*** | **Synthetic variables** |
| ***Response options (d_variable)*** | **Dictionary** |
| Violet boxes | **Conditional blocks** |

**Table of contents**

**LUNG DEVELOPMENT AND RESPIRATORY COMPLICATIONS page 4**

**Surfactant page 4**

**Ventilation page 4**

**Postnatal corticosteroids page 5**

**Inhaled nitric oxide page 6**

**Bronchopulmonary dysplasia page 7**

**NEUROLOGICAL EVALUATION OF THE NEONATE page 9**

**Transfontanelle ultrasound page 9**

**Electroencephalography page 14**

**Magnetic resonance imaging page 15**

**Neurosurgical treatment page 15**

**CARE AND TREATMENT OF THE NEONATE page 16**

**Pain relief  page 16**

**Haematology page 19**

**Nephrology page 20**

**Jaundice page 21**

**Sodium levels page 21**

**Early infection page 21**

**Secondary infections page 23**

**Hemodynamics page 28**

**DEVELOPMENTAL CARE page 31**

**NUTRITION page 35**

**COMPLICATIONS page 40**

**CONGENITAL ANOMALIES page 41**

**RETINOPATHY page 42**

**HEARING SCREENING page 42**

**VACCINATION page 43**

**SUMMARY OF THE INFANT’S PATHWAY page 44**

**ACTIVE TREATMENT LIMITATION OR DISCONTINUATION page 58**

**INFANT’S CONDITION AT DISCHARGE HOME page 60**

**INFANT DECEASED IN NEONATALRESUSCITATION/INTENSIVE CARE UNIT page 62**

**Identifier of the infant**  l__l__l__l__l__ **enr_code_enfant**

**Identifier of the mother** l__l__l__l__l__ **enr_code_mere**

**Infant’s date of birth** l__l__l/ l__l__l /l__l__l__l__l **cor**_**ddn_enfant**

**Is the infant enrolled in a clinical trial:** no = 0, yes = 1 l__l **neo_na1**

If yes, please give the name of the trial:………………………………………………………………… **neo_na2**

***At admission or during the first 12 hours in the first department to which admitted:***

**Corrected birth weight l__l__l__l__l g cor*_*pds_nce**

| **EPOPé birth weight curves (z-score)** | **neo_PNzscore_epope** | continuous variable |
| --- | --- | --- |
|  |  |  |
| **EPOPé birth weight curves (percentiles)** | **neo_PNperc_epope** | continuous variable |
|  |  |  |
|  | **neo_PN10p_epope** | 0: birth weight < 10th percentile of EPOPé curves |
|  |  | 1: birth weight ≥ 10th percentile of EPOPé curves |
|  |  |  |
|  | **neo_PN3p_epope** | 0: birth weight < 3rd percentile of EPOPé curves |
|  |  | 1: birth weight ≥ 3rd percentile of EPOPé curves |

***Reference for EPOPé curves:*** *Ego A, Prunet C, Lebreton E et al. Customized and non-customized French intrauterine growth curves. I – Methodology. J Gynecol Obstet Biol Reprod 2016;45:155-64*

**Head circumference** l__l__l cm **neo_pcnce**

**Temperature** l__l__l, l__l °C **neo_nb5**

**Highest base deficit in the first 12 hours**  l__l__l, l__l mmol/l **neo_nb6**

**Lung development and respiratory complications**

**Surfactant**

**Surfactant administration, including in delivery room:** no = 0, yes = 1 l__l **neo_nc1**

If yes:

Type of surfactant: Curosurf® = 1, other = 2 l__l **neo_nc2**

*d_nc2f*

Age at first dose l__l__l h l__l__l min **neo_nc3 neo_nc4**

First dose administered in: delivery room = 1, NICU = 2 l__l **neo_nc5**

***d_nc5f***

Dose at first administration l__l__l__l mg/kg **neo_nc6**

Administration of surfactant followed by immediate extubation (INSURE): no = 0, yes =1 l__l **neo_nc7**

Total number of doses administered l__l **neo_nc8**

Age at second dose l__l__l h **neo_nc9**

Age at third dose l__l__l__l h **neo_nc10**

**Ventilation**

**Cumulative duration of respiratory assistance:**

Mechanical ventilation (MV) l__l__l__l j **neo_nc11**

Non-invasive ventilation (NIV) l__l__l__l j **neo_nc12**

High-frequency oscillation (HFO) l__l__l__l j **neo_nc13**

**High frequency oscillation (HFO) < D8:** no = 0, yes = 1 l__l **neo_nc14**

If yes, indication: primary treatment = 1, rescue = 2 l__l **neo_nc15**

***d_nc15f***

**Date of first extubation**  l__l__l/ l__l__l /l__l__l__l__l **neo_nc16**

***Age at first extubation (in days) neo_nc16b***

**Date of definitive extubation**  l__l__l/ l__l__l /l__l__l__l__l **neo_nc19**

***Age at definitive extubation (in days) neo_nc19b***

**Age at first use of CPAP**  l__l__l j l__l__l h **neo_nc22 neo_nc23**

**Date of definitive cessation of CPAP**  l__l__l/ l__l__l /l__l__l__l__l **neo_nc24**

***Age at definitive cessation of CPAP (in days) neo_nc24b***

**Date of definitive weaning off oxygen (FiO2 = 21%)**  l__...__l **neo_nc27**

***Age at definitive weaning off oxygen (FiO2 = 21%) (in days) neo_nc27b***

**Date of definitive cessation of subnasal O2 (nasal prongs)**  l__...__l **neo_nc30**

***Age at definitive cessation of subnasal O2 (in days) neo_nc30b***

| ***Infants entered in the common arm of the study, liveborn and admitted to NICU (COR_STATUTNAIS = 4 or 5)*** | | |
| --- | --- | --- |
| **Cumulative duration of mechanical ventilation** | **neo_dureeVM** | continuous (days) |
| **Age at definitive weaning from CPAP (WGA)** | **neo_sevrageCPAP** | continuous (complete weeks GA) |
| **Attempted CPAP in the first 24 hours of life** | **neo_CPAP_H24** | 0: intubation in delivery room or before H1, never extubated in the first 24h of life |
|  |  | 1: put on CPAP in delivery room or 1st extubation before 24h, whether reintubated later or not |

**Respiratory event(s):** no = 0, yes = 1 l__l **neo_nc33**

If yes:

Hyaline membrane disease: no = 0, yes = 1 l__l **neo_nc34**

Pulmonary hemorrhage: no = 0, yes = 1 l__l **neo_nc35**

Proven pulmonary arterial hypertension (PAH): no = 0, yes = 1 l__l **neo_nc36**

If yes, proven by:

Cardiac ultrasound: no = 0, yes = 1 l__l **neo_nc37**

Pre- and postductal SpO2 differential: no = 0, yes = 1 l__l **neo_nc38**

Pneumothorax: no = 0, yes = 1 l__l **neo_nc39**

Accidental extubation requiring reintubation: no = 0, yes = 1 l__l **neo_nc40**

**Postnatal corticosteroids (PCS)**

**Administration of hydrocortisone hemisuccinate (HCHS):** no = 0, yes = 1 l__l **neo_nc41**

If yes, indication  l__l **neo_nc42**

***d_nc42f***

1: Prevention of bronchopulmonary dysplasia

2: Replacement

3: Hemodynamic stabilization

**Use of PCS:** no = 0, yes = 1 l__l **neo_nc43**

If yes, molecule: dexamethasone = 1, betamethasone = 2, both = 3 l__l **neo_nc44**

***d_nc44f***

***1st course of PCS:***

**Route of administration:** systemic = 1, inhaled = 2 l__l **neo_nc45**

***d_nc45f***

**Indication**  l__l **neo_nc46**

***d_nc46f***

1: Extubation

2: Discontinuation of CPAP

3: Other

If other, please detail: **neo_nc47**

**Infant’s status at time of PCS indication:** **NEO_NC70A**

FiO2 l__l__l % **neo_nc48**

Oxygen therapy: nasal prongs = 1, CPAP = 2, MV = 3 l__l **neo_nc49**

***d_nc49f***

**Start date of corticosteroids** l__l__l/ l__l__l /l__l__l__l__l **neo_nc50**

***Age at start of corticosteroids (in days) neo_nc50b***

**End date of corticosteroids** l__l__l/ l__l__l /l__l__l__l__l **neo_nc53**

***Age at end of corticosteroids (in days) neo_nc53b***

**Highest dose used**  l__l__l, l__l__l mg/kg/j **neo_nc56**

**Cumulative dose of course n°1**  l__l__l__l mg **neo_nc57**

**Premature discontinuation of course n°1:** no = 0, yes = 1 l__l **neo_nc58**

***Total n° of courses, if more than one:***

**Administration route:** systemic = 1, inhaled = 2, both = 3 l__l **neo_nc59**

***d_nc59f***

**Date of definitive weaning off corticosteroids** l__l__l/ l__l__l /l__l__l__l__l **neo_nc60**

***Age at definitive weaning off corticosteroids (in days) neo_nc60b***

**Highest dose used**  l__l__l, l__l__l__l mg/kg/j **neo_nc63**

**Total cumulative systemic dose**  l__l__l__l mg/kg **neo_nc64**

**Total cumulative inhaled dose**  l__l__l__l mg/kg **neo_nc65**

**Inhaled nitric oxide (iNO)**

Use of **iNO**: no = 0, yes = 1 l__l **neo_nc66**

If yes:

**Indication:**

Anti-inflammatory: no = 0, yes = 1 l__l **neo_nc67**

Refractory hypoxemia: no = 0, yes = 1 l__l **neo_nc68**

If yes, associated with PAH: no = 0, yes = 1 l__l **neo_nc69**

Other: no = 0, yes = 1 l__l **neo_nc70**

If yes, please detail: ….  **neo_nc70a**

**Cardiac ultrasound showed iNO still required:** no = 0, yes = 1 l__l **neo_nc71**

**Start date**  l__l__l/ l__l__l /l__l__l__l__l **neo_nc72**

***Age at start (in days) neo_nc72b***

**Date of definitive weaning**  l__l__l/ l__l__l /l__l__l__l__l **neo_nc75**

***Age at definitive weaning (in days) neo_nc75b***

**Highest dose used**  l__l__l ppm **neo_nc78**

**Bronchopulmonary dysplasia**

**Number of days of O2 from birth to D28** l__l__l j **neo_nc79**

**Infant receiving O2 at D28:** no = 0, yes = 1 l__l **neo_nc80**

***Infant receiving O2 at D28: no = 0, yes = 1 l__l neo_O2J28***

If yes, type of oxygen treatment: nasal prongs = 1, CPAP = 2, MV = 3 l__l **neo_nc81**

***d_nc81f***

***Respiratory assistance or O2 at D28*** *no = 0, yes = 1 l__l* ***neo_bdpj28***

**Number of days on O2 from birth to 36 WGA**  l__l__l__l j **neo_nc82**

**Infant receiving O2 at 36 WGA:** no = 0, yes = 1 l__l **neo_nc83**

***Infant receiving O2 at 36 WGA: no = 0, yes = 1 l__***l ***neo_O2S36***

If yes, type of oxygen therapy: nasal prongs = 1, CPAP = 2, MV = 3 l__l **neo_nc84**

***d_nc84f***

**FiO2 at 36 WGA** l__l__l % **neo_nc85**

***FiO2 at 36 WGA l__l__l % neo_fio2***

**Walsh test done at 36 WGA*:** no = 0, yes = 1 l__l **neo_nc86**

**No BPD if infant maintains above 88% saturation for 30 min with FiO2 at 21%*

If yes:

Lowest value of SpO2 if FiO2 = 21% l__l__l **neo_nc87**

Weaning off O2 after test: no = 0, yes = 1 l__l **neo_nc88**

If no, please give reason(s):…………………………………………………………………….. **neo_nc89**

***Bronchopulmonary dysplasia in 4 stages***  *l__l* ***neo_DBP_grav***

*0: No BPD*

*1: Mild BPD* (≥ 28 days of O2 and spontaneous breathing of room air at 36 WGA)

*2: Moderate BPD* (≥ 28 days of O2 and mechanical ventilation or CPAP or FiO2 > 21% at 36 WGA)

*3: Severe BPD* (≥ 28 days of O2 and mechanical ventilation or CPAP or FiO2 ≥ 30% at 36 WGA)

***Moderate or severe bronchopulmonary dysplasia***  *l__l* ***neo_DBP_S36***

***(infants alive at 36 WGA)***

*0: No BPD or mild BPD at 36 WGA*

*1: Moderate or severe BPD at 36 WGA*

***O_2_ for at least 28 days with either FiO_2_ ≥ 30% or mechanical ventilation or CPAP at 36 WGA:***

*no = 0, yes = 1*  *l__l* ***neo_bdpsevere***

**Synagis®**

**Was the infant given palivizumab (Synagis®):** no = 0, yes = 1 l__l **neo_nc90**

If yes,

date  l__l__l/ l__l__l /l__l__l__l__l **neo_nc91**

***Age when Synagis was given neo_nc91b***

**Neurological evaluation of the neonate**

**Transfontanelle ultrasound (TFU)**

***TFU scan(s) done during the 1st week of life****:* no = 0, yes = 1  l__l **neo_nd1**

***If yes, complete the items below that were observed on the TFU scan(s) during the first week of life.***

*If the same lesion was observed several times, give the highest stage.*

**Hemorrhage:** no = 0, yes = 1 l__l neo_nd2

If yes,

**Highest stage** l__l **neo_nd3**

***d_nd3f***

1: Subependymal hemorrhage (SEH)

2: Intraventricular hemorrhage (IVH) without ventricular dilation

3: Intraventricular hemorrhage (IVH) with ventricular dilation

**Measurements of ventricular index according to Levene**

**(cf. Fig. 1, Guide to completing the neonatal questionnaire):**

Right  l__l__l, l__l__l mm **neo_nd4**

Left  l__l__l, l__l__l mm **neo_nd5**

**Parenchymal abnormalities:** no = 0, yes = 1 l__l **neo_nd6**

If yes,

**Early findings of periventricular hyperechogenic areas*:** no = 0, yes = 1 l__l **neo_nd7**

**Echogenicity greater than or equal to that of the choroid plexus*

If yes, unilateral = 1, bilateral = 2 l__l **neo_nd8**

***d_nd8f***

If unilateral, Volpe infarction (formerly called grade 4 hemorrhage): no = 0, yes = 1 l__l **neo_nd9**

**Cavitation(s):** no = 0, yes = 1 l__l **neo_nd10**

If yes,

Single = 1, multiple = 2 l__l **neo_nd11**

***d_nd11f***

Unilateral = 1, bilateral = 2 l__l **neo_nd12**

***d_nd12f***

If unilateral, porencephaly*:* no = 0, yes = 1 l__l **neo_nd13**

**Other abnormality(ties):** no = 0, yes = 1 l__l **neo_nd14**

If yes, please detail: …………………………………………………………………………………… **neo_nd15**

**The synthetic variables below relate to infants entering and exiting the common arm alive, aged 22–34 WGA and admitted to the NICU with at least one TFU scan**

***Presence of a cyst*** *l__l* ***neo_kyste_1***

*0: No 3: Paraventricular cyst* ***d_kyste_1f***

*1: Choroid plexus cyst 4: Frontal cyst*

*2: Subependymal cyst 5: Other*

***Presence of a thalamostriate vessel:*** *no = 0, yes = 1 l__l* ***neo_vaisso_1***

***Presence of a ventricular dilation:*** *no = 0, yes = 1 l__l* ***neo_dilatation_1***

***Presence of an abnormality of the central grey nuclei (CGN):*** *no = 0, yes = 1 l__l* ***neo_ngc_1***

***Presence of a cerebellar abnormality:*** *no = 0, yes = 1 l__l* ***neo_cervelet_1***

***Presence of bilateral hemorrhage:*** *no = 0, yes = 1 l__l* ***neo_volpebilat_1***

***TFU scan(s) done during the 2^nd^ week of life:*** no = 0, yes = 1 l__l **neo_nd16**

***If yes, complete the items below that were observed on the TFU scan(s) during the second week of life.***

*If the same lesion was observed several times, give the highest stage.*

**Hemorrhage:** no = 0, yes = 1 __________________________________________________ l__l **neo_nd17**

If yes,

**Highest stage** l__l **neo_nd18**

***d_nd3f***

1: Subependymal hemorrhage (SEH)

2: Intraventricular hemorrhage (IVH) without ventricular dilation

3: Intraventricular hemorrhage (IVH) with ventricular dilation

**Measurements of ventricular index according to Levene**

**(cf. Fig. 1, Guide to completing the neonatal questionnaire):**

Right  l__l__l, l__l__l mm **neo_nd19**

Left  l__l__l, l__l__l mm **neo_nd20**

**Parenchymal abnormalities:** no = 0, yes = 1 l__l **neo_nd21**

If yes,

**Periventricular hyperechogenic areas*:** no = 0, yes = 1 l__l **neo_nd22**

**Echogenicity greater than or equal to that of the choroid plexus*

If yes, unilateral = 1, bilateral = 2 l__l **neo_nd23**

***d_nd8f***

If unilateral, Volpe infarction (formerly called grade 4 hemorrhage): no = 0, yes = 1 l__l **neo_nd24**

**Cavitation(s):** no = 0, yes = 1 l__l **neo_nd25**

If yes,

Single = 1, multiple = 2 l__l **neo_nd26**

***d_nd11f***

Unilateral = 1, bilateral = 2 l__l **neo_nd27**

***d_nd12f***

If unilateral, porencephaly*:* no = 0, yes = 1 l__l **neo_nd28**

**Other abnormality(ties):** no = 0, yes = 1 l__l **neo_nd29**

If yes, please detail: ………………………………………………………………………………… **neo_nd30**

**The synthetic variables below relate to infants entering and exiting the common arm alive, aged 22–34 WGA and admitted to the NICU with at least one TFU scan**

***Presence of a cyst l__l neo_kyste_2***

*0: No 3: Paraventricular cyst* ***d_kyste_1f***

*1: Choroid plexus cyst 4: Frontal cyst*

*2: Subependymal cyst 5: Other*

***Presence of a thalamostriate vessel:*** *no = 0, yes = 1* ***l__l neo_vaisso_2***

***Presence of ventricular dilation:*** *no = 0, yes = 1* ***l__l neo_dilatation_2***

***Presence of an abnormality of the central grey nuclei (CGN):*** *no = 0, yes = 1* ***l__l neo_ngc_2***

***Presence of a cerebellar abnormality:*** *no = 0, yes = 1* ***l__l neo_cervelet_2***

***Presence of bilateral hemorrhage:*** *no = 0, yes = 1* ***l__l neo_volpebilat_2***

***TFU scan(s) done during the 3rd week of life:*** no = 0, yes = 1 l__l neo_**nd31**

***If yes, complete the items below that were observed on the TFU scan(s) during the third week of life****.*

*If the same lesion was observed several times, give the highest stage.*

**Hemorrhage:** no = 0, yes = 1 _______________________________________ l__l **neo_nd32**

If yes,

**Highest stage** l__l neo_**nd33**

***d_nd3f***

1: Subependymal hemorrhage (SEH)

2: Intraventricular hemorrhage (IVH) without ventricular dilation

3: Intraventricular hemorrhage (IVH) with ventricular dilation

**Measurements of ventricular index according to Levene:**

Right  l__l__l, l__l__l mm **neo_nd34**

Left  l__l__l, l__l__l mm **neo_nd35**

**Parenchymal abnormalities:** no = 0, yes = 1 l__l **neo_nd36**

If yes,

**Periventricular hyperechogenic areas*:** no = 0, yes = 1 l__l **neo_nd37**

**Echogenicity greater than or equal to that of the choroid plexus*

If yes, unilateral = 1, bilateral = 2 l__l **neo_nd38**

***d_nd8f***

If unilateral, Volpe infarction (formerly called grade 4 hemorrhage): no = 0, yes = 1 l__l **neo_nd39**

**Cavitation(s):** no = 0, yes = 1 l__l **neo_nd40**

If yes,

Single = 1, multiple = 2 l__l **neo_nd41**

***d_nd11f***

Unilateral = 1, bilateral = 2 ________________________________________________ l__l **neo_nd42**

***d_nd12f***

If unilateral, porencephaly*:* no = 0, yes = 1 l__l **neo_nd43**

**Other abnormality(ties):** no = 0, yes = 1 l__l **neo_nd44**

If yes, please detail: …………………………………………………………………………………… **neo_nd45**

**The synthetic variables below relate to infants entering and exiting the common arm alive, aged 22–34 WGA and admitted to the NICU with at least one TFU scan**

***Presence of a cyst*** *l__l* ***neo_kyste_3***

*0: No 3: Paraventricular cyst* ***d_kyste_1f***

*1: Choroid plexus cyst 4: Frontal cyst*

*2: Subependymal cyst 5: Other*

***Presence of a thalamostriate vessel:*** *no = 0, yes = 1 l__l* ***neo_vaisso_3***

***Presence of ventricular dilation:*** *no = 0, yes = 1 l__l* ***neo_dilatation_3***

***Presence of an abnormality of the central grey nuclei (CGN):*** *no = 0, yes = 1 l__l* ***neo_ngc_3***

***Presence of a cerebellar abnormality:*** *no = 0, yes = 1 l__l* ***neo_cervelet_3***

***Presence of bilateral hemorrhage:*** *no = 0, yes = 1 l__l* ***neo_volpebilat_3***

***Last TFU scan before discharge home:***

**Date of last TFU**  l__l__l/ l__l__l /l__l__l__l__l **neo_nd46**

***Age at last TFU (in days) neo_nd46b***

Investigation carried out by:

senior practitioner = 1, non-senior practitioner = 2 l__l **neo_nd49**

***d_nd49f***

**Widening of the subarachnoid spaces*:** no = 0, yes = 1 l__l **neo_nd50**

* *Normal width of the subarachnoid spaces: all measurements less than 4 mm*

If yes, measurement of the subarachnoid spaces:

Measurement on a section through the 3^rd^ ventricle (cf. Fig. 2, Guide to completing the neonatal questionnaire):

Right l__l__l, l__l__l mm **neo_nd51**

Left  l__l__l, l__l__l mm **neo_nd52**

Measurement on a posterior ventricular section (cf. Fig. 3, Guide to completing the neonatal questionnaire):

Right  l__l__l, l__l__l mm **neo_nd53**

Left  l__l__l, l__l__l mm **neo_nd54**

**Widening of the interhemispheric fissure*:** no = 0, yes = 1 l__l **neo_nd55**

**Normal width of the interhemispheric fissure: less than 3 mm*

If yes, measurement (cf. Fig. 2, Guide to completing the neonatal questionnaire) l__l__l, l__l__l mm

**neo_nd56**

**Hemorrhage:** no = 0, yes = 1 l__l **neo_nd57**

If yes,

**Highest stage** l__l **neo_nd58**

***d_nd3f***

1: Subependymal hemorrhage (SEH)

2: Intraventricular hemorrhage (IVH) without ventricular dilation

3: Intraventricular hemorrhage (IVH) with ventricular dilation

**Measurements of ventricular index according to Levene:**

Right  l__l__l, l__l__l mm **neo_nd59**

Left  l__l__l, l__l__l mm **neo_nd60**

**Parenchymal abnormalities:** no = 0, yes = 1 l__l **neo_nd61**

If yes,

**Periventricular hyperechogenic areas*:** no = 0, yes = 1 l__l **neo_nd62**

**Echogenicity greater than or equal to that of the choroid plexus*

If yes,

unilateral = 1, bilateral = 2 l__l **neo_nd63**

***d_nd8f***

If unilateral, Volpe infarction (formerly called grade 4 hemorrhage): no = 0, yes = 1 l__l **neo_nd64**

**Cavitation(s):** no = 0, yes = 1 l__l **neo_nd65**

If yes,

Single = 1, multiple = 2 l__l **neo_nd66**

***d_nd11f***

Unilateral = 1, bilateral = 2 l__l **neo_nd67**

***d_nd12f***

If unilateral, porencephaly: no = 0, yes = 1 l__l **neo_nd68**

**Other abnormality(ties):** no = 0, yes = 1 l__l **neo_nd69**

If yes, please detail: …………………………………………………………………………………… **neo_nd70**

**The synthetic variables below relate to infants entering and exiting the common arm alive, aged 22–34 WGA and admitted to the NICU with at least one TFU scan**

***Presence of a cyst*** *l__l* ***neo_kyste_S***

*0: No 3: Paraventricular cyst* ***d_kyste_1f***

*1: Choroid plexus cyst 4: Frontal cyst*

*2: Subependymal cyst 5: Other*

***Presence of a thalamostriate vessel:*** *no = 0, yes = 1 l__l* ***neo_vaisso_S***

***Presence of a ventricular dilation:*** *no = 0, yes = 1 l__l* ***neo_dilatation_S***

***Presence of an abnormality of the central grey nuclei (CGN):*** *no = 0, yes = 1 l__l* ***neo_ngc_S***

***Presence of a cerebellar abnormality:*** *no = 0, yes = 1 l__l* ***neo_cervelet_S***

***Presence of bilateral hemorrhage:*** *no = 0, yes = 1 l__l* ***neo_volpebilat_S***

***TFU carried out:*** *l__l* ***neo_etfrealisee***

*0- no TFU 1- at least one TFU 2-at least one of 4 TFUs missing, others not done* ***d_etfrealisee***

***Cavitation on any TFU*** (excluding porencephaly)***:*** *l__l* ***neo_cavitationetf***

*0-No cavitation on any TFU 1- Cavitation on at least one TFU*  ***d_cavitationetf***

***Presence of IVH on any TFU (indicate the most severe stage): l__l*** neo_superhivvolpe

| *1- Volpe* | ***d_superhivvolpe*** |
| --- | --- |
| *2- IVH3* | *30- Unspecified hemorrhage* |
| *3- IVH2* (with choroid plexus cysts) | *35- No hemorrhage* |
| *4- IVH1* (with subependymal cysts) |  |

***Presence of severe lesions:*** *l__l* ***neo_ls***

*0-No severe lesion (cavitation, IVH4, IVH3) 1- Severe lesions* ***d_ls***

***Date of onset of the most severe lesion IVH4>IVH3>IVH2>IVH1: l__l*** neo_superdelai

| *1- 1st week* | ***d_superdelai*** |
| --- | --- |
| *2- 2nd week* | *4- 4th week* |
| *3- 3rd week* |  |

***Parenchymal abnormality on any TFU*** (including Volpe and porencephaly)***: ___________*** *l__l* ***neo_parenetf***

*0-No parenchymal abnormality on any TFU 1- Parenchymal abnormality on at least one TFU* ***d_parenetf***

***Volpe hemorrhage on any TFU*** (including Volpe and porencephaly)***:*** *___________ l__l* ***neo_volpeetf***

*0-No Volpe hemorrhage on any TFU 1- Volpe hemorrhage on at least one TFU* ***d_volpeetf***

**Electroencephalography (EEG)**

**Were one or several EEGs carried out in the neonatal period:** no = 0, yes = 1 l__l **neo_nd71**

If yes, how many  l__l **neo_nd72**

***First EEG:***

**Date** **of 1st EEG**  l__l__l/ l__l__l /l__l__l__l__l  **neo_nd73**

***Age at 1st EEG (in days) neo_nd73b***

**Can you give details on the background pattern:** no = 0, yes = 1 l__l **neo_nd76**

If yes, **background pattern:** Normal: no = 0, yes 1 l__l **neo_nd77**

Dysmature*: no = 0, yes = 1 l__l **neo_nd78**

Disorganized **: no = 0, yes = 1 l__l **neo_nd79**

Too discontinuous***: no = 0, yes = 1 l__l **neo_nd80**

Flat trace: no = 0, yes = 1 l__l **neo_nd81**

** maturational delay of 15 days or more in relation to gestational age*

*** poorly defined maturational features and gestational age impossible to determine*

**** discontinuity > 60 sec before 28 WGA; > 30 sec before 30 WGA, > 20 sec after 30 WGA*

**Abnormal patterns on EEG:** no = 0, yes = 1 l__l **neo_nd82**

If yes, **can you give details on the abnormal patterns:** no = 0, yes = 1 l__l **neo_nd83**

If yes**, abnormal patterns:** Positive rolandic sharp waves type A ≥ 1/min: no = 0, yes = 1 l__l **neo_nd84**

Positive rolandic sharp waves type A < 1/min: no = 0, yes = 1 l__l **neo_nd85**

Abnormal delta brushes: no = 0, yes = 1 l__l **neo_nd86**

Electrical seizures: no = 0, yes = 1 l__l **neo_nd87**

**Conclusion of the examination:** normal = 1, moderate alterations = 2, severe alterations = 3 l__l **neo_nd88**

***d_nd88f***

***Later EEGs:***

**Did later EEGs (except the most recent one) show abnormalities that were not observed on the first**

**trace:** no = 0, yes = 1 l__l **neo_nd89**

If yes,

**Abnormalities of the background pattern:** Dysmature*: no = 0, yes = 1 l__l **neo_nd90**

Disorganized**: no = 0, yes = 1 l__l **neo_nd91**

Too discontinuous***: no = 0, yes = 1 l__l **neo_nd92**

Flat trace: no = 0, yes = 1 l__l **neo_nd93**

**Abnormal patterns:** Positive rolandic sharp waves type A ≥ 1/min: no = 0, yes = 1 l__l **neo_nd94**

Positive rolandic sharp waves type A < 1/min: no = 0, yes = 1 l__l **neo_nd95**

Abnormal delta brushes: no = 0, yes = 1 l__l **neo_nd96**

Electrical seizures: no = 0, yes = 1 l__l **neo_nd97**

***Last EEG:***

**Date of last EEG**  l__l__l/ l__l__l /l__l__l__l__l **neo_nd98**

***Age at last EEG (in days) neo_nd98b***

**Can you give details on the background pattern:** no = 0, yes = 1 l__l **neo_nd101**

If yes, **background pattern:** Normal: no = 0, yes 1 l__l **neo_nd102**

Dysmature*: no = 0, yes = 1 l__l **neo_nd103**

Disorganized**: no = 0, yes = 1 l__l **neo_nd104**

Too discontinuous***: no = 0, yes = 1 l__l **neo_nd105**

Flat trace: no = 0, yes = 1 l__l **neo_nd106**

**Abnormal patterns on EEG:** no = 0, yes = 1 l__l **neo_nd107**

If yes, **can you give details on the abnormal patterns:** no = 0, yes = 1 l__l **neo_nd108**

If yes, **abnormal patterns:** Positive rolandic sharp waves type A ≥ 1/min: no = 0, yes = 1 l__l **neo_nd109**

Positive rolandic sharp waves type A < 1/min: no = 0, yes = 1 l__l **neo_nd110**

Abnormal delta brushes: no = 0, yes = 1 l__l **neo_nd111**

Electrical seizures: no = 0, yes = 1 l__l **neo_nd112**

**Conclusion of the examination:** normal = 1, moderate alterations = 2, severe alterations = 3 l__l **neo_nd113**

***d_nd88f***

**Summary of EEG monitoring** l__l **neo_nd114**

***d_nd114f***

1: All EEGs normal

2: Transient moderate alterations

3: Persistent alterations

4: Severe alterations

**Magnetic resonance imaging (MRI)**

**Was an MRI scan done:** no = 0, yes = 1 l__l **neo_nd115**

If yes,

Total number of MRI scans l__l **neo_nd116**

Result of the most recent MRI scan: normal = 1, abnormal = 2 l__l **neo_nd117**

***d_nd117f***

If abnormal, please detail: ……………………………………………………………………….. **neo_nd118**

**Neurosurgical treatment**

**Ventriculoperitoneal shunt:** no = 0, yes = 1 l__l **neo_nd119**

If yes, number of shunts l__l **neo_nd120**

**Ventriculocisternostomy:** no = 0, yes = 1 l__l **neo_nd121**

**Care and treatment of the neonate**

**Pain relief**

***Sedative and/or analgesic treatment with class III medications:***

**Has the infant received sedative and/or analgesic treatment with class III medications:**

no = 0, yes = 1 l__l **neo_ne1**

If yes, **has the infant received any of the following treatments at least once, even if only for a short time** (for example, extubation):

**Morphine:** no = 0, yes = 1 l__l **neo_ne2**

If yes,

Occasional treatment: no = 0, yes = 1 l__l **neo_ne3**

Continuous treatment: no = 0, yes = 1 l__l **neo_ne4**

If yes,

Treatment start date l__l__l/ l__l__l /l__l__l__l__l **neo_ne5**

*Age at start of treatment (in days)* ***neo_ne5b***

Cumulative number of days of administration l__l__l j **neo_ne8**

**Fentanyl:** no = 0, yes = 1 l__l **neo_ne9**

If yes,

Treatment start date l__l__l/ l__l__l /l__l__l__l__l **neo_ne10**

*Age at start of treatment (in days)* ***neo_ne10b***

Cumulative number of days of administration l__l__l j **neo_ne13**

**Sufentanil:** no = 0, yes = 1 l__l **neo_ne14**

If yes,

Treatment start date l__l__l/ l__l__l /l__l__l__l__l **neo_ne15**

*Age at start of treatment (in days)* ***neo_ne15b***

Cumulative number of days of administration l__l__l j **neo_ne18**

**Midazolam** **(Hypnovel®):** no = 0, yes = 1 l__l **neo_ne19**

If yes,

Treatment start date l__l__l/ l__l__l /l__l__l__l__l **neo_ne20**

*Age at start of treatment (in days)* ***neo_ne20b***

Cumulative number of days of administration l__l__l j **neo_ne23**

**Ketamine:** no = 0, yes = 1 l__l **neo_ne24**

If yes,

Treatment start date l__l__l/ l__l__l /l__l__l__l__l **neo_ne25**

*Age at start of treatment (in days)* ***neo_ne25b***

Cumulative number of days of administration l__l__l j **neo_ne28**

**Other:** no = 0, yes = 1 l__l **neo_ne29**

If yes, Treatment 1: please specify: **neo_ne30**

Treatment start date l__l__l/ l__l__l /l__l__l__l__l **neo_ne31**

*Age at start of treatment (in days)* ***neo_ne31b***

Cumulative number of days of administration l__l__l j **neo_ne34**

Treatment 2: please specify: ………………………………………………………………. **neo_ne35**

Treatment start date l__l__l/ l__l__l /l__l__l__l__l **neo_ne36**

*Age at start of treatment (in days)*  ***neo_ne36b***

Cumulative number of days of administration l__l__l j **neo_ne39**

***Sedative and/or analgesic treatment with class I or II medications:***

**Has the infant received sedative and/or analgesic treatment with class I or II medications:**

no = 0, yes = 1 l__l **neo_ne40**

If yes, **has the infant received any of the following treatments at least once**:

**Oral paracetamol:** no = 0, yes = 1 l__l **neo_ne41**

If yes,

Treatment start date l__l__l/ l__l__l /l__l__l__l__l **neo_ne42**

*Age at start of treatment (in days)* ***neo_ne42b***

Cumulative number of days of administration l__l__l j **neo_ne45**

**Proparacetamol (Perfalgan®):** no = 0, yes = 1 l__l **neo_ne46**

If yes,

Treatment start date l__l__l/ l__l__l /l__l__l__l__l **neo_ne47**

*Age at start of treatment (in days)* ***neo_ne47b***

Cumulative number of days of administration l__l__l j **neo_ne50**

**Nalbuphine (Nubain®):** no = 0, yes = 1 l__l **neo_ne51**

If yes,

Treatment start date l__l__l/ l__l__l /l__l__l__l__l **neo_ne52**

*Age at start of treatment (in days)* ***neo_ne52b***

Cumulative number of days of administration l__l__l j **neo_ne55**

**Codeine:** no = 0, yes = 1 l__l **neo_ne56**

If yes,

Treatment start date l__l__l/ l__l__l /l__l__l__l__l **neo_ne57**

*Age at start of treatment (in days)*  ***neo_ne57b***

Cumulative number of days of administration l__l__l j **neo_ne60**

**Other:** no = 0, yes = 1 l__l **neo_ne61**

If yes,

please specify: **neo_ne62**

Treatment start date l__l__l/ l__l__l /l__l__l__l__l **neo_ne63**

*Age at start of treatment (in days)* ***neo_ne63b***

Cumulative number of days of administration l__l__l j **neo_ne66**

**Has the infant had general anesthesia during a surgical procedure:** no = 0, yes = 1 l__l **neo_ne67**

If yes, surgery for:

Patent ductus arteriosus: no = 0, yes = 1 l__l **neo_ne68**

Necrotizing enterocolitis: no = 0, yes = 1 l__l **neo_ne69**

Inguinal hernia: no = 0, yes = 1 l__l **neo_ne70**

Catheter placement: no = 0, yes = 1 l__l **neo_ne71**

Other: no = 0, yes = 1 l__l **neo_ne72**

If yes, please detail: ……………………………………………………………………………… **neo_ne73**

***Pain management practices for intubation:***

**Has the infant been intubated or reintubated after the delivery room:** no = 0, yes = 1 l__l **neo_ne74**

If yes,

Number of times l__l **neo_ne75**

Usual mode of intubation: nasal = 1, oral = 2 l__l **neo_ne76**

***d_ne76f***

Absence at least once of sedative and/or analgesic treatment during planned or semi-emergency intubation:

no = 0, yes = 1 l__l **neo_ne77**

***Apnea and bradycardia:***

**Has the infant been treated for apnea/bradycardia:** no = 0, yes = 1 l__l **neo_ne79**

**If yes,**

With **caffeine:** no = 0, yes = 1 l__l **neo_ne80**

If yes,

Start date l__l__l/ l__l__l /l__l__l__l__l **neo_ne81**

*Age at start (in days)* ***neo_ne81b***

End date  l__l__l/ l__l__l /l__l__l__l__l **neo_ne84**

Age at discontinuation (in days)  ***neo_ne84b***

Indication: curative = 1, prophylactic = 2, prophylactic then curative = 3 l__l **neo_ne87**

***d_ne87f***

With **doxapram:** no = 0, yes = 1 l__l **neo_ne88**

If yes,

Start date l__l__l/ l__l__l /l__l__l__l__l **neo_ne89**

*Age at start (in days)* ***neo_ne89b***

End date  l__l__l/ l__l__l /l__l__l__l__l **neo_ne92**

Age at discontinuation (in days) ***neo_ne92b***

Indication: curative = 1, prophylactic = 2, prophylactic then curative = 3 l__l **neo_ne95**

***d_ne87f***

By **nasal ventilation or CPAP:**no = 0, yes = 1 l__l **neo_ne96**

If yes,

Start date l__l__l/ l__l__l /l__l__l__l__l **neo_ne97**

*Age at start (in days)*  ***neo_ne97b***

End date  l__l__l/ l__l__l /l__l__l__l__l **neo_ne100**

Age at discontinuation (in days)  **neo_ne100b**

Indication: curative = 1, prophylactic = 2, prophylactic then curative = 3 l__l **neo_ne103**

***d_ne87f***

**Has the infant been intubated or reintubated for apnea/bradycardia:** no = 0, yes = 1 l__l **neo_ne104**

If yes, apnea/bradycardia accompanied by infection: no = 0, yes = 1 l__l **neo_ne105**

**Hematology**

**Thrombocytopenia before D7:** no = 0, yes = 1 l__l **neo_ne106**

If yes: lowest platelet level before transfusion l__l__l__l__l__l__l /mm^3^ **neo_ne107**

**Transfusion of platelet concentrates before D7:** no = 0, yes = 1 l__l **neo_ne108**

If yes,

Indication for the 1st transfusion:

Prophylactic: no = 0, yes = 1 l__l **neo_ne109**

Therapeutic (bleeding): no = 0, yes = 1 l__l **neo_ne110**

Prophylactic before invasive procedure: no = 0, yes = 1 l__l **neo_ne111**

**Total number of platelet concentrate transfusions after D7** l__l **neo_ne112**

**Hemoglobin level at birth**  l__l__l, l__l g/dl **neo_ne113**

Date l__l__l/ l__l__l /l__l__l__l__l **neo_ne114**

*Age (in days)* ***neo_ne114b***

**Lowest hemoglobin level during hospital stay**  l__l__l, l__l g/dl **neo_ne117**

Date l__l__l/ l__l__l /l__l__l__l__l **neo_ne118**

*Age (in days)* ***neo_ne118b***

**Hemoglobin level at infant’s discharge home** l__l__l, l__l g/dl **neo_ne121**

Date l__l__l/ l__l__l /l__l__l__l__l **neo_ne122**

*Age (in days)* ***neo_ne122b***

**Transfusion of packed red blood cells:** no = 0, yes = 1 l__l **neo_ne125**

If yes, number of transfusions l__l **neo_ne126**

**Polymorphonuclear neutrophils < 1500/mm3 before D7:** no = 0, yes = 1 l__l **neo_ne127**

**Transfusional event:** no = 0, yes = 1 l__l **neo_ne128**

If yes, please detail: **neo_ne128a**

***Prevention of anemia:***

**Preventive EPO (Neorecormon®):** no = 0, yes = 1 l__l **neo_ne129**

If yes,

Start date l__l__l/ l__l__l /l__l__l__l__l **neo_ne130**

*Age at start (in days)* ***neo_ne130b***

End date l__l__l/ l__l__l /l__l__l__l__l **neo_ne133**

*Age at discontinuation (in days)* ***neo_ne133b***

Route of administration: intravenous = 1, subcutaneous = 2, both = 3 l__l **neo_ne136**

***d_ne136f***

**Iron**: no = 0, yes = 1 l__l **neo_ne137**

If yes,

Start date l__l__l/ l__l__l /l__l__l__l__l **neo_ne138**

*Age at start (in days)* ***neo_ne138b***

**Nephrology**

**Highest creatinine value between D3 and D7**  l__l__l__l, l__l µmol/l **neo_ne141**

Date l__l__l/ l__l__l /l__l__l__l__l **neo_ne142**

*Age (in days)* ***neo_ne142b***

**Highest creatinine value after D7**  l__l__l__l, l__l µmol/l **neo_ne145**

Date l__l__l/ l__l__l /l__l__l__l__l **neo_ne146**

*Age (in days)* ***neo_ne146b***

**Last creatinine value measured before discharge home** l__l__l__l, l__l µmol/l **neo_ne149**

Date l__l__l/ l__l__l /l__l__l__l__l **neo_ne150**

*Age (in days)* ***neo_ne150b***

**Renal ultrasound performed:** no = 0, yes = 1 l__l **neo_ne153**

If yes,

Date of first ultrasound l__l__l/ l__l__l /l__l__l__l__l **neo_ne154**

*Age at first ultrasound (in days)* ***neo_ne154b***

Result in detail:……………………………………………………………………………………… **neo_ne157**

Date of last available ultrasound l__l__l/ l__l__l /l__l__l__l__l **neo_ne158**

*Age at last ultrasound (in days)* ***neo_ne158b***

Result in detail:…………………………………………………………………………………………. **neo_ne161**

**Antihypertensive treatment given:** no = 0, yes = 1 l__l **neo_ne162**

If yes, please specify treatment:…………………………………………………… ……………. **neo_ne163**

**Was renal insufficiency among the diagnoses made during the infant’s stay in the neonatal unit ?**

no = 0, yes = 1 ________________________________ l__l **neo_ne164**

**Jaundice**

**Highest bilirubin level before D7**  l__l__l__l µmol/l **neo_ne165**

or l__l__l__l mg/l **neo_ne166**

**Phototherapy:** no = 0, yes = 1 l__l **neo_ne167**

**Sodium levels**

**Highest sodium level before D7** l__l__l__l mmol/l **neo_ne168**

Early infection (≤ 72h of life)

| **Neonatal bacterial infection**  ***(Infants entered in the common arm, liveborn and admitted to NICU Unclassified: death before D5 with no clearly identified infectious cause=***  0: no bacterial infection | **neo_infection_precoce_final** |
| --- | --- |
| 1: probable bacterial infection (antibiotherapy started before 72h and duration ≥ 5 days) |  |
| 2: proven bacterial infection with positive blood culture or CSF before 72h |  |

**Date of onset of the infectious episode** l__l__l/ l__l__l /l__l__l__l__l **neo_ne170**

***Age at start of the infectious episode (in days) neo_ne170b***

**Unconfirmed infection** (anti-infection treatment ≤ 3 days): no = 0, yes = 1 l__l **neo_ne173**

**Clinical infection** (anti-infection treatment > 3 days): no = 0, yes = 1 l__l **neo_ne174**

**Infection confirmed by microbiology:** no = 0, yes = 1 l__l **neo_ne175**

**Identified in** l__l **neo_ne176**

***d_ne176f***

1: Mother and infant

2: Infant only

3: Mother only

**Bacteria** l__l **neo_ne177**

***d_ne177f***

0: Unidentified = 0

1: Strepto B = 1

2: E. coli = 2

3: Other = 3

If other, please specify (including combinations of bacteria):………………………… **neo_ne178**

**Virus:** no = 0, yes = 1 l__l **neo_ne179**

If yes, please specify: …………………………………………………………………….……………. **neo_ne180**

**Yeast infections:** no = 0, yes = 1 l__l **neo_ne181**

If yes, please specify: ……………………………………………………………………….…………. **neo_ne182**

**Location:**

Bloodstream (bacteremia): no = 0, yes = 1 l__l **neo_ne183**

Trachea: no = 0, yes = 1 l__l **neo_ne184**

Gastric fluid: no = 0, yes = 1 l__l **neo_ne185**

Anus: no = 0, yes = 1 l__l **neo_ne186**

Ears: no = 0, yes = 1 l__l **neo_ne187**

Local skin/soft tissue infection: no = 0, yes 1 l__l **neo_ne188**

Cerebrospinal fluid: no = 0, yes = 1 l__l **neo_ne189**

Other: no = 0, yes = 1 l__l **neo_ne190**

**Resistant bacteria:** no = 0, yes = 1 l__l **neo_ne191**

If yes,

Ampicillin-resistant: no = 0, yes = 1 l__l **neo_ne192**

C3G-resistant: no = 0, yes = 1 l__l **neo_ne193**

Carbapenem-resistant: no = 0, yes = 1 l__l **neo_ne194**

**Highest C-reactive protein (CRP) value during the episode**  l__l__l__l mg/l **neo_ne195**

**Treatment:**

Ampicillin: no = 0, yes = 1 l__l **neo_ne196**

C3G: no = 0, yes = 1 l__l **neo_ne197**

Aminoglycoside: no = 0, yes = 1 l__l **neo_ne198**

Other: no = 0, yes = 1 l__l **neo_ne199**

If yes, please specify: …………………………………………………………………….…………… **neo_ne200**

**Total duration of treatment** l__l__l j **neo_ne201**

Secondary infections (> 72h of life) during neonatal management

| ***infants in the common arm alive at D3*** | | |
| --- | --- | --- |
| **Bacteremia after 72h** | **neo_infection_bacteriemie** | 0: no secondary infection |
|  |  | 1: probable/possible secondary infection (antibiotherapy started after 72h and duration ≥ 5 days) |
|  |  | 2: proven bacteremia: blood culture + after 72h |
| **Secondary infection after 72h** | **neo_infection_LOS** | 0: no secondary infection |
|  |  | 1: probable/possible secondary infection (antibiotherapy started after 72h and duration ≥ 5 days) |
|  |  | 2: proven secondary infection; location = blood culture, trachea, urine, CSF or osteoarticular |
| **Infectious event after 72h (including conjunctivitis, skin involvement...)** | **neo_infection_secondaire** | 0: no secondary infection |
|  |  | 1: probable/possible secondary infection (antibiotherapy started after 72h and duration ≥ 5 days) |
|  |  | 2: proven, any location (excluding central catheter culture) |

**Secondary infection:** no = 0, yes = 1 l__l **neo_ne202**

If yes, number of infectious episodes l__l **neo_ne203**

***1^st^ secondary infectious episode:***

**Start date of the infectious episode**  l__l__l/ l__l__l /l__l__l__l__l **neo_ne204**

***Age at start of the infectious episode (in days) neo_ne204b***

**Clinical infection** (= anti-infection treatment ≥ 5 days): no = 0, yes = 1 l__l **neo_ne207**

**Infection confirmed by microbiology:** no = 0, yes = 1 l__l **neo_ne208**

**Bacteria**  l__l **neo_ne209**

***d_ne209f***

0: Unidentified

1: Coagulase-negative Staphylococcus*

2: Staphylococcus aureus

3: Enterococcus

4: Gram-negative bacillus (E. coli…)

If 4, please specify: ………………………………………………………….…………… **neo_ne210**

**2 blood cultures*

**Virus:** no = 0, yes = 1 l__l **neo_ne211**

If yes, please specify l__l **neo_ne212**

***d_ne212f***

1: RSV/adenovirus

2: Rotavirus = 2

3: Other

**Fungal infection:** no = 0, yes = 1 l__l **neo_ne213**

If yes, please specify: …………………………………………………………………………………. **neo_ne214**

**Resistant bacteria:** no = 0, yes = 1 l__l **neo_ne215**

If yes, please specify l__l **neo_ne216**

***d_ne216f***

1: Methicillin-resistant

2: Vancomycin-resistant

3: Multi-resistant

If multi-resistant, please specify: …………………………………………………… **neo_ne217**

**Location:**

Bloodstream (bacteremia): no = 0, yes = 1 l__l **neo_ne218**

Trachea (in intubated infant): no = 0, yes = 1 l__l **neo_ne219**

Local skin/soft tissue infection: no = 0, yes 1 l__l **neo_ne220**

Gastric fluid: no = 0, yes = 1 l__l **neo_ne221**

Conjunctivitis: no = 0, yes = 1 l__l **neo_ne222**

Urinary: no = 0, yes = 1 l__l **neo_ne223**

Cerebrospinal fluid: no = 0, yes = 1 l__l **neo_ne224**

Osteoarticular: no = 0, yes = 1 l__l **neo_ne225**

Catheter (positive culture): no = 0, yes = 1 l__l **neo_ne226**

Other: no = 0, yes = 1 l__l **neo_ne227**

If other, please specify: ………………………………………………………………………….……. **neo_ne228**

**At the time of infection, the infant had:**

A central catheter: no = 0, yes = 1 l__l **neo_ne229**

A peripheral venous line: no = 0, yes = 1 l__l **neo_ne230**

A urinary catheter: no = 0, yes = 1 l__l **neo_ne231**

Mechanical ventilation: no = 0, yes = 1 l__l **neo_ne232**

Undergone surgery ≤ 5 days previously: no = 0, yes = 1 l__l **neo_ne233**

**Highest C-reactive protein (CRP) value during the episode** l__l__l__l mg/l **neo_ne234**

**Procalcitonin (PCT) measured during the episode**: no = 0, yes = 1 l__l **neo_ne235**

If yes, highest PCT value during the episode l__l__l, l__l ng/ml **neo_ne236**

**Anti-infection treatment:**

C3G: no = 0, yes = 1 l__l **neo_ne237**

Aminoglycoside: no = 0, yes = 1 l__l **neo_ne238**

Vancomycin: no = 0, yes = 1 l__l **neo_ne239**

Carbapenem: no = 0, yes = 1 l__l **neo_ne240**

Fluconazole: no = 0, yes = 1 l__l **neo_ne241**

Other: no = 0, yes = 1 l__l **neo_ne242**

If yes, please specify: ………………………………………………………………………………… **neo_ne243**

**Duration of treatment (days)** l__l__l j **neo_ne244**

***2^nd^ secondary infectious episode:***

**Start date of the infectious episode** l__l__l/ l__l__l /l__l__l__l__l **neo_ne245**

***Age at start of the infectious episode (in days) neo_ne245b***

**Clinical infection** (= anti-infection treatment ≥ 5 days): no = 0, yes = 1 l__l **neo_ne248**

**Infection confirmed by microbiology:** no = 0, yes = 1 l__l **neo_ne249**

**Bacteria**  l__l **neo_ne250**

***d_ne209f***

0: Unidentified

1: Coagulase-negative Staphylococcus*

2: Staphylococcus aureus

3: Enterococcus

4: Gram-negative bacillus (E. coli…)

**2 blood cultures*

If 4, please specify: ……………………………………………………….……………… **neo_ne251**

**Virus:** no = 0, yes = 1 l__l **neo_ne252**

If yes, please specify  ***d_ne212f***

1: RSV/adenovirus

2: Rotavirus = 2

3: Other

**Fungal infection**: no = 0, yes = 1 l__l **neo_ne254**

If yes, please specify: ………………………………………………………………………………… **neo_ne255**

**Resistant bacteria:** no = 0, yes = 1 l__l **neo_ne256**

If yes, please specify l__l **neo_ne257**

***d_ne216f***

1: Methicillin-resistant

2: Vancomycin-resistant

3: Multi-resistant

If multi-resistant, please specify: …………………………………………….…………… **neo_ne258**

**Location:**

Bloodstream (bacteremia): no = 0, yes = 1 l__l **neo_ne259**

Trachea (in intubated infant): no = 0, yes = 1 l__l **neo_ne260**

Local skin/soft tissue infection: no = 0, yes 1 l__l **neo_ne261**

Gastric fluid: no = 0, yes = 1 l__l **neo_ne262**

Conjunctivitis: no = 0, yes = 1 l__l **neo_ne263**

Urinary: no = 0, yes = 1 l__l **neo_ne264**

Cerebrospinal fluid: no = 0, yes = 1 l__l **neo_ne265**

Osteoarticular: no = 0, yes = 1 l__l **neo_ne266**

Catheter (positive culture): no = 0, yes = 1 l__l **neo_ne267**

Other: no = 0, yes = 1 l__l **neo_ne268**

If other, please specify: ……………………………………………………………………….……… **neo_ne269**

**At the time of the infection, the infant had:**

A central catheter: no = 0, yes = 1 l__l **neo_ne270**

A peripheral venous line: no = 0, yes = 1 l__l **neo_ne271**

A urinary catheter: no = 0, yes = 1 l__l **neo_ne272**

Mechanical ventilation: no = 0, yes = 1 l__l **neo_ne273**

Undergone surgery ≤ 5 days previously: no = 0, yes = 1 l__l **neo_ne274**

**Highest C-reactive protein (CRP) value during the episode**  l__l__l__l mg/l **neo_ne275**

**Procalcitonin (PCT) measured during the episode**: no = 0, yes = 1 l__l **neo_ne276**

If yes, highest PCT value during the episode l__l__l , l__l ng/ml **neo_ne277**

**Anti-infection treatment:**

C3G: no = 0, yes = 1 l__l **neo_ne278**

Aminoglycoside: no = 0, yes = 1 l__l **neo_ne279**

Vancomycin: no = 0, yes = 1 l__l **neo_ne280**

Carbapenem: no = 0, yes = 1 l__l **neo_ne281**

Fluconazole: no = 0, yes = 1 l__l **neo_ne282**

Other: no = 0, yes = 1 l__l **neo_ne283**

If yes, please specify: ………………………………………………………………………………… **neo_ne284**

**Duration of treatment** l__l__l j **neo_ne285**

***3^ème^ secondary infectious episode:***

**Start date of the infectious episode**  l__l__l/ l__l__l /l__l__l__l__l **neo_ne286**

***Age at start of the infectious episode (in days) neo_ne286b***

**Clinical infection** (= anti-infection treatment ≥ 5 days): no = 0, yes = 1 l__l **neo_ne289**

**Infection confirmed by microbiology:** no = 0, yes = 1 l__l **neo_ne290**

**Bacteria**  l__l **neo_ne291**

***d_ne209f***

0: Unidentified

1: Coagulase-negative Staphylococcus*

2: Staphylococcus aureus

3: Enterococcus

4: Gram-negative bacillus (E. coli…)

**2 blood cultures*

If 4, please specify: ……………………………………………………………………… **neo_ne292**

**Virus:** no = 0, yes = 1 l__l **neo_ne293**

If yes, please specify l__l **neo_ne294**

***d_ne212f***

1: RSV/adenovirus

2: Rotavirus = 2

3: Other

**Fungal infection**: no = 0, yes = 1 l__l **neo_ne295**

If yes, please specify: …………………………………………………………………………………. **neo_ne296**

**Resistant bacteria:** no = 0, yes = 1 l__l **neo_ne297**

If yes, please specify l__l **neo_ne298**

***d_ne216f***

1: Methicillin-resistant

2: Vancomycin-resistant

3: Multi-resistant

If multi-resistant, please specify: …………………………………………………….…… **neo_ne299**

**Location:**

Bloodstream (bacteremia): no = 0, yes = 1 l__l **neo_ne300**

Trachea (in intubated infant): no = 0, yes = 1 l__l **neo_ne301**

Local skin/soft tissue infection: no = 0, yes 1 l__l **neo_ne302**

Gastric fluid: no = 0, yes = 1 l__l **neo_ne303**

Conjunctivitis: no = 0, yes = 1 l__l **neo_ne304**

Urinary: no = 0, yes = 1 l__l **neo_ne305**

Cerebrospinal fluid: no = 0, yes = 1 l__l **neo_ne306**

Osteoarticular: no = 0, yes = 1 l__l **neo_ne307**

Catheter (positive culture): no = 0, yes = 1 l__l **neo_ne308**

Other: no = 0, yes = 1 l__l **neo_ne309**

If other, please specify: ……………………………………………………………………….……… **neo_ne310**

**At the time of the infection, the infant had:**

A central catheter: no = 0, yes = 1 l__l **neo_ne311**

A peripheral venous line: no = 0, yes = 1 l__l **neo_ne312**

A urinary catheter: no = 0, yes = 1 l__l **neo_ne313**

Mechanical ventilation: no = 0, yes = 1 l__l **neo_ne314**

Undergone surgery ≤ 5 days previously: no = 0, yes = 1 l__l **neo_ne315**

**Highest C-reactive protein (CRP) value during the episode**  l__l__l__l mg/l **neo_ne316**

**Procalcitonin (PCT) measured during the episode**: no = 0, yes = 1 l__l **neo_ne317**

If yes, highest PCT value during the episode l__l__l , l__l ng/ml **neo_ne318**

**Anti-infection treatment:**

C3G: no = 0, yes = 1 l__l **neo_ne319**

Aminoglycoside: no = 0, yes = 1 l__l **neo_ne320**

Vancomycin: no = 0, yes = 1 l__l **neo_ne321**

Carbapenem: no = 0, yes = 1 l__l **neo_ne322**

Fluconazole: no = 0, yes = 1 l__l **neo_ne323**

Other: no = 0, yes = 1 l__l **neo_ne324**

If yes, please specify:………………………………………………………………………….……… **neo_ne325**

**Duration of treatment** l__l__l j **neo_ne326**

**Hemodynamics**

***During the first 72 hours of life:***

**Lowest mean arterial pressure (MAP) recorded during this period** l__l__l mmHg **neo_ne327**

**Treatment to improve hemodynamics:** no = 0, yes = 1 l__l **neo_ne328**

If yes,

Vascular filling during this period: no = 0, yes = 1 l__l **neo_ne329**

If yes, cumulative volume used during this period l__l__l__l ml/kg **neo_ne330**

Use of catecholamines during this period: no = 0, yes = 1 l__l **neo_ne331**

If yes, please specify:

Dopamine: no = 0, yes = 1 l__l **neo_ne332**

Dobutamine: no = 0, yes = 1 l__l **neo_ne333**

Noradrenaline: no = 0, yes = 1 l__l **neo_ne334**

Use of corticosteroids to improve hemodynamics during this period: no = 0, yes =1 l__l **neo_ne335**

Reason for treatment  l__l **neo_ne336**

***d_ne336f***

1: Isolated low arterial pressure

2: Combination of clinical signs suggesting inadequate hemodynamics

3: Ultrasound criteria alone

4: Ultrasound and clinical criteria

5: Other decisive criteria (NIRS, lactates, …)

**Cardiac ultrasound to assess hemodynamics during this period:** no = 0, yes = 1 l__l **neo_ne337**

***After the first 72 hours of life:***

**Treatment to improve hemodynamics:** no = 0, yes = 1 l__l **neo_ne338**

If yes,

Vascular filling: no = 0, yes = 1 l__l **neo_ne339**

Use of catecholamines: no = 0, yes = 1 l__l **neo_ne340**

Use of corticosteroids to improve hemodynamics: no = 0, yes = 1 l__l **neo_ne341**

***Management of patent ductus arteriosus:***

**Ultrasound examination of ductus arteriosus:** no = 0, yes = 1 l__l **neo_ne342**

If yes,

**Date of first examination**  l__l__l/ l__l__l /l__l__l__l__l **neo_ne343**

***Age at first examination (in days) neo_ne343b***

**Indication:**

Systematic investigation: no = 0, yes = 1 l__l **neo_ne346**

Based on clinical signs: no = 0, yes = 1 l__l **neo_ne347**

**Were the following criteria observed on ultrasound:**

Size of ductus arteriosus > 1.5 mm/kg: no = 0, yes = 1 l__l **neo_ne348**

Tubular appearance of ductal flow: no = 0, yes = 1 l__l **neo_ne349**

Increased pulmonary flow (mean velocity > 40 cm/sec, telediastolic velocity > 20 cm/sec):

no = 0, yes = 1 l__l **neo_ne350**

Low systemic flow (superior vena cava flow < 40 ml/kg/mn, no flow or reversed flow on cerebral/renal/mesenteric Doppler):  no = 0, yes = 1 l__l **neo_ne351**

**Most recent ultrasound of the ductus arteriosus during stay in NICU:**

Date l__l__l/ l__l__l /l__l__l__l__l neo_ne352

*Age (in days) neo_ne352b*

The ductus was: open = 1, closed = 2 l__l **neo_ne355**

***d_ne355f***

**Treatment of patent ductus arteriosus with NSAIDs:** no = 0, yes = 1 l__l **neo_ne356**

If yes,

**Treatment start date**  l__l__l/ l__l__l /l__l__l__l__l **neo_ne357**

***Age at start of treatment (in days) neo_ne357b***

**Indication** l__l **neo_ne360**

***d_ne360f***

1: Preventive

2: Curative based on ultrasound criteria only

3: Curative based on clinical criteria only

4: Curative based on clinical and ultrasound criteria

**Start date of 2^nd^ treatment course** l__l__l/ l__l__l /l__l__l__l__l **neo_ne361**

***Age at start of 2^nd^ course (in days) neo_ne361b***

**Indication** l__l **neo_ne364**

***d_ne364f***

1: Failure of 1st course

2: Reopening of ductus arteriosus (e.g. post sepsis)

**Surgical treatment of patent ductus arteriosus:** no = 0, yes = 1 l__l **neo_ne365**

If yes,

**Date of surgery** l__l__l/ l__l__l /l__l__l__l__l **neo_ne366**

***Age at surgery (in days) neo_ne366b***

**Indication**  l__l **neo_ne369**

***d_ne369f***

1: Primary intention (including if contraindication to medical treatment)

2: After failure of medical treatment

**Which of the following ultrasound criteria contributed to therapeutic decision-making (pharmacological or surgical treatment of ductus arteriosus):**

Size of ductus arteriosus > 1.5 mm/kg: no = 0, yes = 1 l__l **neo_ne370**

Tubular appearance of ductal flow: no = 0, yes = 1 l__l **neo_ne371**

Increased pulmonary flow (mean velocity > 40 cm/s, telediastolic velocity > 20 cm/s):

no = 0, yes = 1 l__l **neo_ne372**

Low systemic flow (superior vena cava flow < 40 ml/kg/mn, no flow or reverse flow on cerebral/renal/mesenteric Doppler): no = 0, yes = 1 l__l **neo_ne373**

Other: no = 0, yes = 1 l__l **neo_ne374**

**Developmental care**

**Summary at D7**

***Material and physical hospital environment:***

**Room at D7** l__l **neo_nf1**

***d_nf1f***

1: Single room

2: Two-bed room

3: Three beds

4: Four or more beds

5: Kangaroo unit (with the mother)

**If the infant is in a crib, use of an incubator cover or blanket to reduce light exposure at D7** l__l **neo_nf2**

***d_nf2f***

0: No

1: Permanent (except possibly during care)

2: Intermittent

3: Don’t know

***Human environment:***

**Skin-to-skin contact since birth (D0 to D7)**: no = 0, yes = 1, don’t know = 2 l__l **neo_nf3**

If yes,

date of first skin-to-skin contact l__l__l/ l__l__l /l__l__l__l__l **neo_nf4**

*Age at first skin-to-skin-contact (in days)* ***neo_nf4b***

If no, please give the main reason l__l **neo_nf7**

***d_nf7f***

1: Team’s choice

2: Lack of availability of care team

3: Parental anxiety

4: Parents not available

5: Infant’s unstable condition

6: Other

If other, please give details: ……………………………………………………………………. **neo_nf8**

| **Practice of skin-to-skin contact during the first week of life** *(For infants exiting the common arm alive)* | **neo_peau_a_peau** | 1 = skin-to-skin started between D0 and D3 2 = skin-to-skin started between D4 and D7 3 = no skin-to-skin between D0 and D7 |
| --- | --- | --- |
| **If no skin-to-skin contact, what were the reasons** *(For infants exiting the common arm alive)* | **neo_raison_pas_pap** | 1 = team-related reasons (team’s choice, lack of availability of care team, skin-to-skin was not offered, umbilical venous catheter, isolated infant) 2 = parent-related reasons (parental anxiety, parents not available, mother did not wish it, mother hospitalized) 3 = infant-related reasons (Infant’s unstable condition, infant’s condition did not allow it, other illness of the infant) 4 = other reason |

***Pain and discomfort:***

**Assessment of the infant’s pain or discomfort at least once on a standardized scale when not receiving care (between D0 and D7):** no = 0, yes = 1, don’t know = 2 l__l **neo_nf9**

If yes,

Highest EDIN score (out of 15) l__l__l /15 **neo_nf10**

Other evaluation method: no = 0, yes = 1 l__l **neo_nf11**

If yes, please give details: ……………………………………………………………………… … **neo_nf12**

Highest score  l__l__l **neo_nf13**

**Assessment of the infant’s pain or discomfort at least once on a standardized scale when receiving care (between D0 and D7):** no = 0, yes = 1, don’t know = 2 l__l **neo_nf14**

If yes,

Highest DAN score during care (out of 10) l__l__l /10 **neo_nf15**

Other evaluation method: no = 0, yes = 1 l__l **neo_nf16**

If yes, please give details: ………………………………………………………………………..… **neo_nf17**

Highest score  l__l__l **neo_nf18**

***Feeding:***

**Parents’ participation in feeding***: no = 0, yes = 1, don’t know = 2 l__l **neo_nf19**

**Holding and wrapping the infant, skin-to-skin during feeding, bottle teat given during tube feeding*

**Infant put to the breast** l__l **neo_nf20**

***d_nf20f***

0: No

1: Yes for contact

2: Yes for non-nutritive sucking

3: Yes for nutritive sucking

4: Don’t know

If yes,

date when infant was first put to the breast l__l__l/ l__l__l /l__l__l__l__l **neo_nf21**

*Age when infant was first put to the breast (in days)* ***neo_nf21b***

**Has the mother chosen to breastfeed the baby herself:**

no = 0, yes =1, don’t know = 2 l__l **neo_nf24**

**Summary at D28**

***Material and physical hospital environment:***

**Room at D28** l__l **neo_nf25**

***d_nf1f***

1: Single room

2: Two-bed room

3: Three beds

4: Four or more beds

5: Kangaroo unit (with the mother)

**If the infant is in a crib, use of an incubator cover or blanket to reduce light exposure at D28**

l__l **neo_nf26**

***d_nf2f***

0: No

1: Permanent (except possibly during care)

2: Intermittent

3: Don’t know

***Pain and discomfort:***

**Assessment of the infant’s pain or discomfort at least once on a standardized scale when not receiving care (between D21 and D28)**:

no = 0, yes = 1, don’t know = 2 l__l **neo_nf27**

If yes,

Highest EDIN score (out of 15) l__l__l /15 **neo_nf28**

Other evaluation method**:** no = 0, yes = 1 l__l **neo_nf29**

If yes, please give details: …………………………………………………………………..……… **neo_nf30**

Highest score  _ l__l__l **neo_nf31**

**Assessment of the infant’s pain or discomfort at least once on a standardized scale when receiving care (between D21 and D28):**

no = 0, yes = 1, don’t know = 2 l__l **neo_nf32**

If yes,

Highest DAN score during care (out of 10) l__l__l /10 **neo_nf33**

Other evaluation method**:** no = 0, yes = 1 l__l **neo_nf34**

If yes, please give details: ………………………………………………………………………… **neo_nf35**

Highest score  l__l__l **neo_nf36**

***Feeding:***

**Parents’ participation in feeding*:** no = 0, yes = 1, don’t know = 2 l__l **neo_nf37**

**Holding and wrapping the infant, skin-to-skin during feeding, bottle teat given during tube feeding*

**Infant put to the breast** l__l **neo_nf38**

***d_nf20f***

0: No

1: Yes for contact

2: Yes for non-nutritive sucking

3: Yes for nutritive sucking

4: Don’t know

**Has the mother chosen to breastfeed the baby herself:**

no = 0, yes =1, don’t know = 2 l__l **neo_nf39**

**Summary since birth**

**Has the infant received a formal and individualized developmental care program since birth**: no = 0, Bullinger method = 1, NIDCAP® = 2 l__l **neo_nf40**

If NIDCAP®,

Number of observations between birth and 36 WGA l__l__l **neo_nf41**

Date of first observation l__l__l/ l__l__l /l__l__l__l__l **neo_nf42**

*Gestational age at first observation (in days)* ***neo_nf42b***

**How many nights has the infant spent in the room with his/her mother before discharge home?**  l__l__l **neo_nf45**

**Has the infant received oral stimulation since birth**: no = 0, yes = 1 l__l **neo_nf46**

**Nutrition**

**Summary at D3**

**Current weight**  l__l__l__l__l g **neo_ng1**

**Weight used to calculate parenteral intake at D3** l__l__l__l__l g **neo_ng2**

**Birth length**  l__l__l cm **neo_ng3**

**Head circumference at birth**  l__l__l cm **neo_pcnce**

***Enteral intake prescribed:***

**Start date of enteral feeding if started during these 3 days** l__...__l **neo_ng5**

***Age*** ***at start of enteral feeding if started during these 3 days (in days) neo_ng5b***

**Total 24h enteral intake**  l__l__l__l ml **neo_ng8**

Type of milk n°1 *(cf appendix - Types of milk - Guide to completing the neonatal questionnaire)*  l__l__l l__l l__l **neo_ng9**

Amount prescribed for 24h l__l__l__l ml **neo_ng10**

Type of milk n°2 *(cf appendix - Types of milk - Guide to completing the neonatal questionnaire)* l__l__l l__l l__l **neo_ng11**

Amount prescribed for 24h l__l__l__l ml **neo_ng12**

***Parenteral intake prescribed:***

**Total 24h parenteral intake, including fat volume** l__l__l__l ml/kg/j **neo_ng13**

***Total intake prescribed (enteral + parenteral):***

**Total 24h intake**  l__l__l__l__l ml/kg/j **neo_ng14**

Protein intake l__l__l__l, l__l g/kg/j **neo_ng15**

Carbohydrate intake l__l__l__l, l__l g/kg/j **neo_ng16**

Fat intake l__l__l__l, l__l g/kg/j **neo_ng17**

**Summary at D7**

**Current weight**  l__l__l__l__l g **neo_ng18**

**Weight used to calculate parenteral intake at D7**  l__l__l__l__l g **neo_ng19**

**Length at about this time**  l__l__l cm **neo_ng20**

**Head circumference at about this time** l__l__l cm **neo_ng21**

***Enteral intake prescribed:***

**Enteral intake (including minimal feeding)**: no = 0, yes = 1 l__l **neo_ng22**

**Start date of enteral feeding if started between D3 and D7** l__...__l **neo_ng23**

***Age at start of enteral feeding if started between D3 and D7 (in days) neo_ng23b***

**Total 24h enteral intake** l__l__l__l ml **neo_ng26**

Type of milk n°1 *(cf appendix - Types of milk - Guide to completing the neonatal questionnaire)*  l__l__l l__l l__l **neo_ng27**

Amount prescribed for 24h __________________________________________ l__l__l__l ml **neo_ng28**

Type of milk n°2 *(cf appendix - Types of milk - Guide to completing the neonatal questionnaire)*  l__l__l l__l l__l **neo_ng29**

Amount prescribed for 24h ___________________________________________ l__l__l__l ml **neo_ng30**

***Parenteral intake prescribed:***

**Total 24h parenteral intake**  l__l__l__l ml/kg/j **neo_ng31**

***Total intake prescribed (enteral + parenteral):***

**Total 24h intake**  l__l__l__l__l ml/kg/j **neo_ng32**

Protein intake l__l__l__l, l__l g/kg/j **neo_ng33**

Carbohydrate intake l__l__l__l, l__l g/kg/j **neo_ng34**

Fat intake l__l__l__l, l__l g/kg/j **neo_ng35**

**Did you use insulin in the first 7 days:** no = 0, yes = 1 l__l **neo_ng36**

If yes, main indication  l__l **neo_ng37**

***d_ng37f***

1: Glycemia ≥ 8.5 mmol/l and < 10 mmol/l

2: Glycemia ≥ 10 mmol/l and < 15 mmol/l

3: Glycemia ≥ 15 mmol/l

4: Glycosuria

5: Other

If other, please give details: **neo_ng38**

**Highest glycemia level observed in the first 7 days**  l__l__l, l__l mmol/l **neo_ng39**

**Gastrointestinal transit considered normal (at least one stool a day):** no = 0, yes = 1 l__l **neo_ng40**

**Summary at D28**

**Current weight**  l__l__l__l__l g **neo_ng41**

**Length at about this time**  l__l__l cm **neo_ng42**

**Head circumference at about this time**  l__l__l cm **neo_ng43**

**Mean arterial pressure (MAP)* recorded**  l__l__l mmHg **neo_ng44**

**First MAP measurement of the day*

*Enteral intake prescribed:*

**Total 24h enteral intake** l__l__l__l ml **neo_ng45**

**Is the infant still tube-fed:** no = 0, yes = 1 l__l **neo_ng46**

If no, end date of tube feeding l__l__l/ l__l__l /l__l__l__l__l **neo_ng47**

*Age at end of tube feeding (in days)* ***neo_ng47b***

**Does the infant feed directly at the breast:** no = 0, yes = 1 l__l **neo_ng50**

If yes, does this feed form the entire enteral intake: no = 0, yes = 1 l__l **neo_ng51**

**If the infant receives feeds instead of or as a complement to breastfeeding, specify with what milks** (including milk from a milk bank, individualized maternal milk given outside of breastfeeding):

Type of milk n°1 *(cf appendix - Types of milk - Guide to completing the neonatal questionnaire)* l__l__l l__l l__l **neo_ng52**

Amount prescribed for 24h l__l__l__l ml **neo_ng53**

Type of milk n°2 *(cf appendix - Types of milk - Guide to completing the neonatal questionnaire)* l__l__l l__l l__l **neo_ng54**

Amount prescribed for 24h l__l__l__l ml **neo_ng55**

***Parenteral intake prescribed:***

**Total 24h parenteral intake**  l__l__l__l ml/kg/j **neo_ng56**

***Total intake prescribed (enteral + parenteral):***

**Total 24h intake**  l__l__l__l__l ml/kg/j **neo_ng57**

Protein intake l__l__l__l, l__l g/kg/j **neo_ng58**

Carbohydrate intake l__l__l__l, l__l g/kg/j **neo_ng59**

Fat intake l__l__l__l, l__l g/kg/j **neo_ng60**

**If the infant is no longer fed parenterally, end date of infusion**  l__...__l **neo_ng61**

***Age at end of infusion (in days) __________________________________________________ neo_ng61b***

**Did you use insulin between D8 and D28**: no = 0, yes = 1 l__l **neo_ng64**

**Summary at 36 WGA**

**Weight at about this time** l__l__l__l__l g **neo_ng65**

**Length at about this time** l__l__l cm **neo_ng66**

**Head circumference at about this time**  l__l__l cm **neo_ng67**

***Enteral intake prescribed:***

**Is the infant still tube-fed:** no = 0, yes = 1 l__l **neo_ng68**

If no and if applicable, end date of tube feeding l__...__l **neo_ng69**

*Age at end of tube feeding (in days)* ***neo_ng69b***

**Does the infant feed directly at the breast:** no = 0, yes = 1 l__l **neo_ng72**

If yes, does this feed form the entire enteral intake: no = 0, yes = 1 l__l **neo_ng73**

**If the infant receives feeds instead of or as a complement to breastfeeding, specify with what milks** (including milk from a milk bank, individualized maternal milk given outside of breastfeeding):

Type of milk n°1 *(cf appendix - Types of milk - Guide to completing the neonatal questionnaire)* l__l__l l__l l__l **neo_ng74**

Amount prescribed for 24h l__l__l__l ml **neo_ng75**

Type of milk n°2 *(cf appendix - Types of milk - Guide to completing the neonatal questionnaire)* l__l__l l__l l__l **neo_ng76**

Amount prescribed for 24h l__l__l__l ml **neo_ng77**

***Parenteral intake prescribed:***

**Total 24h parenteral intake**  l__l__l__l ml/kg/j **neo_ng78**

***Total intake prescribed (enteral + parenteral):***

**Total 24h intake**  l__l__l__l ml/kg/j **neo_ng79**

**Does the infant still have a central catheter:** no = 0, yes = 1 l__l **neo_ng80**

If no, date of removal of last central catheter l__l__l/ l__l__l /l__l__l__l__l **neo_ng81**

*Age at removal of last central catheter (in days)*  ***neo_ng81b***

**Summary since birth**

***Central catheters:***

**Since birth, how many central catheters have been inserted**  l__l **neo_ng84**

**Central catheter-related adverse event:** no = 0, yes = 1 l__l **neo_ng85**

If yes:

Vascular thrombosis: no = 0, yes = 1 l__l **neo_ng86**

If yes, treatment: no = 0, heparin = 1, fibrinolysis = 2 l__l **neo_ng87**

***d_ng87f***

Pleural effusion: no = 0, yes = 1 l__l **neo_ng88**

Peritoneal effusion: no = 0, yes = 1 l__l **neo_ng89**

Tamponade: no = 0, yes = 1 l__l **neo_ng90**

Other: no = 0, yes = 1 l__l **neo_ng90a**

If yes, please give details: **neo_ng90b**

**How many central catheter-related complications were observed**  l__l **neo_ng91**

Number of intravascular thrombosis events l__l **neo_ng92**

Number of blocked catheter withdrawals l__l **neo_ng93**

Number of pericardial effusion events l__l **neo_ng94**

Other: no = 0, yes = 1 l__l **neo_ng95**

If yes, please give details: ……………………………………………………………………….… **neo_ng96**

***Gastrointestinal disturbances/necrotizing enterocolitis:***

**Spontaneous intestinal perforation:** no = 0, yes = 1 l__l **neo_ng97**

If yes,

Date l__l__l/ l__l__l /l__l__l__l__l **neo_ng98**

*Age (in days)* ***neo_ng98b***

Surgery: no = 0, yes = 1 l__l **neo_ng101**

If yes, date of surgery l__l__l/ l__l__l /l__l__l__l__l **neo_ng102**

*Age at surgery (in days)* ***neo_ng102b***

**Necrotizing enterocolitis:** no = 0, yes = 1 l__l **neo_ng105**

If yes,

Clinical Bell stage II *: no = 0, yes = 1 l__l **neo_ng106**

**silent flatulence ± green residuals ± abdominal guarding ± appendiceal mass of right iliac fossa ± abdominal wall inflammation*

If yes, date l__l__l/ l__l__l /l__l__l__l__l **neo_ng107**

*Age (in days)* ***neo_ng107b***

Radiological Bell stage II **: no = 0, yes = 1 l__l **neo_ng110**

*** intestinal dilation ± intestinal and/or portal pneumatosis ± peritoneal effusion*

If yes, date l__l__l/ l__l__l /l__l__l__l__l **neo_ng111**

*Age (in days)* ***neo_ng111b***

Clinical Bell stage III *: no = 0, yes = 1 l__l **neo_ng114**

**hypotension, oligouria, hydroelectrolyte imbalance ± mixed acidosis ± neutropenia ± DIC ± ARDS, voluminous painful flatulence with induration*

If yes, date l__l__l/ l__l__l /l__l__l__l__l **neo_ng115**

*Age (in days)*  ***neo_ng115b***

Radiological Bell stage III **: no = 0, yes = 1 l__l **neo_ng118**

***localized pneumoperitoneum or pneumoperitoneum of the abdominal cavity*

If yes, date l__l__l/ l__l__l /l__l__l__l__l **neo_ng119**

*Age (in days)* ***neo_ng119b***

**Complications**

*In addition to the clinical events already recorded in this questionnaire, has the infant experienced the following complications related to care:*

**Cutaneous event:** no = 0, yes = 1 l__l **neo_nh1**

If yes:

Cutaneous necrosis related to ventilation device (CPAP or endotracheal tube): no = 0, yes = 1 l__l **neo_nh2**

Cutaneous necrosis related to peripheral venous line: no = 0, yes = 1 l__l **neo_nh3**

Lesions due to chemicals/electrodes/dressings: no = 0, yes = 1 l__l **neo_nh4**

**Medication error:** no = 0, yes = 1 l__l **neo_nh5**

If yes:

Prescribing error: no = 0, yes = 1 l__l  **neo_nh6**

Product error: no = 0, yes = 1 l__l **neo_nh7**

Preparation error: no = 0, yes = 1 l__l **neo_nh8**

Administration error: no = 0, yes = 1 l__l **neo_nh9**

**Adverse drug effect:** no = 0, yes = 1 l__l **neo_nh10**

**Other iatrogenic condition(s):** no = 0, yes = 1 l__l **neo_nh11**

If yes, please give details:………………………………………………………………………….… **neo_nh12**

**Congenital anomalies**

**Congenital anomalies:** no = 0, yes = 1 l__l **neo_ni1**

If yes, final description of the congenital anomalies (including syndromes); please describe each anomaly as fully as possible:

1^st^ malformation: …………………………………………………………………………………………. **neo_ni2**

2^nd^ malformation: ………………………………………………………………………………………… **neo_ni3**

3^rd^ malformation: ………………………………………………………………………………………… **neo_ni4**

4^th^ malformation: ………………………………………………………………………………………… **neo_ni5**

5^th^ malformation: ………………………………………………………………………………………… **neo_ni6**

***ICD10 coding for the congenital anomalies of variables NEO_NI2 to NEO_NI6 and ENFANT_MP5 to ENFANT_MP7:***

***|__|__|__|__|__| NEO_CIM10_MALF1***

***|__|__|__|__|__| NEO_CIM10_MALF2***

***|__|__|__|__|__| NEO_CIM10_MALF3***

***|__|__|__|__|__| NEO_CIM10_MALF4***

***|__|__|__|__|__| NEO_CIM10_MALF5***

**Summary of malformations no malformation=0, at least 1 major malformation=1, a single minor malformation =2, doubtful malformation or criterion of gravity =9 l__I ENFANT_MALFO_MAJEUR**

*(relates to infants entered in the common arm - variable N. Lelong, registry of congenital malformations*

**Retinopathy**

**Tests for retinopathy:** no = 0, yes = 1 l__l **neo_nj1**

If yes,

Using Retcam = 1, other method = 2 l__l **neo_nj2**

***d_nj2f***

Date of 1st screen l__l__l/ l__l__l /l__l__l__l__l **neo_nj3**

*Age at 1st screen (in days)* ***neo_nj3b***

Date of last screen l__l__l/ l__l__l /l__l__l__l__l **neo_nj6**

*Age at last screen (in days)* ***neo_nj6b***

Most severe stage of retinopathy l__l **neo_nj9**

Specify: right eye = 1, left eye = 2, both = 3 l__l **neo_nj10**

***d_nj10f***

**Treated retinopathy:** no = 0, laser = 1, cryotherapy = 2 l__l **neo_nj11**

***d_nj11f***

If treated: right eye = 1, left eye = 2, both = 3 l__l **neo_nj12**

***d_nj10f***

**Hearing screening**

**Was hearing screening done:** no = 0, yes = 1 l__l **neo_nk1**

If yes,

Otoacoustic emissions (OAEs): no = 0, yes = 1 l__l **neo_nk2**

If yes, test results l__l **neo_nk3**

***d_nk3f***

0: Normal

1: Abnormal right ear

2: Abnormal left ear

3: Both ears abnormal

Auditory evoked potentials (AEPs): no = 0, yes = 1 l__l **neo_nk4**

If yes, test results l__l **neo_nk5**

***d_nk3f***

0: Normal

1: Abnormal right ear

2: Abnormal left ear

3: Both ears abnormal

**Profound deafness** (> 90 dB): no = 0, right ear = 1, left ear= 2, both ears = 3 l__l **neo_nk6**

***d_nk6f***

**Vaccination**

**Vaccination started during hospital stay:** no = 0, yes = 1 l__l **neo_nv1**

If yes, please specify:

Diphtheria, tetanus, pertussis, Haemophilus influenzae: no = 0, yes = 1 l__l **neo_nv2**

Hepatitis B: no = 0, yes = 1 l__l **neo_nv3**

Pneumococcal: no = 0, yes = 1 l__l **neo_nv4**

BCG: no = 0, yes = 1 l__l **neo_nv5**

**Summary of the infant’s pathway**

*This part of the questionnaire aims to describe the infant’s hospital stays and transfers. A transfer means a change of facility (or geographical area).*

**Was the infant born in the facility where he/she was hospitalized just after birth?** no = 0, yes = 1 l__l **neo_nl1**

***If yes, continue to page 45 (Description of the infant’s stay in the 1^st^*** hospital facility)

| *Infants in the common arm* |  |  |
| --- | --- | --- |
| Facility where the infant spent the first consecutive 48h | **unit48** | Anonymized code number of the facility |
| Level of the facility where the infant spent the first consecutive 48h | **niveau_unit48** | 1 = level 1 facility  21 = level 2A facility  22 = level 2B facility  3 = level 3 facility  ***d_etabniv*** |
| Infant hospitalized in the same facility during the 1st week of life | **etab_J0J7** | 0 = no 1 = yes |
| If yes *(infants transferred in the first 48 h of life have a reference facility if they remained in the same facility until D7)* | | |
| Anonymized number of the reference facility where the infant was hospitalized during the 1st week of life | **num_etab_J0J7** | Anonymized code number of the facility |
| Level of the reference facility where the infant was hospitalized during the 1st week of life | **niveau_etab_J0J7** | 1 = level 1 facility  21 = level 2A facility  22 = level 2B facility  3 = level 3 facility  ***d_etabniv*** |

***If no, describe the infant’s initial transfer from the facility where he/she was born***

1. ***Initial transfer:***

*Journeys to and from the facility and another site for periods < 24h, e.g. for complementary investigations, are not to be counted as transfers*

**Neonatal facility where the infant was born: name of the facility, city/town, n° of the geographical department**

**in full** **enr_etabnaiss**

**FINESS number =** **enr_finessnaiss**

**Anonymized code number of the facility = enr_numetabnaiss**

**Level= enr_niveau2naiss**

**Date of discharge** l__l__l/ l__l__l /l__l__l__l__l **neo_nl5**

***Age at discharge (in days) neo_nl5b***

**Transport** l__l **neo_nl8**

***d_nl8f***

1: Emergency ambulance (road)

2: Emergency ambulance (helicopter)

3: Nurse-accompanied interhospital transfer

4: Other

If other, please give details: ……………………………………………………………… ….. **neo_nl9**

**Transfer to:** higher level* = 1, lower level = 2, other = 3 ______________________________ l__l **neo_nl10**

**Higher level = need for more care or specialized technical facilities*

***d_nl10f***

**Reason(s) for transfer:**

*Give all the reasons leading to transfer*

**Condition(s):**

Prematurity: no = 0, yes = 1 l__l **neo_nl11**

Intrauterine growth retardation (IUGR): no = 0, yes = 1 l__l **neo_nl12**

Respiratory distress: no = 0, yes = 1 l__l **neo_nl13**

Malformation(s): no = 0, yes = 1 l__l **neo_nl14**

**Need for complementary care:**

Surgery: no = 0, yes = 1 l__l **neo_nl15**

Need for parenteral feeding: no = 0, yes = 1 l__l **neo_nl16**

**Organizational reason(s):**

Lack of space: no = 0, yes = 1 l__l **neo_nl17**

**Other:** no = 0, yes = 1 l__l **neo_nl18**

If yes, please give details: **neo_nl19**

1. ***Stay in the 1^st^ hospital facility:***

**Name of discharging facility** in full **neo_nl20**

**City/town of discharging facility** in full **neo_nl21**

**N° of the geographical department of discharging facility** in full l__l__l **neo_nl22**

**FINESS number= neo_finess_etab1**

**Level= neo_niveau_etab1**

***d_etabniv***

***Anonymized code number of the facility neo_numetab1***

**Department to which admitted** l__l **neo_nl23**

***d_nl23***

1: Neonatal resuscitation unit

2: Neonatal intensive care unit

3: Neonatal medicine department

4: Other

If other, please give details: ………………………………………………………………… **neo_nl24**

**Date of admission to the department** l__l__l/ l__l__l /l__l__l__l__l **neo_nl25**

***Age at admission to the department (in days) neo_nl25b***

**Time of admission to the department**  l__l__l h l__l__l min **neo_nl28 neo_nl29**

***Age at admission to the first neonatal facility (in min) neo_nl29b***

**During the infant’s stay in this facility, was he/she hospitalized in:**

**Neonatal resuscitation unit:** no = 0, yes = 1 l__l **neo_nl30**

If yes,

Length of stay < 1 day: no = 0, yes = 1 l__l **neo_nl31**

If stay ≥ 1 day, total number of days in neonatal resuscitation unit l__l__l__l j **neo_nl32**

Number of stays in neonatal resuscitation unit l__l **neo_nl33**

**Neonatal intensive care unit:** no = 0, yes = 1 l__l **neo_nl34**

If yes, stay < 1 day: no = 0, yes = 1 l__l **neo_nl35**

If stay ≥ 1 day, total number of days in neonatal intensive care unit l__l__l__l j **neo_nl36**

**Neonatal medicine department:** no = 0, yes = 1 l__l **neo_nl37**

If yes, stay < 1 day: no = 0, yes = 1 l__l **neo_nl38**

If stay ≥ 1 day, total number of days in neonatal medicine department l__l__l__l j **neo_nl39**

**Other department(s)**: no = 0, yes = 1 l__l **neo_nl40**

If yes,

Other department 1, in full: ……………………………………………………………….………… **neo_nl41**

Total number of days in this department l__l__l__l j **neo_nl42**

Other department 2, in full: ………………………………………………………………………… . **neo_nl43**

Total number of days in this department l__l__l__l j **neo_nl44**

Other department 3, in full: ………………………………………………………….………………. **neo_nl45**

Total number of days in this department l__l__l__l j **neo_nl46**

**Date of discharge from the facility**  l__l__l/ l__l__l /l__l__l__l__l **neo_nl47**

***Age at discharge from the facility (days) neo_nl47b***

**Status at discharge:** alive = 1, dead = 2 l__l **neo_nl50**

***d_nl50f***

If alive, discharge to  l__l **neo_nl51**

***d_nl51f***

1: Home

2: Nursery or other residential care

3: Transfer to another facility*

** Journeys to and from the facility and another site for periods < 24h, e.g. for complementary investigations, are not to be counted as transfers*

4: Other

If 1 or 2, continue to page 60 (Infant’s condition at discharge home)

If other, please give details: …………………………………………………………………… **neo_nl52**

1. ***Transfer to a 2^nd^ hospital facility:***

**Date of discharge**  l__l__l/ l__l__l /l__l__l__l__l **neo_nl53**

***Age at discharge (in days) neo_nl53b***

**Discharging department**  l__l **neo_nl56**

***d_nl23f***

1: Neonatal resuscitation unit

2: Neonatal intensive care unit

3: Neonatal medicine department

4: Other

If other, please give details:…………………………………………………………………… **neo_nl57**

**Transport**  l__l **neo_nl58**

***d_nl8f***

1: Emergency ambulance (road)

2: Emergency ambulance (helicopter)

3: Nurse-accompanied interhospital transfer

4: Other

If other, please give details: ………………………………………………………………….. **neo_nl59**

**Transfer to:** higher level* = 1, lower level = 2, other = 3 l__l **neo_nl60**

**Higher level = need for more care or specialized technical facilities*

***d_nl10f***

**Reason(s) for transfer:**

*Give all the reasons leading to transfer*

**Condition(s):**

Prematurity: no = 0, yes = 1 l__l **neo_nl61**

Intrauterine growth retardation (IUGR): no = 0, yes = 1 l__l **neo_nl62**

Respiratory distress: no = 0, yes = 1 l__l **neo_nl63**

Malformation(s): no = 0, yes = 1 l__l **neo_nl64**

Worsening of a condition: no = 0, yes = 1 l__l **neo_nl65**

If yes, please specify:…………………………………………………………………………………….. **neo_nl66**

**Need for complementary care:**

Surgery: no = 0, yes = 1 l__l **neo_nl67**

Need for parenteral feeding: no = 0, yes = 1 l__l **neo_nl68**

**Organizational reason(s):**

Lack of space: no = 0, yes = 1 l__l **neo_nl69**

Transfer to hospital closer to home: no = 0, yes = 1 l__l **neo_nl70**

**Other**: no = 0, yes = 1 l__l **neo_nl71**

If yes, please specify: ……………………………………………………………………….…………… **neo_nl72**

***Stay in the 2^nd^ hospital facility:***

**Name of discharging facility** in full **neo_nl73**

**City/town of discharging facility** in full **neo_nl74**

**N° of the geographical department of discharging facility** in full l__l__l **neo_nl75**

**FINESS number= neo_finess_etab2**

**Level= neo_niveau_etab2**

***Anonymized code number of the facility neo_numetab2***

**Department to which admitted**  l__l **neo_nl76**

***d_nl23f***

1: Neonatal resuscitation unit

2: Neonatal intensive care unit

3: Neonatal medicine department

4: Other

If other, please give details: ……………………………………………………………….. **neo_nl77**

**Date of admission to the department** l__l__l/ l__l__l /l__l__l__l__l **neo_nl78**

***Age at admission to the department (in days) neo_nl78b***

**During the infant’s stay in this facility, was he/she hospitalized in:**

**Neonatal resuscitation unit**: no = 0, yes = 1 l__l **neo_nl81**

If yes,

Length of stay < 1 day: no = 0, yes = 1 l__l **neo_nl82**

If stay ≥ 1 day, total number of days in neonatal resuscitation unit l__l__l__l j **neo_nl83**

Number of stays in neonatal resuscitation unit l__l **neo_nl84**

**Neonatal intensive care unit**: no = 0, yes = 1 l__l **neo_nl85**

If yes, stay < 1 day: no = 0, yes = 1 l__l **neo_nl86**

If stay ≥ 1 day, total number of days in neonatal intensive care unit l__l__l__l j **neo_nl87**

**Neonatal medicine department:** no = 0, yes = 1 l__l **neo_nl88**

If yes, stay < 1 day: no = 0, yes = 1 l__l **neo_nl89**

If stay ≥ 1 day, total number of days in neonatal medicine department l__l__l__l j **neo_nl90**

**Other department(s)**: no = 0, yes = 1 l__l **neo_nl91**

If yes,

Other department 1, in full: ……………………………………………………………………….…. **neo_nl92**

Total number of days in this department l__l__l__l j **neo_nl93**

Other department 2, in full: …………………………………………………………………………. **neo_nl94**

Total number of days in this department l__l__l__l j **neo_nl95**

Other department 3, in full: ………………………………………………………………………….. **neo_nl96**

Total number of days in this department l__l__l__l j **neo_nl97**

**Date of discharge from the facility**  l__l__l/ l__l__l /l__l__l__l__l **neo_nl98**

***Age at discharge from the facility (days) neo_nl98b***

**Status at discharge:** alive = 1, dead = 2 l__l **neo_nl101**

***d_nl50f***

If alive, discharge to  l__l **neo_nl102**

***d_nl51f***

1: Home

2: Nursery or other residential care

3: Transfer to another facility*

**Journeys to and from the facility and another site for periods < 24h, e.g. for complementary investigations, are not to be counted as transfers*

4: Other

If 1 or 2, continue to page 60 (Infant’s condition at discharge home)

If other, please give details: ……………………………………………………………… **neo_nl103**

1. ***Transfer to a 3^rd^ hospital facility:***

**Name of discharging facility** in full **neo_nl104**

**City/town of discharging facility** in full **neo_nl105**

**N° of the geographical department of discharging facility** in full l__l__l **neo_nl106**

**Discharging department** _______________________________________________________ l__l **neo_nl110**

***d_nl23f***

1: Neonatal resuscitation unit

2: Neonatal intensive care unit

3: Neonatal medicine department

4: Other

If other, please specify:…………………………………………………………………..…… **neo_nl111**

**Transport** ___________________________________________________________________ l__l **neo_nl112**

***d_nl8f***

1: Emergency ambulance (road)

2: Emergency ambulance (helicopter)

3: Nurse-accompanied interhospital transfer

4: Other

If other, please specify: …………………………………………………………………….. **neo_nl113**

**Transfer to:** higher level* = 1, lower level = 2, other = 3 l__l **neo_nl114**

**Higher level = need for more care or specialized technical facilities*

***d_nl10f***

**Reason(s) for transfer:**

*Give all the reasons leading to transfer*

**Condition(s):**

Prematurity: no = 0, yes = 1 l__l **neo_nl115**

Intrauterine growth retardation (IUGR): no = 0, yes = 1 l__l **neo_nl116**

Respiratory distress: no = 0, yes = 1 l__l **neo_nl117**

Malformation(s): no = 0, yes = 1 l__l **neo_nl118**

Worsening of a condition: no = 0, yes = 1 l__l **neo_nl119**

If yes, please specify:……………………………………………………………………………….…. **neo_nl120**

**Need for complementary care:**

Surgery: no = 0, yes = 1 l__l **neo_nl121**

Need for parenteral feeding: no = 0, yes = 1 l__l **neo_nl122**

**Organizational reason(s):**

Lack of space: no = 0, yes = 1 l__l **neo_nl123**

Transfer to hospital closer to home: no = 0, yes = 1 l__l **neo_nl124**

**Other**: no = 0, yes = 1 l__l **neo_nl125**

If yes, please specify: ……………………………………………………………………………….… **neo_nl126**

1. ***Stay in the 3^rd^ hospital facility:***

**Name of discharging facility** in full **neo_nl127**

**City/town of discharging facility** in full **neo_nl128**

**N° of the geographical department of discharging facility** in full l__l__l **neo_nl129**

**FINESS number= neo_finess_etab3**

**Level= neo_niveau_etab3 *d_etabniv***

***Anonymized code number of the facility neo_numetab3***

**Department to which admitted**  l__l **neo_nl130**

***d_nl23f***

1: Neonatal resuscitation unit

2: Neonatal intensive care unit

3: Neonatal medicine department

4: Other

If other, please give details: …………………………………………………………………. **neo_nl131**

**Date of admission to the department** l__l__l/ l__l__l /l__l__l__l__l **neo_nl132**

***Age at admission to the department (in days) neo_nl132b***

**During the infant’s stay in this facility, was he/she hospitalized in:**

**Neonatal resuscitation unit**: no = 0, yes = 1 l__l **neo_nl135**

If yes,

Length of stay < 1 day: no = 0, yes = 1 l__l **neo_nl136**

If stay ≥ 1 day, total number of days in neonatal resuscitation unit l__l__l__l j **neo_nl137**

Number of stays in neonatal resuscitation unit l__l **neo_nl138**

**Neonatal intensive care unit**: no = 0, yes = 1 l__l **neo_nl139**

If yes, stay < 1 day: no = 0, yes = 1 l__l **neo_nl140**

If stay ≥ 1 day, total number of days in neonatal intensive care unit l__l__l__l j **neo_nl141**

**Neonatal medicine department:** no = 0, yes = 1 l__l **neo_nl142**

If yes, stay < 1 day: no = 0, yes = 1 l__l **neo_nl143**

If stay ≥ 1 day, total number of days in neonatal medicine department l__l__l__l j **neo_nl144**

**Other department(s)**: no = 0, yes = 1 l__l **neo_nl145**

If yes,

Other department 1, in full: ……………………………………………………………………… **neo_nl146**

Total number of days in this department ____________ l__l__l__l j **neo_nl147**

Other department 2, in full: ……………………………………………………………………… **neo_nl148**

Total number of days in this department l__l__l__l j **neo_nl149**

Other department 3, in full: …………………………………………………………… ………… **neo_nl150**

Total number of days in this department l__l__l__l j **neo_nl151**

**Date of discharge from the facility**  l__l__l/ l__l__l /l__l__l__l__l **neo_nl152**

***Age at discharge from the facility (days) neo_nl152b***

**Status at discharge:** alive = 1, dead = 2 l__l **neo_nl155**

***d_nl50f***

If alive, discharge to  l__l **neo_nl156**

***d_nl51f***

1: Home

2: Nursery or other residential care

3: Transfer to another facility*

**Journeys to and from the facility and another site for periods < 24h, e.g. for complementary investigations, are not to be counted as transfers*

4: Other

If 1 or 2, continue to page 60 (Infant’s condition at discharge home)

If 4, please give details:  **neo_nl157**

1. ***Transfer to a 4^th^ hospital facility:***

**Name of discharging facility** in full **neo_nl170**

**City/town of discharging facility** in full **neo_nl171**

**N° of the geographical department of discharging facility** in full l__l__l **neo_nl172**

**Date of discharge**  l__l__l/ l__l__l /l__l__l__l__l **neo_nl173**

***Age at discharge (in days) neo_nl173b***

**Discharging department**  l__l **neo_nl175**

***d_nl23f***

1: Neonatal resuscitation unit

2: Neonatal intensive care unit

3: Neonatal medicine department

4: Other

If other, please specify:…………………………………………………..………………… **neo_nl176**

**Transport**  l__l **neo_nl177**

***d_nl8f***

1: Emergency ambulance (road)

2: Emergency ambulance (helicopter)

3: Nurse-accompanied interhospital transfer

4: Other

If other, please specify: ……………………………………………………………….….. **neo_nl178**

**Transfer to:** higher level* = 1, lower level = 2, other = 3 l__l **neo_nl179**

**Higher level = need for more care or specialized technical facilities*

***d_nl10f***

**Reason(s) for transfer:**

*Give all the reasons leading to transfer*

**Condition(s):**

Prematurity: no = 0, yes = 1 l__l **neo_nl180**

Intrauterine growth retardation (IUGR): no = 0, yes = 1 l__l **neo_nl181**

Respiratory distress: no = 0, yes = 1 l__l **neo_nl182**

Malformation(s): no = 0, yes = 1 l__l **neo_nl183**

Worsening of a condition: no = 0, yes = 1 l__l **neo_nl184**

If yes, please specify:………………………………………………………………………………….. **neo_nl185**

**Need for complementary care:**

Surgery: no = 0, yes = 1 l__l **neo_nl186**

Need for parenteral feeding: no = 0, yes = 1 l__l **neo_nl187**

**Organizational reason(s):**

Lack of space: no = 0, yes = 1 l__l **neo_nl188**

Transfer to hospital closer to home: no = 0, yes = 1 l__l **neo_nl189**

**Other**: no = 0, yes = 1 l__l **neo_nl190**

If yes, please specify: ……………………………………………………………………………… **neo_nl191**

1. *Stay in the 4^th^ hospital facility:*

**Name of discharging facility** in full **neo_nl192**

**City/town of discharging facility** in full **neo_nl193**

**N° of the geographical department of discharging facility** in full l__l__l **neo_nl194**

**FINESS number= neo_finess_etab4**

**Level= neo_niveau_etab4 *d_etabniv***

***Anonymized code number of the facility neo_numetab4***

**Department to which admitted**  l__l **neo_nl195**

***d_nl23f***

1: Neonatal resuscitation unit

2: Neonatal intensive care unit

3: Neonatal medicine department

4: Other

If other, please give details: …………………………………………………………………. **neo_nl196**

**Date of admission to the department**________________________ l__l__l/ l__l__l /l__l__l__l__l **neo_nl197**

***Age at admission to the department (in days) neo_nl197b***

**During the infant’s stay in this facility, was he/she hospitalized in:**

**Neonatal resuscitation unit**: no = 0, yes = 1 l__l **neo_nl199**

If yes,

Length of stay < 1 day: no = 0, yes = 1 l__l **neo_nl200**

If stay ≥ 1 day, total number of days in neonatal resuscitation unit l__l__l__l j **neo_nl201**

Number of stays in neonatal resuscitation unit l__l **neo_nl202**

**Neonatal intensive care unit**: no = 0, yes = 1 l__l **neo_nl203**

If yes, stay < 1 day: no = 0, yes = 1 l__l **neo_nl204**

If stay ≥ 1 day, total number of days in neonatal intensive care unit l__l__l__l j **neo_nl205**

**Neonatal medicine department:** no = 0, yes = 1 l__l **neo_nl206**

If yes, stay < 1 day: no = 0, yes = 1 l__l **neo_nl207**

If stay ≥ 1 day, total number of days in neonatal medicine department l__l__l__l j **neo_nl208**

**Other department(s)**: no = 0, yes = 1 l__l **neo_nl209**

If yes,

Other department 1, in full: …………………………………………………………………….… **neo_nl210**

Total number of days in this department l__l__l__l j **neo_nl211**

Other department 2, in full: …………………………………………………………………….… **neo_nl212**

Total number of days in this department l__l__l__l j **neo_nl213**

Other department 3, in full: …………………………………………………………………….… **neo_nl214**

Total number of days in this department l__l__l__l j **neo_nl215**

**Date of discharge from the facility**  l__l__l/ l__l__l /l__l__l__l__l **neo_nl216**

***Age at discharge from the facility (days) neo_nl216b***

**Status at discharge:** alive = 1, dead = 2 l__l **neo_nl218**

***d_nl50f***

If alive, discharge to  l__l **neo_nl219**

***d_nl51f***

1: Home

2: Nursery or other residential care

3: Transfer to another facility*

** Journeys to and from the facility and another site for periods < 24h, e.g. for complementary investigations, are not to be counted as transfers*

4: Other

If 1 or 2, continue to page 60 (Infant’s condition at discharge home)

If 4, please give details: **neo_nl220**

1. ***Transfer to a 5^th^ hospital facility:***

**Name of discharging facility** in full **neo_nl221**

**City/town of discharging facility** in full **neo_nl222**

**N° of the geographical department of discharging facility** in full l__l__l **neo_nl223**

**Date of discharge**  l__l__l/ l__l__l /l__l__l__l__l **neo_nl224**

***Age at discharge (in days) neo_nl224b***

**Discharging department**  l__l **neo_nl226**

***d_nl23f***

1: Neonatal resuscitation unit

2: Neonatal intensive care unit

3: Neonatal medicine department

4: Other

If other, please give details:………………………………………………………………… **neo_nl227**

**Transport**  l__l **neo_nl228**

***d_nl8f***

1: Emergency ambulance (road)

2: Emergency ambulance (helicopter)

3: Nurse-accompanied interhospital transfer

4: Other

If other, please give details: ……………………………………………………………….. **neo_nl229**

**Transfer to:** higher level* = 1, lower level = 2, other = 3 ______________________________ l__l **neo_nl230**

**Higher level = need for more care or specialized technical facilities*

***d_nl10f***

**Reason(s) for transfer:**

*Give all the reasons leading to transfer*

**Condition(s):**

Prematurity: no = 0, yes = 1 l__l **neo_nl231**

Intrauterine growth retardation (IUGR): no = 0, yes = 1 l__l **neo_nl232**

Respiratory distress: no = 0, yes = 1 l__l **neo_nl233**

Malformation(s): no = 0, yes = 1 l__l **neo_nl234**

Worsening of a condition: no = 0, yes = 1 l__l **neo_nl235**

If yes, please specify:…………………………………………………………………….…………… **neo_nl236**

**Need for complementary care:**

Surgery: no = 0, yes = 1 l__l **neo_nl237**

Need for parenteral feeding: no = 0, yes = 1 l__l **neo_nl238**

**Organizational reason(s):**

Lack of space: no = 0, yes = 1 l__l **neo_nl239**

Transfer to hospital closer to home: no = 0, yes = 1 l__l **neo_n240**

**Other:** no = 0, yes = 1 l__l **neo_nl241**

If yes, please specify: ……………………………………………………………………….………… **neo_nl242**

1. ***Stay in the 5^th^ hospital facility:***

**Name of discharging facility** in full **neo_nl243**

**City/town of discharging facility** in full **neo_nl244**

**N° of the geographical department of discharging facility** in full l__l__l **neo_nl245**

**FINESS number= neo_finess_etab5**

**Level= neo_niveau_etab5 *d_etabniv***

***Anonymized code number of the facility neo_numetab5***

**Department to which admitted**  l__l **neo_nl246**

***d_nl23f***

1: Neonatal resuscitation unit

2: Neonatal intensive care unit

3: Neonatal medicine department

4: Other

If other, please give details: ………………………………………………………………… **neo_nl247**

**Date of admission to the department**________________________ l__l__l/ l__l__l /l__l__l__l__l **neo_nl248**

***Age at admission to the department (in days) ______________________________________ neo_nl248***

**During the infant’s stay in this facility, was he/she hospitalized in:**

**Neonatal resuscitation unit:** no = 0, yes = 1 ______________________________________ l__l **neo_nl250**

If yes,

Length of stay < 1 day: no = 0, yes = 1 l__l **neo_nl251**

If stay ≥ 1 day, total number of days in neonatal resuscitation unit l__l__l__l j **neo_nl252**

Number of stays in neonatal resuscitation unit l__l **neo_nl253**

**Neonatal intensive care unit:** no = 0, yes = 1 l__l **neo_nl254**

If yes, stay < 1 day: no = 0, yes = 1 l__l **neo_nl255**

If stay ≥ 1 day, total number of days in neonatal intensive care unit l__l__l__l j **neo_nl256**

**Neonatal medicine department:** no = 0, yes = 1 l__l **neo_n257**

If yes, stay < 1 day: no = 0, yes = 1 l__l **neo_nl258**

If stay ≥ 1 day, total number of days in neonatal medicine department l__l__l__l j **neo_nl259**

**Other department(s**)**:** no = 0, yes = 1 l__l **neo_nl260**

If yes,

Other department 1, in full: ………………………………………………………………………. **neo_nl261**

Total number of days in this department l__l__l__l j **neo_nl262**

Other department 2, in full: ………………………………………………………………………. **neo_nl263**

Total number of days in this department l__l__l__l j **neo_nl264**

Other department 3, in full: …………………………………………………………………….… **neo_nl265**

Total number of days in this department l__l__l__l j **neo_nl266**

**Date of discharge from the facility**  l__l__l/ l__l__l /l__l__l__l__l **neo_nl267**

***Age at discharge (days) neo_nl267b***

**Status at discharge:** alive = 1, dead = 2 l__l **neo_nl269**

***d_nl50f***

If alive, discharge destination: l__l **neo_nl270**

***d_nl51f***

1: Home

2: Nursery or other residential care

3: Transfer to another facility*

** Journeys to and from the facility and another site for periods < 24h, e.g. for complementary investigations, are not to be counted as transfers*

4: Other

If 1 or 2, continue to page 60 (Infant’s condition at discharge home)

If 4, please give details: **neo_nl271**

1. ***Transfer to a 6^th^ hospital facility:***

**Name of discharging facility** in full **neo_nl272**

**City/town of discharging facility** in full **neo_nl273**

**N° of the geographical department of discharging facility** in full l__l__l **neo_nl274**

**Date of discharge**  l__l__l/ l__l__l /l__l__l__l__l **neo_nl275**

***Age at discharge (in days) neo_nl275b***

**Discharging department**  l__l **neo_nl277**

***d_nl23f***

1: Neonatal resuscitation unit

2: Neonatal intensive care unit

3: Neonatal medicine department

4: Other

If other, please give details:……………………………………………………………… **neo_nl278**

**Transport**  l__l **neo_nl279**

***d_nl8f***

1: Emergency ambulance (road)

2: Emergency ambulance (helicopter)

3: Nurse-accompanied interhospital transfer

4: Other

If other, please give details: ………………………………………………………….…. **neo_nl280**

**Transfer to:** higher level* = 1, lower level = 2, other = 3 l__l **neo_nl281**

**Higher level = need for more care or specialized technical facilities*

***d_nl10f***

**Reason(s) for transfer:**

*Give all the reasons leading to transfer*

**Condition(s):**

Prematurity: no = 0, yes = 1 l__l **neo_nl282**

Intrauterine growth retardation (IUGR): no = 0, yes = 1 l__l **neo_nl283**

Respiratory distress: no = 0, yes = 1 l__l **neo_nl284**

Malformation(s): no = 0, yes = 1 l__l **neo_nl285**

Worsening of a condition: no = 0, yes = 1 l__l **neo_nl286**

If yes, please specify ………………………………………………………………………………… **neo_nl287**

**Need for complementary care:**

Surgery: no = 0, yes = 1 l__l **neo_nl288**

Need for parenteral feeding: no = 0, yes = 1 l__l **neo_nl289**

**Organizational reason(s):**

Lack of space: no = 0, yes = 1 l__l **neo_nl290**

Transfer to hospital closer to home: no = 0, yes = 1 l__l **neo_n291**

**Other**: no = 0, yes = 1 l__l **neo_nl292**

If yes, please specify: …………………………………………………………………………….…… **neo_nl293**

1. ***Stay in the 6^th^ hospital facility:***

**Name of discharging facility** in full **neo_nl294**

**City/town of discharging facility** in full **neo_nl295**

**N° of the geographical department of discharging facility** in full l__l__l **neo_nl296**

**FINESS number= neo_finess_etab6**

**Level= neo_niveau_etab6 *d_etabniv***

***Anonymized code number of the facility neo_numetab6***

**Department to which admitted**  l__l **neo_nl297**

***d_nl23f***

1: Neonatal resuscitation unit

2: Neonatal intensive care unit

3: Neonatal medicine department

4: Other

If other, please give details: …………………………………………………………..…… **neo_nl298**

**Date of admission to the department** l__l__l/ l__l__l /l__l__l__l__l **neo_nl299**

***Age at admission to the department (in days) neo_nl299b***

**During the infant’s stay in this facility, was he/she hospitalized in:**

**Neonatal resuscitation unit**: no = 0, yes = 1 l__l **neo_nl301**

If yes,

Length of stay < 1 day: no = 0, yes = 1 l__l **neo_nl302**

If stay ≥ 1 day, total number of days in neonatal resuscitation unit l__l__l__l j **neo_nl303**

Number of stays in neonatal resuscitation unit l__l **neo_nl304**

**Neonatal intensive care unit**: no = 0, yes = 1 l__l **neo_nl305**

If yes, stay < 1 day: no = 0, yes = 1 l__l **neo_nl306**

If stay ≥ 1 day, total number of days in neonatal intensive care unit l__l__l__l j **neo_nl307**

**Neonatal medicine department:** no = 0, yes = 1 l__l **neo_nl308**

If yes, stay < 1 day: no = 0, yes = 1 l__l **neo_nl309**

If stay ≥ 1 day, total number of days in neonatal medicine department l__l__l__l j **neo_nl310**

**Other department(s)**: no = 0, yes = 1 l__l **neo_nl311**

If yes,

Other department 1, in full: ………………………………………………………………………. **neo_nl312**

Total number of days in this department l__l__l__l j **neo_nl313**

Other department 2, in full: ………………………………………………………………………. **neo_nl314**

Total number of days in this department l__l__l__l j **neo_nl315**

Other department 3, in full: ………………………………………………………………………. **neo_nl316**

Total number of days in this department l__l__l__l j **neo_nl317**

**Date of discharge from the facility** l__l__l/ l__l__l /l__l__l__l__l **neo_nl318**

***Age at discharge (days) neo_nl318b***

**Status at discharge:** alive = 1, dead = 2 l__l **neo_nl320**

***d_nl50f***

If alive, discharge to  l__l **neo_nl321**

***d_nl51f***

1: Home

2: Nursery or other residential care

3: Transfer to another facility*

** Journeys to and from the facility and another site for periods < 24h, e.g. for complementary investigations, are not to be counted as transfers*

4: Other

If 1 or 2, continue to page 60 (Infant’s condition at discharge home)

If 4, please specify: **neo_nl322**

**Active treatment limitation or discontinuation**

**Was there a discussion in the neonatal resuscitation unit on limitation or discontinuation of active resuscitation treatment?**: no = 0, yes = 1 l__l **neo_nm1**

If no, continue to the next section page 60 (Conditions of infant’s discharge home) or page 62 (Infant deceased in neonatal resuscitation/intensive care unit)

**If yes,**

***Decision-making process:***

**Reasons for discussion**:

Treatments no longer effective for the infant’s survival: no = 0, yes = 1 l__l **neo_nm2**

In the event of survival, poor prognosis for future quality of life: no = 0, yes = 1 l__l **neo_nm3**

Disproportionate treatment: heavy burden of current or future treatment: no = 0, yes = 1 l__l **neo_nm4**

Were the disproportionate treatments or poor prognosis or illness incompatible with acceptable quality of life related to:

Neurological condition: no = 0, yes = 1 l__l **neo_nm5**

Respiratory condition: no = 0, yes = 1 l__l **neo_nm6**

Gastrointestinal condition: no = 0, yes = 1 l__l **neo_nm7**

Multiorgan condition: no = 0, yes = 1 l__l **neo_nm8**

Other: no = 0, yes = 1 l__l **neo_nm9**

If yes, please give details: ……………………………………………………………….…… **neo_nm10**

**Were there one (or several) meeting(s) to discuss limitation or discontinuation of active treatment for the infant:** no = 0, yes = 1, information not available = 2 l__l **neo_nm11**

If yes,

Number of meetings where the infant’s case was discussed l__l **neo_nm12**

Was the content of one (or several) meeting(s) entered in the records: no = 0, yes = 1 l__l **neo_nm13**

**Were there one (or several) discussion(s) with the parent(s) before a decision was reached:**

no = 0, yes = 1 l__l **neo_nm14**

If yes, with: the father = 1, the mother = 2, the father and the mother together = 3 l__l **neo_nm15**

***d_nm15f***

**Have the parents required a discussion before any meeting?**: no = 0, yes = 1 l__l

**neo_nm16**

**Degree of involvement of the parents in the decision**  l__l **neo_nm17**

***d_nm17f***

1: The parents were not informed that a decision had been made

2: The parents were informed, but were not asked for their opinion

3: The parents were informed, their opinion was obtained indirectly

4: The parents were informed, and their agreement was obtained

**Opinion of the parents:**

Full continuation of curative treatments: no = 0, yes = 1 l__l **neo_nm18**

Abstention, limitation or medical interruption: no = 0, yes = 1 l__l **neo_nm19**

Rely on medical opinion: no = 0, yes = 1 l__l **neo_nm20**

Opposed to medical opinion: no = 0, yes = 1 l__l **neo_nm21**

Information not available in the records: no = 0, yes = 1 l__l **neo_nm22**

Other: no = 0, yes = 1 l__l **neo_nm23**

If other, please give details: …………………………………………………………………….. **neo_nm24**

**What was the final decision:**

No decision possible (lack of consensus or necessary elements): no = 0, yes = 1 l__l **neo_nm25**

Decision to limit or discontinue active treatment: no = 0, yes = 1 l__l **neo_nm26**

Decision to give sedative and/or analgesic drugs, with the risk of causing death:

no = 0, yes = 1 l__l **neo_nm27**

Decision to continue resuscitation (and initiate all necessary treatments, without limitation): no = 0, yes = 1 l__l **neo_nm28**

If decision to limit or discontinue active treatment:

Abstention in the event of cardiocirculatory arrest: no = 0, yes = 1 l__l **neo_nm29**

Limitation of initiation of new treatments (e.g. reintubation, surgery, drainage, transfusions, etc.): no = 0, yes = 1 l__l **neo_nm30**

Interruption of current treatment(s) (e.g. extubation, discontinuation of artificial feeding, etc.):

no = 0, yes = 1 l__l **neo_nm31**

**Final medical reasons for the decision:**

Uncertain diagnosis and/or prognosis: no = 0, yes = 1 l__l **neo_nm32**

Acceptable treatment plan (no disproportionate risk/benefit of continuing active treatments): no = 0, yes = 1 l__l **neo_nm33**

Unacceptable treatment plan (disproportionate risk/benefit of continuing active treatments): no = 0, yes = 1 l__l **neo_nm34**

No chance to survive (NCTS): no = 0, yes = 1 l__l **neo_nm35**

**Date when final decision** **made**  l__l__l/ l__l__l /l__l__l__l__l **neo_nm36**

***Age of infant when final decision made (in days) neo_nm36b***

**Infant’s condition at discharge home**

**Date of discharge**  l__l__l/ l__l__l /l__l__l__l__l **neo_nn1**

***Age at discharge (days) neo_nn1b***

**Weight at discharge**  l__l__l__l__l g **neo_nn4**

**Head circumference at discharge**  l__l__l cm **neo_nn5**

**Length at discharge**  l__l__l cm **neo_nn6**

**Mean arterial pressure (MAP)* recorded** l__l__l mmHg **neo_nn7**

** First MAP measurement of the day*

**Status assessment by cardiorespiratory recording before discharge from NICU:** no = 0, yes = 1 l__l **neo_nn8**

If yes, please give details: ……………………………………………………………….… **neo_nn9**

**Number of facilities in which the infant was hospitalized**  l__l **neo_nn10**

***Treatments at discharge:***

**Treatment for gastroesophageal reflux:** no = 0, yes = 1 l__l **neo_nn11**

If yes,

Milk-thickening agents: no = 0, yes = 1 l__l **neo_nn12**

Prokinetics: no = 0, yes = 1 l__l **neo_nn13**

Antiacid (omeprazole or similar): no = 0, yes = 1 l__l **neo_nn14**

**Treatment for apnea/bradycardia:** no = 0, yes = 1 l__l **neo_nn15**

If yes,

Caffeine: no = 0, yes = 1 l__l **neo_nn16**

Doxapram: no = 0, yes = 1 l__l **neo_nn17**

**O2:** no = 0, yes = 1 l__l **neo_nn18**

**Monitoring:** no = 0, yes = 1 l__l **neo_nn19**

If yes,

Cardiorespiratory monitoring: no = 0, yes = 1 l__l **neo_nn20**

Pulse oximetry: no = 0, yes = 1 l__l **neo_nn21**

**Antihypertensive treatment:** no = 0, yes = 1 l__l **neo_nn22**

**Prescription of Synagis®:** no = 0, yes = 1 l__l **neo_nn23**

***Feeding:***

**Is the infant still tube-fed**: no = 0, yes = 1 l__l **neo_nn24**

If no, end date of tube feeding l__l__l/ l__l__l /l__l__l__l__l **neo_nn25**

*Age at end of tube feeding (in days)* ***neo_nn25b***

**Does the infant feed directly at the breast**: no = 0, yes = 1 l__l **neo_nn28**

If yes, does this feed form the entire enteral intake: no = 0, yes = 1 l__l **neo_nn29**

**If the infant receives feeds instead of or as a complement to breastfeeding, specify with what milks** (including milk from a milk bank, individualized maternal milk given outside of breastfeeding):

Type of milk n°1 *(cf appendix - Types of milk - Guide to completing the neonatal questionnaire)*  l__l__l l__l l__l **neo_nn30**

Amount prescribed for 24h l__l__l__l ml **neo_nn31**

Type of milk n°2 *(cf appendix - Types of milk - Guide to completing the neonatal questionnaire)* l__l__l l__l l__l **neo_nn32**

Amount prescribed for 24h l__l__l__l ml **neo_nn33**

| **Breastfeeding at discharge from neonatal unit** | **neo_allaitclasse** | 0: no maternal breastfeeding |
| --- | --- | --- |
|  |  | 1: mixed feeding (mother’s milk + formula) |
|  |  | 2: breastfeeding only |

***Follow-up of the infant:***

**Is the infant discharged with a hospital-at-home plan:** no = 0, yes = 1 l__l **neo_nn34**

**Is a home visit planned by a nurse from the mother and child health service:** no = 0, yes = 1 l__l **neo_nn35**

**During their stay, have the parents been given information about follow-up of the infant within the network:**

no = 0, yes = 1 l__l **neo_nn36**

**Have the parents received a list of departments and physicians who can undertake follow-up:**

no = 0, yes = 1 l__l **neo_nn37**

**Will the infant be included in the network follow-up:** no = 0, yes = 1 l__l **neo_nn38**

If no, why  l__l **neo_nn39**

***d_nn39f***

1: No follow-up network

2: Not proposed to the parents by oversight

3: Not proposed, as the infant does not meet the criteria

4: Parental refusal

**Does the infant already have a first follow-up appointment:** no = 0, yes = 1 l__l **neo_nn40**

If yes, where  l__l **neo_nn41**

***d_nn41f***

1: Neonatology department

2: Another department of the hospital

3: Specialized center for early medicosocial action (CAMSP)

4: Private practitioner

If 2 or 3, please detail:…………………………………………………….……………… **neo_nn42**

**Infant deceased in neonatal resuscitation/intensive care unit**

**Infant deceased:** no = 0, yes = 1 l__l **neo_no1**

If yes, **did death follow a decision to discontinue, withhold or limit resuscitation treatments (outside the irreversible death process):** no = 0, yes = 1 l__l **neo_no2**

**If no, continue directly to page 63 to item: Date of the infant’s death**

**If yes,**

***Modalities of death in the event of limitation, withholding or discontinuation of resuscitation treatments:***

**Were any treatments discontinued:** no = 0, yes = 1 l__l **neo_no3**

If yes,

Withdrawal of non-invasive ventilation (vs. PEEP or infant flow): no = 0, yes = 1 l__l **neo_no4**

Oxygen withdrawal: no = 0, yes = 1 l__l **neo_no5**

Terminal weaning: no = 0, yes = 1 l__l **neo_no6**

Extubation: no = 0, yes = 1 l__l **neo_no7**

Discontinuation of vasoactive amines: no = 0, yes = 1 l__l **neo_no8**

Discontinuation of parenteral feeding: no = 0, yes = 1 l__l **neo_no9**

Discontinuation of enteral feeding, whether breast or bottle (if previously given): no = 0, yes = 1,

no enteral feeding = 2 l__l **neo_no10**

***d_no10*** f

Start date of treatment discontinuation process l__...__l **neo_no11**

*Age at start of treatment discontinuation process (in days)* ***neo_no11b***

**Were any treatments limited:** no = 0, yes = 1 l__l **neo_no14**

If yes:

No intubation if required: no = 0, yes = 1 l__l **neo_no15**

No NIV if required: no = 0, yes = 1 l__l **neo_no16**

No surgical closure of ductus arteriosus: no = 0, yes = 1 l__l **neo_no17**

No surgery for necrotizing enterocolitis: no = 0, yes = 1 l__l **neo_no18**

No drainage of hydrocephalus: no = 0, yes = 1 l__l **neo_no19**

No administration of antibiotics: no = 0, yes = 1 l__l **neo_no20**

Other: no = 0, yes = 1 l__l **neo_no21**

If yes, please give details: ………………………………………………………………………… **neo_no22**

Start date of treatment limitation process l__... __l **neo_no23**

*Age at start of treatment limitation process (in days)* ***neo_no23b***

**Were analgesic and/or sedative treatments or other treatments added or increased following the decision to limit or discontinue certain treatments**: no = 0, yes = 1 l__l **neo_no26**

If yes, please specify:

Benzodiazepines: no = 0, yes = 1 l__l **neo_no27**

Phenobarbital: no = 0, yes = 1 l__l **neo_no28**

Morphine derivatives: no = 0, yes = 1 l__l **neo_no29**

Thiopenthal: no = 0, yes = 1 l__l **neo_no30**

Curare: no = 0, yes = 1 l__l **neo_no31**

Other: no = 0, yes = 1 l__l **neo_no32**

If yes, please give details: …………………………………………………………………………. **neo_no33**

Were the doses given proportionate to requirements for sedation/analgesia: no = 0, yes = 1 l__l **neo_no34**

If no, was it necessary to intensify treatment: no = 0, yes = 1 l__l **neo_no35**

**Written report in the records on the duration of the infant’s life, treatments and means employed:**

no = 0, yes = 1 l__l **neo_no36**

**Date of the infant’s death**  l__l__l/ l__l__l /l__l__l__l__l **neo_no37**

***Age of the infant at death (in days) neo_no37b***

**Time of the infant’s death (hour and minute)** l__l__l h l__l__l min **neo_no40 neo_no41**

***Age of the infant at death*** *l__l* ***neo_age_dc_rea***

***(infant deaths in neonatal resuscitation unit N = 413 infants)***

*1: Death between D0-D2*

*2: Death between D3-D7*

*3: Death between D8-D28*

*4: Death ≥ D29*

**Were the parents present at the time of death:** no = 0, yes = 1, information not available = 2 l__l **neo_no42**

If yes,

The father: no = 0, yes = 1 l__l **neo_no43**

The mother: no = 0, yes = 1 l__l **neo_no44**

**Infant placed in arms before death or died in the arms of one or both parents:**

no = 0, yes = 1 l__l **neo_no45**

**At the time of death, was the infant placed in a room alone:** no = 0, yes = 1 l__l **neo_no46**

***Infant died before 36 WGA corrected age***  *l__l* ***neo_dc36SA***

***(infants in the common arm)***

*0: Infant alive at 36 WGA corrected age*

*1: Death before 36 WGA (infant stillborn, or died in the delivery room or neonatal unit)*

***Causes of death (sequence of causes according to the death certificat):***

**Causes of fetal or neonatal origin**

**Cause of fetal or neonatal origin that led directly to death, in full: neo_no47**

**Due to**  **neo_no48**

**Other associated cause(s):** no = 0, yes = 1 l__l **neo_no49**

If yes, please specify: **neo_no50**

***Causes of death in neonatal resuscitation unit*** *l__l* ***neo_cause_dc_rea***

***(infant deaths in neonatal resuscitation unit)***

*1: Immaturity*

*2: Respiratory distress syndrome*

*3: Necrotizing enterocolitis (NEC)*

*4: Infection*

*5: CNS injury*

*6: Other*

*7: Unknown*

*8: Congenital anomalies*

***(Classification of causes of death taken from Patel et al., Causes and Timing of Death in Extremely Premature Infants from 2000 through 2011, NEJM 2015)***

**Causes of obstetric or maternal origin**

**Determinant cause of death of obstetric or maternal origin, in full:**  **neo_no51**

**Other associated cause(s):** no = 0, yes = 1 l__l **neo_no52**

If yes, please specify: **neo_no53**

**Was an autopsy carried out (or will an autopsy be carried out)** l__l **neo_no54**

***d_no54f***

0: no

1: yes, result not available

2: yes, result available

If the result is available, please specify l__l **neo_no55**

***d_no55f***

1: Autopsy established the cause of death

2: Autopsy confirmed the suspected cause of death

3: Autopsy was not informative

**Summary**

Severe neonatal morbidity yes/no neo_morbidite

| *(Severe brain lesions (IVH3, IVH4, leukomalacia with cavitations) and/or severe bronchodysplasia, and/or stage 3 or higher retinopathy, or laser treatment and/or stage 2 or 3 necrotizing enterocolitis.)* |
| --- |
