# Supplementary material for: Premedication practices for delivery room intubations in premature infants in France: Results from the EPIPAGE 2 cohort study
Source: PLoS One. 2019 Apr 10;14(4):e0215150. doi: 10.1371/journal.pone.0215150 (PMC6457540; doi:10.1371/journal.pone.0215150)
Supplement: S4 File — Data that had to be fulfilled with the neonates’ files and the neonatal team. (DOCX) [file pone.0215150.s004.docx]

**
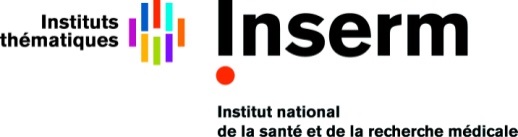
**

**
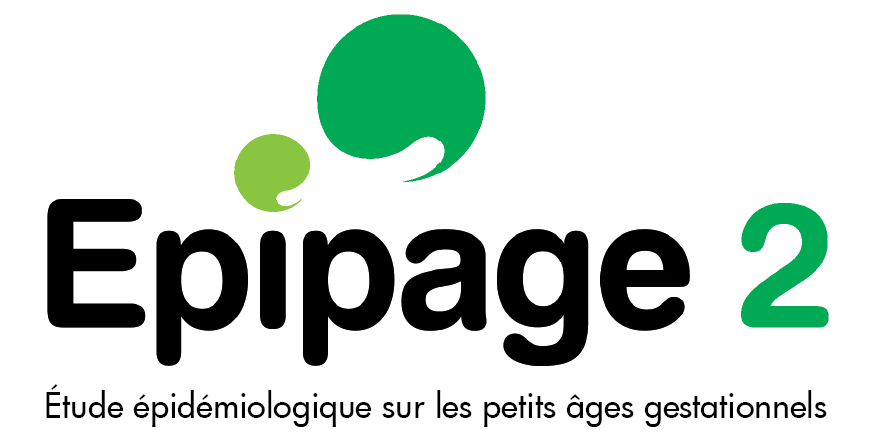
**

**QUESTIONNAIRE NEONATOLOGIE**

**annoté**

**Données à recueillir à partir**

**du dossier néonatal**

**et de l’équipe de néonatologie**

| **Variables surlignées en rouge** | **Variables identifiantes – Non transmises** |
| --- | --- |
| **Variables surlignées en jaune** | **Variables indirectement identifiantes – Transmises sous condition** |
| ***Variables en vert*** | **Variables calculées** |
| ***Variables en marron*** | **Variables de synthèse** |
| ***Modalités de réponses (d_variable)*** | **Dictionnaire** |
| Encadrements en violet | **Blocs conditionnels** |

**Table des matières**

**DEVELOPPEMENT PULMONAIRE ET COMPLICATIONS RESPIRATOIRES page 4**

**Surfactant page 4**

**Ventilation page 4**

**Corticothérapie post natale page 5**

**Monoxyde d’azote inhalé page 6**

**Dysplasie broncho-pulmonaire page 7**

**EVALUATION NEUROLOGIQUE DU NOUVEAU-NE page 8**

**Echographie transfontanellaire page 8**

**Electroencéphalogramme page 17**

**Imagerie par résonnance magnétique page 18**

**Traitement neurochirurgical page 18**

**TRAITEMENTS ET SOINS AU NOUVEAU-NE page 19**

**Antalgie  page 18**

**Hématologie page 22**

**Néphrologie page 23**

**Ictère page 25**

**Natrémie page 25**

**Infection précoce page 25**

**Infections secondaires page 26**

**Hémodynamique page 31**

**SOINS DE DEVELOPPEMENT page 35**

**NUTRITION page 39**

**COMPLICATIONS DIVERSES page 45**

**ANOMALIES CONGENITALES page 46**

**RETINOPATHIE page 47**

**AUDITION page 47**

**VACCINATION page 48**

**SYNTHESE DU PARCOURS DES ENFANTS page 49**

**LIMITATION OU ARRET DES TRAITEMENTS ACTIFS page 67**

**CONDITIONS DE SORTIE DE L’ENFANT A DOMICILE page 69**

**ENFANT DECEDE EN REANIMATION NEONATALE / NEONATOLOGIE page 71**

**Identifiant de l’enfant**  l__l__l__l__l__ **enr_code_enfant**

**Identifiant de la maman**  l__l__l__l__l__ **enr_code_mere**

**Date de naissance de l’enfant**  l__l__l/ l__l__l /l__l__l__l__l **cor**_**ddn_enfant**

**L’enfant participe-t-il à un essai clinique** : non = 0, oui = 1 l__l **neo_na1**

Si oui, préciser en clair le nom de l’essai :……………………………………………………………… **neo_na2**

*A l’admission ou dans les 12 premières heures dans le 1^er^ service d’hospitalisation :*

**Poids de naissance corrigé l__l__l__l__l g cor*_*pds_nce**

| **Poids de naissance courbes EPOPE (z-score)** | **neo_PNzscore_epope** | variable continue |
| --- | --- | --- |
|  |  |  |
| **Poids de naissance courbes EPOPE (percentiles)** | **neo_PNperc_epope** | variable continue |
|  |  |  |
|  | **neo_PN10p_epope** | 0 : poids de naissance <10e percentile selon courbes EPOPé |
|  |  | 1 : poids de naissance ≥10e percentile selon courbes EPOPé |
|  |  |  |
|  | **neo_PN3p_epope** | 0 : poids de naissance <3e percentile selon courbes EPOPé |
|  |  | 1 : poids de naissance ≥3e percentile selon courbes EPOPé |

***REF courbe EPOPé :*** *1 Ego A, Prunet C, Lebreton E et al. Customized and non-customized French intrauterine growth curves. I – Methodology J Gynecol Obstet Biol Reprod 2016 ;45:155-64*

**PC** l__l__l cm **neo_pcnce**

**Température** l__l__l, l__l °C **neo_nb5**

**Base déficit max. dans les 12 premières heures** l__l__l, l__l mmol/l **neo_nb6**

**Développement pulmonaire et complications respiratoires**

Surfactant

**Surfactant administré, y compris en salle de naissance** : non = 0, oui = 1 l__l **neo_nc1**

Si oui :

Type de surfactant : Curosurf® = 1, autre = 2 l__l **neo_nc2**

*d_nc2f*

Age à la première dose l__l__l h l__l__l min **neo_nc3 neo_nc4**

Première dose administrée en : salle de naissance = 1, néonatologie = 2 l__l **neo_nc5**

***d_nc5f***

Dose à la première administration l__l__l__l mg/kg **neo_nc6**

Administration de surfactant suivi d’une extubation immédiate (INSURE) : non=0, oui=1 l__l **neo_nc7**

Nombre total de doses administrées l__l **neo_nc8**

Age à la deuxième dose l__l__l h **neo_nc9**

Age à la troisième dose l__l__l__l h **neo_nc10**

Ventilation

**Durée cumulée d’assistance respiratoire :**

Par ventilation mécanique (VM) l__l__l__l j **neo_nc11**

Par ventilation non invasive (VNI) l__l__l__l j **neo_nc12**

Par oscillation à haute fréquence (OHF) l__l__l__l j **neo_nc13**

**Oscillation à haute fréquence (OHF) < J8** : non = 0, oui = 1 l__l **neo_nc14**

Si oui, indication : en premier = 1, rescue = 2 l__l **neo_nc15**

***d_nc15f***

**Date de la première extubation**  l__l__l/ l__l__l /l__l__l__l__l **neo_nc16**

***Age à la première extubation (en jours) neo_nc16b***

**Date de l’extubation définitive**  l__l__l/ l__l__l /l__l__l__l__l **neo_nc19**

***Age à l’extubation définitive (en jours) neo_nc19b***

**Age à la première mise sous CPAP**  l__l__l j l__l__l h **neo_nc22 neo_nc23**

**Date d’arrêt définitif de la CPAP**  l__l__l/ l__l__l /l__l__l__l__l **neo_nc24**

***Age à l’arrêt définitif de la CPAP (en jours) neo_nc24b***

**Date du sevrage définitif en oxygénothérapie (FiO2 = 21%)**  l__...__l **neo_nc27**

***Age du sevrage définitif en oxygénothérapie (FiO2 = 21%) (en jours) neo_nc27b***

**Date d’arrêt définitif de l’O2 sous-nasal (lunettes)**  l__...__l **neo_nc30**

***Age à l’arrêt définitif de l’O2 sous-nasal (en jours) neo_nc30b***

| *Enfants tronc commun nés vivants et admis en néonatologie (COR_STATUTNAIS = 4 ou 5)* | | |
| --- | --- | --- |
| **Durée cumulée de ventilation mécanique** | **neo_dureeVM** | continue (jours) |
|  |  |  |
|  |  |  |
| **Age au sevrage définitif de la CPAP (SA)** | **neo_sevrageCPAP** | continue (SA révolues) |
|  |  |  |
| **Tentative de CPAP dans les 24 premières heures de vie** | **neo_CPAP_H24** | 0 : intubation en SDN ou avant H1, jamais extubé dans les 24 premières heures de vie |
|  |  | 1 : mise en CPAP en SDN ou 1ère extubation avant H24, qu'il y ait réintubation par la suite ou non |

**Evènement(s) respiratoire(s)** : non = 0, oui = 1 l__l **neo_nc33**

Si oui :

Maladie des membranes hyalines : non = 0, oui = 1 l__l **neo_nc34**

Hémorragie pulmonaire : non = 0, oui = 1 l__l **neo_nc35**

Hypertension artérielle pulmonaire (HTAP) prouvée : non = 0, oui = 1 l__l **neo_nc36**

Si oui :

Par échographie cardiaque : non = 0, oui = 1 l__l **neo_nc37**

Par différentielle SpO2 pré et post ductale : non = 0, oui = 1 l__l **neo_nc38**

Pneumothorax : non = 0, oui = 1 l__l **neo_nc39**

Extubation accidentelle nécessitant une ré-intubation : non = 0, oui = 1 l__l **neo_nc40**

Corticothérapie post natale (CPN)

**Administration d’hémisuccinate d’hydrocortisone (HSHC)**: non = 0, oui = 1 l__l **neo_nc41**

Si oui, indication  l__l **neo_nc42**

***d_nc42f***

1 : Prévention de la dysplasie broncho-pulmonaire

2 : Substitutif

3 : Hémodynamique

**Utilisation de CPN** : non = 0, oui = 1 l__l **neo_nc43**

Si oui, molécule : Dexaméthasone = 1, Bétaméthasone = 2, les deux = 3 l__l **neo_nc44**

***d_nc44f***

*Cure n°1 de CPN :*

**Voie d’administration** : générale = 1, inhalée = 2 l__l **neo_nc45**

***d_nc45f***

**Indication**  l__l **neo_nc46**

***d_nc46f***

1 : Extubation

2 : Arrêt de la CPAP

3 : Autre

Si autre, préciser en clair : **neo_nc47**

**Etat de l’enfant à l’indication :** **NEO_NC70A**

FiO2 l__l__l % **neo_nc48**

Oxygénothérapie : lunettes = 1, CPAP = 2, VM = 3 l__l **neo_nc49**

***d_nc49f***

**Date de début de la corticothérapie**  l__l__l/ l__l__l /l__l__l__l__l **neo_nc50**

***Age au début de la corticothérapie (en jours) neo_nc50b***

**Date de fin de la corticothérapie**  l__l__l/ l__l__l /l__l__l__l__l **neo_nc53**

***Age à la fin de la corticothérapie (en jours) neo_nc53b***

**Dose max utilisée**  l__l__l, l__l__l mg/kg/j **neo_nc56**

**Dose cumulée de la cure n°1**  l__l__l__l mg **neo_nc57**

**Arrêt prématuré de la cure n°1**: non = 0, oui = 1 l__l **neo_nc58**

*Total des cures si plus d’une :*

**Voie d’administration** : générale = 1, inhalée = 2, les deux = 3 l__l **neo_nc59**

***d_nc59f***

**Date de sevrage définitif de la corticothérapie**  l__l__l/ l__l__l /l__l__l__l__l **neo_nc60**

***Age au sevrage définitif de la corticothérapie (en jours) neo_nc60b***

**Dose max utilisée**  l__l__l, l__l__l__l mg/kg/j **neo_nc63**

**Dose cumulée totale en systémique**  l__l__l__l mg/kg **neo_nc64**

**Dose cumulée totale en inhalé**  l__l__l__l mg/kg **neo_nc65**

Monoxyde d’azote inhalé (iNO)

**Utilisation de iNO** : non = 0, oui = 1 l__l **neo_nc66**

Si oui :

**Indication** :

Anti-inflammatoire : non = 0, oui = 1 l__l **neo_nc67**

Hypoxémie réfractaire : non = 0, oui = 1 l__l **neo_nc68**

Si oui, associée à une HTAP : non = 0, oui = 1 l__l **neo_nc69**

Autre : non = 0, oui = 1 l__l **neo_nc70**

Si oui, préciser en clair : ….  **neo_nc70a**

**Indication toujours posée à l’échographie cardiaque** : non = 0, oui = 1 l__l **neo_nc71**

**Date de début**  l__l__l/ l__l__l /l__l__l__l__l **neo_nc72**

***Age au début (en jours) neo_nc72b***

**Date du sevrage définitif**  l__l__l/ l__l__l /l__l__l__l__l **neo_nc75**

***Age au sevrage définitif (en jours) neo_nc75b***

**Dose maximale utilisée**  l__l__l ppm **neo_nc78**

Dysplasie broncho-pulmonaire

**Nombre de jours d’O2 de la naissance à J28** l__l__l j **neo_nc79**

**Enfant recevant de l’O2 à J28** : non = 0, oui = 1 l__l **neo_nc80**

***Enfant recevant de l’O2 à J28 : non = 0, oui = 1 l__l neo_O2J28***

Si oui, type d’oxygénothérapie : lunettes = 1, CPAP = 2, VM = 3 l__l **neo_nc81**

***d_nc81f***

***Assistance respiratoire ou O2 à J28*** *non = 0, oui = 1 l__l* ***neo_bdpj28***

**Nombre de jours d’O2 de la naissance à S36** l__l__l__l j **neo_nc82**

**Enfant recevant de l’O2 à S36** : non = 0, oui = 1 l__l **neo_nc83**

***Enfant recevant de l’O2 à S36 : non = 0, oui = 1 l__***l ***neo_O2S36***

Si oui, type d’oxygénothérapie : lunettes = 1, CPAP = 2, VM = 3 l__l **neo_nc84**

***d_nc84f***

**FiO2 à S36**  l__l__l % **neo_nc85**

***FiO2 à S36 l__l__l % neo_fio2***

**Test de Walsh fait à S36*** : non = 0, oui = 1 l__l **neo_nc86**

**Patient déclaré non dysplasique s’il maintient une saturation supérieure à 88 % pendant 30 minutes sous FiO2 à 21%*

Si oui :

Valeur de la SpO2 minimum si FiO2 = 21 % l__l__l **neo_nc87**

Sevrage de l’O2 suite au test : non = 0, oui = 1 l__l **neo_nc88**

Si non, préciser la cause en clair :…………………………………………………………….. **neo_nc89**

***Dysplasie broncho-pulmonaire en 4 stades***  *l__l* ***neo_DBP_grav***

***(enfants vivants à S36, variable créée par Héloïse Torchin)***

***d_dbp_grav***

*0 : Pas de DBP*

*1 : DBP légère* (≥ 28j d’O2 et VSAA à S36)

*2 : DBP modérée* (≥ 28j d’O2 et Ventilation mécanique ou CPAP ou FiO2>21% à S36)

*3 : DBP sévère* (≥ 28j d’O2 et Ventilation mécanique ou CPAP ou FiO2≥30% à S36)

***Dysplasie broncho-pulmonaire modérée ou sévère***  *l__l* neo_DBP_S36

***(enfants vivants à S36, variable créée par Héloïse Torchin) d_dbps36***

*0 : Pas de DBP ou DBP légère à 36 SA*

*1 : DBP modérée ou sévère à 36SA*

***O_2_ pendant au moins 28 jours avec à S36 soit FiO_2_ >= 30% ou ventilation mécanique ou CPAP :***

*non = 0, oui = 1*  *l__l* ***neo_bdpsevere***

***d_yorn***

Synagis®

**L’enfant a-t-il reçu du Palivizumab (Synagis®)** : non = 0, oui = 1 l__l **neo_nc90**

Si oui, date  l__l__l/ l__l__l /l__l__l__l__l **neo_nc91**

***Age au moment du Synagis neo_nc91b***

**Evaluation neurologique du nouveau-né**

Echographie transfontanellaire (ETF)

*ETF réalisée(s) pendant la 1ère semaine de vie :* non = 0, oui = 1 l__l **neo_nd1**

*Si oui, remplir les items ci-dessous observés sur la (ou les) ETF de la première semaine de vie.*

*En cas d’une même lésion observée à plusieurs reprises, mentionner la plus péjorative.*

**Présence d’hémorragie** : non = 0, oui = 1 l__l neo_nd2

Si oui,

**Stade le plus élevé** l__l **neo_nd3**

***d_nd3f***

1 : Hémorragie sous-épendymaire (HSE)

2 : Hémorragie intraventriculaire (HIV) sans dilatation ventriculaire

3 : Hémorragie intraventriculaire (HIV) avec dilatation ventriculaire

**Mesures de « l’index ventriculaire » selon Levene (cf. figure 1 du « guide de remplissage ») :**

Droite  l__l__l, l__l__l mm **neo_nd4**

Gauche  l__l__l, l__l__l mm **neo_nd5**

**Présence d’anomalies parenchymateuses** : non = 0, oui = 1 l__l **neo_nd6**

Si oui,

**Hyperéchogénicités* périventriculaires « précoces »** : non = 0, oui = 1 l__l **neo_nd7**

**Echogénicités supérieures ou égales à celles du plexus choroïde*

Si oui, unilatérales = 1, bilatérales = 2 l__l **neo_nd8**

***d_nd8f***

Si unilatérales, infarctus de Volpe (anciennement « hémorragie grade 4 ») : non = 0, oui = 1 l__l **neo_nd9**

**Cavitation(s)** : non = 0, oui = 1 l__l **neo_nd10**

Si oui,

Unique = 1, multiples = 2 l__l **neo_nd11**

***d_nd11f***

Unilatérales = 1, bilatérales = 2 l__l **neo_nd12**

***d_nd12f***

Si unilatérales, porencéphalie : non = 0, oui = 1 l__l **neo_nd13**

**Autre(s) anomalie(s)** : non = 0, oui = 1 l__l **neo_nd14**

Si oui, préciser en clair : ……………………………………………………………………………… **neo_nd15**

**Les variables de synthèse ci-dessous concernent les enfants inclus et sortis vivants du tronc commun, de 22-34SA admis en USI avec au moins 1 ETF**

***Présence d’un kyste*** *l__l* ***neo_kyste_1***

*0 : Non 3 : Kyste paraventriculaire* ***d_kyste_1f***

*1 : Kyste plexus choroïde 4 : Kyste frontaux*

*2 : Kyste sous épendymaire 5 : Autres*

***Présence d’un vaisseau thalamo striés*** *: non = 0, oui = 1 l__l* ***neo_vaisso_1***

***Présence d’une dilatation ventriculaire*** *: non = 0, oui = 1 l__l* ***neo_dilatation_1***

***Présence d’une anomalie des noyaux gris centraux (ngc)*** *: non = 0, oui = 1 l__l* ***neo_ngc_1***

***Présence d’une anomalie du cervelet*** *: non = 0, oui = 1 l__l* ***neo_cervelet_1***

***Présence d’une hémorragie bilatérale*** *: non = 0, oui = 1 l__l* ***neo_volpebilat_1***

*ETF réalisée(s) pendant la 2^ème^ semaine de vie :* non = 0, oui = 1 l__l **neo_nd16**

*Si oui, remplir les items ci-dessous observés sur la (ou les) ETF de la deuxième semaine de vie. En cas d’une même lésion observée à plusieurs reprises, mentionner la plus péjorative.*

**Présence d’hémorragie** : non = 0, oui = 1 l__l **neo_nd17**

Si oui,

**Stade le plus élevé** l__l **neo_nd18**

***d_nd3f***

1 : Hémorragie sous-épendymaire (HSE)

2 : Hémorragie intraventriculaire (HIV) sans dilatation ventriculaire

3 : Hémorragie intraventriculaire (HIV) avec dilatation ventriculaire

**Mesures de « l’index ventriculaire » selon Levene (cf. figure 1 du « guide de remplissage ») :**

Droite  l__l__l, l__l__l mm **neo_nd19**

Gauche  l__l__l, l__l__l mm **neo_nd20**

**Présence d’anomalies parenchymateuses** : non = 0, oui = 1 l__l **neo_nd21**

Si oui,

**Hyperéchogénicités* périventriculaires** : non = 0, oui = 1 l__l **neo_nd22**

**Echogénicités supérieures ou égales à celles du plexus choroïde*

Si oui, unilatérales = 1, bilatérales = 2 l__l **neo_nd23**

***d_nd8f***

Si unilatérales, infarctus de Volpe (anciennement « hémorragie grade 4 ») : non = 0, oui = 1 l__l **neo_nd24**

**Cavitation(s)** : non = 0, oui = 1 l__l **neo_nd25**

Si oui,

Unique = 1, multiples = 2 l__l **neo_nd26**

***d_nd11f***

Unilatérales = 1, bilatérales = 2 l__l **neo_nd27**

***d_nd12f***

Si unilatérales, porencéphalie : non = 0, oui = 1 l__l **neo_nd28**

**Autre(s) anomalie(s)** : non = 0, oui = 1 l__l **neo_nd29**

Si oui, préciser en clair : …………………………………………………………………………… **neo_nd30**

**Les variables de synthèse ci-dessous concernent les enfants inclus et sortis vivants du tronc commun, de 22-34SA admis en USI avec au moins 1 ETF**

***Présence d’un kyste l__l neo_kyste_2***

*0 : Non 3 : Kyste paraventriculaire* ***d_kyste_1f***

*1 : Kyste plexus choroïde 4 : Kyste frontaux*

*2 : Kyste sous épendymaire 5 : Autres*

***Présence d’un vaisseau thalamo striés :*** *non = 0, oui = 1* ***l__l neo_vaisso_2***

***Présence d’une dilatation ventriculaire :*** *non = 0, oui = 1* ***l__l neo_dilatation_2***

***Présence d’une anomalie des noyaux gris centraux (ngc) :*** *non = 0, oui = 1* ***l__l neo_ngc_2***

***Présence d’une anomalie du cervelet :*** *non = 0, oui = 1* ***l__l neo_cervelet_2***

***Présence d’une hémorragie bilatérale :*** *non = 0, oui = 1* ***l__l neo_volpebilat_2***

*ETF réalisée(s) pendant la 3^ème^ semaine de vie :* non = 0, oui = 1 l__l neo_**nd31**

*Si oui, remplir les items ci-dessous observés sur la (ou les) ETF de la troisième semaine de vie. En cas d’une même lésion observée à plusieurs reprises, mentionner la plus péjorative.*

**Présence d’hémorragie** : non = 0, oui = 1 l__l **neo_nd32**

Si oui,

**Stade le plus élevé**  l__l neo_**nd33**

***d_nd3f***

1 : Hémorragie sous-épendymaire (HSE)

2 : Hémorragie intraventriculaire (HIV) sans dilatation ventriculaire

3 : Hémorragie intraventriculaire (HIV) avec dilatation ventriculaire

**Mesures de « l’index ventriculaire » selon Levene :**

Droite  l__l__l, l__l__l mm **neo_nd34**

Gauche  l__l__l, l__l__l mm **neo_nd35**

**Présence d’anomalies parenchymateuses** : non = 0, oui = 1 l__l **neo_nd36**

Si oui,

**Hyperéchogénicités* péri ventriculaires** : non = 0, oui = 1 l__l **neo_nd37**

**Echogénicités supérieures ou égales à celles du plexus choroïde*

Si oui, unilatérales = 1, bilatérales = 2 l__l **neo_nd38**

***d_nd8f***

Si unilatérales, infarctus de Volpe (anciennement « hémorragie grade 4 ») : non = 0, oui = 1 l__l **neo_nd39**

**Cavitation(s)** : non = 0, oui = 1 l__l **neo_nd40**

Si oui,

Unique = 1, multiples = 2 l__l **neo_nd41**

***d_nd11f***

Unilatérales = 1, bilatérales = 2 l__l **neo_nd42**

***d_nd12f***

Si unilatérales, porencéphalie : non = 0, oui = 1 l__l **neo_nd43**

**Autre(s) anomalie(s)** : non = 0, oui = 1 l__l **neo_nd44**

Si oui, préciser en clair : ……………………………………………………………………………… **neo_nd45**

**Les variables de synthèse ci-dessous concernent les enfants inclus et sortis vivants du tronc commun, de 22-34SA admis en USI avec au moins 1 ETF**

***Présence d’un kyste***  *l__l* ***neo_kyste_3***

*0 : Non 3 : Kyste paraventriculaire* ***d_kyste_1f***

*1 : Kyste plexus choroïde 4 : Kyste frontaux*

*2 : Kyste sous épendymaire 5 : Autres*

***Présence d’un vaisseau thalamo striés*** *: non = 0, oui = 1 l__l* ***neo_vaisso_3***

***Présence d’une dilatation ventriculaire*** *: non = 0, oui = 1 l__l* ***neo_dilatation_3***

***Présence d’une anomalie des noyaux gris centraux (ngc)*** *: non = 0, oui = 1 l__l* ***neo_ngc_3***

***Présence d’une anomalie du cervelet*** *: non = 0, oui = 1 l__l* ***neo_cervelet_3***

***Présence d’une hémorragie bilatérale*** *: non = 0, oui = 1 l__l* ***neo_volpebilat_3***

*Dernière ETF réalisée avant la sortie à domicile :*

**Date de la dernière ETF**  l__l__l/ l__l__l /l__l__l__l__l **neo_nd46**

***Age à la dernière ETF (en jours) neo_nd46b***

Qui a pratiqué cet examen : senior = 1, non senior = 2 l__l **neo_nd49**

***d_nd49f***

**Elargissement des espaces sous-arachnoïdiens*** : non = 0, oui = 1 l__l **neo_nd50**

* *La largeur de l’espace sous-arachnoïdien est considérée comme normale si toutes les mesures sont inférieures à 4 mm.*

Si oui, mesure des espaces sous-arachnoïdiens :

Mesure sur une coupe passant par le 3^ème^ ventricule (cf. figure 2 du « guide de remplissage ») :

Droite l__l__l, l__l__l mm **neo_nd51**

Gauche  l__l__l, l__l__l mm **neo_nd52**

Mesure sur une coupe ventriculaire postérieure (cf. figure 3 du « guide de remplissage ») :

Droite  l__l__l, l__l__l mm **neo_nd53**

Gauche  l__l__l, l__l__l mm **neo_nd54**

**Elargissement de la scissure inter hémisphérique*** : non = 0, oui = 1 l__l **neo_nd55**

**La largeur de la scissure inter hémisphérique est considérée comme normale lorsqu’elle est inférieure à 3 mm.*

Si oui, mesure (cf. figure 2 du « guide de remplissage ») l__l__l, l__l__l mm **neo_nd56**

**Présence d’hémorragie** : non = 0, oui = 1 l__l **neo_nd57**

Si oui,

**Stade**  l__l **neo_nd58**

***d_nd3f***

1 : Hémorragie sous-épendymaire (HSE)

2 : Hémorragie intraventriculaire (HIV) sans dilatation ventriculaire

3 : Hémorragie intraventriculaire (HIV) avec dilatation ventriculaire

**Mesure de « l’index ventriculaire » selon Levene :**

Droite  l__l__l, l__l__l mm **neo_nd59**

Gauche  l__l__l, l__l__l mm **neo_nd60**

**Présence d’anomalies parenchymateuses** : non = 0, oui = 1 l__l **neo_nd61**

Si oui,

**Hyperéchogénicités* périventriculaires** : non = 0, oui = 1 l__l **neo_nd62**

**Echogénicités supérieures ou égales à celles du plexus choroïde*

Si oui,

Unilatérales = 1, bilatérales = 2 l__l **neo_nd63**

***d_nd8f***

Si unilatérales, infarctus de Volpe (anciennement « hémorragie grade 4 ») : non = 0, oui = 1 l__l **neo_nd64**

**Cavitation(s)** : non = 0, oui = 1 l__l **neo_nd65**

Si oui,

Unique = 1, multiples = 2 l__l **neo_nd66**

***d_nd11f***

Unilatérales = 1, bilatérales = 2 l__l **neo_nd67**

***d_nd12f***

Si unilatérales, porencéphalie : non = 0, oui = 1 l__l **neo_nd68**

**Autre(s) anomalie(s)** : non = 0, oui = 1 l__l **neo_nd69**

Si oui, préciser en clair : ……………………………………………………………………………… **neo_nd70**

**Les variables de synthèse ci-dessous concernent les enfants inclus et sortis vivants du tronc commun, de 22-34SA admis en USI avec au moins 1 ETF**

***Présence d’un kyste***  *l__l* ***neo_kyste_S***

*0 : Non 3 : Kyste paraventriculaire* ***d_kyste_1f***

*1 : Kyste plexus choroïde 4 : Kyste frontaux*

*2 : Kyste sous épendymaire 5 : Autres*

***Présence d’un vaisseau thalamo striés*** *: non = 0, oui = 1 l__l* ***neo_vaisso_S***

***Présence d’une dilatation ventriculaire*** *: non = 0, oui = 1 l__l* ***neo_dilatation_S***

***Présence d’une anomalie des noyaux gris centraux (ngc)*** *: non = 0, oui = 1 l__l* ***neo_ngc_S***

***Présence d’une anomalie du cervelet*** *: non = 0, oui = 1 l__l* ***neo_cervelet_S***

***Présence d’une hémorragie bilatérale*** *: non = 0, oui = 1 l__l* ***neo_volpebilat_S***

***ETF réalisée :*** *l__l* ***neo_etfrealisee***

*1- au moins une ETF réalisée 0-aucune ETF réalisée* ***.****-au moins une ETF sur 4 est manquante et les autres NR* ***d_etfrealisee***

***Cavitation quelque soit l'ETF*** (porencephalies exclues) ***:*** *l__l* ***neo_cavitationetf***

*0-Pas cavitation quelque soit ETF 1- Présence d’une cavitation à au moins une ETF*  ***d_cavitationetf***

***Variable HIV exclusive 4 classes quelque soit l'ETF****: l__l* ***neo_superhivvolpe***

| *1- Volpe* | ***d_superhivvolpe*** |
| --- | --- |
| *2- HIV3* | *30- Hémorragie non précisée* |
| *3- HIV2 (*avec kystes plexus choroides) | *35- Pas d'hémorragie* |
| *4- HIV1* (avec kystes sous épendymaires) |  |

***Présence de lésions sévères :*** *l__l* ***neo_ls***

*0-Pas lésion sévère (cavitation,HIV4,HIV3) 1- Présence de lésions sévères* ***d_ls***

***Date d'apparition de la lésion la plus sévère HIV4>HIV3>HIV2>HIV1 :*** *l__l* ***neo_superdelai***

| *1- 1è semaine* | ***d_superdelai*** |
| --- | --- |
| *2- 2è semaine* | *4- 4è semaine* |
| *3- 3è semaine* |  |

***Anomalie parenchymateuse quelque soit l’ETF*** (VOLPE inclus et porencephalies inclues) ***:*** *l__l* ***neo_parenetf***

*0-Pas anomalie parenchymateuse quelque soit ETF 1- Présence anomalie parenchymateuse à au moins une ETF* ***d_parenetf***

***Hémorragie de Volpe quelque soit l'ETF*** (VOLPE inclus et porencephalies inclues) ***:*** *l__l* ***neo_volpeetf***

*0-Pas hémorragie de Volpe quelque soit ETF 1- Présence d’une hémorragie de Volpe à au moins une ETF* ***d_volpeetf***

Electroencéphalogramme (EEG)

**Un ou plusieurs EEG ont-ils été pratiqués en période néonatale** : non = 0, oui = 1 l__l **neo_nd71**

Si oui, combien  l__l **neo_nd72**

*Premier EEG réalisé :*

**Date** **du 1^er^ EEG réalisé**  l__l__l/ l__l__l /l__l__l__l__l  **neo_nd73**

***Age au 1^er^ EEG réalisé (en jours) neo_nd73b***

**Pouvez-vous donner des détails sur le tracé de fond** : non = 0, oui = 1 l__l **neo_nd76**

Si oui, **tracé de fond** : Normal : non = 0, oui 1 l__l **neo_nd77**

Dysmature* : non = 0, oui = 1 l__l **neo_nd78**

Mal organisé** : non = 0, oui = 1 l__l **neo_nd79**

Trop discontinu*** : non = 0, oui = 1 l__l **neo_nd80**

Tracé plat : non = 0, oui = 1 l__l **neo_nd81**

**éléments maturatifs de 15 jours ou plus en moins par rapport au terme annoncé*

***éléments maturatifs mal dessinés et pas de détermination de terme possible*

****discontinuité > 60 sec avant 28 SA ; > 30 sec avant 30 SA, > 20 sec après 30 SA*

**Figures anormales présentes à l’EEG** : non = 0, oui = 1 l__l **neo_nd82**

Si oui, **pouvez-vous donner des détails sur les figures anormales** : non = 0, oui = 1 l__l **neo_nd83**

Si oui**, figures anormales** : Pointes positives rolandiques type A ≥ 1/min : non = 0, oui = 1 l__l **neo_nd84**

Pointes positives rolandiques type A< 1/min : non = 0, oui = 1 l__l **neo_nd85**

Delta brushes anormales : non = 0, oui = 1 l__l **neo_nd86**

Crises électriques : non = 0, oui = 1 l__l **neo_nd87**

**Conclusion de l’examen** : normal = 1, altérations modérées = 2, altérations sévères = 3 l__l **neo_nd88**

***d_nd88f***

*EEG ultérieurs réalisés :*

**Les EEG ultérieurs (à l’exception du dernier) ont-ils montré une ou des anomalies non observées**

**sur le premier tracé :** non = 0, oui = 1 l__l **neo_nd89**

Si oui,

**Anomalies du tracé de fond** : Dysmature* : non = 0, oui = 1 l__l **neo_nd90**

Mal organisé** : non = 0, oui = 1 l__l **neo_nd91**

Trop discontinu*** : non = 0, oui = 1 l__l **neo_nd92**

Tracé plat : non = 0, oui = 1 l__l **neo_nd93**

**Figures anormales** : Pointes positives rolandiques type A ≥ 1/min : non = 0, oui = 1 l__l **neo_nd94**

Pointes positives rolandiques type A < 1/min : non = 0, oui = 1 l__l **neo_nd95**

Delta brushes anormales : non = 0, oui = 1 l__l **neo_nd96**

Crises électriques : non = 0, oui = 1 l__l **neo_nd97**

*Dernier EEG réalisé :*

**Date du dernier EEG réalisé**  l__l__l/ l__l__l /l__l__l__l__l **neo_nd98**

***Age au dernier EEG réalisé (en jours) neo_nd98b***

**Pouvez-vous donner des détails sur le tracé de fond** : non = 0, oui = 1 l__l **neo_nd101**

Si oui, **tracé de fond** : Normal : non = 0, oui 1 l__l **neo_nd102**

Dysmature* : non = 0, oui = 1 l__l **neo_nd103**

Mal organisé** : non = 0, oui = 1 l__l **neo_nd104**

Trop discontinu*** : non = 0, oui = 1 l__l **neo_nd105**

Tracé plat : non = 0, oui = 1 l__l **neo_nd106**

**Figures anormales présentes à l’EEG** : non = 0, oui = 1 l__l **neo_nd107**

Si oui, **pouvez-vous donner des détails sur les figures anormales** : non = 0, oui = 1 l__l **neo_nd108**

Si oui, **figures anormales** : Pointes positives rolandiques type A ≥ 1/min : non = 0, oui = 1 l__l **neo_nd109**

Pointes positives rolandiques type A < 1/min : non = 0, oui = 1 l__l **neo_nd110**

Delta brushes anormales : non = 0, oui = 1 l__l **neo_nd111**

Crises électriques : non = 0, oui = 1 l__l **neo_nd112**

**Conclusion de l’examen** : normal = 1, altérations modérées = 2, altérations sévères = 3 l__l **neo_nd113**

***d_nd88f***

**Synthèse de la surveillance EEG** l__l **neo_nd114**

***d_nd114f***

1 : EEG tous normaux

2 : Altérations modérées transitoires

3 : Altérations persistantes

4 : Altérations sévères

Imagerie par résonnance magnétique (IRM)

**Une IRM a-t-elle été pratiquée** : non = 0, oui = 1 l__l **neo_nd115**

Si oui,

Nombre total d’IRM pratiquées l__l **neo_nd116**

Résultat de la dernière IRM pratiquée : normal = 1, anormal = 2 l__l **neo_nd117**

***d_nd117f***

Si anormal, préciser en clair : …………………………………………………………………… **neo_nd118**

Traitement neurochirurgical

**Dérivation ventriculopéritonéale**: non = 0, oui = 1 l__l **neo_nd119**

Si oui, nombre l__l **neo_nd120**

**Ventriculocisternostomie** : non = 0, oui = 1 l__l **neo_nd121**

**Traitements et soins au nouveau-né**

Antalgie

*Traitement médicamenteux sédatif et/ou antalgique de classe III :*

**L’enfant a-t-il reçu un traitement médicamenteux sédatif et/ou antalgique de classe III** :

non = 0, oui = 1 l__l **neo_ne1**

Si oui, **l’enfant a-t-il reçu au moins une fois l’un des traitements suivants, y compris de courte durée** (ex : extubation) :

**Morphine** : non = 0, oui = 1 l__l **neo_ne2**

Si oui,

Traitement ponctuel : non = 0, oui = 1 l__l **neo_ne3**

Traitement continu : non = 0, oui = 1 l__l **neo_ne4**

Si oui,

Date de début de traitement l__l__l/ l__l__l /l__l__l__l__l **neo_ne5**

*Age au début de traitement (en jours)* ***neo_ne5b***

Nombre cumulé de jours d’administration l__l__l j **neo_ne8**

**Fentanyl** : non = 0, oui = 1 l__l **neo_ne9**

Si oui,

Date de début de traitement l__l__l/ l__l__l /l__l__l__l__l **neo_ne10**

*Age au début de traitement (en jours)* ***neo_ne10b***

Nombre cumulé de jours d’administration l__l__l j **neo_ne13**

**Sufentanil** : non = 0, oui = 1 l__l **neo_ne14**

Si oui,

Date de début de traitement l__l__l/ l__l__l /l__l__l__l__l **neo_ne15**

*Age au début de traitement (en jours)* ***neo_ne15b***

Nombre cumulé de jours d’administration l__l__l j **neo_ne18**

**Midazolam** **(Hypnovel®)** : non = 0, oui = 1 l__l **neo_ne19**

Si oui,

Date de début de traitement l__l__l/ l__l__l /l__l__l__l__l **neo_ne20**

*Age au début de traitement (en jours)* ***neo_ne20b***

Nombre cumulé de jours d’administration l__l__l j **neo_ne23**

**Kétamine** : non = 0, oui = 1 l__l **neo_ne24**

Si oui,

Date de début de traitement l__l__l/ l__l__l /l__l__l__l__l **neo_ne25**

*Age au début de traitement (en jours)* ***neo_ne25b***

Nombre cumulé de jours d’administration l__l__l j **neo_ne28**

**Autre(s)** : non = 0, oui = 1 l__l **neo_ne29**

Si oui, Traitement 1 : Préciser le traitement en clair : **neo_ne30**

Date de début de traitement l__l__l/ l__l__l /l__l__l__l__l **neo_ne31**

*Age au début de traitement (en jours)* ***neo_ne31b***

Nombre cumulé de jours d’administration l__l__l j **neo_ne34**

Traitement 2 : Préciser le traitement en clair : …………………………………………… **neo_ne35**

Date de début de traitement l__l__l/ l__l__l /l__l__l__l__l **neo_ne36**

*Age au début de traitement (en jours)*  ***neo_ne36b***

Nombre cumulé de jours d’administration l__l__l j **neo_ne39**

*Traitement médicamenteux antalgique de classe I ou II :*

**L’enfant a-t-il reçu un traitement médicamenteux sédatif et/ou antalgique de classe I ou II** :

non = 0, oui = 1 l__l **neo_ne40**

Si oui, **l’enfant a-t-il reçu au moins une fois l’un des traitements suivants**:

**Paracétamol oral** : non = 0, oui = 1 l__l **neo_ne41**

Si oui,

Date de début de traitement l__l__l/ l__l__l /l__l__l__l__l **neo_ne42**

*Age au début de traitement (en jours)* ***neo_ne42b***

Nombre cumulé de jours d’administration l__l__l j **neo_ne45**

**Proparacétamol (Perfalgan®)** : non = 0, oui = 1 l__l **neo_ne46**

Si oui,

Date de début de traitement l__l__l/ l__l__l /l__l__l__l__l **neo_ne47**

*Age au début de traitement (en jours)* ***neo_ne47b***

Nombre cumulé de jours d’administration l__l__l j **neo_ne50**

**Nalbuphine (Nubain®)** : non = 0, oui = 1 l__l **neo_ne51**

Si oui,

Date de début de traitement l__l__l/ l__l__l /l__l__l__l__l **neo_ne52**

*Age au début de traitement (en jours)* ***neo_ne52b***

Nombre cumulé de jours d’administration l__l__l j **neo_ne55**

**Codéine** : non = 0, oui = 1 l__l **neo_ne56**

Si oui,

Date de début de traitement l__l__l/ l__l__l /l__l__l__l__l **neo_ne57**

*Age au début de traitement (en jours)*  ***neo_ne57b***

Nombre cumulé de jours d’administration l__l__l j **neo_ne60**

**Autre(s)** : non = 0, oui = 1 l__l **neo_ne61**

Si oui, Préciser en clair : **neo_ne62**

Date de début de traitement l__l__l/ l__l__l /l__l__l__l__l **neo_ne63**

*Age au début de traitement (en jours)* ***neo_ne63b***

Nombre cumulé de jours d’administration l__l__l j **neo_ne66**

**L’enfant a-t-il eu une anesthésie générale au cours d’une chirurgie** : non = 0, oui = 1 l__l **neo_ne67**

Si oui, intervention pour :

Canal artériel : non = 0, oui = 1 l__l **neo_ne68**

ECUN : non = 0, oui = 1 l__l **neo_ne69**

Hernie inguinale : non = 0, oui = 1 l__l **neo_ne70**

Pose de cathéter : non = 0, oui = 1 l__l **neo_ne71**

Autre : non = 0, oui = 1 l__l **neo_ne72**

Si oui, préciser en clair : ………………………………………………………………………… **neo_ne73**

*Pratiques antalgiques en cas d’intubation :*

**L’enfant a-t-il été intubé ou ré-intubé depuis la salle de naissance** : non = 0, oui = 1 l__l **neo_ne74**

Si oui,

Nombre de fois  l__l **neo_ne75**

Intubation le plus souvent : nasale = 1, orale = 2 l__l **neo_ne76**

***d_ne76f***

Absence au moins une fois de sédation/antalgie en cas d’intubation programmée ou semi-urgente :

non = 0, oui = 1 l__l **neo_ne77**

*Apnées et bradycardies :*

**L’enfant a-t-il bénéficié d’un traitement contre les apnées-bradycardies** : non = 0, oui = 1 l__l **neo_ne79**

Si oui,

Par **Caféine** : non = 0, oui = 1 l__l **neo_ne80**

Si oui,

Date de début l__l__l/ l__l__l /l__l__l__l__l **neo_ne81**

*Age au début (en jours)* ***neo_ne81b***

Date d’arrêt  l__l__l/ l__l__l /l__l__l__l__l **neo_ne84**

Age à l’arrêt (en jours)  ***neo_ne84b***

Indication : curatif = 1, prophylactique = 2, prophylactique puis curatif = 3 l__l **neo_ne87**

***d_ne87f***

Par **Doxapram** : non = 0, oui = 1 l__l **neo_ne88**

Si oui,

Date de début l__l__l/ l__l__l /l__l__l__l__l **neo_ne89**

*Age au début (en jours)* ***neo_ne89b***

Date d’arrêt  l__l__l/ l__l__l /l__l__l__l__l **neo_ne92**

Age à l’arrêt (en jours) ***neo_ne92b***

Indication : curatif = 1, prophylactique = 2, prophylactique puis curatif = 3 l__l **neo_ne95**

***d_ne87f***

Par **ventilation ou PPC nasale** : non = 0, oui = 1 l__l **neo_ne96**

Si oui,

Date de début l__l__l/ l__l__l /l__l__l__l__l **neo_ne97**

*Age au début (en jours)*  ***neo_ne97b***

Date d’arrêt  l__l__l/ l__l__l /l__l__l__l__l **neo_ne100**

Age à l’arrêt (en jours)  **neo_ne100b**

Indication : curatif = 1, prophylactique = 2, prophylactique puis curatif = 3 l__l **neo_ne103**

***d_ne87f***

**L’enfant a-t-il été intubé ou réintubé pour des apnées-bradycardies** : non = 0, oui = 1 l__l **neo_ne104**

Si oui, apnées-bradycardies contemporaines d’une infection : non = 0, oui = 1 l__l **neo_ne105**

Hématologie

**Thrombopénie avant J7** : non = 0, oui = 1 l__l **neo_ne106**

Si oui : taux de plaquettes le plus bas avant transfusion l__l__l__l__l__l__l /mm^3^ **neo_ne107**

**Transfusion de concentrés plaquettaires** **avant J7** : non = 0, oui = 1 l__l **neo_ne108**

Si oui,

Indication de la 1^ère^ transfusion :

Prophylactique : non = 0, oui = 1 l__l **neo_ne109**

Thérapeutique (saignement) : non = 0, oui = 1 l__l **neo_ne110**

Prophylactique avant geste invasif : non = 0, oui = 1 l__l **neo_ne111**

**Nombre total de transfusions de concentrés plaquettaires après J7** l__l **neo_ne112**

**Taux d’hémoglobine observé à la naissance**  l__l__l, l__l g/dl **neo_ne113**

Date l__l__l/ l__l__l /l__l__l__l__l **neo_ne114**

*Age (en jours)* ***neo_ne114b***

**Taux d’hémoglobine le plus bas observé pendant l’hospitalisation**  l__l__l, l__l g/dl **neo_ne117**

Date l__l__l/ l__l__l /l__l__l__l__l **neo_ne118**

*Age (en jours)* ***neo_ne118b***

**Taux d’hémoglobine observé à la sortie de l’enfant à domicile**  l__l__l, l__l g/dl **neo_ne121**

Date l__l__l/ l__l__l /l__l__l__l__l **neo_ne122**

*Age (en jours)* ***neo_ne122b***

**Transfusion de culot globulaire** : non = 0, oui = 1 l__l **neo_ne125**

Si oui, nombre de transfusions l__l **neo_ne126**

**Polynucléaires neutrophiles < 1500/mm3 avant J7** : non = 0, oui = 1 l__l **neo_ne127**

**Evènement transfusionnel** : non = 0, oui = 1 l__l **neo_ne128**

Si oui, préciser en clair : **neo_ne128a**

*Politique de prévention de l’anémie :*

**EPO (Neorecormon®) préventif** : non = 0, oui = 1 l__l **neo_ne129**

Si oui,

Date de début l__l__l/ l__l__l /l__l__l__l__l **neo_ne130**

Age au début (en jours) ***neo_ne130b***

Date de fin l__l__l/ l__l__l /l__l__l__l__l **neo_ne133**

*Age à la fin (en jours)* ***neo_ne133b***

Voie d’administration : intraveineux = 1, sous-cutané = 2, les deux = 3 l__l **neo_ne136**

***d_ne136f***

**Fer**: non = 0, oui = 1 l__l **neo_ne137**

Si oui, date de début l__l__l/ l__l__l /l__l__l__l__l **neo_ne138**

*Age au début (en jours)* ***neo_ne138b***

Néphrologie

**Valeur de créatininémie max. atteint** **entre J3 et J7**  l__l__l__l, l__l µmol/l **neo_ne141**

Date l__l__l/ l__l__l /l__l__l__l__l **neo_ne142**

*Age (en jours)* ***neo_ne142b***

**Valeur de créatininémie max. atteint** **après J7**  l__l__l__l, l__l µmol/l **neo_ne145**

Date l__l__l/ l__l__l /l__l__l__l__l **neo_ne146**

*Age (en jours)* ***neo_ne146b***

**Dernière valeur de créatininémie mesurée avant la sortie à domicile** l__l__l__l, l__l µmol/l **neo_ne149**

Date l__l__l/ l__l__l /l__l__l__l__l **neo_ne150**

*Age (en jours)* ***neo_ne150b***

**Echographie rénale pratiquée** : non = 0, oui = 1 l__l **neo_ne153**

Si oui,

Date de la première échographie l__l__l/ l__l__l /l__l__l__l__l **neo_ne154**

*Age à la première échographie (en jours)* ***neo_ne154b***

Résultat, en clair :……………………………………………………………………………………… **neo_ne157**

Date de la dernière échographie disponible l__l__l/ l__l__l /l__l__l__l__l **neo_ne158**

*Age à la dernière échographie (en jours)* ***neo_ne158b***

Résultat, en clair :……………………………………………………………………………………… **neo_ne161**

**Traitement antihypertenseur reçu** : non = 0, oui = 1 l__l **neo_ne162**

Si oui, traitement en clair :………………………………………………………………………… **neo_ne163**

**Notification d’une insuffisance rénale dans les diagnostics portés au cours de**

**l’hospitalisation en néonatologie** : non = 0, oui = 1 l__l **neo_ne164**

Ictère

**Taux max. de bilirubine avant J7**  l__l__l__l µmol/l **neo_ne165**

ou l__l__l__l mg/l **neo_ne166**

**Photothérapie :** non = 0, oui = 1 l__l **neo_ne167**

Natrémie

**Niveau max. de natrémie avant J7** l__l__l__l mmol/l **neo_ne168**

Infection précoce (≤ 72h de vie)

| **Infection néonatale bactérienne**  0 : pas d'infection néonatale bactérienne | |  | **neo_infection_precoce_final** | |
| --- | --- | --- | --- | --- |
| 1 : infection néonatale bactérienne probable (Antibiothérapie débutée avant H72 et durée ≥ 5 jours) | |  | *d_infprecoce* | |
| 2 : infection néonatale bactérienne certaine avec hémoculture ou LCR + avant H72 | |  |  | |
| ***Enfants tronc commun nés vivants et admis en néonat***  ***Non classés : décès avant J5 sans cause infectieuse clairement identifiée*** |  | | |  |

**Date du début de l’épisode infectieux**  l__l__l/ l__l__l /l__l__l__l__l **neo_ne170**

***Age au début de l’épisode infectieux (en jours) neo_ne170b***

**Infection non confirmée** (traitement anti-infectieux ≤ 3 jours) : non = 0, oui = 1 l__l **neo_ne173**

**Infection clinique** (traitement anti-infectieux > 3 jours) : non = 0, oui = 1 l__l **neo_ne174**

**Infection avec confirmation microbiologique** : non = 0, oui = 1 l__l **neo_ne175**

**Identifiée chez** l__l **neo_ne176**

***d_ne176f***

1 : La mère et l'enfant

2 : L'enfant seulement

3 : La mère seulement

**Bactéries** l__l **neo_ne177**

***d_ne177f***

0 : Non identifiée = 0

1 : Strepto B = 1

2 : E Coli = 2

3 : Autre = 3

Si autre, préciser en clair (y compris les associations de germes) :………………… **neo_ne178**

**Virus** : non = 0, oui = 1 l__l **neo_ne179**

Si oui, préciser en clair : ………………………………………………………………………………. **neo_ne180**

**Levures** : non = 0, oui = 1 l__l **neo_ne181**

Si oui, préciser en clair : ………………………………………………………………………………. **neo_ne182**

**Localisation :**

Bactériémie : non = 0, oui = 1 l__l **neo_ne183**

Trachée : non = 0, oui = 1 l__l **neo_ne184**

Liquide gastrique : non = 0, oui = 1 l__l **neo_ne185**

Anus : non = 0, oui = 1 l__l **neo_ne186**

Oreilles : non = 0, oui = 1 l__l **neo_ne187**

Infection locale peau/tissu mou : non = 0, oui 1 l__l **neo_ne188**

Liquide céphalorachidien : non = 0, oui = 1 l__l **neo_ne189**

Autres non = 0, oui = 1 l__l **neo_ne190**

**Résistance du germe**: non = 0, oui = 1 l__l **neo_ne191**

Si oui,

Ampicilline résistant : non = 0, oui = 1 l__l **neo_ne192**

C3G résistant : non = 0, oui = 1 l__l **neo_ne193**

Carbapénème résistant : non = 0, oui = 1 l__l **neo_ne194**

**Valeur maximale de CRP** (protéine C-réactive) **au cours de l’épisode**  l__l__l__l mg/l **neo_ne195**

**Traitement :**

Ampicilline : non = 0, oui = 1 l__l **neo_ne196**

C3G : non = 0, oui = 1 l__l **neo_ne197**

Aminoside : non = 0, oui = 1 l__l **neo_ne198**

Autre : non = 0, oui = 1 l__l **neo_ne199**

Si oui, préciser en clair : ……………………………………………………………………………… **neo_ne200**

**Durée totale du traitement**  l__l__l j **neo_ne201**

Infections secondaires (> 72h de vie) au cours de la prise en charge néonatale

| ***Enfants tronc commun vivants à J3*** | | |
| --- | --- | --- |
| **Bactériémie après H72** | **neo_infection_bacteriemie** | 0 : pas d'infection secondaire |
| ***d_bact*** |  | 1 : infection secondaire probable/possible (Antibiothérapie débutée après H72 ≥ 5 jours) |
|  |  | 2 : bactériémie certaine : hémoculture + après de 72h de vie |
| **Infection secondaire après H72** | **neo_infection_LOS** | 0 : pas d'infection secondaire |
| ***d_los*** |  | 1 : infection secondaire probable/possible (Antibiothérapie débutée après H72 et durée ≥ 5 jours) |
|  |  | 2 : infection secondaire certaine; localisation = hémoculture, trachée,urines, LCR, ou ostéo articulaire |
| **Evenement infectieux après H72 (inclus conjonctivites, atteintes cutanées, ..)** | **neo_infection_secondaire** | 0 : pas d'infection secondaire |
| ***d_evinfec*** |  | 1 : infection secondaire probable/possible (Antibiothérapie débutée après H72 ≥ 5 jours) |
|  |  | 2 : certaine, toute localisation (hormis culture du KT central) |

**Infection secondaire** : non = 0, oui = 1 l__l **neo_ne202**

Si oui, nombre d’épisodes infectieux l__l **neo_ne203**

*1^er^ épisode infectieux secondaire :*

**Date du début de l’épisode infectieux**  l__l__l/ l__l__l /l__l__l__l__l **neo_ne204**

***Age au début de l’épisode infectieux (en jours) neo_ne204b***

**Infection clinique** (= traitement anti-infectieux ≥ 5 jours) : non = 0, oui = 1 l__l **neo_ne207**

**Infection avec confirmation microbiologique** : non = 0, oui = 1 l__l **neo_ne208**

**Germe**  l__l **neo_ne209**

***d_ne209f***

0 : Non identifié

1 : Staphylocoque coagulase négative*

2 : Staphylocoque doré

3 : Entérocoque

4 : Bacille gram négatif (E Coli,…)

Si 4, préciser en clair : …………………………………………………………………… **neo_ne210**

**2 hémocultures*

**Virus** : non = 0, oui = 1 l__l **neo_ne211**

Si oui, lequel l__l **neo_ne212**

***d_ne212f***

1 : VRS / Adenovirus

2 : Rotavirus = 2

3 : Autre

**Infection fongique** : non = 0, oui = 1 l__l **neo_ne213**

Si oui, préciser en clair : ………………………………………………………………………………. **neo_ne214**

**Résistance du germe**: non = 0, oui = 1 l__l **neo_ne215**

Si oui, laquelle l__l **neo_ne216**

***d_ne216f***

1 : Méthicilline-résistant

2 : Vancomycine-résistant

3 : Multi-résistant

Si multi-résistant, préciser en clair : ………………………………………………… **neo_ne217**

**Localisation :**

Bactériémie : non = 0, oui = 1 l__l **neo_ne218**

Trachée (chez l’enfant intubé) : non = 0, oui = 1 l__l **neo_ne219**

Infection locale peau/tissu mou : non = 0, oui 1 l__l **neo_ne220**

Digestive : non =0, oui = 1 l__l **neo_ne221**

Conjonctivite : non = 0, oui = 1 l__l **neo_ne222**

Urinaire : non = 0, oui = 1 l__l **neo_ne223**

Liquide céphalorachidien : non = 0, oui = 1 l__l **neo_ne224**

Ostéo-articulaire : non = 0, oui = 1 l__l **neo_ne225**

Catheter (culture positive) non = 0, oui = 1 l__l **neo_ne226**

Autre non = 0, oui = 1 l__l **neo_ne227**

Si autre, préciser en clair : ……………………………………………………………………………. **neo_ne228**

**Au moment de l’infection, l’enfant :**

Avait un cathéter central : non = 0, oui = 1 l__l **neo_ne229**

Avait une voie veineuse périphérique : non = 0, oui = 1 l__l **neo_ne230**

Avait une sonde urinaire : non = 0, oui = 1 l__l **neo_ne231**

Avait une ventilation mécanique : non = 0, oui = 1 l__l **neo_ne232**

Etait en postopératoire (dans les 5 jours suivant l’intervention) : non = 0, oui = 1 l__l **neo_ne233**

**Valeur maximale de CRP** (protéine C-réactive) **au cours de l’épisode**  l__l__l__l mg/l **neo_ne234**

**PCT (procalcitonine) dosée au cours de l’épisode** : non = 0, oui = 1 l__l **neo_ne235**

Si oui, valeur maximale de la PCT au cours de l’épisode l__l__l, l__l ng/ml **neo_ne236**

**Traitement anti-infectieux :**

C3G : non = 0, oui = 1 l__l **neo_ne237**

Aminoside : non = 0, oui = 1 l__l **neo_ne238**

Vancomycine : non = 0, oui = 1 l__l **neo_ne239**

Carbapénème : non = 0, oui = 1 l__l **neo_ne240**

Triflucan: non = 0, oui = 1 l__l **neo_ne241**

Autres : non = 0, oui = 1 l__l **neo_ne242**

Si oui, préciser en clair : ……………………………………………………………………………… **neo_ne243**

**Durée du traitement** l__l__l j **neo_ne244**

*2^ème^ épisode infectieux secondaire :*

**Date du début de l’épisode infectieux**  l__l__l/ l__l__l /l__l__l__l__l **neo_ne245**

***Age au début de l’épisode infectieux (en jours) neo_ne245b***

**Infection clinique** (= traitement anti-infectieux ≥ 5 jours) : non = 0, oui = 1 l__l **neo_ne248**

**Infection avec confirmation microbiologique** : non = 0, oui = 1 l__l **neo_ne249**

**Germe**  l__l **neo_ne250**

***d_ne209f***

0 : Non identifié

1 : Staphylocoque coagulase négative*

2 : Staphylocoque doré

3 : Entérocoque

4 : Bacille gram négatif (E Coli,…)

**2 hémocultures*

Si 4, préciser en clair : …………………………………………………………………… **neo_ne251**

**Virus** : non = 0, oui = 1 l__l **neo_ne252**

Si oui, lequel l__l **neo_ne253**

***d_ne212f***

1 : VRS / Adenovirus

2 : Rotavirus = 2

3 : Autre

**Infection fongique** : non = 0, oui = 1 l__l **neo_ne254**

Si oui, préciser en clair : ……………………………………………………………………………… **neo_ne255**

**Résistance du germe**: non = 0, oui = 1 l__l **neo_ne256**

Si oui, laquelle l__l **neo_ne257**

***d_ne216f***

1 : Méthicilline-résistant

2 : Vancomycine-résistant

3 : Multi-résistant

Si multi-résistant, préciser en clair : ……………………………………………………… **neo_ne258**

**Localisation :**

Bactériémie : non = 0, oui = 1 l__l **neo_ne259**

Trachée (chez l’enfant intubé) : non = 0, oui = 1 l__l **neo_ne260**

Infection locale peau/tissu mou : non = 0, oui 1 l__l **neo_ne261**

Digestive : non =0, oui = 1 l__l **neo_ne262**

Conjonctivite : non = 0, oui = 1 l__l **neo_ne263**

Urinaire : non = 0, oui = 1 l__l **neo_ne264**

Liquide céphalorachidien : non = 0, oui = 1 l__l **neo_ne265**

Ostéo-articulaire : non = 0, oui = 1 l__l **neo_ne266**

Catheter (culture positive) non = 0, oui = 1 l__l **neo_ne267**

Autre non = 0, oui = 1 l__l **neo_ne268**

Si autre, préciser en clair : …………………………………………………………………………… **neo_ne269**

**Au moment de l’infection, l’enfant :**

Avait un cathéter central : non = 0, oui = 1 l__l **neo_ne270**

Avait une voie veineuse périphérique : non = 0, oui = 1 l__l **neo_ne271**

Avait une sonde urinaire : non = 0, oui = 1 l__l **neo_ne272**

Avait une ventilation mécanique : non = 0, oui = 1 l__l **neo_ne273**

Etait en postopératoire (dans les 5 jours suivant l’intervention) : non = 0, oui = 1 l__l **neo_ne274**

**Valeur maximale de CRP** (protéine C-réactive) **au cours de l’épisode**  l__l__l__l mg/l **neo_ne275**

**PCT (procalcitonine) dosée au cours de l’épisode** : non = 0, oui = 1 l__l **neo_ne276**

Si oui, valeur maximale de la PCT au cours de l’épisode l__l__l , l__l ng/ml **neo_ne277**

**Traitement anti-infectieux :**

C3G : non = 0, oui = 1 l__l **neo_ne278**

Aminoside : non = 0, oui = 1 l__l **neo_ne279**

Vancomycine : non = 0, oui = 1 l__l **neo_ne280**

Carbapénème : non = 0, oui = 1 l__l **neo_ne281**

Triflucan: non = 0, oui = 1 l__l **neo_ne282**

Autres : non = 0, oui = 1 l__l **neo_ne283**

Si oui, préciser en clair : ……………………………………………………………………………… **neo_ne284**

**Durée du traitement** l__l__l j **neo_ne285**

*3^ème^ épisode infectieux secondaire :*

**Date du début de l’épisode infectieux**  l__l__l/ l__l__l /l__l__l__l__l **neo_ne286**

***Age au début de l’épisode infectieux (en jours) neo_ne286b***

**Infection clinique** (= traitement anti-infectieux ≥ 5 jours) : non = 0, oui = 1 l__l **neo_ne289**

**Infection avec confirmation microbiologique** : non = 0, oui = 1 l__l **neo_ne290**

**Germe**  l__l **neo_ne291**

***d_ne209f***

0 : Non identifié

1 : Staphylocoque coagulase négative*

2 : Staphylocoque doré

3 : Entérocoque

4 : Bacille gram négatif (E Coli,…)

**2 hémocultures*

Si 4, préciser en clair : …………………………………………………………………… **neo_ne292**

**Virus** : non = 0, oui = 1 l__l **neo_ne293**

Si oui, lequel l__l **neo_ne294**

***d_ne212f***

1 : VRS / Adenovirus

2 : Rotavirus = 2

3 : Autre

**Infection fongique** : non = 0, oui = 1 l__l **neo_ne295**

Si oui, préciser en clair : ………………………………………………………………………………. **neo_ne296**

**Résistance du germe**: non = 0, oui = 1 l__l **neo_ne297**

Si oui, laquelle l__l **neo_ne298**

***d_ne216f***

1 : Méthicilline-résistant

2 : Vancomycine-résistant

3 : Multi-résistant

Si multi-résistant, préciser en clair : ……………………………………………………… **neo_ne299**

**Localisation :**

Bactériémie : non = 0, oui = 1 l__l **neo_ne300**

Trachée (chez l’enfant intubé) : non = 0, oui = 1 l__l **neo_ne301**

Infection locale peau/tissu mou : non = 0, oui 1 l__l **neo_ne302**

Digestive : non =0, oui = 1 l__l **neo_ne303**

Conjonctivite : non = 0, oui = 1 l__l **neo_ne304**

Urinaire : non = 0, oui = 1 l__l **neo_ne305**

Liquide céphalorachidien : non = 0, oui = 1 l__l **neo_ne306**

Ostéo-articulaire : non = 0, oui = 1 l__l **neo_ne307**

Catheter (culture positive) non = 0, oui = 1 l__l **neo_ne308**

Autre non = 0, oui = 1 l__l **neo_ne309**

Si autre, préciser en clair : …………………………………………………………………………… **neo_ne310**

**Au moment de l’infection, l’enfant :**

Avait un cathéter central : non = 0, oui = 1 l__l **neo_ne311**

Avait une voie veineuse périphérique : non = 0, oui = 1 l__l **neo_ne312**

Avait une sonde urinaire : non = 0, oui = 1 l__l **neo_ne313**

Avait une ventilation mécanique : non = 0, oui = 1 l__l **neo_ne314**

Etait en postopératoire (dans les 5 jours suivant l’intervention) : non = 0, oui = 1 l__l **neo_ne315**

**Valeur maximale de CRP** (protéine C-réactive) **au cours de l’épisode**  l__l__l__l mg/l **neo_ne316**

**PCT (procalcitonine) dosée au cours de l’épisode** : non = 0, oui = 1 l__l **neo_ne317**

Si oui, valeur maximale de la PCT au cours de l’épisode l__l__l , l__l ng/ml **neo_ne318**

**Traitement anti-infectieux :**

C3G : non = 0, oui = 1 l__l **neo_ne319**

Aminoside : non = 0, oui = 1 l__l **neo_ne320**

Vancomycine : non = 0, oui = 1 l__l **neo_ne321**

Carbapénème : non = 0, oui = 1 l__l **neo_ne322**

Triflucan: non = 0, oui = 1 l__l **neo_ne323**

Autres : non = 0, oui = 1 l__l **neo_ne324**

Si oui, préciser en clair :……………………………………………………………………………… **neo_ne325**

**Durée du traitement** l__l__l j **neo_ne326**

Hémodynamique

*Durant les premières 72 heures de vie :*

**Pression artérielle moyenne (PAM) minimum vérifiée au cours de cette période**  l__l__l mmHg **neo_ne327**

**Traitement à visée hémodynamique** : non = 0, oui = 1 l__l **neo_ne328**

Si oui,

Remplissage vasculaire au cours de cette période : non = 0, oui = 1 l__l **neo_ne329**

Si oui, volume cumulé utilisé au cours de cette période l__l__l__l ml/kg **neo_ne330**

Utilisation de catécholamines au cours de cette période : non = 0, oui = 1 l__l **neo_ne331**

Si oui, préciser :

Dopamine : non = 0, oui = 1 l__l **neo_ne332**

Dobutamine : non = 0, oui = 1 l__l **neo_ne333**

Noradrénaline : non = 0, oui = 1 l__l **neo_ne334**

Utilisation de corticoïdes à visée hémodynamique au cours de cette période : non=0, oui=1 l__l **neo_ne335**

Motif du traitement  l__l **neo_ne336**

***d_ne336f***

1 : Pression artérielle basse isolée

2 : Association de signes cliniques évoquant une hémodynamique insuffisante

3 : Critères échographiques seuls

4 : Critères échographiques et cliniques

5 : Autres critères décisifs (NIRS, lactates, …)

**Echographie cardiaque à visée hémodynamique au cours de cette période** : non = 0, oui = 1 l__l **neo_ne337**

*Après les premières 72 heures de vie :*

**Traitement à visée hémodynamique** : non = 0, oui = 1 l__l **neo_ne338**

Si oui,

Remplissage vasculaire : non = 0, oui = 1 l__l **neo_ne339**

Utilisation de catécholamines : non = 0, oui = 1 l__l **neo_ne340**

Utilisation de corticoïdes à visée hémodynamique : non = 0, oui = 1 l__l **neo_ne341**

*Prise en charge du canal artériel :*

**Exploration du canal artériel par échographie** : non = 0, oui = 1 l__l **neo_ne342**

Si oui,

**Date de la première exploration**  l__l__l/ l__l__l /l__l__l__l__l **neo_ne343**

***Age à la première exploration (en jours) neo_ne343b***

**Indication** :

A titre systématique : non = 0, oui = 1 l__l **neo_ne346**

Sur signes cliniques : non = 0, oui = 1 l__l **neo_ne347**

**Lors des échographies, a-t-on observé les critères suivants** :

Taille du canal artériel > 1,5 mm/kg : non = 0, oui = 1 l__l **neo_ne348**

Aspect tubulaire du flux ductal : non = 0, oui = 1 l__l **neo_ne349**

Elévation du débit pulmonaire (vélocité moyenne >40 cm/sec, vélocité télédiastolique >20 cm/sec) :

non = 0, oui = 1 l__l **neo_ne350**

Signe de bas débit systémique (débit de veine cave supérieure<40ml/kg/mn, flux nul ou reverse flow au doppler cérébral-rénal-mésentérique) : non = 0, oui = 1 l__l **neo_ne351**

**Dernière échographie du canal artériel au cours du séjour néonatal** :

Date l__l__l/ l__l__l /l__l__l__l__l **neo_ne352**

*Age (en jours)* ***neo_ne352b***

Le canal était : ouvert = 1, fermé = 2 l__l **neo_ne355**

***d_ne355f***

**Traitement du canal artériel par AINS** : non = 0, oui = 1 l__l **neo_ne356**

Si oui,

**Date de début de traitement**  l__l__l/ l__l__l /l__l__l__l__l **neo_ne357**

***Age au début du traitement (en jours) neo_ne357b***

**Indication** l__l **neo_ne360**

***d_ne360f***

1 : Préventif

2 : Curatif sur des critères uniquement échographiques

3 : Curatif sur des critères uniquement cliniques

4 : Curatif sur des critères cliniques et échographiques

**Date de début d’une 2^ème^ cure** l__l__l/ l__l__l /l__l__l__l__l **neo_ne361**

***Age au début d’une 2^ème^ cure (en jours) neo_ne361b***

**Indication** l__l **neo_ne364**

***d_ne364f***

1 : Echec de première cure

2 : Réouverture du canal artériel (post sepsis par ex.)

**Traitement du canal artériel par chirurgie** : non = 0, oui = 1 l__l **neo_ne365**

Si oui,

**Date de la chirurgie** l__l__l/ l__l__l /l__l__l__l__l **neo_ne366**

***Age à la chirurgie (en jours) neo_ne366b***

**Indication**  l__l **neo_ne369**

***d_ne369f***

1 : De première intention (y compris si contre-ind au tt med.)

2 : Après échec du traitement médical

**Parmi les critères échographiques suivants, quels sont ceux qui ont contribué à la prise de décision thérapeutique (traitement pharmacologique ou chirurgical de ce canal artériel) :**

Taille du canal artériel > 1,5 mm/kg : non = 0, oui = 1 l__l **neo_ne370**

Aspect tubulaire du flux ductal : non = 0, oui = 1 l__l **neo_ne371**

Elévation du débit pulmonaire (vélocité moyenne >40 cm/s, vélocité télé diastolique >20 cm/s) :

non = 0, oui = 1 l__l **neo_ne372**

Bas débit systémique (débit de veine cave supérieure<40ml/kg/mn, flux nul ou reverse flow au doppler cérébral-rénal-mésentérique) : non = 0, oui = 1 l__l **neo_ne373**

Autre : non = 0, oui = 1 l__l **neo_ne374**

**Soins de développement**

Bilan à J7

*Conditions matérielles et architecturales d’hospitalisation :*

**Chambre** **à J7** l__l **neo_nf1**

***d_nf1f***

1 : D’un enfant

2 : De deux enfants

3 : De trois enfants

4 : De quatre enfants ou plus

5 : Chambre en unité kangourou (avec la mère)

**Utilisation d’un couvre (cache)-couveuse ou d’un drap si le bébé est au berceau pour limiter l’exposition lumineuse à J7** l__l **neo_nf2**

***d_nf2f***

0 : Non

1 : Permanente (arrêt éventuel lors des soins)

2 : Intermittente

3 : Ne sait pas

*Environnement humain :*

**Mise en peau à peau depuis la naissance (de J0 à J7)** : non = 0, oui = 1, ne sait pas = 2 l__l **neo_nf3**

Si oui, date du premier peau à peau  l__l__l/ l__l__l /l__l__l__l__l **neo_nf4**

*Age au premier peau à peau (en jours)* ***neo_nf4b***

Si non, indiquer la raison principale l__l **neo_nf7**

***d_nf7f***

1 : Choix de l’équipe

2 : Manque de disponibilité du personnel soignant

3 : Anxiété parentale

4 : Non disponibilité des parents

5 : Instabilité de l’enfant

6 : Autre

Si autre, préciser en clair : ……………………………………………………………………. **neo_nf8**

| **Pratique du peau à peau pendant la première semaine de vie** | **neo_peau_a_peau** | 1 = peau à peau commencé entre J0 et J3 2 = peau à peau commencé entre J4 et J7 3 = pas de peau à peau entre J0 et J7 |
| --- | --- | --- |
| **Si pas de peau à peau, les raisons** | **neo_raison_pas_pap** | 1 = raisons liés à l'équipe (choix de l'équipe, manque de disponibilité du personnel soignant, on ne vous l'a pas proposé, KTVO, isolement enfant) 2 = raisons liés aux parents (anxiété parentale, non disponibilité des parents, vous ne le souhaitiez pas, mère hospitalisée) 3 = raisons liés à l'enfant (instabilité de l'enfant, l'état de santé de votre enfant ne le permettait pas, autre maladie de l'enfant) 4 = autre raison |

*Pour les enfants sortis vivants du tronc commun.*

*Douleur et inconfort :*

**Evaluation de la douleur ou de l’inconfort de l’enfant réalisée au moins une fois avec une échelle standardisée en dehors d’un soin (entre J0 et J7)** : non = 0, oui = 1, ne sait pas = 2 l__l **neo_nf9**

Si oui,

Score d’EDIN le plus élevé (sur 15) l__l__l /15 **neo_nf10**

Autre méthode d’évaluation : non = 0, oui = 1 l__l **neo_nf11**

Si oui, préciser en clair : …………………………………………………………………………… **neo_nf12**

Score le plus élevé  l__l__l **neo_nf13**

**Evaluation de la douleur ou de l’inconfort de l’enfant réalisée au moins une fois avec une échelle standardisée pendant un soin (entre J0 et J7)**: non = 0, oui = 1, ne sait pas = 2 l__l **neo_nf14**

Si oui,

Score DAN le plus élevé lors d’un soin (sur 10) l__l__l /10 **neo_nf15**

Autre méthode d’évaluation : non = 0, oui = 1 l__l **neo_nf16**

Si oui, préciser en clair : …………………………………………………………………………… **neo_nf17**

Score le plus élevé  l__l__l **neo_nf18**

*Alimentation :*

**Participation des parents à l’alimentation*** : non = 0, oui = 1, ne sait pas = 2 l__l **neo_nf19**

**Soutien et enveloppement du bébé, peau à peau pendant l’alimentation, tétine offerte pendant l’alimentation par sonde*

**Mise au sein** l__l **neo_nf20**

***d_nf20f***

0 : Non

1 : Oui pour contact

2 : Oui pour tétée non nutritive

3 : Oui pour tétée nutritive

4 : Ne sait pas

Si oui, date de la première mise au sein  l__l__l/ l__l__l /l__l__l__l__l **neo_nf21**

*Age à la première mise au sein (en jours)* ***neo_nf21b***

**La mère a-t-elle fait le choix de donner son propre lait à son enfant :**

non = 0, oui =1, ne sait pas = 2 l__l **neo_nf24**

Bilan à J28

*Conditions matérielles et architecturales d’hospitalisation :*

**Chambre** **à J28** l__l **neo_nf25**

***d_nf1f***

1 : D’un enfant

2 : De deux enfants

3 : De trois enfants

4 : De quatre enfants ou plus

5 : Chambre en unité kangourou (avec la mère)

**Utilisation d’un couvre(cache)-couveuse ou d’un drap si le bébé est au berceau pour limiter l’exposition lumineuse à J28** l__l **neo_nf26**

***d_nf2f***

0 : Non

1 : Permanente (arrêt éventuel lors des soins)

2 : Intermittente

3 : Ne sait pas

*Douleur et inconfort :*

**Evaluation de la douleur ou de l’inconfort de l’enfant réalisée au moins une fois, avec une échelle standardisée, en dehors d’un soin (entre J21 et J28)** :

non = 0, oui = 1, ne sait pas = 2 l__l **neo_nf27**

Si oui,

Score d’EDIN le plus élevé (sur 15) l__l__l /15 **neo_nf28**

Autre méthode d’évaluation : non = 0, oui = 1 l__l **neo_nf29**

Si oui, préciser en clair : …………………………………………………………………………… **neo_nf30**

Score le plus élevé  l__l__l **neo_nf31**

**Evaluation de la douleur ou de l’inconfort de l’enfant réalisée au moins une fois avec une échelle standardisée pendant un soin (entre J21 et J28)**:

non = 0, oui = 1, ne sait pas = 2 l__l **neo_nf32**

Si oui,

Score DAN le plus élevé lors d’un soin (sur 10) l__l__l /10 **neo_nf33**

Autre méthode d’évaluation : non = 0, oui = 1 l__l **neo_nf34**

Si oui, préciser en clair : ………………………………………………………………………… **neo_nf35**

Score le plus élevé  l__l__l **neo_nf36**

*Alimentation :*

**Participation des parents à l’alimentation*** : non = 0, oui = 1, ne sait pas = 2 l__l **neo_nf37**

**Soutien et enveloppement du bébé, peau à peau pendant l’alimentation, tétine offerte pendant l’alimentation par sonde*

**Mise au sein** l__l **neo_nf38**

***d_nf20f***

0 : Non

1 : Oui pour contact

2 : Oui pour tétée non nutritive

3 : Oui pour tétée nutritive

4 : Ne sait pas

**La mère a-t-elle fait le choix de donner son propre lait à son enfant :**

non = 0, oui =1, ne sait pas = 2 l__l **neo_nf39**

Bilan depuis la naissance

**L’enfant a-t-il bénéficié depuis sa naissance d’un programme formalisé de soins de** **développement avec suivi particulier** : non = 0, méthode Bullinger = 1, NIDCAP® = 2 l__l **neo_nf40**

***d_nf40f***

Si NIDCAP®,

Nombre d’observations réalisées entre la naissance et S36 l__l__l **neo_nf41**

Date de la première observation terme l__l__l/ l__l__l /l__l__l__l__l **neo_nf42**

*Age à la première observation terme (en jours)* ***neo_nf42b***

**Combien de nuits l’enfant a-t-il passé en chambre avec sa mère avant la sortie à domicile ?**  l__l__l **neo_nf45**

**L’enfant a-t-il bénéficié depuis sa naissance de stimulations de l’oralité** : non = 0, oui = 1 l__l **neo_nf46**

**Nutrition**

Bilan à J3

**Poids ce jour**  l__l__l__l__l g **neo_ng1**

**Poids utilisé pour les calculs d’apport parentéral à J3**  l__l__l__l__l g **neo_ng2**

**Taille de naissance**  l__l__l cm **neo_ng3**

**PC de naissance**  l__l__l cm **neo_pcnce**

*Apport entéral prescrit :*

**Date** **du début de l’alimentation entérale si commencée dans ces 3 jours** l__...__l **neo_ng5**

***Age*** ***au début de l’alimentation entérale si commencée dans ces 3 jours (en jours) neo_ng5b***

**Apport entéral total pour les 24h**  l__l__l__l ml **neo_ng8**

Type de lait n°1 *(cf annexe - types de laits - guide de remplissage des questionnaires)* l__l__l l__l l__l **neo_ng9**

Quantité prescrite pour 24h l__l__l__l ml **neo_ng10**

Type de lait n°2 *(cf annexe - types de laits - guide de remplissage des questionnaires)* l__l__l l__l l__l **neo_ng11**

Quantité prescrite pour 24h l__l__l__l ml **neo_ng12**

*Apport parentéral prescrit :*

**Apport parentéral total pour les 24h, y compris le volume des lipides**  l__l__l__l ml/kg/j **neo_ng13**

*Apport total prescrit (entéral + parentéral) :*

**Apport total pour les 24h**  l__l__l__l__l ml/kg/j **neo_ng14**

Apport en protides l__l__l__l, l__l g/kg/j **neo_ng15**

Apport en glucides l__l__l__l, l__l g/kg/j **neo_ng16**

Apport en lipides l__l__l__l, l__l g/kg/j **neo_ng17**

Bilan à J7

**Poids ce jour**  l__l__l__l__l g **neo_ng18**

**Poids utilisé pour les calculs d’apport parentéral à J7**  l__l__l__l__l g **neo_ng19**

**Taille autour de ce jour**  l__l__l cm **neo_ng20**

**PC autour de ce jour**  l__l__l cm **neo_ng21**

*Apport entéral prescrit :*

**Apport entéral (y compris le « minimal feeding »)** : non = 0, oui = 1 l__l **neo_ng22**

**Date du début de l’alimentation entérale si commencée entre J3 et J7** l__...__l **neo_ng23**

***Age au début de l’alimentation entérale si commencée entre J3 et J7 (en jours) neo_ng23b***

**Apport entéral total pour les 24h**  l__l__l__l ml **neo_ng26**

Type de lait n°1 *(cf annexe - types de laits - guide de remplissage des questionnaires)* l__l__l l__l l__l **neo_ng27**

Quantité prescrite pour 24h l__l__l__l ml **neo_ng28**

Type de lait n°2 *(cf annexe - types de laits - guide de remplissage des questionnaires)* l__l__l l__l l__l **neo_ng29**

Quantité prescrite pour 24h l__l__l__l ml **neo_ng30**

*Apport parentéral prescrit :*

**Apport parentéral total pour les 24h**  l__l__l__l ml/kg/j **neo_ng31**

*Apport total prescrit (entéral + parentéral) :*

**Apport total pour les 24h**  l__l__l__l__l ml/kg/j **neo_ng32**

Apport en protides l__l__l__l, l__l g/kg/j **neo_ng33**

Apport en glucides l__l__l__l, l__l g/kg/j **neo_ng34**

Apport en lipides l__l__l__l, l__l g/kg/j **neo_ng35**

**Avez-vous utilisé de l’insuline dans les 7 premiers jours** : non = 0, oui = 1 l__l **neo_ng36**

Si oui, principale indication  l__l **neo_ng37**

***d_ng37f***

1 : Glycémie ≥ 8.5 mmol/l et < 10 mmol/l

2 : Glycémie ≥ 10 mmol/l et < 15 mmol/l

3 : Glycémie ≥ 15 mmol/l

4 : Glycosurie

5 : Autre

Si autre, préciser en clair : **neo_ng38**

**Valeur maximale de glycémie repérée dans les 7 premiers jours**  l__l__l, l__l mmol/l **neo_ng39**

**Transit intestinal considéré comme normal (au moins une selle par jour) :** non = 0, oui = 1 l__l **neo_ng40**

Bilan à J28

**Poids ce jour**  l__l__l__l__l g **neo_ng41**

**Taille autour de ce jour**  l__l__l cm **neo_ng42**

**PC autour de ce jour**  l__l__l cm **neo_ng43**

**Pression artérielle moyenne* vérifiée (PAM)**  l__l__l mmHg **neo_ng44**

**Première pression artérielle moyenne de la journée*

*Apport entéral prescrit :*

**Apport entéral total pour les 24h**  l__l__l__l ml **neo_ng45**

**L’enfant est-il encore alimenté par sonde gastrique**: non = 0, oui = 1 l__l **neo_ng46**

Si non, date de fin de sonde gastrique l__l__l/ l__l__l /l__l__l__l__l **neo_ng47**

*Age à la de fin de sonde gastrique (en jours)* ***neo_ng47b***

**L’enfant tête-t-il directement le sein**: non = 0, oui = 1 l__l **neo_ng50**

Si oui, la tétée représente la totalité de l’alimentation entérale : non = 0, oui = 1 l__l **neo_ng51**

**En cas d’alimentation à la place ou en complément de l’allaitement au sein précisez les laits** (y compris de lactarium, lait de mère personnalisé donné en dehors de la tétée) :

Type de lait n°1 *(cf annexe - types de laits - guide de remplissage des questionnaires)* l__l__l l__l l__l **neo_ng52**

Quantité prescrite pour 24h l__l__l__l ml **neo_ng53**

Type de lait n°2 *(cf annexe - types de laits - guide de remplissage des questionnaires)* l__l__l l__l l__l **neo_ng54**

Quantité prescrite pour 24h l__l__l__l ml **neo_ng55**

*Apport parentéral prescrit :*

**Apport parentéral total pour les 24h**  l__l__l__l ml/kg/j **neo_ng56**

*Apport total prescrit (entéral + parentéral) :*

**Apport total pour les 24h**  l__l__l__l__l ml/kg/j **neo_ng57**

Apport en protides l__l__l__l, l__l g/kg/j **neo_ng58**

Apport en glucides l__l__l__l, l__l g/kg/j **neo_ng59**

Apport en lipides l__l__l__l, l__l g/kg/j **neo_ng60**

**S’il n’y a plus d’apport parentéral, date de fin de perfusion**  l__...__l **neo_ng61**

***Age à la de fin de perfusion (en jours) neo_ng61b***

**Avez-vous utilisé de l’insuline entre J8 et J28** : non = 0, oui = 1 l__l **neo_ng64**

Bilan à S36

**Poids autour de la date du bilan**  l__l__l__l__l g **neo_ng65**

**Taille autour de la date du bilan**  l__l__l cm **neo_ng66**

**PC autour de la date du bilan**  l__l__l cm **neo_ng67**

*Apport entéral prescrit :*

**L’enfant est-il encore alimenté par sonde gastrique**: non = 0, oui = 1 l__l **neo_ng68**

Si non et si concerné, date de fin gastrique de sonde l__...__l **neo_ng69**

*Age à la fin gastrique de sonde (en jours)* ***neo_ng69b***

**L’enfant tête-t-il directement le sein** : non = 0, oui = 1 l__l **neo_ng72**

Si oui, la tétée représente la totalité de l’alimentation entérale : non = 0, oui = 1 l__l **neo_ng73**

**En cas d’alimentation à la place ou en complément de l’allaitement au sein précisez les laits** (y compris de lactarium, lait de mère personnalisé donné en dehors de la tétée) :

Type de lait n°1 *(cf annexe - types de laits - guide de remplissage des questionnaires)* l__l__l l__l l__l **neo_ng74**

Quantité prescrite pour 24h l__l__l__l ml **neo_ng75**

Type de lait n°2 *(cf annexe - types de laits - guide de remplissage des questionnaires)* l__l__l l__l l__l **neo_ng76**

Quantité prescrite pour 24h l__l__l__l ml **neo_ng77**

*Apport parentéral prescrit :*

**Apport parentéral total pour les 24h**  l__l__l__l ml/kg/j **neo_ng78**

*Apport total prescrit (entéral + parentéral) :*

**Apport total pour les 24h** l__l__l__l ml/kg/j **neo_ng79**

**L’enfant a-t-il encore un cathéter central**: non = 0, oui = 1 l__l **neo_ng80**

Si non, date de retrait du dernier cathéter central l__l__l/ l__l__l /l__l__l__l__l **neo_ng81**

*Age au retrait du dernier cathéter central (en jours)*  ***neo_ng81b***

Bilan depuis la naissance

*Cathéters centraux :*

**Depuis la naissance, combien de cathéters centraux ont été mis en place**  l__l **neo_ng84**

**Accident de cathétérisme central** : non = 0, oui = 1 l__l **neo_ng85**

Si oui :

Thrombose vasculaire : non = 0, oui = 1 l__l **neo_ng86**

Si oui, traitement : non = 0, héparine = 1, fibrinolyse = 2 l__l **neo_ng87**

***d_ng87f***

Épanchement pleural : non = 0, oui = 1 l__l **neo_ng88**

Epanchement péritonéal : non = 0, oui = 1 l__l **neo_ng89**

Tamponnade : non = 0, oui = 1 l__l **neo_ng90**

Autre : non = 0, oui= 1 l__l **neo_ng90a**

Si oui, préciser en clair : **neo_ng90b**

**Combien de complications liées aux cathéters centraux ont été observées**  l__l **neo_ng91**

Nombre d’accidents de type thrombose intravasculaire l__l **neo_ng92**

Nombre de retraits pour cathéter occlus l__l **neo_ng93**

Nombre d’accidents de perfusion péricardique l__l **neo_ng94**

Autre : non = 0, oui= 1 l__l **neo_ng95**

Si oui, préciser en clair : …………………………………………………………………………… **neo_ng96**

*Troubles digestifs/entérocolite ulcéro nécrosante :*

**Perforation intestinale spontanée** : non = 0, oui = 1 l__l **neo_ng97**

Si oui,

Date l__l__l/ l__l__l /l__l__l__l__l **neo_ng98**

*Age (en jours)* ***neo_ng98b***

Chirurgie : non = 0, oui = 1 l__l **neo_ng101**

Si oui, date de la chirurgie l__l__l/ l__l__l /l__l__l__l__l **neo_ng102**

*Age à la chirurgie (en jours)* ***neo_ng102b***

**Entérocolite ulcéro nécrosante** : non = 0, oui = 1 l__l **neo_ng105**

Si oui,

Stade II de Bell clinique* : non = 0, oui = 1 l__l **neo_ng106**

**météorisme silencieux ± résidus verts ± défense ± plastron FID ± paroi inflammatoire*

Si oui, date l__l__l/ l__l__l /l__l__l__l__l **neo_ng107**

*Age (en jours)* ***neo_ng107b***

Stade II de Bell radiologique** : non = 0, oui = 1 l__l **neo_ng110**

***dilatation intestinale ± pneumatose intestinale et/ou portale ± épanchement péritonéal*

Si oui, date l__l__l/ l__l__l /l__l__l__l__l **neo_ng111**

*Age (en jours)* ***neo_ng111b***

Stade III de Bell clinique* : non = 0, oui = 1 l__l **neo_ng114**

**hypotension, oligurie, troubles hydro-électrolytiques, ± acidose mixte ± neutropénie ± CIVD ± SDRA, météorisme volumineux, douloureux avec contracture*

Si oui, date l__l__l/ l__l__l /l__l__l__l__l **neo_ng115**

*Age (en jours)*  ***neo_ng115b***

Stade III de Bell radiologique* : non = 0, oui = 1 l__l **neo_ng118**

***Pneumopéritoine de la grande cavité ou localisé*

Si oui, date l__l__l/ l__l__l /l__l__l__l__l **neo_ng119**

*Age (en jours)* ***neo_ng119b***

**Complications diverses**

*En plus des évènements cliniques déjà recueillis dans le questionnaire, cet enfant a-t-il fait l’objet de ces complications liées aux soins :*

**Evènement cutané** : non = 0, oui = 1 l__l **neo_nh1**

Si oui :

Nécrose cutanée sur dispositif de ventilation (CPAP ou sonde trachéale) : non = 0, oui = 1 l__l **neo_nh2**

Nécrose cutanée sur diffusion voie veineuse périphérique : non = 0, oui = 1 l__l **neo_nh3**

Lésion d’origine chimique/électrodes/pansements : non = 0, oui = 1 l__l **neo_nh4**

**Erreur médicamenteuse** : non = 0, oui = 1 l__l **neo_nh5**

Si oui :

Erreur de prescription : non = 0, oui = 1 l__l  **neo_nh6**

Erreur de produit : non = 0, oui = 1 l__l **neo_nh7**

Erreur de préparation : non = 0, oui = 1 l__l **neo_nh8**

Erreur d’administration : non = 0, oui = 1 l__l **neo_nh9**

**Effet secondaire d’un médicament** : non = 0, oui = 1 l__l **neo_nh10**

**Autre(s) pathologie(s) iatrogène(s)** : non = 0, oui = 1 l__l **neo_nh11**

Si oui, préciser en clair :……………………………………………………………………………… **neo_nh12**

**Anomalies congénitales**

**Anomalies congénitales**: non = 0, oui = 1 l__l **neo_ni1**

Si oui, description finale des anomalies congénitales (y compris les syndromes) en précisant pour chacune d’elle la description la plus détaillée possible en clair :

1^ère^ malformation : …………………………………………………………………………………………. **neo_ni2**

2^ème^ malformation : ………………………………………………………………………………………… **neo_ni3**

3^ème^ malformation : ………………………………………………………………………………………… **neo_ni4**

4^ème^ malformation : ………………………………………………………………………………………… **neo_ni5**

5^ème^ malformation : ………………………………………………………………………………………… **neo_ni6**

***Codage CIM10 des anomalies congénitales des variables NEO_NI2 à NEO_NI6 et ENFANT_MP5 à ENFANT_MP7 :***

***|__|__|__|__|__| NEO_CIM10_MALF1***

***|__|__|__|__|__| NEO_CIM10_MALF2***

***|__|__|__|__|__| NEO_CIM10_MALF3***

***|__|__|__|__|__| NEO_CIM10_MALF4***

***|__|__|__|__|__| NEO_CIM10_MALF5***

**Synthèse Malformation pas de malformation=0, au moins 1 malformation majeure=1, uniquement une malformation mineure=2, doute sur une malformation ou sur critère de gravité=9 l__I ENFANT_MALFO_MAJEUR**

*(Concerne les enfants inclus du tronc commun- Variable N.Lelong, registre des malformations congénitales*

**Rétinopathie**

**Rétinopathie recherchée** : non = 0, oui = 1 l__l **neo_nj1**

Si oui,

Avec Retcam = 1, autre méthode = 2 l__l **neo_nj2**

***d_nj2f***

Date de 1^er^ dépistage l__l__l/ l__l__l /l__l__l__l__l **neo_nj3**

*Age au 1^er^ dépistage (en jours)* ***neo_nj3b***

Date du dernier examen l__l__l/ l__l__l /l__l__l__l__l **neo_nj6**

*Age au dernier examen (en jours)* ***neo_nj6b***

Stade le plus sévère de rétinopathie l__l **neo_nj9**

Préciser : œil droit = 1, œil gauche = 2, les deux = 3 l__l **neo_nj10**

***d_nj10f***

**Rétinopathie traitée** : non = 0, laser = 1, cryothérapie = 2 l__l **neo_nj11**

***d_nj11f***

Si traitement : œil droit = 1, œil gauche = 2, les deux = 3 l__l **neo_nj12**

***d_nj12f***

**Audition**

**Dépistage auditif réalisé** : non = 0, oui = 1 l__l **neo_nk1**

Si oui,

OEAP (otoémissions) : non = 0, oui = 1 l__l **neo_nk2**

Si oui examen l__l **neo_nk3**

***d_nk3f***

0 : Normal

1 : Anormal oreille droite

2 : Anormal oreille gauche

3 : Anormal les deux

PEA : non = 0, oui = 1 l__l **neo_nk4**

Si oui examen  l__l **neo_nk5**

***d_nk3f***

0 : Normal

1 : Anormal oreille droite

2 : Anormal oreille gauche

3 : Anormal les deux

**Surdité profonde** (> 90 dB) : non = 0, oreille droite = 1, oreille gauche = 2, des deux côtés = 3 l__l **neo_nk6**

***d_nk6f***

**Vaccination**

**Vaccination initiée en cours d’hospitalisation** : non = 0, oui = 1 l__l **neo_nv1**

Si oui, préciser :

Diphtérie Tétanos Coqueluche Haemophilus : non = 0, oui = 1 l__l **neo_nv2**

Hépatite B : non = 0, oui = 1 l__l **neo_nv3**

Pneumocoque : non = 0, oui = 1 l__l **neo_nv4**

BCG : non = 0, oui = 1 l__l **neo_nv5**

**Synthèse du parcours des enfants**

*Cette partie a pour objectif de décrire les séjours et les transferts des enfants. Les transferts correspondent à des changements d’établissement (ou de site géographique).*

**L’enfant est-il né dans l’établissement dans lequel il a été hospitalisé juste après sa naissance ?**

non = 0, oui = 1 l__l **neo_nl1**

Si oui, aller à la description de son séjour dans le 1^er^ établissement d’hospitalisation, page 34.

| *Enfants tronc commun* |  |  |
| --- | --- | --- |
| Etablissement ou l’enfant a passé ses 48 premières heures consécutives | **unit48** | Numero établissement anonymisé |
| Niveau Etablissement ou l’enfant a passé ses 48 premières heures consécutives | **niveau_unit48** | 1 = établissement de type 1 21 = établissement de type "2A" 22 = établissement de type "2B" 3 = établissement de type 3 |
| Enfant hospitalisé dans le même établissement pendant la 1ère semaine de vie | **etab_J0J7** | 0 = non 1 = oui |
| Si oui, *(les enfants tranférés dans les 48 ères heures de vie ont un établissement de référence s'ils sont restés dans le même établissement jusqu'à J7.)* | | |
| Numéro anonymisé de l'établissement de référence pour la 1ère semaine d'hopistalisation de l'enfant | **num_etab_J0J7** | Numero établissement anonymisé |
| Niveau de l'établissement de référence pendant la 1ère semaine d'hospitalisation de l'enfant | **niveau_etab_J0J7** | 1 = établissement de type 1 21 = établissement de type "2A" 22 = établissement de type "2B" 3 = établissement de type 3 |
| Nbre de transferts | **Neo_transf** | Nbre de transferts |

Si non, décrire son transfert initial depuis la maternité.

1. *Transfert initial :*

**Etablissement de naissance : nom de la maternité, ville, n° département, en clair :**

………………………………………………………………………………………………………… **enr_etabnaiss**

**Numéro de finess=** **enr_finessnaiss**

**N° établissement anonymisé= enr_numetabnaiss**

**Niveau= enr_niveau2naiss**

**Date du départ**  l__l__l/ l__l__l /l__l__l__l__l **neo_nl5**

***Age au départ (en jours) neo_nl5b***

**Transport** l__l **neo_nl8**

***d_nl8f***

1 : SMUR route

2 : SMUR hélicoptère

3 : Transfert infirmier inter-hospitalier (TIIH)

4 : Autre

Si autre, préciser en clair : …………………………………………………………………….. **neo_nl9**

**Transfert** : ascendant* = 1, descendant = 2, autre = 3 l__l **neo_nl10**

**Ascendant = besoins de plus de soins ou d’un plateau technique adapté à la pathologie*

***d_nl10f***

**Motif(s) de transfert :**

*Noter tous les motifs qui ont conduit au transfert.*

**Pathologie(s) :**

Prématurité : non = 0, oui = 1 l__l **neo_nl11**

Retard de croissance intra-utérin (RCIU) : non = 0, oui = 1 l__l **neo_nl12**

Détresse respiratoire : non = 0, oui = 1 l__l **neo_nl13**

Malformation(s) : non = 0, oui = 1 l__l **neo_nl14**

**Besoin de soins complémentaires :**

Chirurgie : non = 0, oui = 1 l__l **neo_nl15**

Besoin d’une alimentation parentérale : non = 0, oui = 1 l__l **neo_nl16**

**Organisationnel(s) :**

Manque de place : non = 0, oui = 1 l__l **neo_nl17**

**Autre**: non = 0, oui = 1 l__l **neo_nl18**

Si oui, préciser en clair : ………………………………………………………………………………… **neo_nl19**

1. *Séjour dans le 1^er^ établissement d’hospitalisation :*

**Etablissement : nom de l’établissement, ville, n° département, en clair :**

…………………………………………………………………………………………………… **neo_nl20 neo_nl21**

l__l__l__l **neo_nl22**

**Numéro finess= neo_finess_etab1**

**Niveau= neo_niveau_etab1**

***d_etabniv***

***Code anonyme établissement neo_numetab1***

**Service d’entrée**  l__l **neo_nl23**

***d_nl23***

1 : Réanimation néonatale

2 : Unité de soins intensifs néonatals

3 : Médecine néonatale

4 : Autre

Si autre, préciser en clair : ………………………………………………………………… **neo_nl24**

**Date d’entrée dans le service**  l__l__l/ l__l__l /l__l__l__l__l **neo_nl25**

***Age à l’entrée dans le service (en jours) neo_nl25b***

**Heure d’entrée dans le service**  l__l__l h l__l__l min **neo_nl28 neo_nl29**

***Age à l’entrée dans le 1^er^ service de néonat (en min) neo_nl29b***

**Au cours du séjour dans cet établissement, l’enfant a-t-il été hospitalisé en :**

**Réanimation néonatale** : non = 0, oui = 1 l__l **neo_nl30**

Si oui,

Séjour < 1 jour : non = 0, oui = 1 l__l **neo_nl31**

Si séjour ≥ 1 jour, nombre de jours en réanimation néonatale au total l__l__l__l j **neo_nl32**

Nombre de passages en réanimation néonatale l__l **neo_nl33**

**Soins intensifs néonatals** : non =0, oui = 1 l__l **neo_nl34**

Si oui, séjour < 1 jour : non = 0, oui = 1 l__l **neo_nl35**

Si séjour ≥ 1 jour, nombre de jours en unité de soins intensifs néonatals au total l__l__l__l j **neo_nl36**

**Médecine néonatale**: non = 0, oui = 1 l__l **neo_nl37**

Si oui, séjour < 1 jour : non = 0, oui = 1 l__l **neo_nl38**

Si séjour ≥ 1 jour, nombre de jours en médecine néonatale au total l__l__l__l j **neo_nl39**

**Autre(s) service(s)** : non = 0, oui = 1 l__l **neo_nl40**

Si oui,

Autre service 1, en clair : ……………………………………………………………………………. **neo_nl41**

Nombre de jours dans ce service au total l__l__l__l j **neo_nl42**

Autre service 2, en clair : ……………………………………………………………………………. **neo_nl43**

Nombre de jours dans ce service au total l__l__l__l j **neo_nl44**

Autre service 3, en clair : ……………………………………………………………………………. **neo_nl45**

Nombre de jours dans ce service au total l__l__l__l j **neo_nl46**

**Date de sortie de l’établissement**  l__l__l/ l__l__l /l__l__l__l__l **neo_nl47**

***Age à la sortie de l’établissement (en jours) neo_nl47b***

**Mode de sortie**: vivant = 1, décédé = 2 l__l **neo_nl50**

***d_nl50f***

Si vivant, lieu de sortie  l__l **neo_nl51**

***d_nl51f***

1 : Domicile

2 : Pouponnière ou autre lieu de vie

3 : Transfert dans un autre établissement*

**Ne pas comptabiliser en transfert les allers-retours entre site ou établissement pour des séjours < 24h, par exemple pour examen complémentaire…*

4 : Autre

Si 1 ou 2, passer à la page 41 (« conditions de sortie de l’enfant à domicile »)

Si autre, préciser en clair : …………………………………………………………………… **neo_nl52**

1. *Transfert vers un 2^ème^ établissement d’hospitalisation :*

**Date du départ**  l__l__l/ l__l__l /l__l__l__l__l **neo_nl53**

***Age au départ (en jours) neo_nl53b***

**Service de départ**  l__l **neo_nl56**

***d_nl23f***

1 : Réanimation néonatale

2 : Unité de soins intensifs néonatals

3 : Médecine néonatale

4 : Autre

Si autre, préciser en clair :…………………………………………………………………… **neo_nl57**

**Transport**  l__l **neo_nl58**

***d_nl8f***

1 : SMUR route

2 : SMUR hélicoptère

3 : Transfert infirmier inter-hospitalier (TIIH)

4 : Autre

Si autre, préciser en clair : ………………………………………………………………….. **neo_nl59**

**Transfert** : ascendant* = 1, descendant = 2, autre = 3 l__l **neo_nl60**

**Ascendant = besoins de plus de soins ou d’un plateau technique adapté à la pathologie*

***d_nl10f***

**Motif(s) de transfert :**

*Noter tous les motifs qui ont conduit au transfert.*

**Pathologie(s) :**

Prématurité : non = 0, oui = 1 l__l **neo_nl61**

Retard de croissance intra-utérin (RCIU) : non = 0, oui = 1 l__l **neo_nl62**

Détresse respiratoire : non = 0, oui = 1 l__l **neo_nl63**

Malformation(s) : non = 0, oui = 1 l__l **neo_nl64**

Aggravation d’une pathologie : non = 0, oui = 1 l__l **neo_nl65**

Si oui, laquelle en clair :………………………………………………………………………………….. **neo_nl66**

**Besoin de soins complémentaires :**

Chirurgie : non = 0, oui = 1 l__l **neo_nl67**

Besoin d’une alimentation parentérale : non = 0, oui = 1 l__l **neo_nl68**

**Organisationnel(s) :**

Manque de place : non = 0, oui = 1 l__l **neo_nl69**

Rapprochement domicile : non = 0, oui = 1 l__l **neo_nl70**

**Autre** : non = 0, oui = 1 l__l **neo_nl71**

Si oui, préciser en clair : ………………………………………………………………………………… **neo_nl72**

1. *Séjour dans le 2^ème^ établissement d’hospitalisation :*

*Ne pas comptabiliser les séjours < 24h de type « allers-retours » entre 2 sites ou 2 établissements pour réalisation par exemple d’un examen complémentaire.*

**Etablissement : nom de l’établissement, ville, n° département, en clair :**

…………………………………………………………………………………………………… **neo_nl73 neo_nl74**

l__l__l__l **neo_nl75**

**Numéro finess= neo_finess_etab2**

**Niveau= neo_niveau_etab2**

***Code anonyme établissement neo_numetab2***

**Service d’entrée**  l__l **neo_nl76**

***d_nl23f***

1 : Réanimation néonatale

2 : Unité de soins intensifs néonatals

3 : Médecine néonatale

4 : Autre

Si autre, préciser en clair : ……………………………………………………………….. **neo_nl77**

**Date d’entrée dans le service**  l__l__l/ l__l__l /l__l__l__l__l **neo_nl78**

***Age à l’entrée dans le service (en jours) neo_nl78b***

**Au cours du séjour dans cet établissement, l’enfant a-t-il été hospitalisé en :**

**Réanimation néonatale** : non = 0, oui = 1 l__l **neo_nl81**

Si oui,

Séjour < 1 jour : non = 0, oui = 1 l__l **neo_nl82**

Si séjour ≥ 1 jour, nombre de jours en réanimation néonatale au total l__l__l__l j **neo_nl83**

Nombre de passages en réanimation néonatale l__l **neo_nl84**

**Soins intensifs néonatals** : non =0, oui = 1 l__l **neo_nl85**

Si oui, séjour < 1 jour : non = 0, oui = 1 l__l **neo_nl86**

Si séjour ≥ 1 jour, nombre de jours en unité de soins intensifs néonatals au total l__l__l__l j **neo_nl87**

**Médecine néonatale**: non = 0, oui = 1 l__l **neo_nl88**

Si oui, séjour < 1 jour : non = 0, oui = 1 l__l **neo_nl89**

Si séjour ≥ 1 jour, nombre de jours en médecine néonatale au total l__l__l__l j **neo_nl90**

**Autre(s) service(s)** : non = 0, oui = 1 l__l **neo_nl91**

Si oui,

Autre service 1, en clair : ……………………………………………………………………………. **neo_nl92**

Nombre de jours dans ce service au total l__l__l__l j **neo_nl93**

Autre service 2, en clair : ……………………………………………………………………………. **neo_nl94**

Nombre de jours dans ce service au total l__l__l__l j **neo_nl95**

Autre service 3, en clair : ……………………………………………………………………………. **neo_nl96**

Nombre de jours dans ce service au total l__l__l__l j **neo_nl97**

**Date de sortie de l’établissement**  l__l__l/ l__l__l /l__l__l__l__l **neo_nl98**

***Age à la sortie de l’établissement (en jours) neo_nl98b***

**Mode de sortie**: vivant = 1, décédé = 2 l__l **neo_nl101**

***d_nl50f***

Si vivant, lieu de sortie  l__l **neo_nl102**

***d_nl51f***

1 : Domicile

2 : Pouponnière ou autre lieu de vie

3 : Transfert dans un autre établissement*

**Ne pas comptabiliser en transfert les allers-retours entre site ou établissement pour des séjours < 24h, par exemple pour examen complémentaire…*

4 : Autre

Si 1 ou 2, passer à la page 41 (« conditions de sortie de l’enfant à domicile »)

Si autre, préciser en clair : ……………………………………………………………… **neo_nl103**

1. *Transfert vers un 3^ème^ établissement d’hospitalisation :*

**Nom établissement de départ** en clair ……………………………………………………………… **neo_nl104**

**Ville établissement de départ** en clair ……………………………………………………………… **neo_nl105**

**N° département établissement de départ** en clair ………………………………………………… **neo_nl106**

**Service de départ**  l__l **neo_nl110**

***d_nl23f***

1 : Réanimation néonatale

2 : Unité de soins intensifs néonatals

3 : Médecine néonatale

4 : Autre

Si autre, préciser en clair :………………………………………………………………… **neo_nl111**

**Transport**  l__l **neo_nl112**

***d_nl8f***

1 : SMUR route

2 : SMUR hélicoptère

3 : Transfert infirmier inter-hospitalier (TIIH)

4 : Autre

Si autre, préciser en clair : ……………………………………………………………….. **neo_nl113**

**Transfert** : ascendant* = 1, descendant = 2, autre = 3 l__l **neo_nl114**

**Ascendant = besoins de plus de soins ou d’un plateau technique adapté à la pathologie*

***d_nl10f***

**Motif(s) de transfert :**

*Noter tous les motifs qui ont conduit au transfert.*

**Pathologie(s) :**

Prématurité : non = 0, oui = 1 l__l **neo_nl115**

Retard de croissance intra-utérin (RCIU) : non = 0, oui = 1 l__l **neo_nl116**

Détresse respiratoire : non = 0, oui = 1 l__l **neo_nl117**

Malformation(s) : non = 0, oui = 1 l__l **neo_nl118**

Aggravation d’une pathologie : non = 0, oui = 1 l__l **neo_nl119**

Si oui, laquelle en clair :………………………………………………………………………………. **neo_nl120**

**Besoin de soins complémentaires :**

Chirurgie : non = 0, oui = 1 l__l **neo_nl121**

Besoin d’une alimentation parentérale : non = 0, oui = 1 l__l **neo_nl122**

**Organisationnel(s) :**

Manque de place : non = 0, oui = 1 l__l **neo_nl123**

Rapprochement domicile : non = 0, oui = 1 l__l **neo_nl124**

**Autre** : non = 0, oui = 1 l__l **neo_nl125**

Si oui, préciser en clair : ……………………………………………………………………………… **neo_nl126**

1. *Séjour dans le 3^ème^ établissement :*

**Etablissement : nom de l’établissement, ville, n° département, en clair :**

………………………………………………………………………………………………… **neo_nl127 neo_nl128**

l__l__l__l **neo_nl129**

**Numéro finess= neo_finess_etab3**

**Niveau= neo_niveau_etab3 *d_etabniv***

***Code anonyme établissement neo_numetab3***

**Service d’entrée**  l__l **neo_nl130**

***d_nl23f***

1 : Réanimation néonatale

2 : Unité de soins intensifs néonatals

3 : Médecine néonatale

4 : Autre

Si autre, préciser en clair : …………………………………………………………………. **neo_nl131**

**Date d’entrée dans le service**  l__l__l/ l__l__l /l__l__l__l__l **neo_nl132**

***Age à l’entrée dans le service (en jours) neo_nl132b***

**Au cours du séjour dans cet établissement, l’enfant a-t-il été hospitalisé en :**

**Réanimation néonatale** : non = 0, oui = 1 l__l **neo_nl135**

Si oui,

Séjour < 1 jour : non = 0, oui = 1 l__l **neo_nl136**

Si séjour ≥ 1 jour, nombre de jours en réanimation néonatale au total l__l__l__l j **neo_nl137**

Nombre de passages en réanimation néonatale l__l **neo_nl138**

**Soins intensifs néonatals** : non =0, oui = 1 l__l **neo_nl139**

Si oui, séjour < 1 jour : non = 0, oui = 1 l__l **neo_nl140**

Si séjour ≥ 1 jour, nombre de jours en unité de soins intensifs néonatals au total l__l__l__l j **neo_nl141**

**Médecine néonatale**: non = 0, oui = 1 l__l **neo_nl142**

Si oui, séjour < 1 jour : non = 0, oui = 1 l__l **neo_nl143**

Si séjour ≥ 1 jour, nombre de jours en médecine néonatale au total l__l__l__l j **neo_nl144**

**Autre(s) service(s)** : non = 0, oui = 1 l__l **neo_nl145**

Si oui,

Autre service 1, en clair : ………………………………………………………………………… **neo_nl146**

Nombre de jours dans ce service au total l__l__l__l j **neo_nl147**

Autre service 2, en clair : ………………………………………………………………………… **neo_nl148**

Nombre de jours dans ce service au total l__l__l__l j **neo_nl149**

Autre service 3, en clair : ………………………………………………………………………… **neo_nl150**

Nombre de jours dans ce service au total l__l__l__l j **neo_nl151**

**Date de sortie de l’établissement**  l__l__l/ l__l__l /l__l__l__l__l **neo_nl152**

***Age à la sortie de l’établissement (en jours) neo_nl152b***

**Mode de sortie**: vivant = 1, décédé = 2 l__l **neo_nl155**

***d_nl50f***

Si vivant, lieu de sortie  l__l **neo_nl156**

***d_nl51f***

1 : Domicile

2 : Pouponnière ou autre lieu de vie

3 : Transfert dans un autre établissement*

**Ne pas comptabiliser en transfert les allers-retours entre site ou établissement pour des séjours < 24h, par exemple pour examen complémentaire…*

4 : Autre

Si 1 ou 2, passer à la page 41 (« conditions de sortie de l’enfant à domicile »)

Si 4, préciser en clair :  **neo_nl157**

1. *Transfert vers un 4^ème^ établissement d’hospitalisation :*

**Nom établissement de départ** en clair ……………………………………………………………… **neo_nl170**

**Ville établissement de départ** en clair ……………………………………………………………… **neo_nl171**

**N° département établissement** de départ en clair ………………………………………………… **neo_nl172**

**Date du départ**  l__l__l/ l__l__l /l__l__l__l__l **neo_nl173**

***Age au départ (en jours) neo_nl173b***

**Service de départ**  l__l **neo_nl175**

***d_nl23f***

1 : Réanimation néonatale

2 : Unité de soins intensifs néonatals

3 : Médecine néonatale

4 : Autre

Si autre, préciser en clair :………………………………………………………………… **neo_nl176**

**Transport**  l__l **neo_nl177**

***d_nl8f***

1 : SMUR route

2 : SMUR hélicoptère

3 : Transfert infirmier inter-hospitalier (TIIH)

4 : Autre

Si autre, préciser en clair : ……………………………………………………………….. **neo_nl178**

**Transfert** : ascendant* = 1, descendant = 2, autre = 3 l__l **neo_nl179**

**Ascendant = besoins de plus de soins ou d’un plateau technique adapté à la pathologie*

***d_nl10f***

**Motif(s) de transfert :**

*Noter tous les motifs qui ont conduit au transfert.*

**Pathologie(s) :**

Prématurité : non = 0, oui = 1 l__l **neo_nl180**

Retard de croissance intra-utérin (RCIU) : non = 0, oui = 1 l__l **neo_nl181**

Détresse respiratoire : non = 0, oui = 1 l__l **neo_nl182**

Malformation(s) : non = 0, oui = 1 l__l **neo_nl183**

Aggravation d’une pathologie : non = 0, oui = 1 l__l **neo_nl184**

Si oui, laquelle en clair :………………………………………………………………………………. **neo_nl185**

**Besoin de soins complémentaires :**

Chirurgie : non = 0, oui = 1 l__l **neo_nl186**

Besoin d’une alimentation parentérale : non = 0, oui = 1 l__l **neo_nl187**

**Organisationnel(s) :**

Manque de place : non = 0, oui = 1 l__l **neo_nl188**

Rapprochement domicile : non = 0, oui = 1 l__l **neo_nl189**

**Autre** : non = 0, oui = 1 l__l **neo_nl190**

Si oui, préciser en clair : ……………………………………………………………………………… **neo_nl191**

1. *Séjour dans le 4^ème^ établissement :*

**Etablissement : nom de l’établissement, ville, n° département, en clair :**

………………………………………………………………………………………………… **neo_nl192 neo_nl193**

l__l__l__l **neo_nl194**

**Numéro finess= neo_finess_etab4**

**Niveau= neo_niveau_etab4 *d_etabniv***

***Code anonyme établissement neo_numetab4***

**Service d’entrée**  l__l **neo_nl195**

***d_nl23f***

1 : Réanimation néonatale

2 : Unité de soins intensifs néonatals

3 : Médecine néonatale

4 : Autre

Si autre, préciser en clair : …………………………………………………………………. **neo_nl196**

**Date d’entrée dans le service**  l__l__l/ l__l__l /l__l__l__l__l **neo_nl197**

***Age à l’entrée dans le service (en jours) neo_nl197b***

**Au cours du séjour dans cet établissement, l’enfant a-t-il été hospitalisé en :**

**Réanimation néonatale** : non = 0, oui = 1 l__l **neo_nl199**

Si oui,

Séjour < 1 jour : non = 0, oui = 1 l__l **neo_nl200**

Si séjour ≥ 1 jour, nombre de jours en réanimation néonatale au total l__l__l__l j **neo_nl201**

Nombre de passages en réanimation néonatale l__l **neo_nl202**

**Soins intensifs néonatals** : non =0, oui = 1 l__l **neo_nl203**

Si oui, séjour < 1 jour : non = 0, oui = 1 l__l **neo_nl204**

Si séjour ≥ 1 jour, nombre de jours en unité de soins intensifs néonatals au total l__l__l__l j **neo_nl205**

**Médecine néonatale**: non = 0, oui = 1 l__l **neo_nl206**

Si oui, séjour < 1 jour : non = 0, oui = 1 l__l **neo_nl207**

Si séjour ≥ 1 jour, nombre de jours en médecine néonatale au total l__l__l__l j **neo_nl208**

**Autre(s) service(s)** : non = 0, oui = 1 l__l **neo_nl209**

Si oui,

Autre service 1, en clair : ………………………………………………………………………… **neo_nl210**

Nombre de jours dans ce service au total l__l__l__l j **neo_nl211**

Autre service 2, en clair : ………………………………………………………………………… **neo_nl212**

Nombre de jours dans ce service au total l__l__l__l j **neo_nl213**

Autre service 3, en clair : ………………………………………………………………………… **neo_nl214**

Nombre de jours dans ce service au total l__l__l__l j **neo_nl215**

**Date de sortie de l’établissement**  l__l__l/ l__l__l /l__l__l__l__l **neo_nl216**

***Age à la sortie de l’établissement (en jours) neo_nl216b***

**Mode de sortie**: vivant = 1, décédé = 2 l__l **neo_nl218**

***d_nl50f***

Si vivant, lieu de sortie  l__l **neo_nl219**

***d_nl51f***

1 : Domicile

2 : Pouponnière ou autre lieu de vie

3 : Transfert dans un autre établissement*

**Ne pas comptabiliser en transfert les allers-retours entre site ou établissement pour des séjours < 24h, par exemple pour examen complémentaire…*

4 : Autre

Si 1 ou 2, passer à la page 41 (« conditions de sortie de l’enfant à domicile »)

Si 4, préciser en clair : **neo_nl220**

1. *Transfert vers un 5^ème^ établissement d’hospitalisation :*

**Nom établissement de départ** en clair ……………………………………………………………… **neo_nl221**

**Ville établissement de départ** en clair ……………………………………………………………… **neo_nl222**

**N° département établissement** de départ en clair ………………………………………………… **neo_nl223**

**Date du départ**  l__l__l/ l__l__l /l__l__l__l__l **neo_nl224**

***Age au départ (en jours) neo_nl224b***

**Service de départ**  l__l **neo_nl226**

***d_nl23f***

1 : Réanimation néonatale

2 : Unité de soins intensifs néonatals

3 : Médecine néonatale

4 : Autre

Si autre, préciser en clair :………………………………………………………………… **neo_nl227**

**Transport**  l__l **neo_nl228**

***d_nl8f***

1 : SMUR route

2 : SMUR hélicoptère

3 : Transfert infirmier inter-hospitalier (TIIH)

4 : Autre

Si autre, préciser en clair : ……………………………………………………………….. **neo_nl229**

**Transfert** : ascendant* = 1, descendant = 2, autre = 3 l__l **neo_nl230**

**Ascendant = besoins de plus de soins ou d’un plateau technique adapté à la pathologie*

***d_nl10f***

**Motif(s) de transfert :**

*Noter tous les motifs qui ont conduit au transfert.*

**Pathologie(s) :**

Prématurité : non = 0, oui = 1 l__l **neo_nl231**

Retard de croissance intra-utérin (RCIU) : non = 0, oui = 1 l__l **neo_nl232**

Détresse respiratoire : non = 0, oui = 1 l__l **neo_nl233**

Malformation(s) : non = 0, oui = 1 l__l **neo_nl234**

Aggravation d’une pathologie : non = 0, oui = 1 l__l **neo_nl235**

Si oui, laquelle en clair :……………………………………………………………………………… **neo_nl236**

**Besoin de soins complémentaires :**

Chirurgie : non = 0, oui = 1 l__l **neo_nl237**

Besoin d’une alimentation parentérale : non = 0, oui = 1 l__l **neo_nl238**

**Organisationnel(s) :**

Manque de place : non = 0, oui = 1 l__l **neo_nl239**

Rapprochement domicile : non = 0, oui = 1 l__l **neo_n240**

**Autre** : non = 0, oui = 1 l__l **neo_nl241**

Si oui, préciser en clair : ……………………………………………………………………………… **neo_nl242**

1. *Séjour dans le 5^ème^ établissement :*

**Etablissement : nom de l’établissement, ville, n° département, en clair :**

……………………………………………………………………………………………………**neo_nl243 neo_nl244**

l__l__l__l **neo_nl245**

**Numéro finess= neo_finess_etab5**

**Niveau= neo_niveau_etab5 *d_etabniv***

***Code anonyme établissement neo_numetab5***

**Service d’entrée**  l__l **neo_nl246**

***d_nl23f***

1 : Réanimation néonatale

2 : Unité de soins intensifs néonatals

3 : Médecine néonatale

4 : Autre

Si autre, préciser en clair : ………………………………………………………………… **neo_nl247**

**Date d’entrée dans le service**  l__l__l/ l__l__l /l__l__l__l__l **neo_nl248**

***Age à l’entrée dans le service (en jours) neo_nl248b***

**Au cours du séjour dans cet établissement, l’enfant a-t-il été hospitalisé en :**

**Réanimation néonatale** : non = 0, oui = 1 l__l **neo_nl250**

Si oui,

Séjour < 1 jour : non = 0, oui = 1 l__l **neo_nl251**

Si séjour ≥ 1 jour, nombre de jours en réanimation néonatale au total l__l__l__l j **neo_nl252**

Nombre de passages en réanimation néonatale l__l **neo_nl253**

**Soins intensifs néonatals** : non =0, oui = 1 l__l **neo_nl254**

Si oui, séjour < 1 jour : non = 0, oui = 1 l__l **neo_nl255**

Si séjour ≥ 1 jour, nombre de jours en unité de soins intensifs néonatals au total l__l__l__l j **neo_nl256**

**Médecine néonatale**: non = 0, oui = 1 l__l **neo_n257**

Si oui, séjour < 1 jour : non = 0, oui = 1 l__l **neo_nl258**

Si séjour ≥ 1 jour, nombre de jours en médecine néonatale au total l__l__l__l j **neo_nl259**

**Autre(s) service(s)** : non = 0, oui = 1 l__l **neo_nl260**

Si oui,

Autre service 1, en clair : ………………………………………………………………………… **neo_nl261**

Nombre de jours dans ce service au total l__l__l__l j **neo_nl262**

Autre service 2, en clair : ………………………………………………………………………… **neo_nl263**

Nombre de jours dans ce service au total l__l__l__l j **neo_nl264**

Autre service 3, en clair : ………………………………………………………………………… **neo_nl265**

Nombre de jours dans ce service au total l__l__l__l j **neo_nl266**

**Date de sortie de l’établissement**  l__l__l/ l__l__l /l__l__l__l__l **neo_nl267**

***Age à la sortie (en jours) neo_nl267b***

**Mode de sortie**: vivant = 1, décédé = 2 l__l **neo_nl269**

***d_nl50f***

Si vivant, lieu de sortie  l__l **neo_nl270**

***d_nl51f***

1 : Domicile

2 : Pouponnière ou autre lieu de vie

3 : Transfert dans un autre établissement*

**Ne pas comptabiliser en transfert les allers-retours entre site ou établissement pour des séjours < 24h, par exemple pour examen complémentaire…*

4 : Autre

Si 1 ou 2, passer à la page 41 (« conditions de sortie de l’enfant à domicile »)

Si 4, préciser en clair : **neo_nl271**

1. *Transfert vers un 6^ème^ établissement d’hospitalisation :*

**Nom établissement de départ** en clair ……………………………………………………………… **neo_nl272**

**Ville établissement de départ** en clair ……………………………………………………………… **neo_nl273**

**N° département établissement** de départ en clair ………………………………………………… **neo_nl274**

**Date du départ**  l__l__l/ l__l__l /l__l__l__l__l **neo_nl275**

***Age au départ (en jours) neo_nl275b***

**Service de départ**  l__l **neo_nl277**

***d_nl23f***

1 : Réanimation néonatale

2 : Unité de soins intensifs néonatals

3 : Médecine néonatale

4 : Autre

Si autre, préciser en clair :……………………………………………………………… **neo_nl278**

**Transport**  l__l **neo_nl279**

***d_nl8f***

1 : SMUR route

2 : SMUR hélicoptère

3 : Transfert infirmier inter-hospitalier (TIIH)

4 : Autre

Si autre, préciser en clair : ………………………………………………………………. **neo_nl280**

**Transfert** : ascendant* = 1, descendant = 2, autre = 3 l__l **neo_nl281**

**Ascendant = besoins de plus de soins ou d’un plateau technique adapté à la pathologie*

***d_nl10f***

**Motif(s) de transfert :**

*Noter tous les motifs qui ont conduit au transfert.*

**Pathologie(s) :**

Prématurité : non = 0, oui = 1 l__l **neo_nl282**

Retard de croissance intra-utérin (RCIU) : non = 0, oui = 1 l__l **neo_nl283**

Détresse respiratoire : non = 0, oui = 1 l__l **neo_nl284**

Malformation(s) : non = 0, oui = 1 l__l **neo_nl285**

Aggravation d’une pathologie : non = 0, oui = 1 l__l **neo_nl286**

Si oui, laquelle en clair :……………………………………………………………………………… **neo_nl287**

**Besoin de soins complémentaires :**

Chirurgie : non = 0, oui = 1 l__l **neo_nl288**

Besoin d’une alimentation parentérale : non = 0, oui = 1 l__l **neo_nl289**

**Organisationnel(s) :**

Manque de place : non = 0, oui = 1 l__l **neo_nl290**

Rapprochement domicile : non = 0, oui = 1 l__l **neo_n291**

**Autre** : non = 0, oui = 1 l__l **neo_nl292**

Si oui, préciser en clair : ……………………………………………………………………………… **neo_nl293**

1. *Séjour dans le 6^ème^ établissement :*

**Etablissement : nom de l’établissement, ville, n° département, en clair :**

……………………………………………………………………………………………………**neo_nl294 neo_nl295**

l__l__l__l **neo_nl296**

**Numéro finess= neo_finess_etab6**

**Niveau= neo_niveau_etab6 *d_etabniv***

***Code anonyme établissement neo_numetab6***

**Service d’entrée**  l__l **neo_nl297**

***d_nl23f***

1 : Réanimation néonatale

2 : Unité de soins intensifs néonatals

3 : Médecine néonatale

4 : Autre

Si autre, préciser en clair : ………………………………………………………………… **neo_nl298**

**Date d’entrée dans le service**  l__l__l/ l__l__l /l__l__l__l__l **neo_nl299**

***Age à l’entrée dans le service (en jours) neo_nl299b***

**Au cours du séjour dans cet établissement, l’enfant a-t-il été hospitalisé en :**

**Réanimation néonatale** : non = 0, oui = 1 l__l **neo_nl301**

Si oui,

Séjour < 1 jour : non = 0, oui = 1 l__l **neo_nl302**

Si séjour ≥ 1 jour, nombre de jours en réanimation néonatale au total l__l__l__l j **neo_nl303**

Nombre de passages en réanimation néonatale l__l **neo_nl304**

**Soins intensifs néonatals** : non =0, oui = 1 l__l **neo_nl305**

Si oui, séjour < 1 jour : non = 0, oui = 1 l__l **neo_nl306**

Si séjour ≥ 1 jour, nombre de jours en unité de soins intensifs néonatals au total l__l__l__l j **neo_nl307**

**Médecine néonatale**: non = 0, oui = 1 l__l **neo_nl308**

Si oui, séjour < 1 jour : non = 0, oui = 1 l__l **neo_nl309**

Si séjour ≥ 1 jour, nombre de jours en médecine néonatale au total l__l__l__l j **neo_nl310**

**Autre(s) service(s)** : non = 0, oui = 1 l__l **neo_nl311**

Si oui,

Autre service 1, en clair : ………………………………………………………………………… **neo_nl312**

Nombre de jours dans ce service au total l__l__l__l j **neo_nl313**

Autre service 2, en clair : ………………………………………………………………………… **neo_nl314**

Nombre de jours dans ce service au total l__l__l__l j **neo_nl315**

Autre service 3, en clair : ………………………………………………………………………… **neo_nl316**

Nombre de jours dans ce service au total l__l__l__l j **neo_nl317**

**Date de sortie de l’établissement**  l__l__l/ l__l__l /l__l__l__l__l **neo_nl318**

***Age à la sortie (en jours) neo_nl318b***

**Mode de sortie**: vivant = 1, décédé = 2 l__l **neo_nl320**

***d_nl50f***

Si vivant, lieu de sortie  l__l **neo_nl321**

***d_nl51f***

1 : Domicile

2 : Pouponnière ou autre lieu de vie

3 : Transfert dans un autre établissement*

**Ne pas comptabiliser en transfert les allers-retours entre site ou établissement pour des séjours < 24h, par exemple pour examen complémentaire…*

4 : Autre

Si 1 ou 2, passer à la page 41 (« conditions de sortie de l’enfant à domicile »)

Si 4, préciser en clair : **neo_nl322**

**Limitation ou arrêt des traitements actifs**

**Y a-t-il eu une discussion de limitation ou d’arrêt des traitements actifs de réanimation en réanimation néonatale** : non = 0, oui = 1 l__l **neo_nm1**

Si non, aller à la rubrique suivante page 41 ou 43 (« conditions de sortie de l’enfant à domicile »/  « enfant décédé en néonatologie »)

Si oui,

*Processus de prise de la décision :*

**Motif(s) de la discussion** **:**

Traitements devenus inefficaces pour la survie de l’enfant : non = 0, oui = 1 l__l **neo_nm2**

Pronostic de mauvaise qualité de vie future en cas de survie : non = 0, oui = 1 l__l **neo_nm3**

Traitements disproportionnés : lourdeur des traitements actuels ou à entreprendre : non = 0, oui = 1 l__l **neo_nm4**

Si traitements disproportionnés ou mauvais pronostic et maladie incompatible avec une qualité de vie acceptable liés à :

Pathologie neurologique : non = 0, oui = 1 l__l **neo_nm5**

Pathologie respiratoire : non = 0, oui = 1 l__l **neo_nm6**

Pathologie digestive : non = 0, oui = 1 l__l **neo_nm7**

Pathologie multiviscérale : non = 0, oui = 1 l__l **neo_nm8**

Autre : non = 0, oui = 1 l__l **neo_nm9**

Si oui, préciser en clair : ……………………………………………………………………… **neo_nm10**

**Ya-t-il eu une(des) réunion(s) où une limitation ou un arrêt des traitements actifs a été discuté** **pour cet enfant** : non = 0, oui = 1, information non disponible = 2 l__l **neo_nm11**

Si oui,

Nombre de réunions où le cas de cet enfant a été discuté  l__l **neo_nm12**

Le contenu d’une (ou plusieurs) réunion(s) est-il retranscrit dans le dossier : non = 0, oui = 1 l__l **neo_nm13**

**Y a-t-il eu un (ou plusieurs) entretien(s) préalable(s) à la décision avec le ou les parents** :

non = 0, oui = 1 l__l **neo_nm14**

Si oui, avec : le père = 1, la mère = 2, le père et la mère ensemble = 3 l__l **neo_nm15**

***d_nm15f***

**Les parents ont-ils d’emblée exprimé une demande avant toute discussion**: non = 0, oui = 1 l__l **neo_nm16**

**Degré d’implication des parents dans la décision**  l__l **neo_nm17**

***d_nm17f***

1 : Les parents n’ont pas été informés de la prise d’une décision

2 : Les parents ont été informés, sans qu’un avis leur ait été demandé

3 : Les parents ont été informés, leur avis a été recueilli indirectement

4 : Les parents ont été informés et leur accord a été recueilli

**Opinion des parents :**

Poursuite complète des traitements curatifs : non = 0, oui = 1 l__l **neo_nm18**

Abstention, limitation ou interruption médicale : non = 0, oui = 1 l__l **neo_nm19**

S’en remettent à l’avis médical : non = 0, oui = 1 l__l **neo_nm20**

Opposés à l’avis médical : non = 0, oui = 1 l__l **neo_nm21**

Information non disponible dans le dossier : non = 0, oui = 1 l__l **neo_nm22**

Autre : non = 0, oui = 1 l__l **neo_nm23**

Si autre, préciser en clair : ……………………………………………………………………… **neo_nm24**

**Quelle a été la décision** **finale** **:**

Pas de décision possible (manque de consensus ou d’éléments nécessaires) : non = 0, oui = 1 l__l **neo_nm25**

Décision de limitation ou d’arrêt de traitement actif : non = 0, oui = 1 l__l **neo_nm26**

Décision d’administrer des médicaments analgésiques ou sédatifs au risque de provoquer le décès :

non = 0, oui = 1 l__l **neo_nm27**

Décision de poursuite de la réanimation (et instauration de tous les traitements nécessaires, sans limitation) : non = 0, oui = 1 l__l **neo_nm28**

Si décision de limitation ou arrêt de traitement actif :

Abstention en cas d’arrêt cardio-circulatoire : non = 0, oui = 1 l__l **neo_nm29**

Limitation de mise en place de nouveaux traitements (ex : réintubation, chirurgie, drainage, transfusions, etc.) : non = 0, oui = 1 l__l **neo_nm30**

Interruption de traitement(s) en cours (ex : extuber, arrêter l’alimentation artificielle, etc.) :

non = 0, oui = 1 l__l **neo_nm31**

**Motifs médicaux finaux de la décision** **:**

Incertitude diagnostique et/ou pronostique : non = 0, oui = 1 l__l **neo_nm32**

« Projet thérapeutique acceptable » (pas de disproportion avantages/risques à poursuivre les traitements actifs) : non = 0, oui = 1 l__l **neo_nm33**

« Projet thérapeutique inacceptable » (disproportion avantages/risques à poursuivre les traitements actifs) : non = 0, oui = 1 l__l **neo_nm34**

Aucune survie possible « no chance to survive » (NCTS) : non = 0, oui 1 l__l **neo_nm35**

**Date de la prise de décision** **finale**  l__l__l/ l__l__l /l__l__l__l__l **neo_nm36**

***Age à la prise de décision finale (en jours) neo_nm36b***

**Conditions de sortie de l’enfant a domicile**

**Date de sortie**  l__l__l/ l__l__l /l__l__l__l__l **neo_nn1**

***Age à la sortie (en jours) neo_nn1b***

**Poids de sortie**  l__l__l__l__l g **neo_nn4**

**PC de sortie**  l__l__l cm **neo_nn5**

**Taille de sortie**  l__l__l cm **neo_nn6**

**Pression artérielle moyenne* vérifiée (PAM)**  l__l__l mmHg **neo_nn7**

**Première pression artérielle moyenne de la journée*

**Enregistrement cardio-respiratoire particulier en pré-sortie de néonatologie**: non = 0, oui = 1 l__l **neo_nn8**

Si oui, préciser en clair : …………………………………………………………………… **neo_nn9**

**Nombre d’établissements dans lesquels l’enfant a été hospitalisé**  l__l **neo_nn10**

*Traitements à la sortie :*

**Traitement du RGO**: non = 0, oui = 1 l__l **neo_nn11**

Si oui,

Epaississant : non = 0, oui = 1 l__l **neo_nn12**

Prokinétique : non = 0, oui = 1 l__l **neo_nn13**

Anti-acide (type omeprazole) : non = 0, oui = 1 l__l **neo_nn14**

**Traitement des apnées/bradycardies** : non = 0, oui = 1 l__l **neo_nn15**

Si oui,

Caféine : non = 0, oui = 1 l__l **neo_nn16**

Doxapram : non = 0, oui = 1 l__l **neo_nn17**

**O2**: non = 0, oui = 1 l__l **neo_nn18**

**Monitoring**: non = 0, oui = 1 l__l **neo_nn19**

Si oui,

Scope : non = 0, oui = 1 l__l **neo_nn20**

Saturomètre : non = 0, oui = 1 l__l **neo_nn21**

**Traitement antihypertenseur** : non = 0, oui = 1 l__l **neo_nn22**

**Prescription de Synagis®**: non = 0, oui = 1 l__l **neo_nn23**

*Alimentation :*

**L’enfant est-il encore alimenté par sonde gastrique**: non = 0, oui = 1 l__l **neo_nn24**

Si non, date de fin de sonde gastrique l__l__l/ l__l__l /l__l__l__l__l **neo_nn25**

*Age à la fin de sonde gastrique (en jours)* ***neo_nn25b***

**L’enfant tête-t-il directement le sein** : non = 0, oui = 1 l__l **neo_nn28**

Si oui, la tétée représente la totalité de l’alimentation entérale : non = 0, oui = 1 l__l **neo_nn29**

**En cas d’alimentation à la place ou en complément de l’allaitement au sein précisez les laits** (y compris de lactarium, lait de mère personnalisé donné en dehors de la tétée) :

Type de lait n°1 *(cf annexe - types de laits - guide de remplissage des questionnaires)* l__l__l l__l l__l **neo_nn30**

Quantité prescrite pour 24h l__l__l__l ml **neo_nn31**

Type de lait n°2 *(cf annexe - types de laits - guide de remplissage des questionnaires)* l__l__l l__l l__l **neo_nn32**

Quantité prescrite pour 24h l__l__l__l ml **neo_nn33**

| **Allaitement à la sortie de néonatalologie (variable A.Mitha)** | **neo_allaitclasse** | 0 : pas d'allaitement maternel |
| --- | --- | --- |
|  |  | 1 : allaitement mixte (maternel + artificiel) |
|  |  | 2 : allaitement exclusif |

*Suivi de l’enfant :*

**L'enfant sort-il avec une HAD**: non = 0, oui = 1 l__l **neo_nn34**

**Une visite à domicile d’un personnel de la PMI est-elle prévue**: non = 0, oui = 1 l__l **neo_nn35**

**Des informations sur le suivi de l’enfant dans le cadre du réseau ont-elles été données aux parents pendant le séjour**: non = 0, oui = 1 l__l **neo_nn36**

**Les parents ont-ils reçu une liste des services et médecins pouvant assurer le suivi :**

non = 0, oui = 1 l__l **neo_nn37**

**L’enfant va-t-il entrer dans le réseau de suivi**: non = 0, oui = 1 l__l **neo_nn38**

Si non, pourquoi  l__l **neo_nn39**

***d_nn39f***

1 : Pas de réseau de suivi

2 : Non proposé aux parents par oubli

3 : Non proposé, car l’enfant n’entre pas dans les critères

4 : Refus des parents

**Un premier rendez-vous de suivi de l’enfant a-t-il été fixé**: non = 0, oui = 1 l__l **neo_nn40**

Si oui, où  l__l **neo_nn41**

***d_nn41f***

1 : Le service de néonatologie

2 : Un autre service de l’hôpital

3 : Le CAMSP

4 : Un médecin de ville

Si 2 ou 3, préciser lequel en clair :……………………………………………………… **neo_nn42**

**Enfant décédé en réanimation néonatale / néonatologie**

**Enfant décédé**: non = 0, oui = 1 l__l **neo_no1**

Si oui, **le décès fait-il suite à une décision d’arrêt, d’abstention ou de limitation des thérapeutiques de réanimation - sauf phase agonique (processus de décès irréversible)**: non = 0, oui = 1 l__l **neo_no2**

Si non, aller directement à la page 44 à l’item « Date du décès de l’enfant »

**Si oui,**

*Modalités de décès en cas de limitation abstention ou arrêt des thérapeutiques de réanimation :*

**Des traitements ont-ils été arrêtés** : non = 0, oui = 1 l__l **neo_no3**

Si oui,

Retrait de la VNI (VS PEEP ou Infant flow) : non = 0, oui = 1 l__l **neo_no4**

Sevrage d’oxygène : non = 0, oui = 1 l__l **neo_no5**

Déventilation progressive (« terminal weaning ») : non = 0, oui = 1 l__l **neo_no6**

Extubation : non = 0, oui = 1 l__l **neo_no7**

Arrêt des amines vaso-actives : non = 0, oui = 1 l__l **neo_no8**

Arrêt de la nutrition parentérale : non = 0, oui = 1 l__l **neo_no9**

Arrêt de l’alimentation entérale au sein ou biberon (si en cours précédemment) : non = 0, oui = 1,

pas d’alimentation entérale = 2 l__l **neo_no10**

***d_no10*** f

Date de début du processus d’arrêt des thérapeutiques l__...__l **neo_no11**

*Age au début du processus d’arrêt des thérapeutiques (en jours)* ***neo_no11b***

**Des traitements ont été limités** : non = 0, oui = 1 l__l **neo_no14**

Si oui,

Ne pas intuber si besoin : non = 0, oui = 1 l__l **neo_no15**

Ne pas mettre en VNI si besoin : non = 0, oui = 1 l__l **neo_no16**

Ne pas fermer canal chirurgicalement : non = 0, oui = 1 l__l **neo_no17**

Ne pas opérer entérocolite : non = 0, oui = 1 l__l **neo_no18**

Ne pas drainer hydrocéphalie : non = 0, oui = 1 l__l **neo_no19**

Ne pas mettre d’antibiotiques : non = 0, oui = 1 l__l **neo_no20**

Autre : non = 0, oui = 1 l__l **neo_no21**

Si oui, préciser en clair : ………………………………………………………………………… **neo_no22**

Date de début du processus de limitation des thérapeutiques l__... __l **neo_no23**

*Age au début du processus de limitation des thérapeutiques (en jours)* ***neo_no23b***

**Des traitements analgésiques et/ou sédatifs ou autres ont-ils été ajoutés ou augmentés**

**à la suite de la décision de limitation ou d’arrêt de certains traitements** : non = 0, oui = 1 l__l **neo_no26**

Si oui, lesquels :

Benzodiazépines : non = 0, oui = 1 l__l **neo_no27**

Phénobarbital : non = 0, oui = 1 l__l **neo_no28**

Morphiniques : non = 0, oui = 1 l__l **neo_no29**

Thiopenthal : non = 0, oui = 1 l__l **neo_no30**

Curare : non = 0, oui = 1 l__l **neo_no31**

Autre(s) : non = 0, oui = 1 l__l **neo_no32**

Si oui, préciser en clair : …………………………………………………………………………… **neo_no33**

Les doses ont été proportionnées aux besoins de sédation/analgésie : non = 0, oui = 1 l__l **neo_no34**

Si non, a-t-il été nécessaire d’intensifier le traitement : non = 0, oui = 1 l__l **neo_no35**

**Rapport écrit dans le dossier sur la durée de vie de l’enfant et les moyens mis en œuvre** :

non = 0, oui = 1 l__l **neo_no36**

**Date du décès** **de l’enfant**  l__l__l/ l__l__l /l__l__l__l__l **neo_no37**

***Age au décès de l’enfant (en jours) neo_no37b***

**Heure et minute du décès** **de l’enfant**  l__l__l h l__l__l min **neo_no40 neo_no41**

***Age au décès de l’enfant***  *l__l* ***neo_age_dc_rea***

***(enfants décédés en réanimation néonatale N = 413 enfants)***

*1 : Décès entre J0-J2*

*2 : Décès entre J3-J7*

*3 : Décès entre J8-J28*

*4 : Décès ≥ J29*

**Les parents ont-ils été présents au moment du décès** : non = 0, oui = 1,

information non disponible = 2 l__l **neo_no42**

Si oui,

Le père : non = 0, oui = 1 l__l **neo_no43**

La mère : non = 0, oui = 1 l__l **neo_no44**

**Mise dans les bras avant le décès ou décès dans les bras d’un ou des deux parents**:

non = 0, oui = 1 l__l **neo_no45**

**Au moment du décès, l’enfant a-t-il été placé dans une chambre seul** : non = 0, oui = 1 l__l **neo_no46**

***Enfant décédé avant 36 SA d’âge corrigé***  *l__l* ***neo_dc36SA***

***(enfants du tronc commun, variable créée par Héloïse Torchin)***

*0 : Enfant vivant à 36 SA d’âge corrigé*

*1 : Décès avant 36 SA (enfants morts-nés, décédés en salle de naissance ou en néonatologie)*

*Causes du décès (enchaînement des causes suivant le certificat de décès) :*

**Causes d’origine fœtale ou néonatale**

**Cause d’origine fœtale ou néonatale ayant directement provoqué le décès en clair**: ………..**neo_no47**

**Due à** ………………………………………………………………………………………………………….**neo_no48**

**Autre(s) cause(s) associée(s)** : non = 0, oui = 1 l__l **neo_no49**

Si oui, préciser en clair : ……………………………………………………………………………… **neo_no50**

***Causes de décès en réanimation néonatale*** *l__l* ***neo_cause_dc_rea***

***(enfants décédés en réanimation néonatale N = 413 enfants, variable créée par Anaëlle Coquelin)***

*1 : Immaturity*

*2 : Respiratory distress syndrome*

*3 : Necrotizing enterocolitis (NEC)*

*4 : Infection*

*5 : CNS Injury*

*6 : Other*

*7 : Unknown*

*8 : Congenital anomaly*

***(Classification des causes de décès à partir de la classification de l’article du New England : Causes and Timing of Death in Extremely Premature Infants from 2000 through 2011)***

**Causes d’origine obstétricale ou maternelle**

**Cause d’origine obstétricale ou maternelle déterminante de la mort en clair** : ……………… **neo_no51**

**Autre(s) cause(s) associée(s)** : non = 0, oui = 1 l__l **neo_no52**

Si oui, préciser en clair : ……………………………………………………………………………… **neo_no53**

**Une autopsie a-t-elle été (ou va-t-elle être) pratiquée**  l__l **neo_no54**

***d_no54f***

0 : non

1 : oui, résultat non disponible

2 : oui, résultat disponible

Si résultat disponible, préciser  l__l **neo_no55**

***d_no55f***

1 : L’autopsie établit la cause du décès

2 : L’autopsie confirme la cause suspectée du décès

3 : L’autopsie est non informative

**Synthèse**

**Morbidité néonatale sévère en oui/non neo_morbidite**

| Lésions cérébrales sévères (HIV3, HIV4, leucomalacie cavitaire) et /ou bronchodysplasie sévère,  et /ou rétinopathie de stade 3 ou + ou traitement laser et /ou enterocolite ulcéro-nécrosante stade 2 ou 3.  *! variable corrigée : si enfant 32/33/34 SA et pas d’ETF => lesion cérébrale= NON*  *si enfant 32/33/34 SA et pas de recherche de retino => retino= NON* |
| --- |
|  |
|  |
|  |
|  |
|  |
|  |
